# Modelling protein-protein interactions for the design of vaccine chimeric antigens with protective epitopes

**DOI:** 10.1371/journal.pone.0318439

**Published:** 2025-02-10

**Authors:** Marinela Contreras, Marta Rafael, Isidro Sobrino, Consuelo Almazán, Juan J. Pastor Comín, James J. Valdés, Carlos Roberto Prudencio, Daniel Ferreira de Lima Neto, Veniamin A. Borin, Pratul K. Agarwal, Paul D. Kasaija, Rubén Fernández-Melgar, Justus Rutaisire, José de la Fuente

**Affiliations:** 1 SaBio, Instituto de Investigación en Recursos Cinegéticos (IREC), Consejo Superior de Investigaciones Científicas (CSIC), Universidad de Castilla-La Mancha (UCLM)-Junta de Comunidades de Castilla-La Mancha (JCCM), Ciudad Real, Spain; 2 Laboratorio de Inmunología y Vacunas, Facultad de Ciencias Naturales, Universidad Autónoma de Querétaro, Juriquilla, Querétaro, Mexico; 3 Centro de Investigación y Documentación Musical CIDoM-UCLM-CSIC, Facultad de Educación de Ciudad Real, Ciudad Real, Spain; 4 Institute of Parasitology, Biology Centre, Czech Academy of Sciences, České Budějovice, Czech Republic; 5 Immunology Center, Adolfo Lutz Institute, São Paulo, SP, Brazil; 6 Graduate Program Interunits in Biotechnology, University of São Paulo, São Paulo, Brazil; 7 General Coordination of Public Health Laboratories, Health Surveillance Secretariat, Ministry of Health, Brasília, Brazil; 8 Department of Physiological Sciences, Oklahoma State University, Stillwater, Oklahoma, United States of America; 9 High-Performance Computing Center, Oklahoma State University, Stillwater, Oklahoma, United States of America; 10 National Livestock Resources Research Institute (NaLIRRI/NARO), Kampala, Uganda; 11 Department of Veterinary Pathobiology, Center for Veterinary Health Sciences, Oklahoma State University, Stillwater, Oklahoma, United States of America; Beni Suef University Faculty of Veterinary Medicine, EGYPT

## Abstract

Ticks and tick-borne diseases are a growing burden worldwide and vaccines are effective control interventions. Vaccine formulations with tick antigens such as BM86/BM95 (BM) and Subolesin (SUB) have shown reduction in tick fitness and infestation in immunized hosts. However, antigen combination is a challenging approach to improve vaccine efficacy (E) against multiple tick species. Herein, *in silico* and *in music* algorithms were integrated to model BM-SUB protein-protein interactions to apply a quantum vaccinology approach for combining protective epitopes or immunological quantum in the chimeric antigen Q38-95. Cattle immunized with Q38-95 and infested with African blue tick *Rhipicephalus decoloratus* showed an 82% E similar to BM86 and higher than SUB. The immune mechanisms activated in cattle in response to vaccination with Q38-95 were mediated by anti-BM/SUB antibodies that interfered with BM-SUB interactions and through activation of other innate and adaptive immune pathways. The results support modelling protein-protein interactions affecting E to identify and combine candidate protective epitopes in chimeric antigens.

## Introduction

Ticks are a major blood-feeding ectoparasite vector of pathogens affecting public and animal health worldwide [1–5]. However, despite multiple extensive studies, tick-borne diseases and associated pathologies remain underdiagnosed worldwide [2]. It is considered that tick-borne diseases affect over half a million people globally with Lyme borreliosis as the most common disease with estimated seroprevalence of 20.7%, 15.9%, and 13.5% in Central Europe, East Asia, and Western Europe, respectively [2] and total burden of 10.55 disability-adjusted life years (DALY) per 100 000 population in Europe [3]. In animals, cattle show highest tick burden with for example reported to be between 0.922 and 1.0 and causing economic loss of USD 787.63 million/year in India [4]. Only in Uganda, ticks cause annual losses of up to USD 1.1 billion, with 30% calf crop mortality, losses in milk and meat production of USD 174 million and 433 million, respectively, and over USD 77 million for import of chemical acaricides and pesticides [4,5]. Within the One Health perspective, vaccines are a key component for the control of tick infestations and tick-borne diseases [6–9].

The pioneering work with *Rhipicephalus microplus* BM86/BM95 (BM) homologous antigens with more than 99% identity led to the registration and commercialization in the early nineties of the only vaccines for the control of tick infestations [10]. Since then, new protective antigens against multiple tick species such as Subolesin (SUB; also known as 4D8 and Akirin) have been validated for the control of vector infestations and pathogen infection/transmission [9,11]. However, despite recent advances in the identification of new candidate protective antigens, the development of effective, efficacious, and safe vaccines for the control of tick infestations is a major challenge [12–14].

One of the alternatives to face this challenge is the combination of tick and/or pathogen derived protective antigens in a single vaccine formulation [6]. However, the development of multi-antigen vaccine formulations requires innovative approaches to prevent antigen competitions with negative effect on host protective immunity [15–17]. Quantum vaccinology (also known as quantum vaccinomics when combined with omics technologies) is an innovative approach for the design of new protective antigens by identifying and combining protective epitopes or immunological quantum in a chimeric antigen [18,19]. The quantum vaccinology approach arises from the random processes in the immune system supporting quantum immunology and the definition of immune protective epitopes as immunological quantum in allegory to the Einstein´s quantum of light [19].

Multiple methodological approaches based on the characterization of protein-protein interactions and their role in immune response have been developed for quantum vaccinology [9,18,20–24]. *In silico* models of protein-protein interactions are well established but the combination of several models including other innovative approaches such as *in music* model based on the interpretation of amino acid protein sequence as a melodic *continuum* provides support for identified key amino acids [9,18]. Then, vaccine formulations based on chimeric antigens combining the identified protective epitopes may be used to boost protective immune response against multiple antigens [21,25,26].

To further advance in the production of multi-antigen tick vaccines, in this study we focused on the characterization of SUB-BM protein interactions that may interfere with protective immune response to combined antigens vaccine formulation. Multiple innovative methodological approaches were used and integrated to model protein-protein interactions and identify candidate interacting epitopes. The results identified candidate protective epitopes that were combined with a quantum vaccinology approach in chimeric Q38-95 antigen to improve vaccine efficacy for the control of multi-species tick infestations.

## Materials and methods

### Experimental design

**Fig 1:**
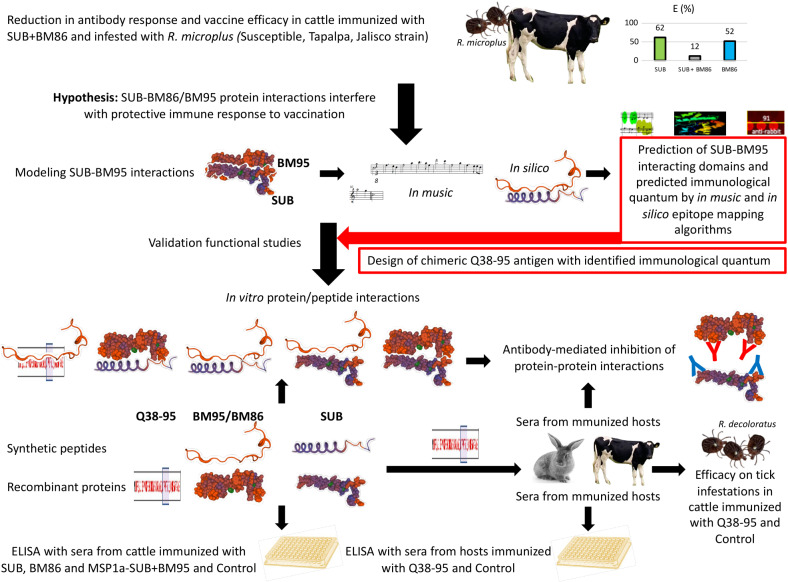
Experimental design. Based on the finding that SUB+BM86 combination in a single formulation reduces E when compared to individual antigens, in this study we approached the hypothesis that protein-protein interactions interfere with protective immune response. Experimental approaches include modelling SUB-BM95 protein interactions using *in silico* and *in music* algorithms, *in vitro* validation of protein/peptide interactions, and efficacy of vaccination with chimeric antigen Q38-95 in cattle infested with *R. decoloratus*.

The experimental design was based on a trial in which BM86, SUB, and MSP1a-SUB-BM86 (SUB+BM86) combination were used for the evaluation of vaccine efficacy (E) on cattle tick infestations and summarized in Fig 1. The results of the trial led to the hypothesis that SUB-BM86 protein interactions interfere with protective immune response to vaccination. To address this hypothesis, the SUB-BM95 interactions were modeled using different *in silico* and *in music* algorithms by different researchers and without interactions between them, *in silico* model 1 by C.R.P. and D.F.L.N., *in silico* model 2 by J.J.V., *in silico* model 3 by V.A.B. and P.K.A., *in music* model by J.J.P.C. and J.F.). The different *in silico* models were conducted by different individuals to reduce bias, ensure cross-validation and robustness of the results. The sequences for SUB (NCBI RefSeq - MT241515.1 *Rhipicephalus appendiculatus* from Uganda) and BM95 (NCBI RefSeq - AF150891.2 *Rhipicephalus (Boophilus) microplus*) were used for analysis. The BM95 (AF150891.2) [27,28] and BM86 (ABY58968.1 *R. (Boophilus) microplus*) [29] proteins are homologs with more than 99% sequence identity and thus used in different experiments as described and defined as BM86/BM95 (BM) when required. The chimeric antigen Q38-95 was designed based on modeled SUB-BM95 interactions and applying a quantum vaccinology approach for epitopes predicted to be involved in protective immune response [30]. The interacting epitopes were compared with SUB protective epitopes and validated by *in vitro* peptide/protein combinations and ELISA using sera from different immunized animal species, cattle and rabbits. Antibody-mediated inhibition of protein-protein interactions was evaluated using sera from rabbits and cattle immunized with Q38-95 and controls. The E of the recombinant antigen Q38-95 was evaluated in cattle infested with the one-host tick, *R. decoloratus*. All recombinant proteins used in different experiments (BM86/BM95, SUB, SUB+BM86, Q38-95) were purified with more than 98% purity and integrity. The expression of selected biomarkers (complement component 3, tumor necrosis factor alpha, interleukin 1 beta, interleukin 2, akirin 2, interleukin 12) was evaluated in blood samples from Q38-95 vaccinated and control cattle before tick infestations.

### Ethics statements

Cattle at the first vaccination trial conducted in Mexico (section “Cattle vaccination, tick infestation, serum collection and determination of antibody levels”) were cared for in accordance with standards specified in the Guide for Care and Use of Laboratory Animals and experimentation was approved by the animal ethical committee of the Faculty of Veterinary Medicine, University of Tamaulipas, Mexico [27]. The other cattle vaccine experiments were conducted at NaLIRRI, Uganda (section “Validation studies”) and experimental animals were treated in accordance with the Ugandan National Council of Science and Technology (UNCT) guiding Principles for Biomedical Research Involving Animals and with the approval of Ugandan National Agricultural Research Organization (NARO) Institutional Animal Care and Use Committee (IACUC No. 2020-0802-20). Experiment with rabbits immunized with Q38-95 (section “Validation studies”) were conducted in strict accordance with the recommendations of the European Guide for the Care and Use of Laboratory Animals at experimental facility (El Chaparrillo, IREC, Ciudad Real, Spain) approved by the Counseling of Agriculture, Environment and Rural Development of Castilla La Mancha (REGA: ES130340000281).

### Cattle vaccination, tick infestation, serum collection and determination of antibody levels

(a) Protein production and purification. Recombinant *R. microplus* SUB and SUB+BM86 proteins were produced in *Escherichia coli* and *R. microplus* BM86 was produced in *Pichia pastoris* [27–29,31] and all proteins were purified with > 98% purity. The recombinant SUB was produced as inclusion bodies denatured with 6M Urea buffer and subsequently purified using a HIS-trap column (Profinia IMAC cartridge, Biorad, California, USA) while BM86 and SUB+BM86 were secreted.
(b) Cattle vaccination and tick infestation. Beef *Bos taurus* cattle (3 animals per group) were intramuscularly immunized with recombinant BM86, SUB, and SUB+BM86 and infested with 10,000 *Rhipicephalus microplus* (Susceptible CENAPA, Tapalpa, Jalisco, Mexico strain) larvae applied individually to each animal in separate cotton cells attached to the back of the cows [27]. Cattle were vaccinated 3 times with 100 μg/2 ml doses (100 μg for each antigen in SUB+BM86 combined vaccine) at weeks 1, 4 and 6 in the neck region with recombinant antigen or control in water in oil adjuvant (Montanide ISA 50V2, Seppic, Paris, France). Negative controls were injected with adjuvant/saline alone. Ticks were obtained from a laboratory colony maintained at the University of Tamaulipas, Mexico, where tick larvae were fed on cows and collected until repletion to allow for oviposition and hatching in humidity chambers at 12 h light: 12 h dark photoperiod, 22–25 °C and 95% relative humidity. Larvae were 15 days of age at the time of infestations. Adult female ticks dropping from each cattle were daily collected, counted, weighted, and assessed for oviposition and egg fertility to calculate E as previously reported [32] (Table 1).
(c) Serological assay. Sera were prepared from blood samples collected 2 weeks after the last immunization and before tick infestation. Serum antibody levels were determined using an antigen-specific indirect ELISA with purified recombinant BM86 and SUB. Antigens (0.1 μg/well) were used to coat ELISA plates overnight at 4°C. Sera were serially diluted to 1:10, 1:100 and 1:1000 (optimum 1:100) in PBST (PBS/0.5% Tween 20, pH 7.2) and 10% fetal bovine serum (Sigma-Aldrich, St. Louis, MO, USA). The plates were incubated with the diluted sera for 1 h at 37 °C and then incubated with 1:10,000 rabbit anti-bovine IgG-HRP conjugates (Sigma) for 1 h at 37 °C. After incubation the plates were washed with PBST. The color reaction was developed with 3,3’,5,5’-tetramethylbenzidine (Sigma) and the OD_450 nm_ was determined. Antibody titers in immunized cattle were expressed as the OD_450 nm_ value and compared between vaccinated and control cattle using a One-way ANOVA test with post-Tukey Honestly Significant Difference HSD (p = 0.05; https://astatsa.com/OneWay_Anova_with_TukeyHSD/).

**Table 1:** Results of the vaccination trial with SUB+BM86 on *R. microplus* cattle tick infestations.

Effect on tick life cycle	Control	SUB	SUB+BM86	BM86
	Values per cow (n = 3) Average ± S.D. Percent reduction vs. phosphate-buffered saline (PBS) control Student’s t-test with unequal variance vs. Control (p-value)			
DT, Effect on the number engorged female ticks	1688, 1400, 1290	677, 983, 832	1285, 1433, 1045	704, 900, 613
	1459 ± 206	831 ± 153	1254 ± 196	739 ± 147
	---	43%	14%	49%
	---	p = 0.008	p = 0.140	p = 0.005
DW, Effect on the weight engorged female ticks (mg/tick)	276, 254, 288	277, 258, 295	243, 255, 224	234, 198, 222
	273 ± 17	277 ± 19	241 ± 16	218 ± 18
	---	0%	12%	20%
	---	p = 0.399	p = 0.038	p = 0.010
DO, Effect on oviposition (mg weight eggs/tick)	109, 121, 98	100, 119, 101	76, 98, 100	92, 88, 113
	109 ± 12	107 ± 11	91 ± 13	98 ± 13
	---	2%	16%	11%
	---	p = 0.392	p = 0.076	p = 0.159
DF, Effect on fertility (weight larvae/weigh eggs)	0.62, 0.53, 0.41	0.32, 0.38, 0.35	0.61, 0.56, 0.58	0.32, 0.39, 0.34
	0.52 ± 0.11	0.35 ± 0.03	0.58 ± 0.03	0.35 ± 0.04
	---	33%	0%	33%
	---	p = 0.049	p = 0.204	p = 0.048
Vaccine efficacy (E)	---	62%	12%	52%

DT = 100 [l-(NTV/NTC)], where NTV is the number of adult female ticks in the vaccinated group and NTC is the number of adult female ticks in the control group. DW = 100 [1-(WTV/WTC)], where WTV is the average adult female tick weight in the vaccinated group and WTC is the average adult female tick weight in the control group. DO = 100 [1-(PATV/PATC)], where PATV is the average weight of the eggs per survived tick in the vaccinated group and PATC is the average weight of the eggs per survived tick in the control group. DF = 100 [1-(PPLOV/PPLOC)], where PPLOV is the average weight of the larvae per gram of eggs in the vaccinated group and PPLOC is the average weight of the larvae per gram of eggs in the control group. E (%) = 100 [l-(CRT × CR0 × CRF)], where CRT = NTV/NTC, CR0 = PATV/PATC and CRF = PPLOV/PPLOC that represent the reduction in the number of adult female ticks, oviposition and egg fertility as compared to the control group, respectively. Only parameters with statistically significant differences between vaccinated and control groups (Student’s t-test with unequal variance; p < 0.05) were included in the E calculation.

### *In silico* model 1

#### Homology modeling.

The sequences for SUB and BM95 were modeled using Modeller (program for comparative protein structure modelling by satisfactions of spatial restraints, https://salilab.org/modeller/; accessed November 2022) by selecting the best structures from the alignments based on structure and loop modeling. Models were then submitted to model validation tools (MolProbity, http://molprobity.biochem.duke.edu and PDBsum Generate, https://bio.tools/pdbsum_generate; accessed in December 2022). Ramachandran two-dimensional (2-D) plots and scoring functions were applied to the best models according to Discrete Optimized Protein Energy (DOPE) results (S1 Fig). The structures were minimized using software suite for high-performance molecular dynamics and output analysis (GROMACS v.2021.3, https://www.gromacs.org; accessed in December 2022) using the steepest descent methodology until forces converged below 0.001 kJ mol^−1^ nm^−1^.

#### Docking.

Following model validations, the structures were prepared for protein-protein docking. We used a two-fold approximation of the problem by running both blind and guided docking indicating the attracting residues in BM95 to be D-268, C-269, R-270, V-271, Q-272, K-273, G-274, T-275, V-276, L-277, C-278, E-279, C-280, P-281, W-282, N-283, Q-284, H-285, L-286, V-287, G-288, D-289, T-290, C-291, I-292, S-293, D-294, C-295, V-296, D-297, K-298, K-299, C-300, H-301, E-302, E-303, F-304, M-305, D-306, C-307, and the interacting residues in SUB to be D-138, T-139, F-140, V-141, K-142, F-143, T-144, Y-145, D-146. We then applied two docking servers to the task (High Ambiguity Driven protein-protein DOCKing, HADDOCK v.2.2, https://milou.science.uu.nl/services/HADDOCK2.2/ and protein-protein docking, ClusPro v.2.0, https://cluspro.org/; accessed in December 2022). The results were selected based on the most favorable interacting energies (S2 Fig). The top cluster is the most reliable according to HADDOCK. Its Z-score indicates how many standard deviations from the average this cluster is located in terms of score (the more negative the better).

#### Protein-protein interface interactions analysis.

To investigate the protein-protein interface interactions, server for analysis of protein interactions in macromolecular assembly (PIMA) was employed (http://caps.ncbs.res.in/pima/; accessed in December 2022). The 2-D representation of the found interactions was generated with ligand-protein interaction diagrams (LigPlot^ +^ v.2.2, https://www.ebi.ac.uk/thornton-srv/software/LigPlus/; accessed in December 2022). Briefly, PIMA calculates various energy terms (electrostatic energy, Van der Waals VDW energy, and total stabilization energy) in kJ/mol and number of interactions (hydrogen bonds, VDW pairs, and salt bridges) along with number of interacting residues on the protein-protein interface.

#### Molecular dynamics.

Molecular dynamics analyses were conducted using the simulation package GROMACS v.2021.3 for the BM95-SUB complex. The ff99SB force field was employed in all the simulations. The TIP3P water model was also used in the simulations. The system was placed in the center of the dodecahedron box, and the minimum distance between the system and the box boundaries was set to 1 nm. Integration of the equations of motion was done at a time step of 2 fs with full periodic boundary conditions (PBC) applied along the three Cartesian directions. The systems were energy-minimized using the conjugate gradient (CG) method, with 1 × 10^6^ (kJ/mol and kJ/mol x nm for energy difference and root mean square (RMS) force, respectively) convergence criteria. Then, a 200 ns constant temperature and volume (NVT) production run (2 ns for wetting calculations) at 300 K was performed using a Berendsen thermostat algorithm with a damping constant of 0.1 ps. During the simulations, a 1.0 nm cut off for Lennard-Jones (LJ) and Coulomb interactions was applied and the particle mesh-Ewald method was used for long-range electrostatics. The LINCS method was used as a constraint algorithm [33]. All the images were generated with Visual Molecular Dynamics (VMD, https://www.ks.uiuc.edu/Research/vmd/) and Xmgrace (https://plasma-gate.weizmann.ac.il/Grace/) (accessed in December 2022).

### *In silico* model 2

#### Structural prediction and molecular docking.

The primary sequence amino-terminus for SUB was truncated (residues 1-113) to parallel the resolved carboxyl-terminus of Akirin2 [34]. The BM95 signal peptide (residues 1 – 19), as predicted by the SignalP server [35], was also truncated from the primary sequence. Both protein models were then individually constructed by the I-TASSER server (https://zhanggroup.org/I-TASSER/) [36]. The predicted I-TASSER tertiary structural pairings [pairings of two predicted tertiary structures for BM95 with five predicted for SUB), S3 Fig] were independently submitted to the HDOCK server (http://hdock.phys.hust.edu.cn/), a protein-protein docking algorithm that merges template-based modeling and *ab initio* free docking [37]. The docking parameters were kept blind (i.e., without indicating binding residues). The top ten resulting docked conformations for each predicted structural pairing (n = 100) were prepared and optimized using the Maestro software package (MacroModel Schrödinger Release 2022-1, Schrödinger, LLC, New York, NY, 2021) [38]. Firstly, the termini were capped, and the hydrogen atoms were replaced. Secondly, local minimizations were performed to remove any steric clashes. Thirdly, the hydrogen-bond networks were optimized with the Protein Preparation Wizard (https://www.schrodinger.com/science-articles/protein-preparation-wizard) [39]. Lastly, the prepared and optimized docked conformations were screened for residue contacts. All images were captured using the Maestro software package. All applications accessed in December 2022.

#### Molecular dynamics.

The extended BM95 termini from the two selected docked conformations were truncated (residues 1 – 165 and 496 – 550) and capped to minimize computational expenditure. The two docked complexes were then individually solvated in a 10 Å^3^ orthorhombic box with a TIP3P water model neutralized [40] and salted with 0.15 M NaCl. The CHARMM36 force field [41] was used to parameterize the docked complexes and ions (approximately 90000 atoms). All molecular dynamics (MD) simulations were conducted using a GPU-accelerated workstation with the Desmond software [42]. The protein model refinement designed by Zhu et al. [43] running 10  ×  5-ns MD simulations, was implemented using the Desmond default protocol. The Desmond protocol has several initial equilibration steps, and a final MD production step conducted under isotropic conditions with an NPT ensemble coupled with a Nose-Hoover thermostat [44] and a Martyna-Tobias-Klein barostat [45]. The temperature was set at 300 K with a RESPA [46] integrator at an inner time step of 2 fs. After refinement, a 500 ns MD was performed for analysis under the Desmond default protocol.

#### Amino acids contact analyses.

The two selected docked conformations were processed with PDBePISA [47] to analyze initial consistencies among residue interactions. The docked complexes and 500 ns MD trajectories were analyzed under the VMD software [48]. Residue interactions were inspected using the H-bond contacts, a VMD plugin. The distance cutoff was set at 3.5 Å with a 90 º angle threshold. Detailed information for residue pairs was included. The centroid of interacting residues with aromatic rings were calculated and the distances measured using the π-π stacking Tcl script (https://doi.org/10.5281/zenodo.6408973). The π-π stacking Tcl script also calculated the dihedral angle formed between the planes of the aromatic pairing.

### *In silico* model 3

#### Model generation.

The individual structures of BM95 (UniProt ID: E1B2Y1) and SUB (UniProt ID: A0A0U2T382) were obtained from using the AlphaFold website (https://alphafold.ebi.ac.uk). Four different BM95-SUB complexes were also generated with AlphaFold using the multimer model [49,50]. As an alternative approach, four complexes were obtained using the HADDOCK 2.4 webserver using the single protein structures generated using AlphaFold [51,52]. The initial structures of BM95 and SUB were first simulated using 1 𝜇s MD simulation, followed by the clustering in UCSF Chimera software (UCSF Chimera--a visualization system for exploratory research and analysis) [53]. The four most populous conformers of each protein were used to generate 16 possible structures of the BM95-SUB complexes using the HADDOCK server. Four complexes with the best HADDOCK scores were used for further MD simulations.

#### Molecular dynamics simulations.

Molecular dynamics simulations were performed for BM95-SUB complexes in explicit water solvent, using a protocol similar to our previous studies [54,55]. Model preparation and simulations were performed using the AMBER v16 suite of programs for biomolecular simulations [56]. AMBER’s *ff14SB* force-fields were used for all simulations [57]. The MD simulations were performed and refined using NVIDIA graphical processing units (GPUs) and AMBER’s *pmemd.cuda* simulation engine using our lab protocols published previously [58,59]. We have verified the suitability of ff14SB for simulations at microsecond timescales for a number of proteins complexes [54,55]. Standard parameters from ff14SB force-field were used for the protein residues and nucleotides. SPC model was used for water [60,61]. The parameters for the counter-ions were used from AMBER, as available in the *frcmod.ionsjc_spce* and *frcmod.ionslrcm_hfe_spce* files.

Starting with the complex structures obtained from AlphaFold and HADDOCK servers, the missing hydrogen atoms were added with the AMBER’s *tleap* program. After processing the coordinates of the protein and substrate, all systems were neutralized by addition of counter-ions and the resulting system were solvated in a rectangular box of SPC/E water, with a 10 Å minimum distance between the protein and the edge of the periodic box. The prepared systems were equilibrated using a protocol described previously. The equilibrated systems were then used to run 1.0 μs of production MD under constant energy conditions (NVE ensemble). The use of NVE ensemble is preferred as it offers better computational stability and performance [62,63]. The production simulations were performed at a temperature of 300 K. As NVE ensemble was used for production runs, these values correspond to initial temperature at start of simulations. Temperature adjusting thermostat was not used in simulations; over the course of 1.0 μs simulations the temperature fluctuated around 300 K with RMS fluctuations between 2–4 K, which is typical for well equilibrated systems. A total of 1,000 conformational snapshots (stored every 1,000 ps) collected for each system was used for analysis.

#### Energy of interaction.

The energy for interactions was calculated as a sum of electrostatic and van der Waals energy between atom pairs. This protocol was previously developed to investigate other protein-substrate systems [64,65].


$$E_{pro-subs}=\sum \left(E_{el}+E_{vdw}\right)$$ (1)

*E*_*el*_ is the electrostatic contribution, *E*_*vdw*_ is the van der Waals term and the summation runs over all atom pairs for the protein-substrate complex. The *E*_*el*_ and *E*_*vdw*_ terms were computed as follows,


$$E_{el}=\frac{q_{i}q_{j}}{\varepsilon (r)r_{ij}}$$

and


$$E_{vdw}=\frac{A_{ij}}{r_{ij}^{12}}-\frac{B_{ij}}{r_{ij}^{6}}$$ (2)

where *q*_*i*_ are partial charges, and *A*_*ij*_*, B*_*ij*_ are Lennard-Jones parameters. These parameters were obtained from the AMBER force field. A distance-dependent dielectric function was used:


$$\varepsilon (r_{ij})=A+\frac{B}{1+k\exp(-\lambda Br_{ij})}$$ (3)

*B* = *ε*_*o*_*-A*; *ε*_*o*_ = 78.4 for water; *A* = -8.5525; *λ*= 0.003627 and *k* = 7.7839

All BM95 and SUB atom pairs were included in the calculations and resulting interaction energies were summed up per residue pair. The energies were calculated for 1,000 snapshots, every 1 ns, sampled during the full 1.0 μs simulation and were averaged over these 1,000 snapshots.

### *In music* model

#### Data sonification.

The algorithm used to translate protein sequence into musical scores (Ms.) was previously described and validated [22,66,67]. Each codon either a constant base of a note on which one or two more notes may follow, or a base of two musical notes followed by one or two more. We provided a ternary structure (three beats of a quarter note) and a binary subdivision measure (3/4), where the base of a single note occupies the entire measure (UGC = *B*, dotted-half-note, and the base of two notes is expressed as a half-plus-quarter-note (CAA = *EF*). If the base is a single note accompanied by others, we always chose to extend the base in the first two beats of the measure as half-note when it had the succession of a single note (UCG = *GD* half-plus-quarter-note) or two notes (UCC = *GED*, half-plus-two-eighth notes). If the base is of two notes, a different metrical scheme was stablished for its expression, dotted-quarter-note-plus-eighth-note followed by a quarter-note (GCG = *CDD*) or by two eighth-notes (GCC = *CDED*). When the algorithm provided the same melodic formula for a double base (GGA = *SL*, GGC = *SLDR*) and single base (UCA = *S*; UCU = *SL*), the metric scheme half-plus-quarter-note in the first case and dotted-quarter-note-plus-dotted-quarter-note in the second case was selected. In this way, each codon has a unique rhythmic and melodic definition.

#### *In silico* modeling based on *in music* model.

*In silico* modeling based on results of *in music* model were constructed using Swiss-Model (https://swissmodel.expasy.org), I-TASSER-MTD (https://zhanggroup.org/I-TASSER-MTD/) and Fupred contact map-based domain partition (https://zhanggroup.org/FUpred/), all accessed in November 2022.

### Validation studies

#### Sequence alignment.

Protein sequences for SUB (MT241515.1) and BM95 (AF150891.2) were aligned using the EMBOSS Needle optimal global alignment of two sequences using the Needleman-Wunsch algorithm (https://www.ebi.ac.uk/Tools/psa/).

#### Production of recombinant proteins and vaccine formulation.

Consensus protein epitope sequences selected based on different models as involved in SUB-BM95 interactions (SUB: 130-STKLAEQYDTFVKFTYDQIQKRFEGATPSYLS-161, BM95: 270-RVQKGTVLCECPWNQHLVGDTCISDCVDKKCHE-302) were chemically synthesized with Keyhole Limpet Hemocyanin (KLH) conjugation on C-terminal and more than 95% purity (Synpeptide Co., Ltd, Shanghai, China). Additionally, a quantum vaccinology approach was applied to combine SUB Q38 protective chimera with candidate BM95 consensus epitopes interacting with SUB and thus likely involved in protective immune response. The resulting chimeric antigen Q38-95 (NH2-MACATLKRTHDWDPLHSPNGRSPKPSPFGEVPPKSSPLESGSPSATPPASPTGLSPGGLLSPVRRDQPLFTFRQVGLICERMMKERESQIRDEYDHVLSAKLAEQYDTFVKFTYDQIQKRFEGATPSYLSgggsHKPFGSPSSPSSSAIAAAAAAAKRPSPFAEAVCPKQLTFNTGSRPDSPPSMVLFTFKQALREQYDAVLTNKLAEQYDAAAPSYLSgggsRVQKGTVLCECPWNQHLVGDTCISDCVDKKCHE-COOH; the position of GGGS linkers is shown in lowercase letters) was produced for vaccine formulation. Recombinant Q38-95 chimera was produced from a synthetic gene (GenScript, Rijswijk, Netherlands) optimized for codon usage and expression in *E. coli* using pET-21a (+) vector (cloning site NdeI/XhoI) and purified using Ni affinity chromatography using 1 ml HisTrap FF columns mounted on an AKTAgo–FPLC system (Cytiva, Marlborough, MA, USA) in the presence of 7 M urea lysis buffer [18]. The eluted fraction containing the purified proteins was dialyzed against 1000 volumes of PBS (137 mM NaCl, 2.7 mM KCl, 10 mM Na_2_HPO_4_, 1.8 mM KH_2_PO_4_), pH 7.4 for 12 h at 4 ºC. Recombinant proteins were then concentrated using an Amicon Ultra-15 ultrafiltration device (cut off 10 kDa) (Millipore-Merck, Darmstadt, Germany), and adjusted to 0.20 mg/ml. For vaccine formulation, recombinant proteins were adjuvated in Montanide ISA 50 V2 (Seppic, Paris, France).

#### Modelling Q38-95 and analysis of predicted B-cell, T-cell and conserved protective epitopes.

Modelling of chimeric Q38-95 antigen was conducted using Swiss-Model (https://swissmodel.expasy.org). Predicted T-cell epitopes (MHC I and MHC II) were determined using MHCPred v.2. (http://www.ddg-pharmfac.net/mhcpred/MHCPred/) with IC_50_ value (nM) confidence of prediction max = 1. For continuous B-cell epitopes, prediction was conducted with Bcepred Prediction Server of continuous B-cell epitopes in antigenic sequences using physico-chemical properties (https://webs.iiitd.edu.in/raghava/bcepred/bcepred_submission.html) with Flexi parameter respective threshold = 2. The analysis of Q38-95 protective epitopes was conducted using UniProt CLUSTAL O(1.2.4) (https://www.uniprot.org, accessed on Feb 4, 2024) tools (a) peptide search (https://www.uniprot.org/peptide-search) for Q38 SAKLAEQYDTFVKFTYDQIQKRFEGATPSYLS and BM86/BM95 RVQKGTVLCECPWNQHLVGDTCISDCVDKKCHE consensus sequences, and (b) align (https://www.uniprot.org/align/) for SUB SAKLAEQYDTFVKFTYDQIQKRFEGATPSYLS in tick species. The Q38-95 self-interaction was modeled with AlphaFold3 and AlphaFold-multimer v2.3 using 20 recycles and 3 seeds (https://www.alphafoldserver.com).

#### *In vitro* analysis of protein/peptide interactions.

The peptide/recombinant protein combinations produced were between (a) SUB and BM86 proteins, (b) BM95 (pBM95) and SUB (pSUB) peptides, (c) BM86 protein and pSUB, (d) BM86 protein and pBM95, (e) SUB protein and pSUB, (f) SUB protein and pBM95, (g) Q38-95 protein and pBM95, and (h) Q38-95 protein and pSUB. For antigen combinations, equal molar ratios of each protein or peptide were incubated on a Stuart SB3 tube rotator (Richmond Scientific Ltd., Chorley, UK) at 4 °C overnight.

#### ELISA using sera from immunized cattle.

Pooled sera from crossbred cows collected 15 days after the third immunization and before tick infestation were obtained from not immunized control cattle (n = 3) and cattle immunized with MSP1a-SUB-BM95 (SUB+BM95; n = 6), BM86 (n = 8) or SUB (n = 5) recombinant proteins and tested with antigen-specific indirect ELISA against the 8 peptide/recombinant protein combinations (a–h) described above and the individual recombinant proteins BM86 and SUB, pSUB and pBM95 and the Q38-95 chimeric antigen. The plates were coated with 0.1 μg per well and incubated overnight at 4 °C with gentle shaking. Plates were then washed once with 100 μl/well PBST and blocked with 100 μl/well of blocking solution (2.5% of skimmed milk in PBS) at room temperature (RT) with gentle agitation for 1 h. Plates were then washed three times with PBST, and bovine serum samples were added at 1:100 dilution in blocking solution and incubated for 1 h at 37 °C. Plates were washed three times with PBST and 1:10,000 dilution of rabbit anti-bovine IgG-HRP conjugate (Sigma) was added to each well. The plates were incubated for 1 h at RT with gentle shaking and after three washes with PBST, 100 μl/well of TMB One Solution (Promega, Madison, WI, USA) were added and incubated for 5–10 min at RT with agitation in dark. Reactions were stopped with 50 μl/well of H_2_SO_4_ and absorbance was measured in a spectrophotometer at O.D.450nm. Antibody titers were compared between the different peptide/protein interactions for each serum plate using a One-way ANOVA test with post-Tukey Honestly Significant Difference (HSD) (p = 0.05) using Real Statistics Resource Pack (Release 8.6.3) from Microsoft Excel.

#### Immunization of rabbits with Q38-95.

Two rabbits were subcutaneously immunized with 0.5 ml vaccine formulation containing 50 µg of the recombinant antigen Q38-95 in Montanide ISA 50 V2 (Seppic) per dose [23]. Serum samples were obtained from whole blood by centrifugation at 2,500 x g for 15 min in samples collected before the first immunization (T0), before the second immunization 22 days after the first immunization (T1), and 33 and 49 days after the first immunization (T2 and T3, respectively).

#### ELISA using sera from Q38-95-immunized rabbits.

Sera from immunized rabbits were tested using antigen-specific indirect ELISA plates coated with the recombinant proteins SUB, BM86 and Q38-95. Plates were coated with 0.1 μg antigen per well and incubated overnight at 4 ºC with gentle shaking. Then, plates were washed with 100 μl/well of PBST, followed by blocking with 100 μl/well of blocking solution (2.5% of skimmed milk in PBS) at RT with gentle agitation for 1 h. Plates were then washed three times with the washing solution, and rabbit serum samples were added at 1:100 dilution in blocking solution and incubated for 1 h at 37 °C. After three washes with the washing solution, a 1:10,000 dilution of anti-rabbit IgG peroxidase from goats (Sigma) was added to each well and incubated for 1 h at RT with gentle shaking. Plates were washed three times with the washing solution, and 100 μl/well of TMB One Solution (Promega) was added and incubated for 5–10 min at RT with agitation in the dark. The reaction was stopped by adding 50 μl/well of H_2_SO_4_, and the absorbance was measured in a spectrophotometer at O.D._450 nm_. Antibody titers were analyzed by comparing the mean of the duplicate values between T3 and T0 by Student’s t-test with unequal variance and for T3-T0 by one-way ANOVA with post-hoc Tukey HSD (https://astatsa.com/OneWay_Anova_with_TukeyHSD/) (p = 0.05; n = 2 for each group).

#### Antibody-mediated inhibition of SUB-BM95 protein-protein interactions.

Experiments were conducted using sera from rabbits and cattle immunized with Q38-95 and controls. For protein-protein interactions using rabbit sera, equal molar ratios of each protein and anti-Q38-95 antibodies were incubated overnight at 4 °C on a Stuart SB3 tube rotator (Richmond Scientific Ltd.) as described above for *in vitro* analysis of protein/peptide interactions. The quality of anti-Q38-95 antibodies was assessed by comparing its specificity in plates coated with Q38-95 or *E. coli* protein extract after antigen purification by Ni affinity chromatography to discard unspecific binding. Ten μg of each interaction were loaded onto a 12% SDS-polyacrylamide pre-cast gel (Genscript Corporation, Piscataway, NJ, USA) and transferred to a nitrocellulose membrane. Membranes were blocked with 5% bovine serum albumin (BSA) (Sigma-Aldrich, St. Louis, MI, USA) for 1.5 h at RT and washed three times with tris-buffered saline (TBS)T (50 mM Tris-Cl, pH 7.5, 150 mM NaCl, 0.5% Tween 20). Purified IgG antibodies from rabbits immunized with recombinant SUB or BM86 were used as primary antibodies at 1:500 and 1:200 dilutions in TBS, respectively. Membranes were incubated overnight at 4 °C and then washed four times with TBST. Then, membranes were incubated with an anti-rabbit IgG-horseradish peroxidase (HRP) conjugate (Sigma-Aldrich) diluted 1:1000 in blocking solution (TBS with 3% BSA). Membranes were washed four times with TBST and finally developed with ECL western blotting substrate kit (Thermo Fisher Scientific, Waltham, MA, USA) according to the manufacturer’s recommendations. For cattle sera, a quantitative analysis was conducted by ELISA as described for rabbits but using sera from immunized cattle in the proof-of-concept study described below. The ELISA plates were coated with no protein (negative control), 0.1 μg SUB (positive control) and 0.1 μg BM86 per well and incubated overnight at 4 °C with gentle shaking. Plates were then incubated with none or an equal molar concentration of pSUB (6 ng) and anti-Q38-95 antibodies (0 to 0.2 μg). Antibody titers against SUB were compared between the different treatments by one-way ANOVA with post-hoc Tukey HSD (https://astatsa.com/OneWay_Anova_with_TukeyHSD/) (p = 0.05; n = 9 for each treatment).

#### Vaccine efficacy of recombinant Q38-95 antigen.

The Q38-95 E was evaluated in a proof-of-concept preliminary experiment with cross-bred *Bos indicus* x *Bos taurus* (local Ankole x Viking Jersey) cattle infested with *R. decoloratus*. Before the experiment, cattle were assessed for general health status, and selected cattle were those with no previous exposure to ticks and tick-borne diseases, particularly anaplasmosis, theileriosis and babesiosis as previously described [68]. Eight healthy male (n = 4) and female (n = 4) 7 to 10 months calves were selected and randomly distributed with gender balance between vaccinated and control groups. Animals were housed individually in arthropod-free, well-ventilated isolation pens and fed on fodder and 20% protein concentrate, receiving water *ad libitum*. Cattle were vaccinated with 100 µg Q38-95 in 1 ml vaccine formulation produced as described above. Control animals were treated with PBS in Montanide ISA 50 V2 (Seppic). Treatments were injected in the neck muscles using a 5 ml syringe and needle. The first dose was administered on day zero (T0) and the second dose on day 30 (T1). Pathogen-free colonies of *R. decoloratus* were prepared from ticks previously collected in Uganda’s agro-ecological zones as previously described [68]. Tick laid eggs were incubated in humidity chambers at 12 h light: 12 h dark photoperiod, 20 ˚C and 95% relative humidity for hatching. Fifteen days after hatching, the emerging larvae were used for challenge at day 45 (T2) with 300 *R. decoloratus* larvae per animal applied on the back and held in place by a glued bag with a zipper. Adult engorged female ticks dropping off the animal were collected daily at days 73 to 89, counted and weighed. All collected ticks were incubated and assessed for oviposition (egg mass/tick) and egg fertility (weight of larvae/tick). Vaccine efficacy, E (%) = 100 [l - (CRT x CRO x CRF)], where CRT, CRO and CRF are the reduction in the number of adult female ticks, oviposition and egg fertility compared with the control group was calculated as described above (Table 1) and previously reported [31,68].

#### Analysis of antibody response and expression of selected biomarkers in Q38-95 immunized cattle.

Blood samples were collected from vaccinated and control cattle at T0 (before first treatment), day 30 (T1) (before second treatment), day 45 (T2) before tick infestations and day 100 (T3) at the end of the trial. Antibody response against SUB, BM86 and Q38-95 were characterized by ELISA in cattle sera as described above in rabbits. Total RNA was extracted from blood samples collected at T0, T1 and T2 using TRI Reagent (Sigma-Aldrich, St. Louis, USA), following the manufacturer’s instructions and used for quantitative real-time polymerase chain reaction (qRT-PCR) analysis using the Quantitect SYBR Green RT-PCR Kit and SYBR Green Master Mix (Bio-Rad, Applied Biosystems, Waltham, USA) in a Rotor Gene Q thermocycler (Qiagen, Inc. Valencia, CA, USA) with forward (F, 5´-3´) and reverse (R, 5´-3´) primers and annealing temperature of the selected genes involved in immunity, complement component 3 (*C3*; NM_001040469.2, F: ATTGCCAGGTTCTTGTACGG and R: GTCACTGCCTGATTGCAAGA, 56°C), tumor necrosis factor alpha (*tnf-α*; AF348421, F: CCTCACCCACACCATCAG, and R: GCGATCTCCCTTCTCCAG, 54 °C), interleukin 1 beta (*il-1β*; NM174093, F: TCAGAATGGAAACCCTCTCTC and R: GCATGGATCAGACAACAGTG, 56 °C), interleukin 2 (*il-2*; NM180997, F: AAGTGAAGTCATTGCTGCTG and R: TGTCCATTGAATCCTTGATCTC, 54 °C), akirin 2 (*akr2*; NM_001110087.1, F: CATTTATGGGCTGCCTTGTT and R: TGCACAGCTTCTACCACGAC, 54 °C), and interleukin 12 (*il-12*; U11815.1, F: TCTGGACACTTCACCTGCTG and R: TGCACAGCTTCTACCACGAC, 58 °C) (33, 36). Each PCR reaction had two technical replicates and two negative controls per sample. The mRNA Ct values were normalized against *Bos taurus ß-actin* (AY141970.1, F: GGCCGAGCGGAAATCG and R: GCCATCTCCTGCTCGAAGTC, 52 °C) using the 2^− ΔΔCt^ method for relative quantification. Values were compared between control and vaccinated groups at T0, T1 and T2 by one-way ANOVA with post-hoc Tukey HSD (https://astatsa.com/OneWay_Anova_with_TukeyHSD/) (p = 0.05; n = 3 for each group). Antibody response against SUB, BM86 and Q38-95 were characterized by ELISA as described above in rabbits.

## Results

### Vaccine efficacy with combined SUB and BM86 antigens

**Fig 2:**
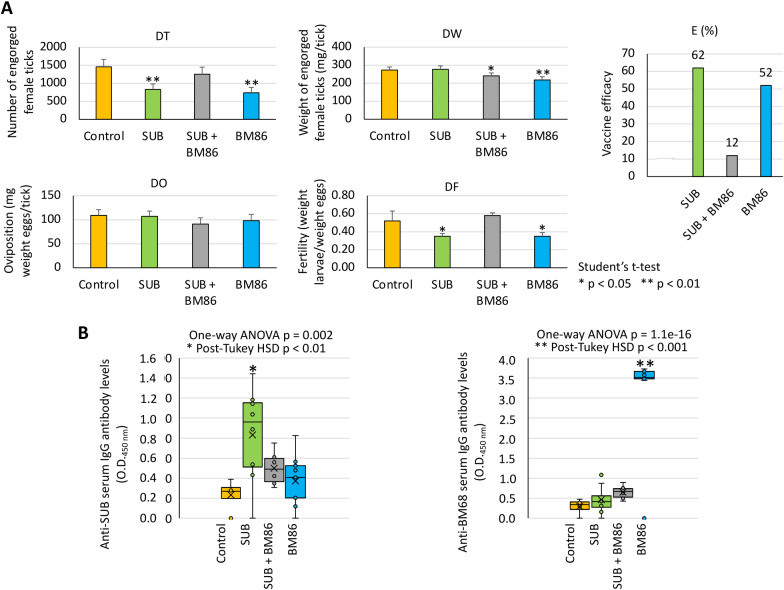
Interference in protective immune response to combined SUB+BM86 vaccination. Cattle were vaccinated with SUB, BM86 or SUB+BM86 combination and compared to control animals treated with adjuvant/saline alone. (A) Effect of vaccination on cattle tick infestations (*p < 0.05, **p < 0.01; Student’s t-test, n = 3 animals per group). (B) Antibody titers determined by ELISA against vaccine antigens (*p < 0.01, **p < 0.001; One-way ANOVA test with post-Tukey HSD, n = 3 animals per group).

A vaccination trial was conducted to evaluate the efficacy on *R. microplus* cattle tick infestations of SUB+BM86 combination and individual antigens in comparison with control animals treated with adjuvant/saline alone (Table 1). The results showed that vaccination with SUB significantly (p < 0.05) reduced DT and DF with 62% E (Fig 2A). Vaccination with BM86 significantly (p < 0.05) reduced DT, DW and DF with 52% E (Fig 2A). In comparison, SUB+BM86 antigen combination only significantly (p < 0.05) reduced DW with 12% E (Fig 2A). As expected, antibody titers were higher for SUB and BM86 in SUB and BM86 vaccinated cattle (p < 0.01) when compared to controls (Fig 2B). However, in cattle vaccinated with the SUB+BM86 antigen combination, both anti-SUB and anti-BM86 antibody titers were not different from control animals (Fig 2B). These results correlated with E (Table 1) and suggested a possible interaction between SUB and BM86 that may affect vaccine protective immunity against antigen combination.

To approach this hypothesis, the interaction between SUB and BM was modelled using different independent model algorithms with *Rhipicephalus appendiculatus* SUB and *R. microplus* BM95 protein sequences.

### *In silico* model 1

**Fig 3:**
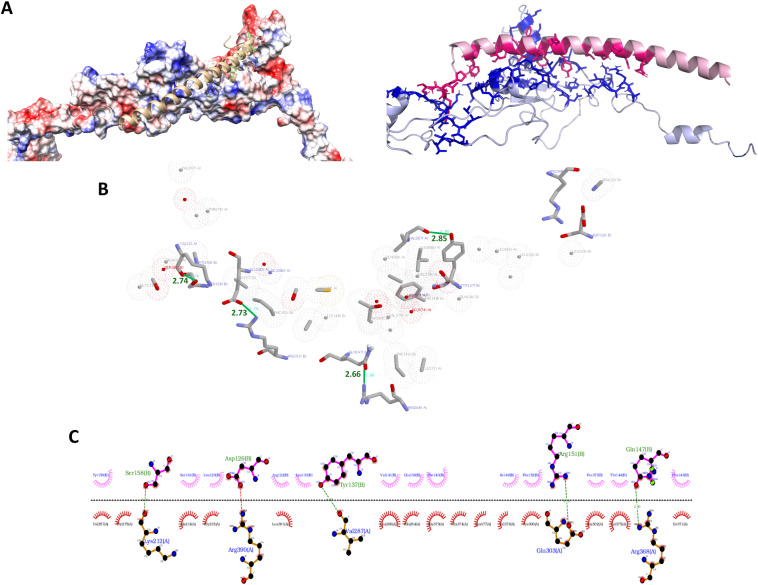
*In silico* model 1. Modeling the interactions between SUB and BM95. (A) Pymol representation of the docked complex between SUB (red) and BM95 (blue). Interacting residues are represented in sticks and the remainder of the proteins in cartoon. APBS (https://www.poissonboltzmann.org) without the appropriate range in PyMol generates these fluctuations. Key residues contributing to SUB-BM95 interaction are disclosed in S2 Table. (B) RasMol representation of the interacting residues. Hydrogen bonds are represented in green with its respective distances. Chain A represents the protein BM95 and chain B represents SUB. (C) LigPlot+ representation of the interacting residues. Hydrogen bonds are represented in green with its respective distances. Chain A represents the protein BM95 and chain B represents SUB.

For homology modelling, protein structures passed the quality control based on Ramachandran evaluations for SUB and BM95 with more than 80.0% of the sequences in most favored regions (S1 Fig). For analysis of protein docking, the representation of the docked complex (blind docking) between SUB (red) and BM95 (blue) showed interacting residues and hydrogen bonds (Fig 3A–3C, S1 Table). The interactions included hydrogen bonds between amino acid pairs (A - BM95, B - SUB), Lys212(A)-Ser158(B) [2.74 Å], Val287(A)-Tyr137(B) [2.85 Å], Glu303(A)-Arg151(B) [2.73 Å], and Arg368(A)-Gln147(B) [2.66 Å]. Hydrophobic interactions were formed between amino acid pairs Val257-Tyr159(B), Thr275(A)-Tyr159(B), Ala214(A)-Ser161(B), Gly215(A)-Leu129(B), Leu381(A)-Arg122(B), Leu381(A)-Leu133(B), Leu286(A)-Val141(B), Gln284(A)-Gln136(B), Glu379(A)-Phe(140(B), Glu374(A)-Phe140(B), Lys377(A)-Phe140(B), Val378(A)-PHe-140(B), Cys300(A)-Ile148(B), Cys300(A)-Phe152(B), Glu302(A)-Pro157(B), Val375(A)-Thr144(B), Ile371(A)-Phe143(B). The results for the guided docking showed BM95-SUB pairwise interactions (S2 Table). The predicted BM95 residues interacting with SUB reference sequence are D-268, C-269, R-270, V-271, Q-272, K-273, G-274, T-275, V-276, L-277, C-278, E-279, C-280, P-281, W-282, N-283, Q-284, H-285, L-286, V-287, G-288, D-289, T-290, C-291, I-292, S-293, D-294, C-295, V-296, D-297, K-298, K-299, C-300, H-301, E-302, E-303, F-304, M-305, D-306, C-307 and the interacting SUB amino acids with BM95 are D-138, T-139, F-140, V-141, K-142, F-143, T-144, Y-145, D-146. These amino acids were used as attracting residues in the input files at the HADDOCK2.2 webserver that clustered 156 structures in 8 clusters, which represent 78% of the generated water-refined models (S3 Table). The best result from the guided docking clusters was then used for the subsequent analyses at the PRODIGY website to predict the number of Interfacial Contacts (ICs) per property guided docking (S4 Table).

The results of molecular dynamics are disclosed in S2 Fig. The root main square deviation (RMSD) of both BM95 and SUB stabilized after 10 ns of simulation, and small variations occurred in the subsequent 10 ns but with intervals of 0.6 nm only in the case of BM95. The disorganized regions at the end of SUB had great variation in the beginning of the simulation (burn-in) and were stable on the rest of the production. The RMSD of BM95 showed greater fluctuations in the C-terminal region of the protein in the range of 0.5 to 1 nm, while SUB also presented greater fluctuations in the N and C-terminal regions in accordance with modeled variations in stability due to the disorganized conformation of these regions. The minimum distance between the alpha (BM95) and beta (SUB) chains varied from 0.15 nm to 0.17 nm over the course of the last 10 ns of the simulation in terms of the closest residues and taking the alpha chain as a reference to the distance. The minimum interchain range ranged from 0.15 to 2 nm between residues 100 to 400 of BM95. The radius of gyration of the proteins showed no evidence of decompression during the simulation and the secondary structure of BM95 and SUB remained stable. However, in the last 10 ns of simulation, SUB showed a tendency of decreasing residues in alpha-helix conformation to the emergence of more turns in its secondary structure. Finally, the network of hydrogen bonds formed was stable at 10 in the remaining 10 ns, fluctuating to 12 and 13 in the last 5 ns.

### *In silico* model 2

The analysis of initial π-π stacking upon BM95-SUB binding was conducted. The SUB carboxyl-terminus contains the residues integral for binding specificity of the BM95 surface protein (S3A–S3D and S4 Figs). The structural prediction of SUB and its high conservation with the resolved carboxyl-terminus structure of Akirin 2 was demonstrated (S3A and S3B Figs). However, due to the low homologous conservation of BM95, the DALI server (http://ekhidna2.biocenter.helsinki.fi/dali/) was subsequently employed to identify consistencies among the BM95 predictions against resolved structures from the Protein Databank (PDB; https://www.rcsb.org/?ref=nav_home). Two predicted BM95 structures posed a 20% sequence conservation, with a global structural homology average of 1.8 Å to the human delta-like 1 cell-surface protein of the Notch family (S3D Fig). Each predicted structure for BM95 (n = 2) and SUB (n = 5) thus served as initial conformations for molecular docking (S3C–S3D Figs). Interactions between the integral binding residues (S3A and S3B Figs) for BM95 (amino acids 249 – 288) and SUB (amino acids 138 – 146) were the main regions for interpreting the molecular docking results.

**Fig 4:**
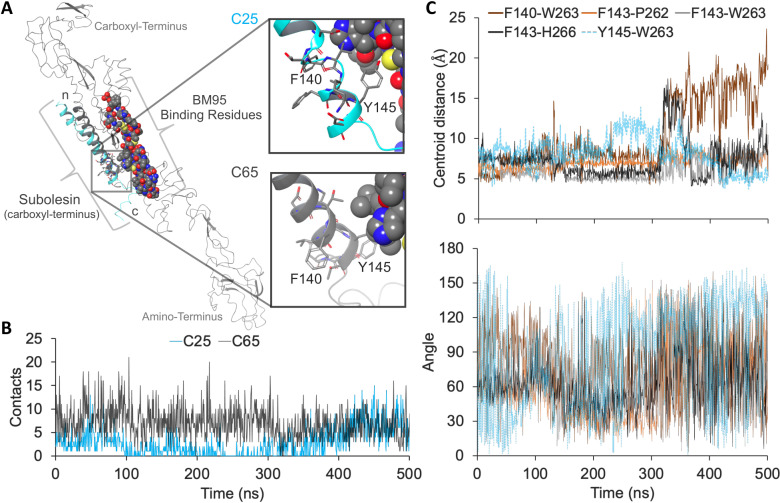
*In silico* model 2. (A) The tertiary structures of the top two docked conformations depicting integral residues of BM95 (spheres) with SUB (sticks). The SUB amino- (n), carboxyl-termini (c), F140, and Y145 (insets) are labeled. (B) Total residue contacts (y-axis) maintained during the 500-ns MD simulation (x-axis). (C) Potential π-π stacking during the 500-ns MD simulation (x-axis) illustrating the centroid distances of interacting aromatic rings (y-axis; upper panel) and the dihedral angles between the aromatic planes (y-axis; lower panel). The potential π-π interaction for the C25 conformation is indicated in dashed cyan.

More than a quarter of the docked conformations (n = 27) form integral BM95-SUB residue contacts (S4 Fig). These structures were subsequently screened for comparable criteria regarding proximity of the bound SUB conformations and of the integral binding residues (S3A and S3B Figs). The screening resulted in two docked conformations with their SUB structures deviating 22.3 Å (named herein as, C25 and C65), respective to the BM95 (Fig 4A). The orientation of the SUB termini for both selected conformations are anti-parallel to the BM95 termini (Fig 4A). This orientation resulted in SUB integral residues approximating 260 ~ 270 BM95 residues. A PDBePISA analysis indicated that SUB F140 and Y145 amino acids coincide as BM95 interacting residues for both conformations (Fig 4A). Merely 15% of the docked structures deviated more than 50 Å from the two selected conformations, whereas 60% averaged 40.9 ± 6.8 Å (S4 Fig). The remaining 25% approximated both docked conformations (Fig 4A) with a deviation range of 3.4 ≤ *x* ≤ 24.9 Å (S4 Fig).

A 500-ns molecular dynamics (MD) indicated that in the selected conformation, C65 forms more integral contacts than C25 (Fig 4B). However, the integral contacts for C25 increased during the last 100 ns of MD (Fig 4B). For C25, SUB residues T144 – D146 mainly interacted with BM95 residues E260 – W263 and L267 – T271. This approximation was illustrated by the decreased centroid distance between C25 SUB residue Y145 and BM95 W263 during the last 100-ns (Fig 4C). On the other hand, for C65, SUB residues T139 – T144 mainly interact with BM95 residues C259 – H266 for 17 ≤ *x* ≤ 64% of the MD simulation. The more stabilized C65 contacts were those potentially forming π-π interactions between SUB residue F143 (average distance) and BM95 residues P262 (7.0 ± 0.8 Å), W263 (6.3 ± 1.4 Å) and H266 (7.3 ± 2.3 Å) (Fig 4C). The C65 centroid distances for the aromatic residue pairing of F140-W263 were also stable (10.4 ± 4.4 Å) but increased for the last 200-ns. The average dihedral angle between the C65 aromatic pairing were 77.3 ± 31.6 ° (F140-W263), 62.5 ± 30.8 ° (F143-P262), 69.5 ± 24.0 ° (F143-W263), and 77.3 ± 31.6 ° (F143-H266). The average dihedral angle for C25 Y145-W263 was 86.2 ± 47.2 ° (Fig 4C).

Aromatic residue moieties form face-to-face, T-shaped, and offset π-π stacked conformations via non-covalent interactions. As examined by Zhao et al. [69], structurally resolved, aromatic π-π interactions prefer centroid distances ≤ 7.2 Å. At these distances, moieties conform as T-shaped if aromatic dihedral angles are 50 ≤ *x* ≤ 70 °. Aromatic dihedral angles 30 ≤ *x* ≤ 50 ° are in an intermediate phase with potential parallel conformations (face-to-face or offset) [69]. Aromatic interactions between phenylalanine and proline were experimentally observed almost 50 years ago [70], although at a lower propensity than for tryptophan or tyrosine [71]. The probability of a non-canonical, tripartite aromatic interaction, or π-π-π stacking, has been demonstrated in the yeast histone acylation reader, YEATS domain [72].

### *In silico* model 3

**Fig 5:**
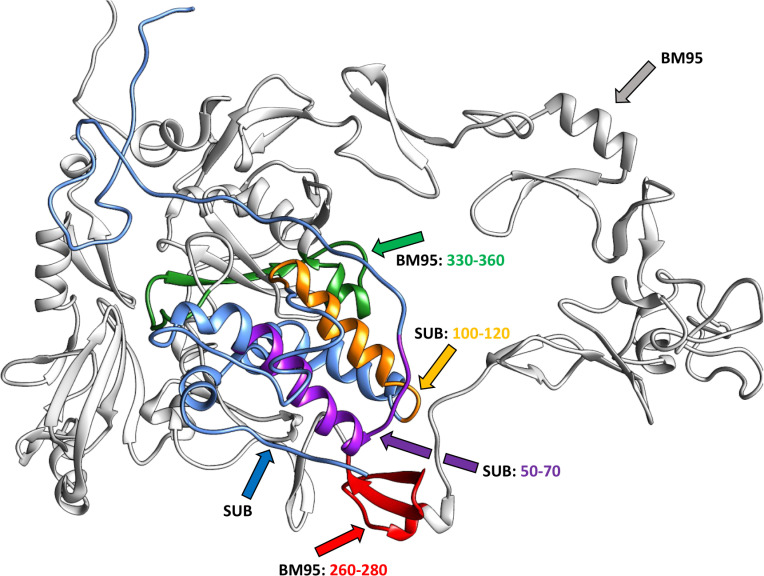
*In silico* model 3. BM95-SUB complex with most probable interacting region highlighted in color. Compiling results from Alphafold and Haddock identify critical regions involved in protein interactions.

The computer simulations were used to generate alternative models of BM95-SUB complexes. Molecular dynamics simulations were used to characterize the interaction between the two proteins. Using protein-protein interaction energy analysis, we identified the regions of the proteins that show the largest probability of interactions. The interaction energies are based on summation of the van der Waals and electrostatic energy between the different residue pairs. We have successfully used this analysis for identifying the regions of interaction between protein complexes [64,65]. The BM95-SUB regions are summarized in Fig 5 and full details are provided in the supporting information (S1 Data).

### *In music* model

It should be noted that the amino acid sequence can be interpreted as a melodic *continuum*. These long melodic lines can be analyzed in terms of long values involving cadential places, which allow the structuring of certain “melodic rests”, the reiteration of melodic motifs or the recurrence of similar pitches or tunes. For this reason, it is important to look at how each of the sequences can be analyzed musically as melodically structured lines (S5 Fig).

**Fig 6:**
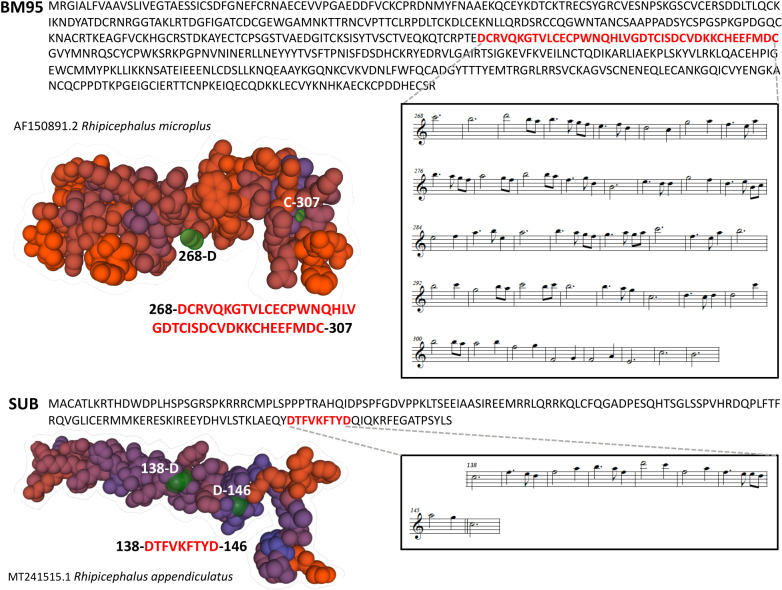
*In music* model. Predicted protein interactions based on coherent Ms. Protein melodic lines corresponding to identified interacting protein regions are shown.

We structured the SUB melodic line (musical scores Ms. 161) in 8 distinct parts, characterized by the insistence in each segment on a characteristic pitch (S2 Data). The BM95 melodic sequence is much broader (Ms. 569) and more difficult to segment when compared to SUB (S2 Data). However, there is a basic fact that allows us to understand sections to the ear by establishing long recurring values as cadential or resting points. This coincides with the amino acid Cys (UGC) expression, *S*/ pitch (*B*). This appears as a recurrent point with a regularity. Where this regularity disappears, there is a greater complexity of motifs whose recurrence gives coherence to the musical discourse. The melodic structure of the SUB-BM95 interaction was very coherent (i.e., leading thread for the listener to follow as the piece progresses) and protein interactions based on coherent Ms. were identified for measures 138–146 in SUB and measures 268–307 in BM95 (Fig 6; additional information in S2 Data and S1–S5 Audio files). The model shows strand coil (BM95) and helix (SUB) predicted secondary structures for identified interacting regions with high confidence score and variable solvent accessibility (Figs 5 and S6).

### Summary of integrated modelled SUB-BM95 interactions

**Fig 7:**
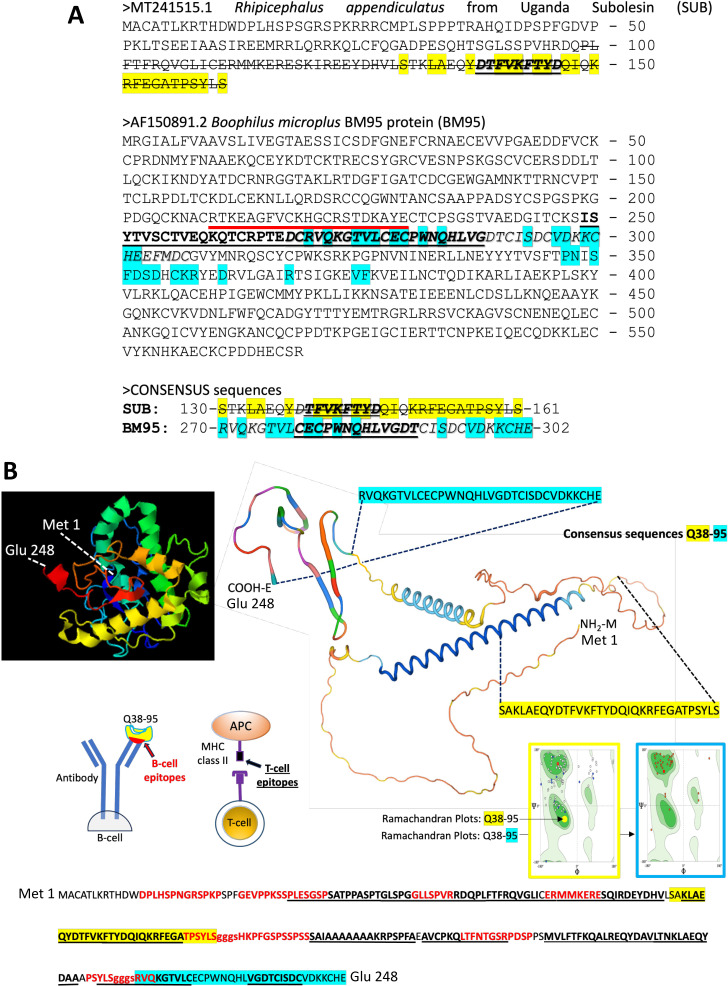
Summary of the results of SUB-BM95 interaction models. (A) Yellow and light blue: *in silico* model 1 based on the interacting residues as defined by the guided docking procedure (S2 Table). Bold underlined: *in silico* model 2 based on the main observations for interpreting the molecular docking results in both C25 and C65 (S3A and S3B Figs). Red overlined: *in silico* model 3 with BM95-subolesin complex with most probable interacting region (Fog. 4). Italics with outer shadow: *in music* model. Consensus sequences were established based on the coincidence by 2 or 3 of the models. Strikethrough: SUB-SUB interacting domains and SUB protective epitopes present in Q38/Q41 chimeric antigens. (B) Modeling Q38-95 protein and predicted B-cell and T-cell epitopes. Protein structure was modelled using I-TASSER-MTD (https://zhanggroup.org/I-TASSER-MTD/) and Swiss-Model (https://swissmodel.expasy.org). Consensus sequences are highlighted in yellow for Q38 and blue for BM95. Predicted continuous B-cell epitopes (shown in red amino acids in Q38-95 sequence) were obtained with Bcepred Prediction Server of continuous B-cell epitopes in antigenic sequences using physico-chemical properties with Flexi parameters = 2.010 - 3.363 (https://webs.iiitd.edu.in/raghava/bcepred/bcepred_submission.html). Predicted T-cell MHC I and MHC II epitopes (shown in black bold underlined amino acids in Q38-95 sequence) were obtained with MHCPred v.2. (http://www.ddg-pharmfac.net/mhcpred/MHCPred/). Only those with max confidence of prediction = 1 are shown. All results are disclosed in S4 Data and S7 Fig). The position of GGGS linkers is shown in lowercase letters.

Results of the analysis of SUB-BM95 interactions are summarized in Fig 7A based on the interacting residues as defined by the guided docking procedure used by *in silico* model 1 (S2 Table), *in silico* model 2 (S3A and S3B Figs), and *in music* model (Figs 6 and S6). Furthermore, sequences present in the SUB-SUB interacting domains and in SUB protective epitopes compiled in Q38/Q41 chimeric antigens are shown [18,22]. Despite limitations in some models such as *in silico* model 2 in which the homologous conservation of BM95 is low and docked conformations deviates quite big and *in music* model with limited scientific support, the integration of the different models is a key step to model SUB-BM95 interactions. These results supported the design of consensus sequences to validate *in vitro* SUB-BM95 protein-protein interactions and design the chimeric Q38-95 antigen (Fig 7A).

**Fig 8:**
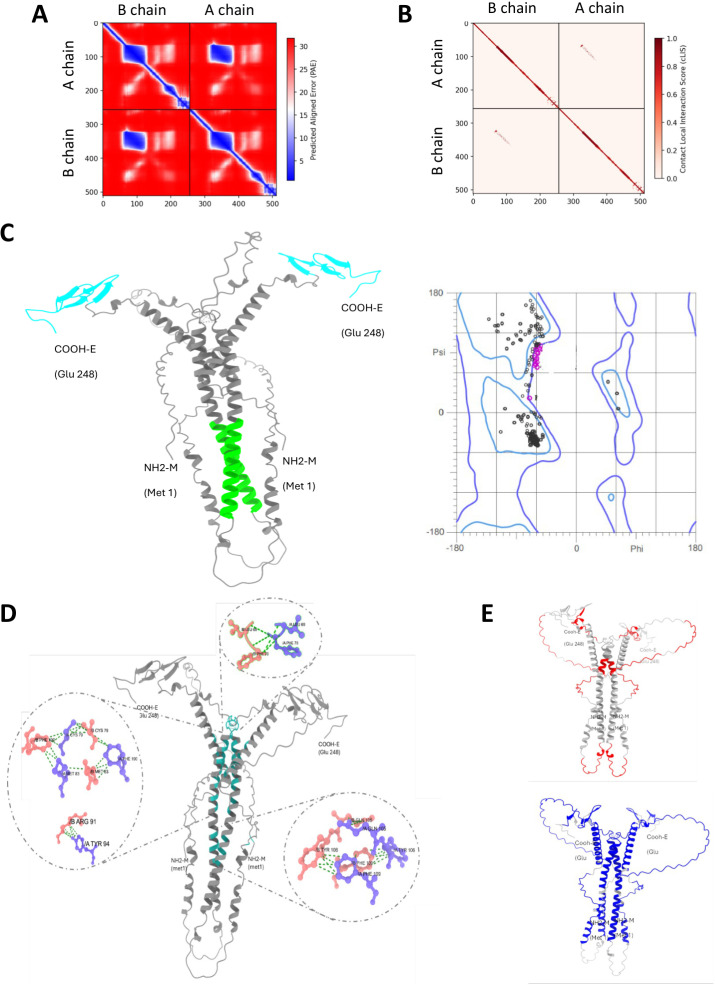
Analysis of Q38-95 self-interaction. (A) AlphaFold predictions of binding evaluation by prediction alignment error (PAE) score, which apprears on the right part of the figure showing the best-predicted residue pairs on blue and the less confidable prediction on red. The error is measured in Amstrongs. (B) Control local interaction (cLIS) score, showing the nearest residues and the ones with more probability of being interacting. (C) Predicted structure with the interacting domains for BM95 in blue and Q38 in green. Ramachandran plot for all the aminoacids is shown on the right of the structure. (D) Structure analysis of the chimeric antigen Q38-95 dimer interacting zones. Interacting zones are zoomed to show the amino acid pairs in contact. (E) Epitope distribution for T-cells (Blue) and B-cells (red) on the Q38-95 dimers predicted structure. High resolution images for panels D and E are shown in S7 Fig.

Based on these results and applying a quantum vaccinology approach, combined SUB-derived Q38 protective chimeric antigen with proposed BM95 consensus protective epitopes interacting with SUB and likely involved in protective immune response was used to design and produce the recombinant chimeric antigen Q38-95. Multiple sequence alignment of Q38 conserved epitopes KLAEQYD and FVKFTYDQI showed an 85.7% identity for tick spp., mosquitoes *Aedes* spp. and pathogen *Vibrio* spp. (S3 Data). For Q38 SAKLAEQYDTFVKFTYDQIQKRFEGATPSYLS in tick spp., multiple sequence alignment resulted in 71.9% identity (S3 Data). For BM consensus sequence RVQKGTVLCECPWNQHLVGDTCISDCVDKKCHE, a 100% identity was identified in *Rhipicephalus* spp. (S3 Data). These results support cross-reactive epitopes in Q38-95 for multiple tick species and other arthropod vectors (e.g., mosquitoes) and pathogens (e.g., *Vibrio* spp.), thus suggesting the possibility of eliciting a protective response after vaccination against different ectoparasites and pathogens. Modelling algorithms were used for prediction of T-cells and B-cells epitopes (Fig 7B and S4 Data) and solvent accessibility (S7 Fig). The results showed helix (Q38 consensus sequence) and strand-coil (BM consensus sequence) structure with same predicted solvent accessibility (3 ± 2 average ± S.D., highly exposed residues = 8) and a high representation of T-cells and B-cells epitopes, covering the predicted protective epitopes in Q38-95 consensus sequences (Figs 7B and S7). The analysis of Q38-95 self-interaction showed homodimers with a-helix sequences that prevent formation of homocomplexes between Q38 and BM95 regions and therefore do not affect recognition of B and T cell epitopes and immune response to predicted protective epitopes in Q38-95 (Figs 8A–8E and S8). Taken together, these results predict a high immunogenicity and accessibility with low interactions for consensus epitopes in the Q38-95 chimeric antigen.

### Validation of SUB-BM95 interactions

**Fig 9:**
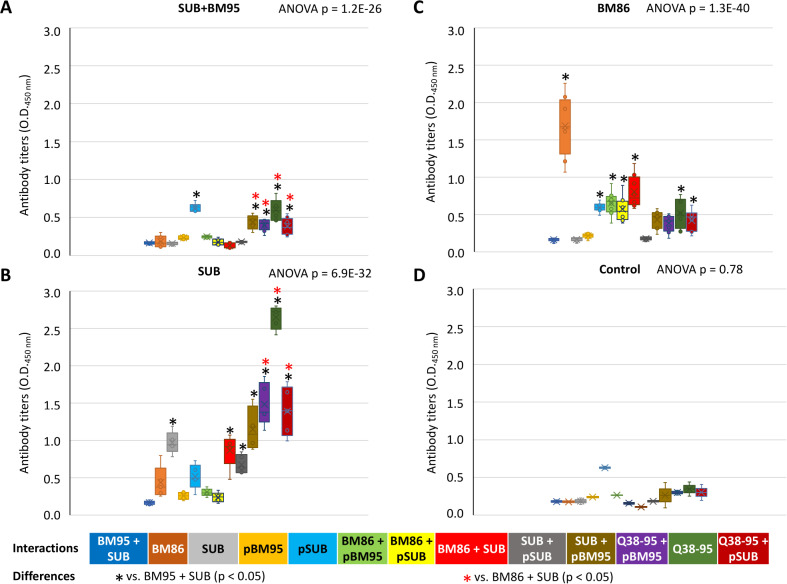
Results of ELISA tests for the validation of SUB-BM95 interactions. Sera from cattle immunized with (A) SUB+BM95, (B) SUB, (C) BM86 and (D) controls were evaluated against various peptide/protein interactions. Antibody titers (OD_450nm_) were compared between the different interactions using a One-way ANOVA test with post-Tukey Honestly Significant Difference (HSD) (black (*): p < 0.05 when compared to BM95+SUB, red (*): p < 0.05 when compared to BM86+SUB).

**Fig 10:**
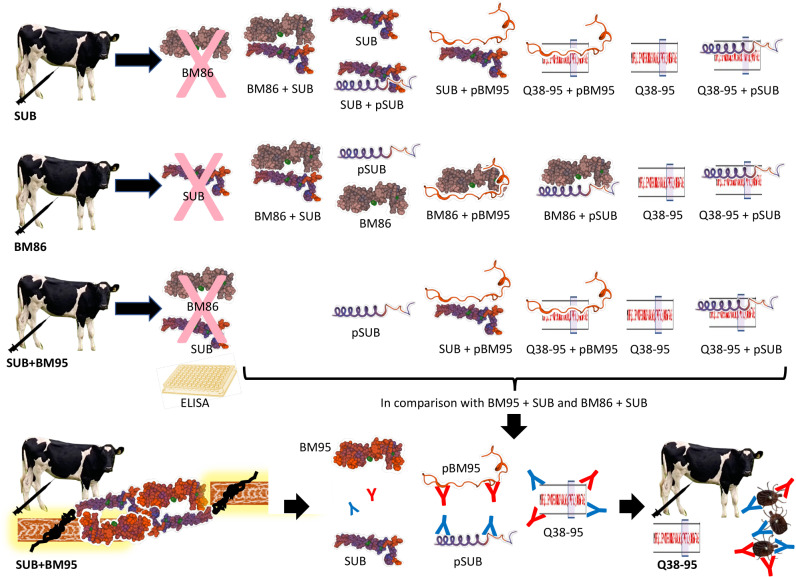
Summary of the impact of protein/peptide interactions on vaccine efficacy. The SUB-BM protein interactions reduced the antibody response to SUB and BM86 protective epitopes. Nevertheless, cattle antibody response to SUB, BM86 and SUB+BM95 vaccination recognized candidate pSUB and pBM95 protective peptides and the combined recombinant antigen Q38-95, supporting that vaccination with Q38-95 must boost protective immune response against tick infestations by targeting protective epitopes involved in protein-protein interactions.

For validation of modelled SUB-BM95 interactions, sera from cattle immunized with SUB+BM95, BM86 or SUB were used to evaluate reactions with peptide/protein combinations by ELISA (Figs. 9A-9D). The results showed relatively low anti-BM86 and anti-SUB IgG antibody titers in cattle immunized with SUB+BM95 (0.18 ± 0.08 and 0.16 ± 0.02 for anti-BM86 and anti-SUB IgG, respectively) (Fig 9A) and controls (0.18 ± 0.01 and 0.19 ± 0.06 for anti-BM86 and anti-SUB IgG, respectively) (Fig 9D) when compared to antibody response to vaccination with BM86 (1.69 ± 0.40 and 0.17 ± 0.03 for anti-BM86 and anti-SUB IgG, respectively) (Fig 9C) and SUB (0.44 ± 0.22 and 0.97 ± 0.15 for anti-BM86 and anti-SUB IgG, respectively) (Fig 9C). As expected, cattle vaccinated with BM86 or SUB produced higher antibody levels to vaccine antigens when compared to non-vaccine antigens (Figs 9B, 9C and 10).

Nevertheless, cattle vaccinated with BM86 produced significantly higher anti-SUB antibody levels when compared to anti-BM95+SUB titers (Fig 9C). These results suggested some cross-reactivity between BM95 and SUB proteins. In agreement with this possibility, SUB-BM95 protein sequence alignment identified common epitopes including the consensus interacting sequence region KRFE (SUB amino acids 150–153), also involved in SUB-SUB interactions (Fig 7A and S5 Data).

The antibodies against SUB and BM86 protective epitopes were underrepresented in response to immunization with combined SUB+BM95 antigen (Figs 9A and 10), likely due to SUB-BM95 protein-protein interactions. Interaction of pSUB with Q38-95 did not affect antibody response in cattle immunized with SUB, BM86 and SUB-BM95, while interactions of pBM95 with Q38-95 and SUB only interfered with antibody response to BM86 (Fig 9A). However, antibodies in cattle immunized with SUB, BM86 and SUB+BM95 did recognize candidate pSUB and pBM95 protective peptides and the combined recombinant antigen Q38-95 (Figs 9A–9C and 10). The average antibody response to Q38-95 was higher than 0.5 O.D._450 nm_ (2.7 average O.D._450_ nm in response to SUB) in all vaccinated groups (Fig 9A–9D). Therefore, vaccination with Q38-95 must boost protective immune response against tick infestations by targeting protective epitopes involved in protein-protein interactions (Fig 10).

**Fig 11:**
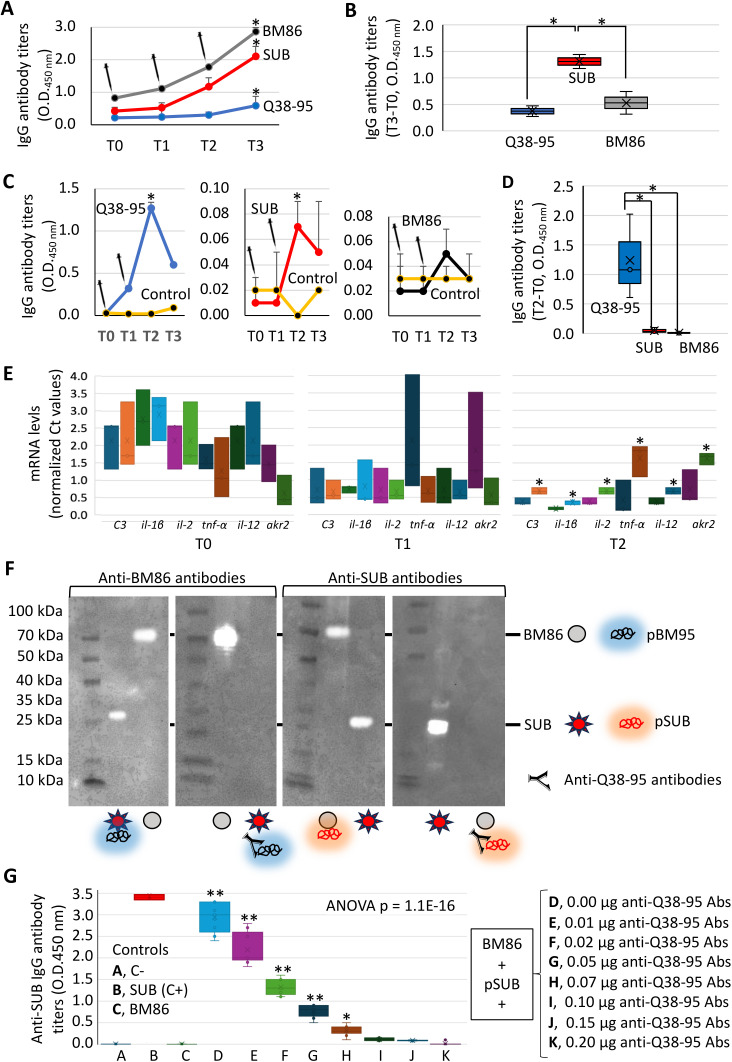
Rabbit and cattle immune response to Q38-95. The IgG antibody titers against vaccine antigens Q38-95, SUB and BM86 were determined by ELISA in rabbits and cattle vaccinated with Q38-95. (A) Rabbit antibody titers represented as the average + S.D. of the O.D._450 nm_ and the mean of the duplicate values were compared between T3 and T0 by Student’s t-test with unequal variance (*p < 0.05; n = 2 for each group). (B) Rabbit antibody titers (T3-T0 O.D._450 nm_) in vaccinated animals were compared between groups by one-way ANOVA with post-hoc Tukey HSD (*p < 0.05; n = 2 for each group). (C) Cattle antibody titers represented as the average + S.D. of the O.D._450 nm_ and the mean of the quadruplicate values were compared between T2 and T0 by Student’s t-test with unequal variance for vaccinated and control animals (*p < 0.05; n = 4 for each group). (D) Cattle antibody titers (T2-T0 O.D._450 nm_) in vaccinated animals were compared between groups by one-way ANOVA with post-hoc Tukey HSD (*p < 0.05; n = 4 for each group). (E) Normalized mRNA cycle threshold (Ct) values for selected immune biomarkers in control and Q38-95 vaccinated cattle at T0, T1 and T2. Values were compared between control and vaccinated groups at T0, T1 and T2 by one-way ANOVA with post-hoc Tukey HSD (*p < 0.05; n = 3 for each group). (F) Anti-Q38-95 antibody-mediated inhibition of SUB-BM95 protein-protein interactions was analyzed by Western blot using sera from rabbits immunized with Q38-95 and controls. The SUB-pBM95 and BM86-pSUB interactions were inhibited by anti-Q38-95 antibodies. (G) Anti-Q38-95 antibody-mediated inhibition of SUB-BM95 protein-protein interactions was quantified by ELISA using sera from cattle immunized with Q38-95 and controls (no protein C-, SUB C+ and BM86). Results (O.D._450 nm_) of interactions between BM86 and pSUB with different amounts on cattle anti-Q38-95 antibodies were compared between groups by one-way ANOVA with post-hoc Tukey HSD (**p < 0.01 for treatments when compared to all with higher amount of anti-Q38-95 Abs and *p < 0.05 for treatment H when compared to treatment K; n = 9 for each treatment).

Rabbits were then immunized with recombinant Q38-95, and sera used to evaluate IgG antibody titers against Q38-95, SUB and BM86 (Figs 11A and 11B). The results showed significantly higher antibody titers 49 days after the first immunization (T3) when compared to pre-vaccination samples (T0) (p < 0.05; Fig 11A). In agreement with results with cattle anti-SUB and anti-BM86 antibodies against Q38-95 (Figs 9B and 9C), the cross-reactivity of anti-Q38-95 antibodies was significantly higher against SUB (p < 0.05; Fig 11B).

### Vaccine efficacy and immune response in cattle treated with Q38-95

The results of the preliminary analysis of Q38-95 E in cattle resulted in reduction of 12% in *Rhipicephalus decoloratus* tick infestations (DT, 22 vs. 25 engorged female ticks), 13% in tick weight (DW, 140 ± 60 mg vs. 170 ± 40 mg), 18% in oviposition (DO, 240 ± 220 mg vs. 300 ± 180 mg), and 46% in egg fertility (DF, 1.1 ± 0.9 mg vs. 4.4 ± 1.3) in vaccinated vs. control cattle. These results translated into a E of 82% calculated as E =100 x [1-((22/25) x (240/300) x (1.1/4.4))]. Antibody response increased in vaccinated cattle with significant differences at T2 for Q38-95 and SUB when compared to pre-vaccination samples (T0) (p < 0.05; Fig 11C). The antibody response to Q38-95 was different between rabbits (Fig 11B) and cattle (Fig 11D) with the cross-reactivity of anti-Q38-95 antibodies significantly higher against chimeric antigen in cattle (p < 0.05; Fig 11D). The mRNA levels for selected immune biomarkers showed significant differences between control and vaccinated cattle only at T2 (day 45 before tick infestations) with higher levels in response to Q38-95 (p < 0.05; Fig 11E).

### Antibody-mediated inhibition of SUB-BM95 protein-protein interactions

Finally, experiments were conducted to evaluate the capacity of anti-Q38-95 antibodies to inhibit SUB-BM95 interactions using sera from immunized rabbits (Fig 11F) and cattle (Fig 11G). The results supported the interaction of anti-Q38-95 antibodies with pBM95 and pSUB resulting in the inhibition of SUB-BM95 protein-protein interactions with significant correlation with antibody protein amount (Fig 11G).

## Discussion

The possibility of combining protective antigens may improve E but only if protein-protein interactions do not affect host immune response, a challenge that could be approached with quantum vaccinology algorithms to combine protective epitopes in a chimeric antigen [9]. As shown here, the combination and integration of different algorithms to model protein interactions resulted in the identification of interacting epitopes that may be associated with E.

Recent applications such as protein molecular modelling, prediction of B-cell epitopes, assembling multiple sequences and reverse vaccinology have advanced in the design of multiepitope vaccines (e.g., [20,22,23,73–78]) and as shown here the combination and integration of different algorithms may improve antigen design and predictive protective capacity. In this study, the interaction between tick vaccine antigens with proven efficacy, BM and SUB reduced E, and protein-protein interactions were inhibited in response to antibodies against the designed chimeric antigen, Q38-95, with combined modeled interacting and protective epitopes. The identified SUB-derived region was present in the previously designed Q38 chimeric antigen [21] validated by *in music* algorithm [22] and peptide array analysis [18] with E against various ectoparasite infestations [21,26] and pathogen infection [79]. These results support the approach used here to model protein-protein interactions affecting E of antigen combination to identify candidate protective epitopes. Furthermore, the size of immunodominant epitopes but particularly positively altering immunodominance and the size of competing clonal B-cell pools should be considered [80].

In previous studies, *R. decoloratus* SUB E was reported as 69% with effect only on tick fertility [68]. Regarding BM86, E of 87% was reported in *R. decoloratus* [81]. In our preliminary study, E with Q38-95 (82%) was similar to BM86 and higher than SUB and the main advantage of this antigen is the possibility of providing protection against multiple tick species and genetic variants.

The antibody response in cattle vaccinated with Q38-95 showed differences with immunized rabbits, which as in previous studies may be associated with species-specific protein reactive epitopes recognized by IgG antibodies [24]. Nevertheless, both rabbit and cattle anti-Q38-95 antibodies inhibited protein-protein interactions associated with E in vaccinated cattle. Therefore, the antibody response against Q38-95 may be different between animal species but with protective capacity against tick infestations. Additionally, differences in protective immunity between host species may be also used to identify additional protective epitopes [24].

The immune mechanisms activated in cattle in response to vaccination with Q38-95 were not only mediated by antibodies but also through activation of other innate and adaptive immune pathways. Antibody-mediated protective immune response to vaccination with BM and SUB antigens affects tick life cycle after feeding on immunized hosts by still unknown mechanisms that may be associated with antibody-interference with their regulatory function affecting multiple biological processes [82]. Additionally, oral immunization with SUB combined with heat-inactivated mycobacteria as immunostimulant has shown activation of C3 pathway [83,84]. In our study, *C3*, *tnf-α*, *il-1β*, *il-2*, *il-12*, and *akr2* mRNA levels increased in response to Q38-95 in vaccinated cattle. Some of these biomarkers such as C3 are associated with bridging of innate and adaptive immune responses [85,86], a finding that has been previously associated with correlation between anti-SUB antibody titers, *C3*, *il-1β* and *akr2* mRNA levels and reduction in tick infestations [83]. Taken together, these results suggested the possibility that the combination of B and T-cell protective epitopes in Q38-95 could activate various immune mechanisms with potential effects on reducing tick infestations and pathogen infection/transmission.

## Conclusion

In conclusion, the results of this study showed production of anti-BM and anti-SUB reactive antibodies and upregulation of other immune biomarkers in response to immunization with the designed Q38-95 chimeric antigen. Future experiments should evaluate protective mechanisms and Q38-95 E against multiple tick species of different geographical origin and against other arthropod vectors and pathogen infection/transmission in multiple hosts. These results are relevant not only for vaccinology but also for design of biomolecules with pharmaceutical applications.

## Supporting information

S1 Supporting Information Supplementary Data on *in silico*, *in music*, Q38-95 and SUB-BM95 interaction models. (PDF)

S1 Supplementary audio files Audio files of *in music* models for SUB, BM95 and SUB-BM95 interactions. (7z)

S1 raw images Original images of Western blot analysis of anti-Q38-95 antibody-mediated inhibition of SUB-BM95 protein-protein interactions (Fig 11F). (PDF)
