# Supplementary material for: Modelling protein-protein interactions for the design of vaccine chimeric antigens with protective epitopes
Source: PLoS One. 2025 Feb 10;20(2):e0318439. doi: 10.1371/journal.pone.0318439 (PMC11809815; doi:10.1371/journal.pone.0318439)
Supplement: S1 Supporting Information — (PDF) [file pone.0318439.s001.pdf]

Supporting information for

Modelling protein-protein interactions for the design of vaccine chimeric antigens with protective epitopes

Marinela Contreras[1], Marta Rafael[1], Isidro Sobrino[1], Consuelo Almazán[2], Juan J. Pastor Comín[3], James J. Valdés[4], Carlos Roberto Prudencio[5,6], Daniel Ferreira de Lima Neto[7], Veniamin A. Borin[8], Pratul K. Agarwal[8,9], Paul D. Kasaija[10], Rubén Fernández-Melgar[1], Justus Rutaisire[10], José de la Fuente[1,11*]

[1]SaBio, Instituto de Investigación en Recursos Cinegéticos (IREC), Consejo Superior de Investigaciones Científicas (CSIC), Universidad de Castilla-La Mancha (UCLM)-Junta de Comunidades de Castilla-La Mancha (JCCM), Ronda de Toledo 12, 13005 Ciudad Real, Spain.
[2]Laboratorio de Inmunología y Vacunas, Facultad de Ciencias Naturales, Universidad Autónoma de Querétaro. 76140, Querétaro, Mexico.
[3]Centro de Investigación y Documentación Musical CIDoM-UCLM-CSIC, Facultad de Educación de Ciudad Real, Ronda Calatrava 3, 13071 Ciudad Real, Spain.
[4]Institute of Parasitology, Biology Centre, Czech Academy of Sciences, Branišovská 1160/31, 37005 České Budějovice, Czech Republic.
[5]Immunology Center, Adolfo Lutz Institute, São Paulo, SP, Brazil.
[6]Graduate Program Interunits in Biotechnology, University of São Paulo, São Paulo, Brazil.
[7]General Coordination of Public Health Laboratories, Health Surveillance Secretariat, Ministry of Health, Brasília, Federal District, Brazil.
[8]Department of Physiological Sciences, Oklahoma State University, Stillwater, Stillwater, OK 74078, USA.
[9]High-Performance Computing Center, Oklahoma State University, Stillwater, Stillwater, OK 74078, USA.
[10]National Livestock Resources Research Institute (NaLIRRI/NARO), Kampala P.O. Box 5704, Uganda.
[11]Center for Veterinary Health Sciences, Department of Veterinary Pathobiology, Oklahoma State University, Stillwater, OK 74078, USA.

*Correspondence: José de la Fuente, SaBio, Instituto de Investigación en Recursos Cinegéticos (IREC-CSIC-UCLM-JCCM), Ronda de Toledo 12, 13005 Ciudad Real, Spain. Email: jose_delafuente@yahoo.com / josedejesus.fuente@uclm.es

Subolesin

BM95

Model

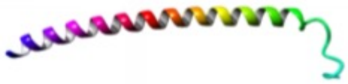

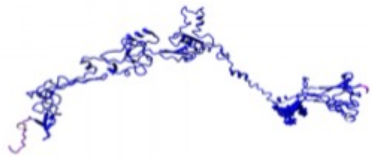

Overall structure quality

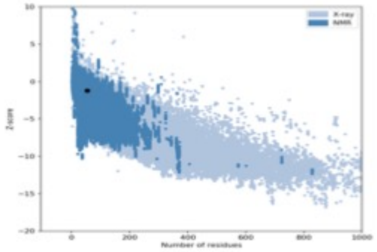

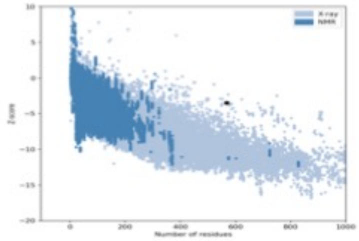

Ramachandran plot

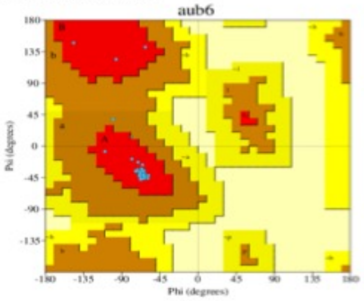

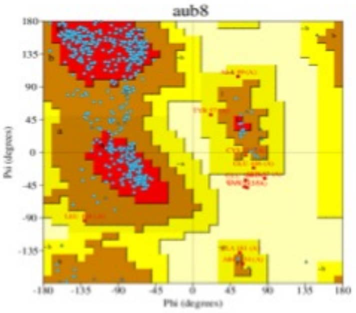

Pockets

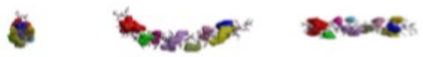

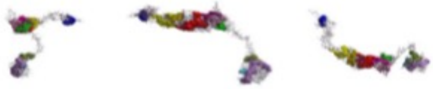

Clefts

Cleft	Volume	R1 ratio	Accessible vertices		Buried vertices		Average depth		Residue type						
1	714.23	1.84	47.39	10	7.91	1	9.83	1	0	1	3	3	3	2	0
2	387.28	0.00	50.80	7	6.10	4	6.30	5	0	0	3	2	3	0	0
3	315.98	0.00	51.25	6	4.75	5	5.84	7	2	1	1	1	1	0	0
4	210.94	0.00	52.43	4	2.95	8	5.21	10	3	2	1	0	0	0	0
5	194.91	0.00	51.82	5	3.24	7	5.82	8	1	0	2	1	1	0	0
6	228.66	0.00	59.56	1	6.66	2	7.52	3	2	2	0	2	0	0	0
7	210.94	0.00	49.01	8	1.81	9	8.06	2	2	2	0	1	0	0	0
8	185.20	0.00	53.88	3	4.49	6	6.25	6	1	0	1	3	1	0	0
9	165.38	0.00	48.73	9	6.36	3	6.35	4	1	1	0	1	1	0	0
10	104.20	0.00	55.81	2	1.61	10	5.45	9	1	2	1	0	0	0	0

Protein structure

Clefts

Cleft	Volume	R1 ratio	Accessible vertices		Buried vertices		Average depth		Residue type						
1	6161.06	1.68	64.60	2	12.09	2	17.35	1	17	11	17	17	4	7	10
2	3660.19	0.00	60.50	6	10.80	4	15.49	3	9	8	8	8	7	9	7
3	2964.09	0.00	60.28	7	10.89	3	17.00	2	8	8	10	9	1	6	5
4	1567.27	0.00	55.76	10	9.06	7	12.81	4	2	6	3	9	2	2	0
5	2013.19	0.00	69.75	1	12.88	1	11.07	8	4	5	8	12	2	4	4
6	1899.70	0.00	63.69	3	10.57	6	11.36	7	5	3	8	4	2	8	4
7	1624.64	0.00	61.79	4	7.38	10	12.38	5	6	3	6	9	4	2	2
8	1575.70	0.00	55.93	9	7.54	9	11.46	6	4	6	6	5	3	1	1
9	1090.12	0.00	61.68	5	10.72	5	7.44	10	1	1	6	2	6	3	1
10	608.34	0.00	56.97	8	8.15	8	7.48	9	4	1	1	3	0	1	2

Protein structure

Overall structure quality

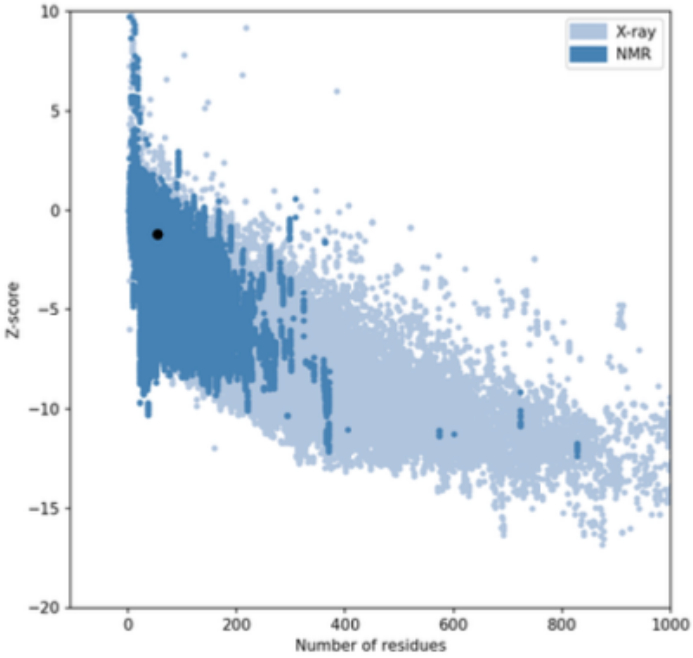

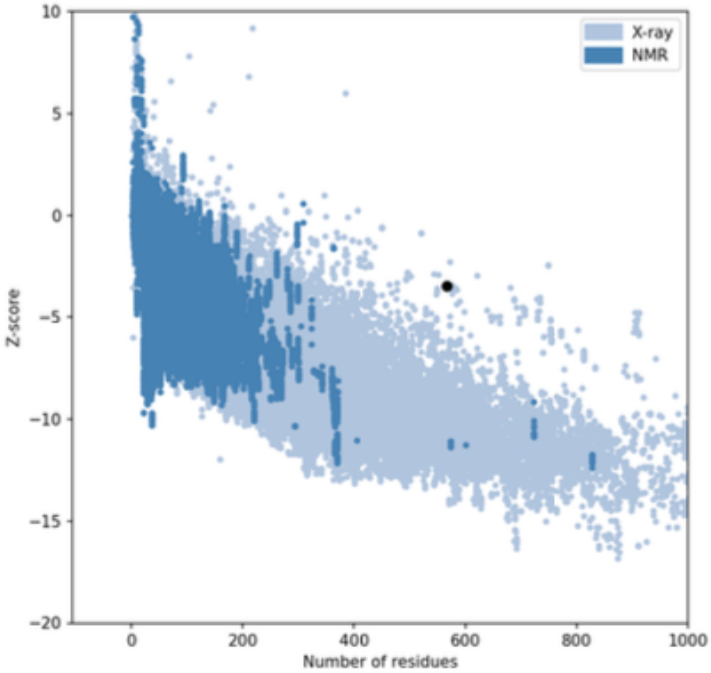

Pockets

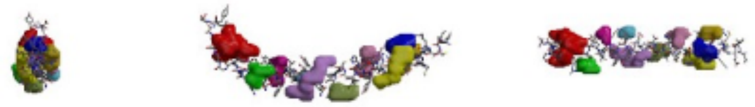

Clefts

Clefts	Volume	R1 ratio	Accessible vertices		Buried vertices		Average depth		Residue type						
1	714.23	1.84	47.39	10	7.91	1	9.83	1	0	1	3	3	3	2	0
2	387.28	0.00	50.80	7	6.10	4	6.30	5	0	0	3	2	3	0	0
3	315.98	0.00	51.25	6	4.75	5	5.84	7	2	1	1	1	1	0	0
4	210.94	0.00	52.43	4	2.95	8	5.21	10	3	2	1	0	0	0	0
5	194.91	0.00	51.82	5	3.24	7	5.82	8	1	0	2	1	1	0	0
6	228.66	0.00	59.56	1	6.66	2	7.52	3	2	2	0	2	0	0	0
7	210.94	0.00	49.01	8	1.81	9	8.06	2	2	2	0	1	0	0	0
8	185.20	0.00	53.88	3	4.49	6	6.25	6	1	0	1	3	1	0	0
9	165.38	0.00	48.73	9	6.36	3	6.35	4	1	1	0	1	1	0	0
10	104.20	0.00	55.81	2	1.61	10	5.45	9	1	2	1	0	0	0	0

Protein structure

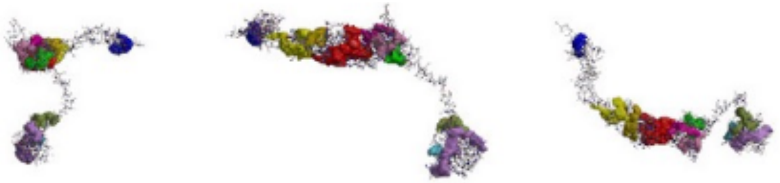

Clefts

Clefts	Volume	R1 ratio	Accessible vertices		Buried vertices		Average depth		Residue type						
1	6161.06	1.68	64.60	2	12.09	2	17.35	1	17	11	17	17	4	7	10
2	3660.19	0.00	60.50	6	10.80	4	15.49	3	9	8	8	8	7	9	7
3	2964.09	0.00	60.28	7	10.89	3	17.00	2	8	8	10	9	1	6	5
4	1567.27	0.00	55.76	10	9.06	7	12.81	4	2	6	3	9	2	2	0
5	2013.19	0.00	69.75	1	12.88	1	11.07	8	4	5	8	12	2	4	4
6	1899.70	0.00	63.69	3	10.57	6	11.36	7	5	3	8	4	2	8	4
7	1624.64	0.00	61.79	4	7.38	10	12.38	5	6	3	6	9	4	2	2
8	1575.70	0.00	55.93	9	7.54	9	11.46	6	4	6	6	5	3	1	1
9	1090.12	0.00	61.68	5	10.72	5	7.44	10	1	1	6	2	6	3	1
10	608.34	0.00	56.97	8	8.15	8	7.48	9	4	1	1	3	0	1	2

Protein structure

S1 Fig. *In silico* model 1. Overall structure, quality analyses, Ramachandran plot and pockets for SUB and BM95. For overall structure quality and pockets see bellow high resolution images.

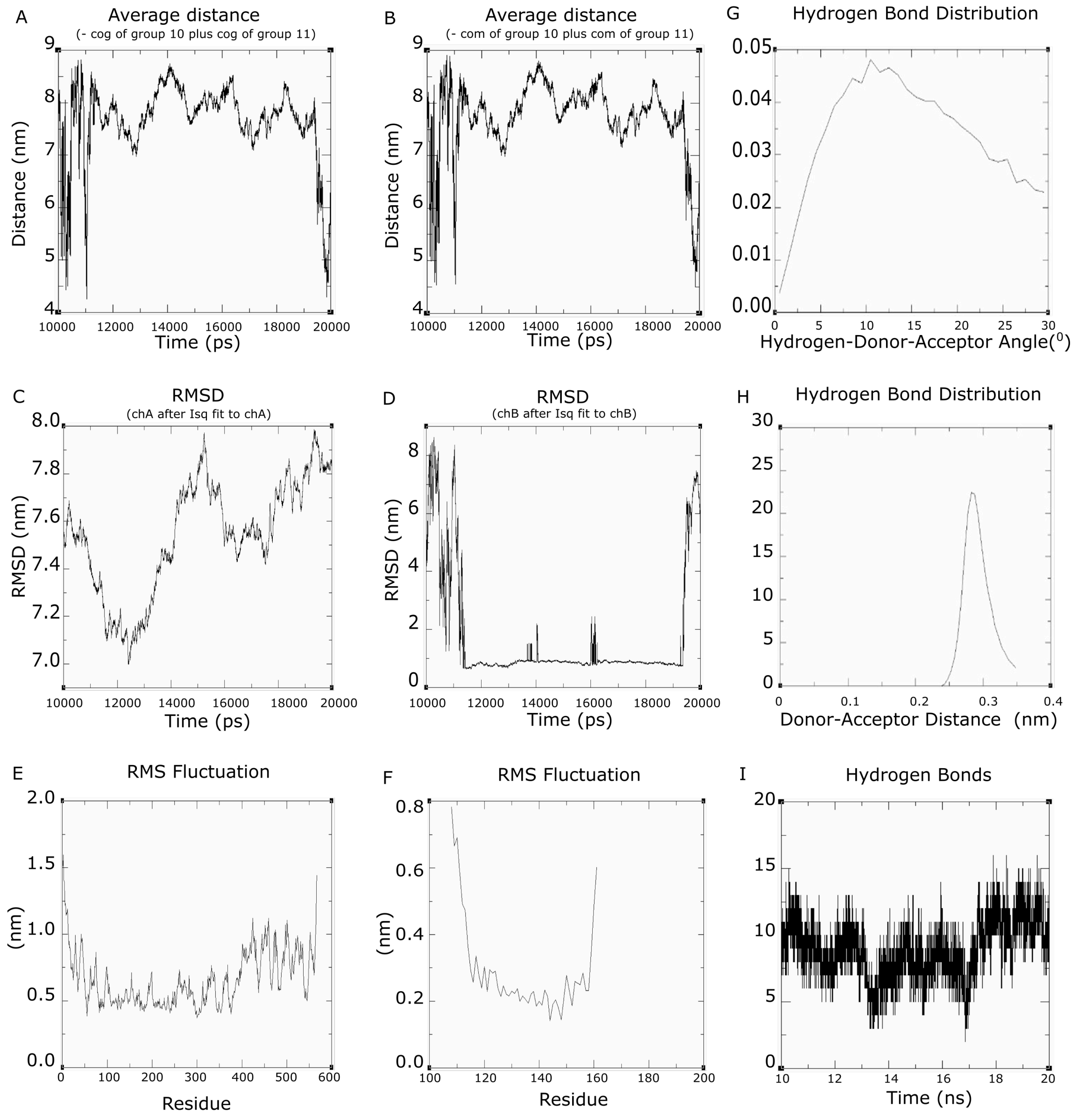

S2 Fig. Molecular dynamics of the docked pair and hydrogen bond characteristics between BM95 and SUB. (A) Distances between the center of gravity of the BM95 and SUB over the last 10 ns of the simulation. (B) Distances between the center of mass of the BM95 and SUB over the last 10 ns of the simulation. (C) Root mean squared deviation of the BM95 after the equilibration period of the first 10 ns, representation spans the last 10 ns. (D) Root mean squared deviation of the SUB after the SUB period of the first 10 ns, representation spans the last 10 ns. (E) Root mean squared fluctuation of the BM95 after the equilibration period of the first 10 ns, representation spans the last 10 ns. (F) Root mean squared fluctuation of the SUB after the equilibration period of the first 10 ns, representation spans the last 10 ns. (G) H-bond donor and acceptor angle. (H) H-bond donor and acceptor distance. (I) Number of H-bonds formed during the production run.

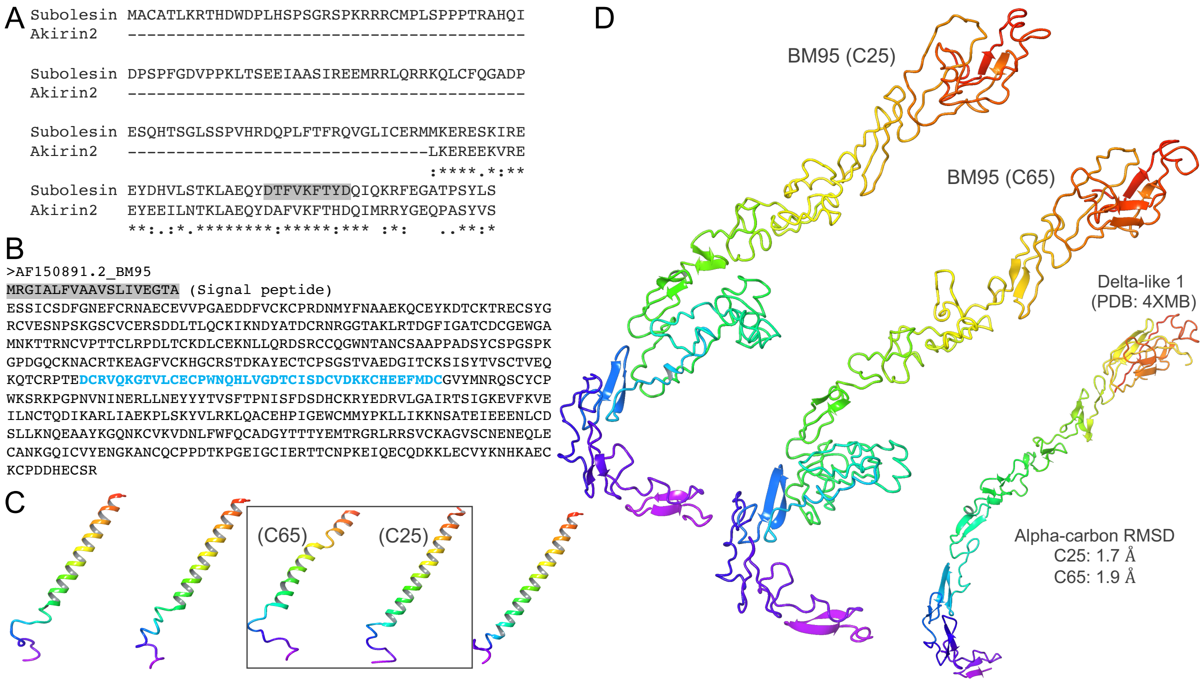

S3 Fig. *In silico* model 2. (A) Primary sequence, pairwise alignment of SUB with the resolved carboxyl-terminus of Akinin2 (PDB: 7NHT). The grey highlighted SUB region is integral for BM95 binding. (B) Primary sequences for BM95 with the integral binding residues in cyan. (C) The five predicted SUB tertiary structures color labeled from the amino-terminus (red) to the carboxyl-terminus (purple). The enclosed structures are the initial models for the two selected docked conformations. (D) The top predicted BM95 tertiary structures color labeled as for SUB in (C). The human delta-1 like cell surface protein is also shown with the two BM95, alpha-carbon backbone, root mean square deviation (RMSD) indicated.

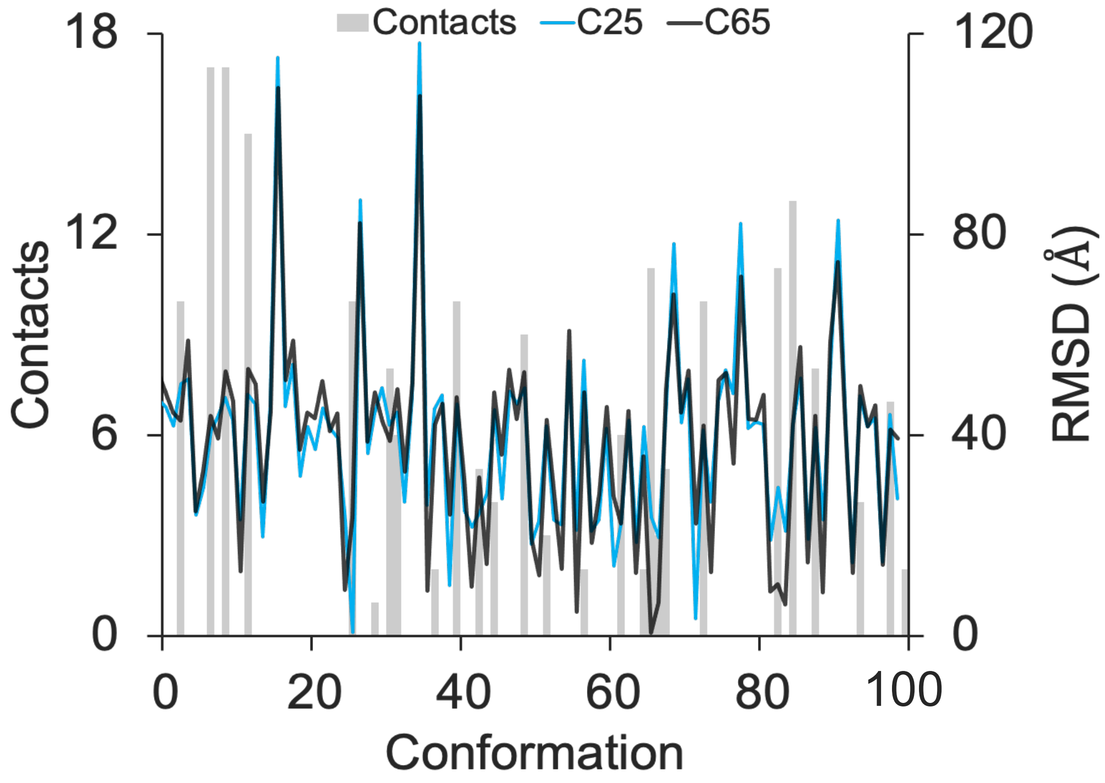

S4 Fig. Protein residue contacts for all docked conformations of BM95 with the truncated SUB. The number of residue contacts (y-axis) for all docked conformations of BM95 with the truncated SUB (n = 100; x-axis). The secondary y-axis indicates the alpha-carbon backbone root mean square deviation (RMSD) of the top two conformations (legend) compared with all docked conformations (x-axis).

SUB

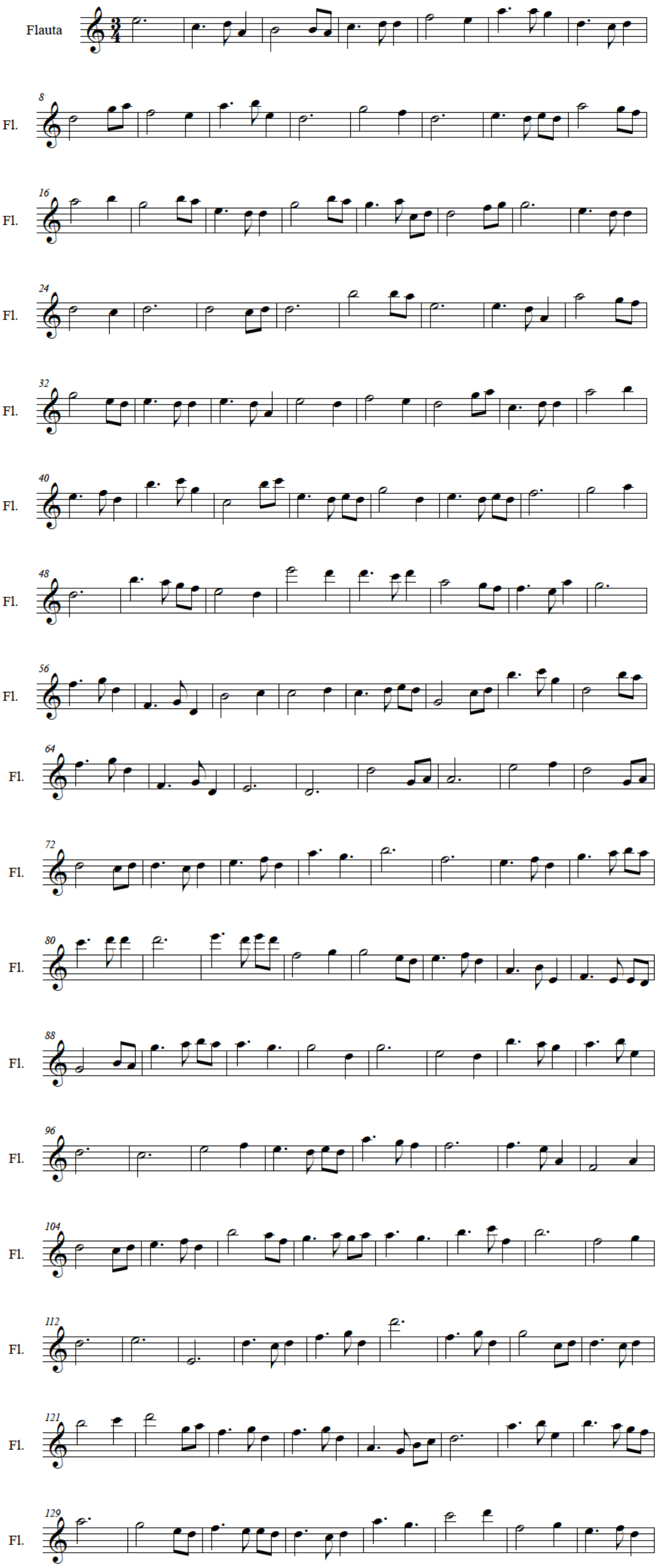

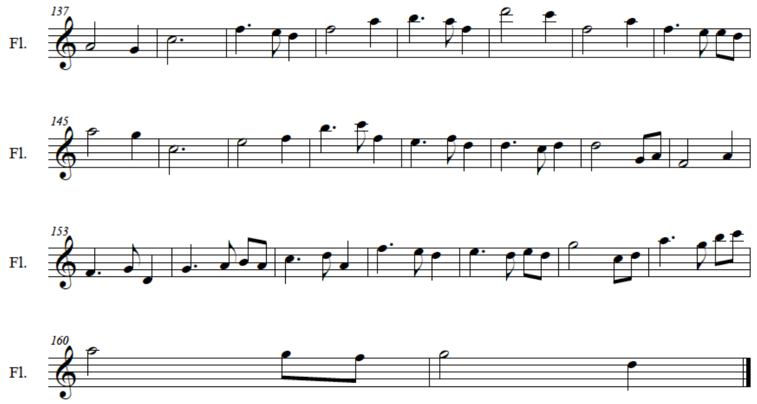
137
Fl.
145
Fl.
153
Fl.
160
Fl.

BM95

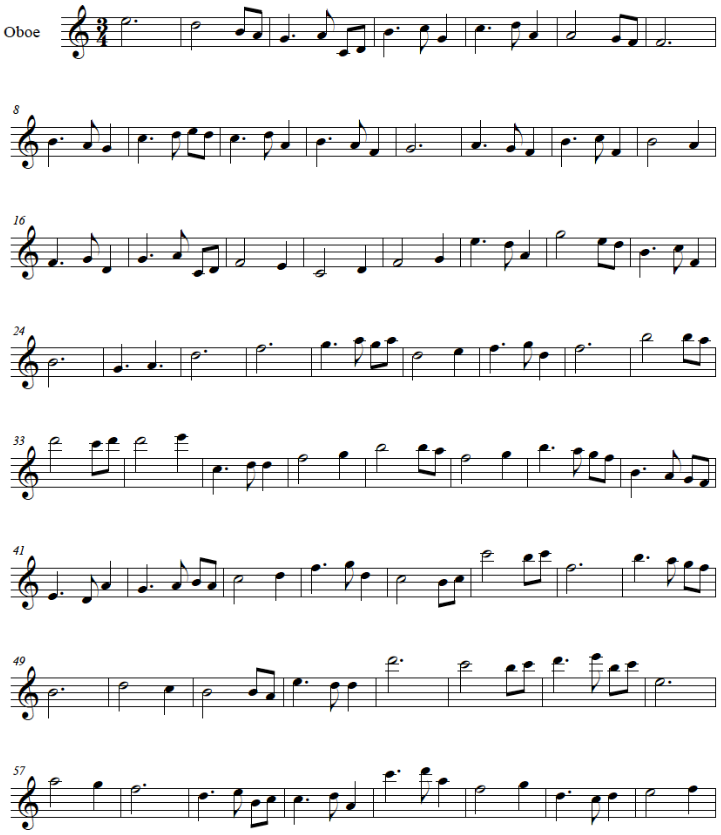
Oboe
8
Ob.
16
Ob.
24
Ob.
33
Ob.
41
Ob.
49
Ob.
57
Ob.

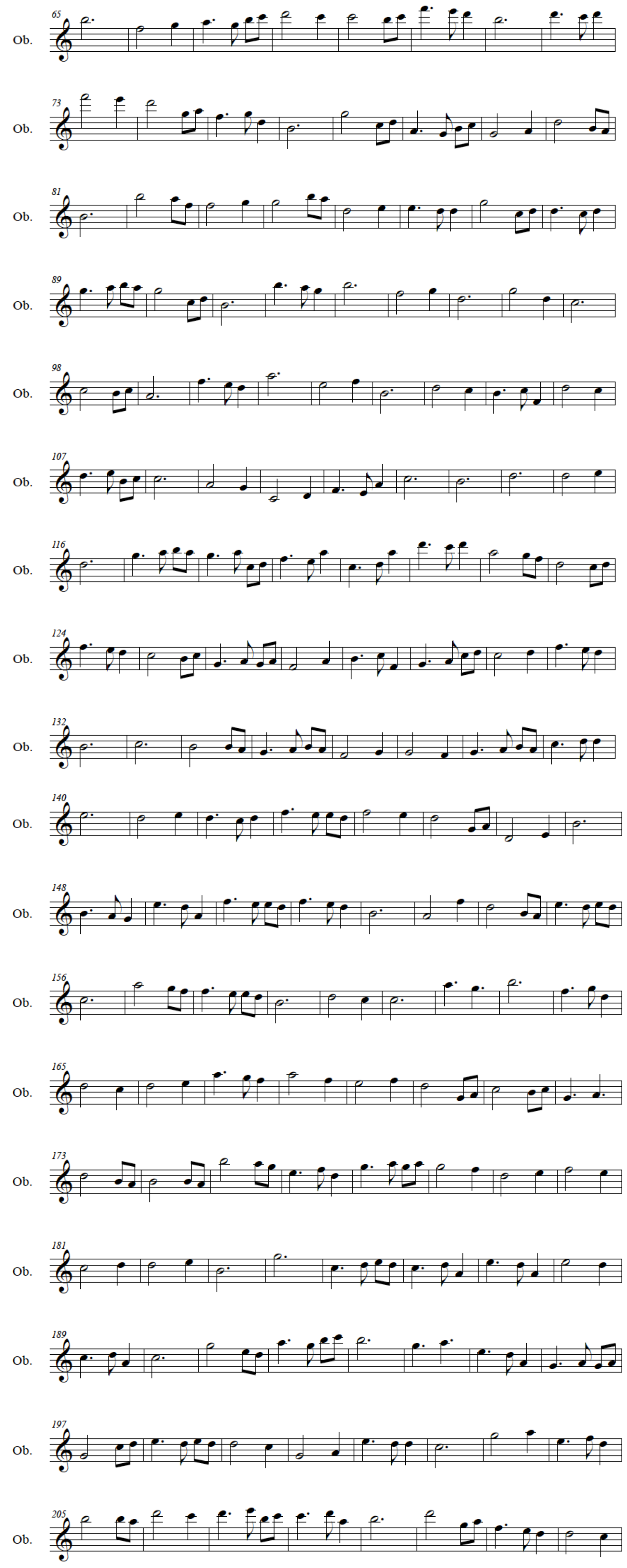
65
Ob.
73
Ob.
81
Ob.
89
Ob.
98
Ob.
107
Ob.
116
Ob.
124
Ob.
132
Ob.
140
Ob.
148
Ob.
156
Ob.
165
Ob.
173
Ob.
181
Ob.
189
Ob.
197
Ob.
205
Ob.

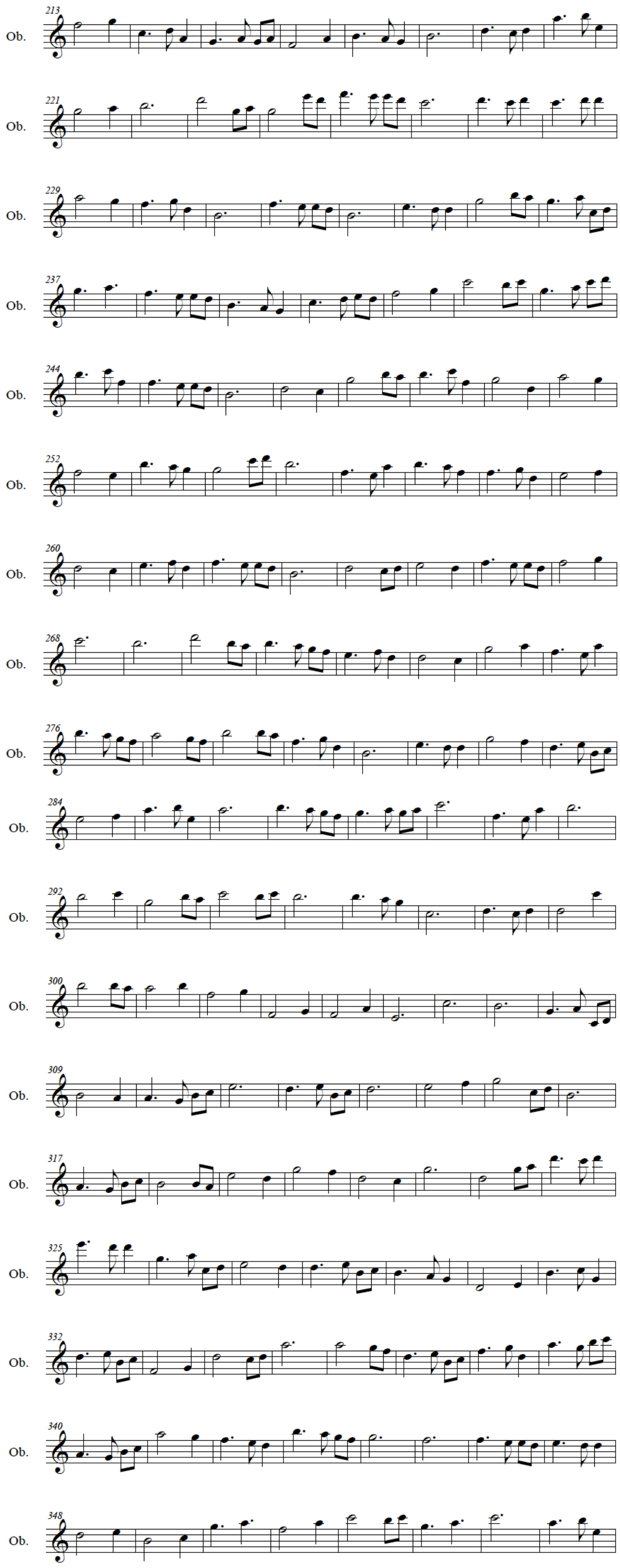
213 Ob.
221 Ob.
229 Ob.
237 Ob.
244 Ob.
252 Ob.
260 Ob.
268 Ob.
276 Ob.
284 Ob.
292 Ob.
300 Ob.
309 Ob.
317 Ob.
325 Ob.
332 Ob.
340 Ob.
348 Ob.

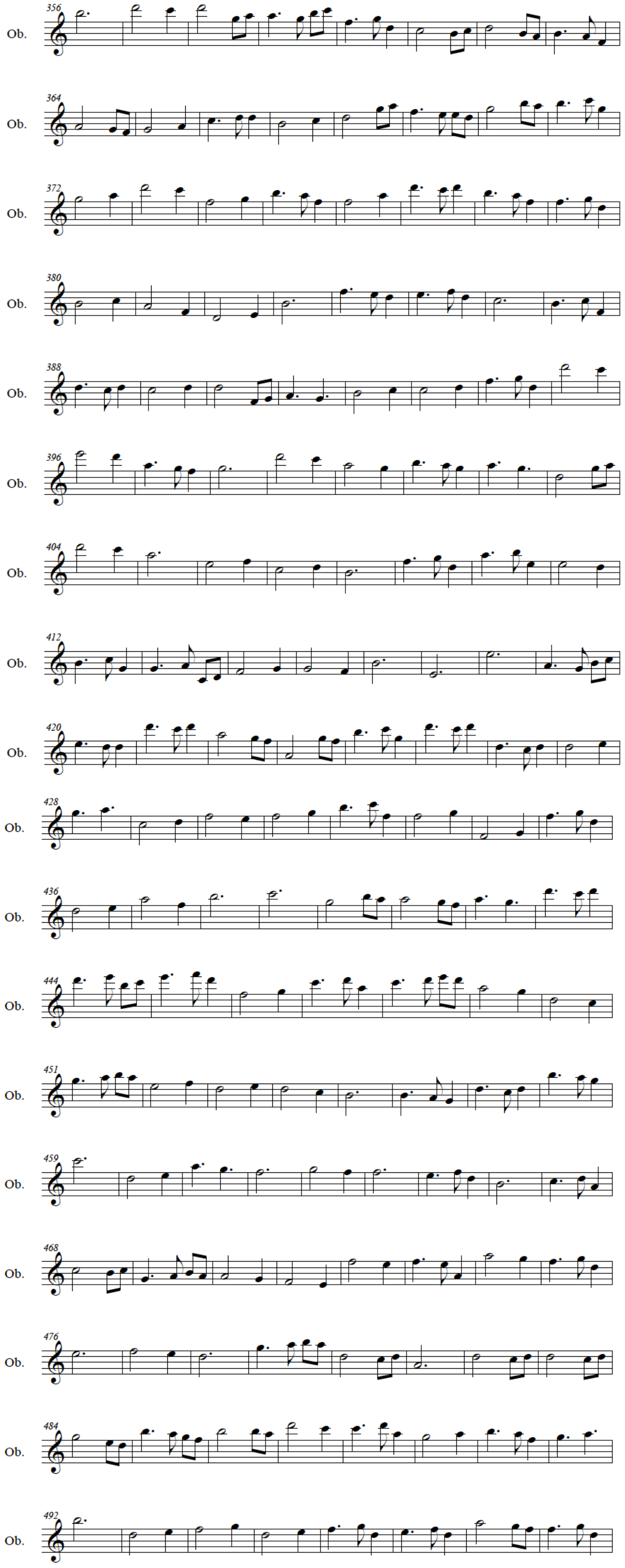
356
Ob.
364
Ob.
372
Ob.
380
Ob.
388
Ob.
396
Ob.
404
Ob.
412
Ob.
420
Ob.
428
Ob.
436
Ob.
444
Ob.
451
Ob.
459
Ob.
468
Ob.
476
Ob.
484
Ob.
492
Ob.

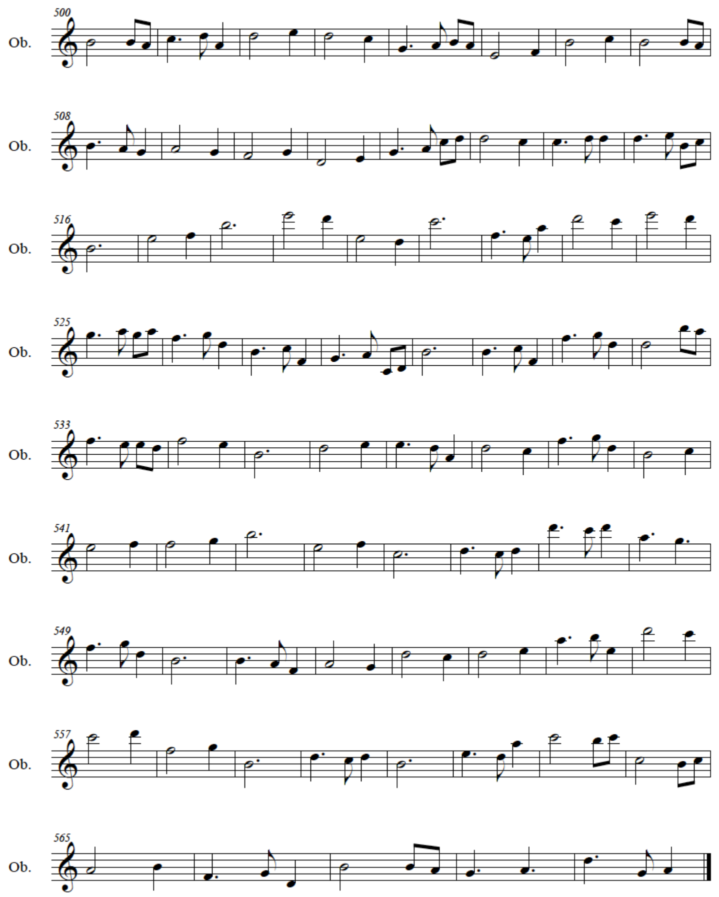

S5 Fig. Musical scores for SUB and BM95.

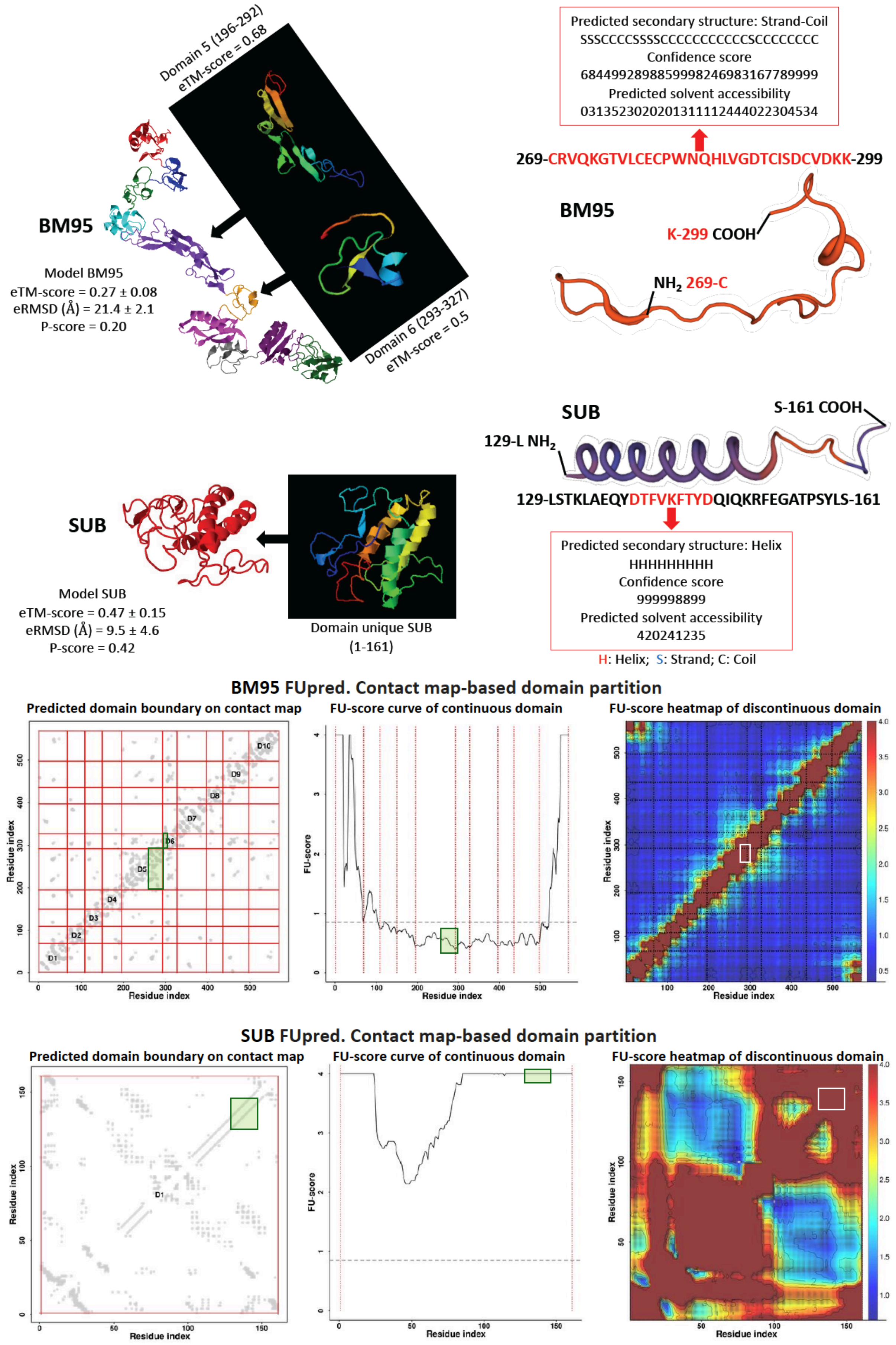
Domain 5 (196-292)
eTM-score = 0.68
BM95
Model BM95
eTM-score = 0.27 ± 0.08
eRMSD (Å) = 21.4 ± 2.1
P-score = 0.20
Domain 6 (293-327)
eTM-score = 0.5
Predicted secondary structure: Strand-Coil
SSSCCCCSSSSCCCCCCCCCCCSCCCCCCCC
Confidence score
6844992898859998246983167789999
Predicted solvent accessibility
0313523020201311112444022304534
269-CRVQKGTVLCECPWNQHLVGDTCISDCVDKK-299
BM95
K-299 COOH
NH2 269-C
SUB
S-161 COOH
129-L NH2
129-LSTKLAEQYDTFVKFTYDQIQKRFEGATPSYLS-161
SUB
Model SUB
eTM-score = 0.47 ± 0.15
eRMSD (Å) = 9.5 ± 4.6
P-score = 0.42
Domain unique SUB
(1-161)
Predicted secondary structure: Helix
HHHHHHHHH
Confidence score
999998899
Predicted solvent accessibility
420241235
H: Helix; S: Strand; C: Coil
BM95 FUpred. Contact map-based domain partition
Predicted domain boundary on contact map
FU-score curve of continuous domain
FU-score heatmap of discontinuous domain
Residue index
FU-score
SUB FUpred. Contact map-based domain partition
Predicted domain boundary on contact map
FU-score curve of continuous domain
FU-score heatmap of discontinuous domain
Residue index
FU-score

S6 Fig. Predicted secondary structure and solvent accessibility in SUB-BM95 interacting regions obtained from the *in music* model. Template modeling (eTM)-score, contact map-based distance (eRMSD), and confidence score are shown. *In silico* models were constructed using Swiss-Model (https://swissmodel.expasy.org), I-TASSER-MTD (https://zhanggroup.org/I-TASSER-MTD/) and Fupred contact map-based domain partition (https://zhanggroup.org/FUpred/). As described on the web page, FUpred is a contact map-based domain prediction method which utilizes a recursion strategy to detect domain boundary based on predicted contact-map and secondary structure information. Large scale benchmark analysis shows that FUpred has significantly better ability of domain boundary prediction than threading-based method and machine learning-based methods. Particularly, FUpred has excellent performance in detecting discontinuous domain boundary.

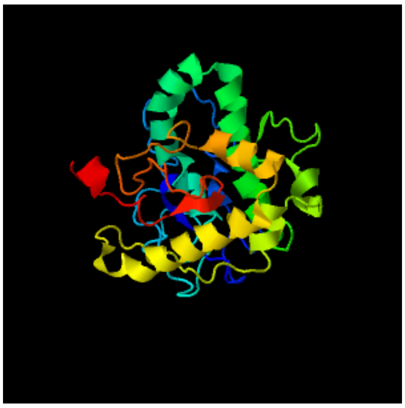

eTM-score = 0.35 ± 0.12, **eRMSD(Å)** = 13.6 ± 4.0, **P-score** = 0.4

Predicted secondary structure

Sequence
MACATLKRTHDWDPLHSPNGRSPKPSPFGEVPPKSSPLESGSPSATPPASPTGLSPGGLLSPVR
RDQPLFTFRQVGLICERMMKERESQIRDEYDHVLSAKLAEQYDTFVKFTYDQIQKRFEGATPSY
LSHKPFGSPSSPSSSAIAAAAAAAKRPSPFAEAVCPKQLTFNTGSRPDSPPSMVLFTFKQALRE
QYDAVLTNKLAEQYDAAAPSYLSRVQKGTVLCECPWNQHLVGDTCISDCVDKKCHE

Prediction
CCCCCCCCCCCCCCCCCCCCCCCCCCCCCCCCCCCCCCCCCCCCCCCCCCCCCCCCCCCCCCCC
CCCCCCCHHHHHHHHHHHHHHHHHHHHHHHHHHHHHHHHHHHHHHHHHHHHHHHHHHCCCCCCC
CCCCCCCCCCCCCCCCCCCCCCCCCCCCCHHHHHCHHHCCCCCCCCCCCCCCCCCCCHHHHHHH
HHHHHCCCCHHHHHHHHCCCCCCSSSCCCCSSSCCCCCCSCCCSSCCCCCCCCCCC

Conf.Score
9865568888877666899999998876678999982335766678999998888887766777 6
7876578999999999999999999999999988999999999998899999996258986 4
4678889998888765444555556776145542355414443345677886432022113455
54321355189999871577531223486122278642102872220226766678 9

H: Helix; S: Strand; C: Coil

Predicted solvent accessibility

Sequence
MACATLKRTHDWDPLHSPNGRSPKPSPFGEVPPKSSPLESGSPSATPPASPTGLSPGGLLSPVR
RDQPLFTFRQVGLICERMMKERESQIRDEYDHVLSAKLAEQYDTFVKFTYDQIQKRFEGATPSY
LSHKPFGSPSSPSSSAIAAAAAAAKRPSPFAEAVCPKQLTFNTGSRPDSPPSMVLFTFKQALRE
QYDAVLTNKLAEQYDAAAPSYLSRVQKGTVLCECPWNQHLVGDTCISDCVDKKCHE

Conf.Score
7314424252514435536664454543472556245564456455446345444444444545
5643301152023004400662364125304510454145224112413351035417745241
2454326444375444454454454444314412355324645543644344243232552035
41543252533543631352531404663230403354312645025503665158

Values range from 0 (buried residue) to 8 (highly exposed residue)

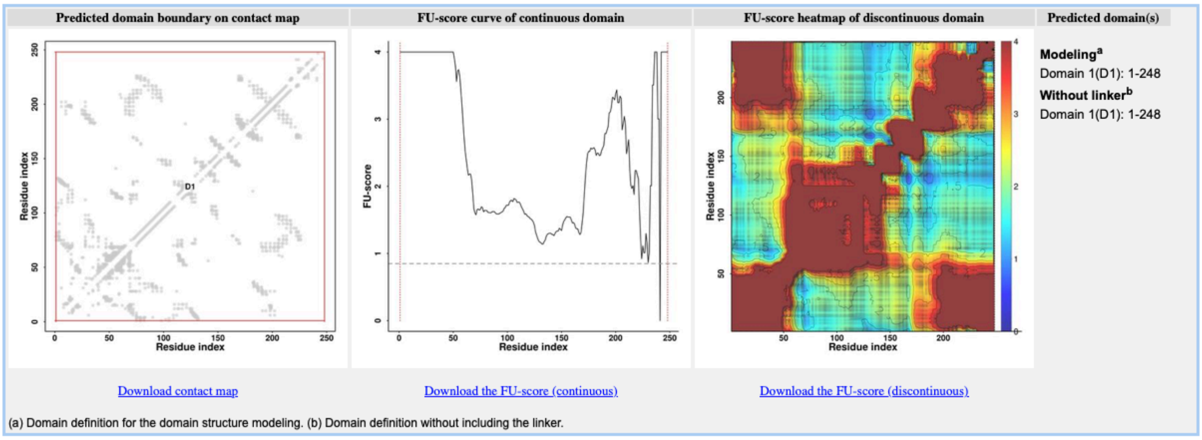

(a) Domain definition for the domain structure modeling. (b) Domain definition without including the linker.

S7 Fig. Predicted secondary structure and solvent accessibility in chimeric antigen Q38-95. Template modeling (eTM)-score, contact map-based distance (eRMSD), and confidence score are shown. *In silico* model was constructed using I-TASSER-MTD (https://zhanggroup.org/I-TASSER-MTD/).

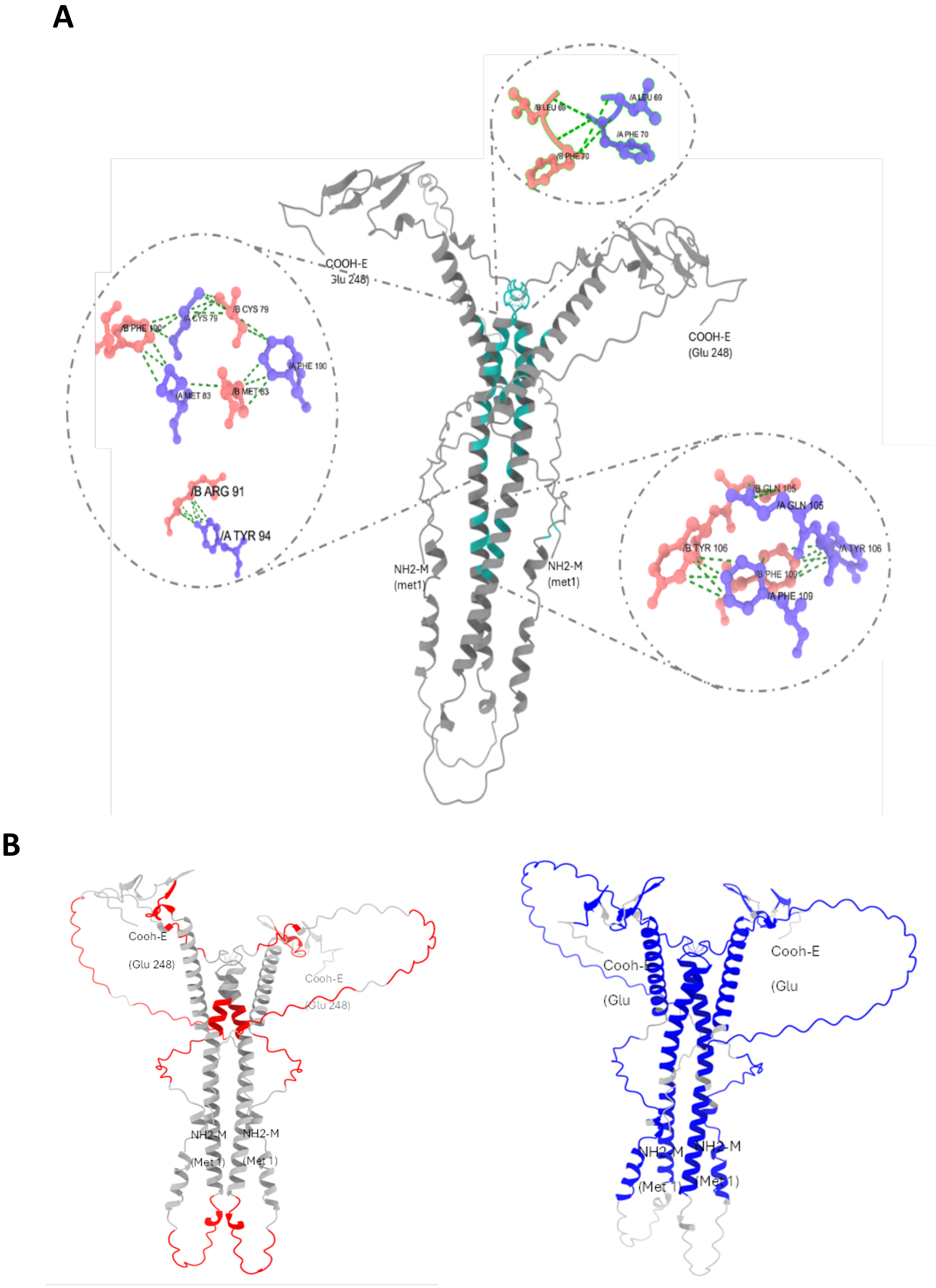
A
/B LEU 69
/A LEU 69
/A PHE 70
/B PHE 70
COOH-E
(Glu 248)
/B PHE 190
/A CYS 79
/B CYS 79
/A PHE 190
/A MET 83
/B MET 83
/B ARG 91
/A TYR 94
/B GLN 105
/A GLN 105
/B TYR 106
/A TYR 106
/B PHE 109
/A PHE 109
NH2-M
(met1)
B
Cooh-E
(Glu 248)
NH2-M
(Met 1)
(Glu

S8 Fig. Analysis of Q38-95 self-interaction using AlphaFold algorithms. (A) Structure analysis of the chimeric antigen Q38-95 dimer interacting zones. Interacting zones are zoomed to show the amino acid pairs in contact. (B) Epitope distribution for T-cells (Blue) and B-cells (red) on the Q38-95 dimers predicted structure. AlphaFold predicts protein structures from a single sequence using machine learning, combining orthology and sequence inputs. It starts with a multiple sequence alignment (MSA) of the input sequence against a database, which is then used to create a distance matrix, plotting distances between amino acid residues. This process determines the protein's 3D structure and confidence in predictions. AlphaFold provides structural accuracy metrics like the Predicted Local Distance Difference Test (pLDDT) for local reliability and the Predicted Aligned Error (PAE) for domain position confidence. In the latest versions, additional metrics like pTM and ipTM scores assess protein-protein complex structures, with high scores indicating confidence in the predictions. These metrics and others like pLDDT offer a comprehensive evaluation of predicted structures.

S1 Table. Number of Interfacial Contacts (ICs) per property (blind docking).

ICs charged-charged:	5
ICs charged-polar:	9
ICs charged-apolar:	27
ICs polar-polar:	0
ICs polar-apolar:	21
ICs apolar-apolar:	32

S2 Table. Interacting amino acid residues as defined by the guided docking procedure.

**BM95**	**Residue**	**Chain**	**SUB**	**Residue**	**Chain**
LYS	299	A	VAL	141	B
ASP	352	A	PHE	152	B
VAL	296	A	ARG	151	B
GLU	279	A	PHE	140	B
TRP	358	A	SER	158	B
HIS	272	A	TYR	137	B
VAL	375	A	ARG	151	B
CYS	300	A	PHE	152	B
PHE	351	A	SER	158	B
PRO	347	A	PHE	152	B
TRP	358	A	THR	156	B
LYS	273	A	SER	130	B
VAL	271	A	TYR	137	B
VAL	296	A	GLN	147	B
TRP	358	A	PRO	157	B
ASP	354	A	ARG	151	B
ARG	368	A	GLY	154	B

SER	353	A	PRO	157	B
CYS	356	A	THR	156	B
LYS	299	A	ILE	148	B
THR	275	A	TYR	137	B
ASP	352	A	ALA	155	B
SER	353	A	GLY	154	B
CYS	300	A	ARG	151	B
GLN	284	A	GLN	147	B
GLU	302	A	TYR	145	B
ARG	368	A	PHE	152	B
CYS	300	A	ILE	148	B
GLU	302	A	LYS	142	B
ARG	270	A	PHE	140	B
ARG	368	A	GLU	153	B
PHE	376	A	GLY	154	B
SER	350	A	TYR	159	B
GLU	279	A	VAL	141	B
LYS	299	A	THR	144	B
ARG	368	A	LYS	150	B
GLU	302	A	PHE	140	B
ARG	368	A	ARG	151	B
PHE	376	A	PHE	152	B
TRP	358	A	SER	161	B
HIS	301	A	PHE	152	B
SER	353	A	THR	156	B
TRP	282	A	PHE	140	B
CYS	300	A	THR	144	B
LYS	357	A	THR	156	B

GLU	302	A	VAL	141	B
LEU	277	A	TYR	137	B
SER	350	A	SER	158	B
ASP	354	A	THR	156	B
PHE	351	A	THR	156	B
GLU	279	A	TYR	137	B
ARG	368	A	ALA	155	B
HIS	272	A	LEU	133	B
ASP	352	A	TYR	159	B
ASP	361	A	THR	156	B
LYS	357	A	GLY	154	B
LYS	299	A	PHE	140	B
PHE	351	A	PRO	157	B
CYS	280	A	PHE	140	B
ASP	354	A	GLY	154	B
HIS	272	A	ALA	134	B
VAL	276	A	TYR	137	B
HIS	301	A	ILE	148	B
ASP	352	A	SER	158	B
PHE	376	A	ALA	155	B
GLU	302	A	ILE	148	B
ASN	348	A	TYR	159	B
CYS	300	A	GLN	147	B
LYS	299	A	TYR	145	B
CYS	356	A	SER	158	B
TRP	282	A	PHE	143	B
ASP	352	A	THR	156	B
ARG	270	A	TYR	137	B

ASP	352	A	PRO	157	B
ASP	297	A	ARG	151	B
VAL	296	A	THR	144	B
SER	353	A	ALA	155	B
GLU	302	A	THR	144	B
CYS	356	A	PRO	157	B
PHE	376	A	ARG	151	B
HIS	301	A	ARG	151	B
SER	293	A	ARG	151	B
ASP	354	A	ALA	155	B

S3 Table. HADDOCK2.2 results for the best and thus more reliable cluster of dockings.

HADDOCK score	-101.6 +/- 7.9
Cluster size	5
RMSD from the overall lowest-energy structure	1.1 +/- 0.8
Van der Waals energy	-70.3 +/- 6.0
Electrostatic energy	-192.4 +/- 41.7
Desolvation energy	-32.9 +/- 10.6
Restraints violation energy	401.3 +/- 71.66
Buried Surface Area	2193.9 +/- 196.6
Z-Score indicates how many standard deviations from the average this cluster is in terms of score (the more negative the better)	-1.6
Binding affinity (ΔG)	-13.6 kcal mol^{-1}
Dissociation constant (Kd)	1.1E-10 at 25 °C

S4 Table. Number of Interfacial Contacts (ICs) per property (guided docking).

**Number of Interfacial Contacts (ICs) per property**	
ICs charged-charged:	7
ICs charged-polar:	9
ICs charged-apolar:	32
ICs polar-polar:	3
ICs polar-apolar:	17
ICs apolar-apolar:	15
**Non-Interacting Surface (NIS) per property**	
NIS charged:	27.95%
NIS apolar:	34.72%

S1 Data. ***In silico* model 3.** Modelling BM95-SUB interactions with Alphafold-HADDOCK algorithms. All interaction energies were obtained as an average LJ + electrostatics over the 1μs MD simulation.

Interaction energy for the in-silico model 1 (AlphaFold). Total interaction energy -298.9 kcal/mol (-0.41 kcal/mol/residue).

**BM95**	**subolesin**	**Interaction energy (kcal/mol)**
ARG270	GLU56	-9.15
ARG145	ASP42	-8.77
ARG116	ASP42	-7.86
ASP361	ARG104	-7.72
ASP98	ARG37	-7.18
GLU360	ARG104	-7.17
ASN330	GLN74	-3.38
LYS142	HIS39	-3.21
ASN328	GLU65	-3.01

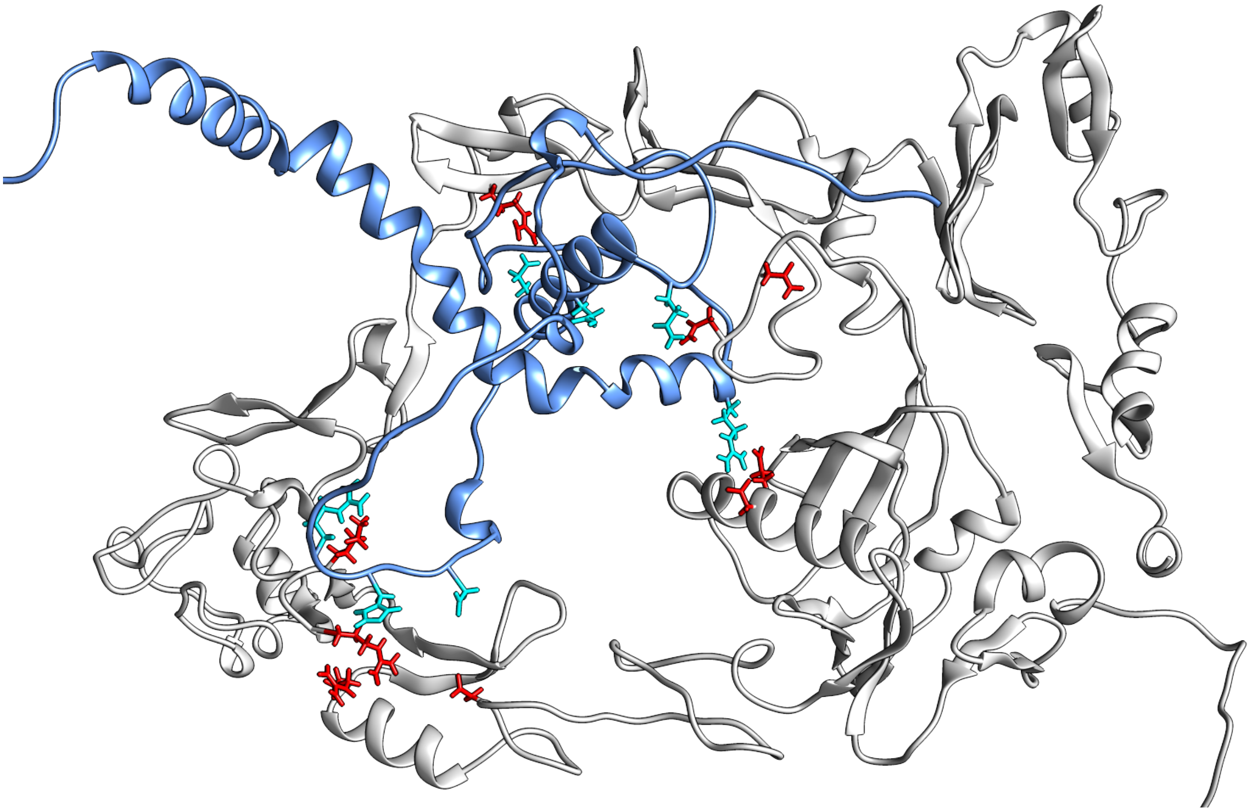

Interaction energy for the in-silico model 2 (AlphaFold). Total interaction energy -378.0 kcal/mol (-0.52 kcal/mol/residue).

**BM95**	**subolesin**	**Interaction energy (kcal/mol)**	**BM95**	**subolesin**	**Interaction energy (kcal/mol)**
ARG569	ARG27	-6.91	GLU558	ARG21	-3.83
ASP26	ARG8	-6.18	LYS321	ASP138	-3.80
ASP361	ARG104	-5.41	GLU30	LYS7	-3.75
ARG390	GLU135	-5.32	TYR317	THR139	-3.60
ARG569	ARG25	-4.95	ARG323	GLU135	-3.41
TRP282	TYR125	-4.67	TYR310	PHE143	-3.38
ARG270	GLU56	-4.67	TRP282	GLH124	-3.32
SER322	ASP138	-4.19	GLU566	LYS24	-3.09
GLU20	ARG8	-3.84	GLU16	ARG8	-3.06

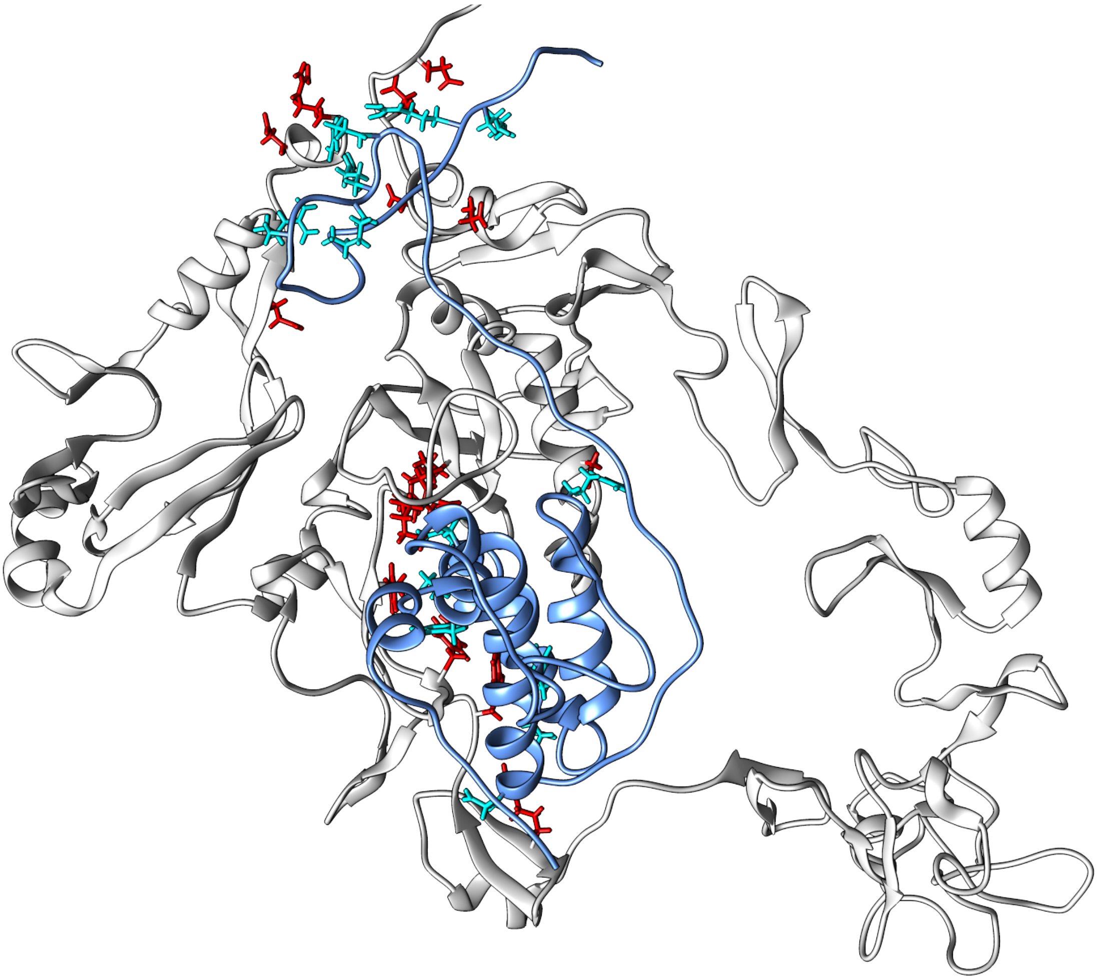

Interaction energy for the in-silico model 3 (AlphaFold). Total interaction energy -192.0 kcal/mol (-0.26 kcal/mol/residue).

BM95	subolesin	Interaction energy (kcal/mol)
ASP352	ARG112	-5.21
GLU333	GLU135	-3.99
GLU333	TYR137	-3.43
ASP354	ARG112	-3.40
ARG270	GLU118	-3.29
TRP282	ARG122	-3.09
GLU379	ARG104	-3.01

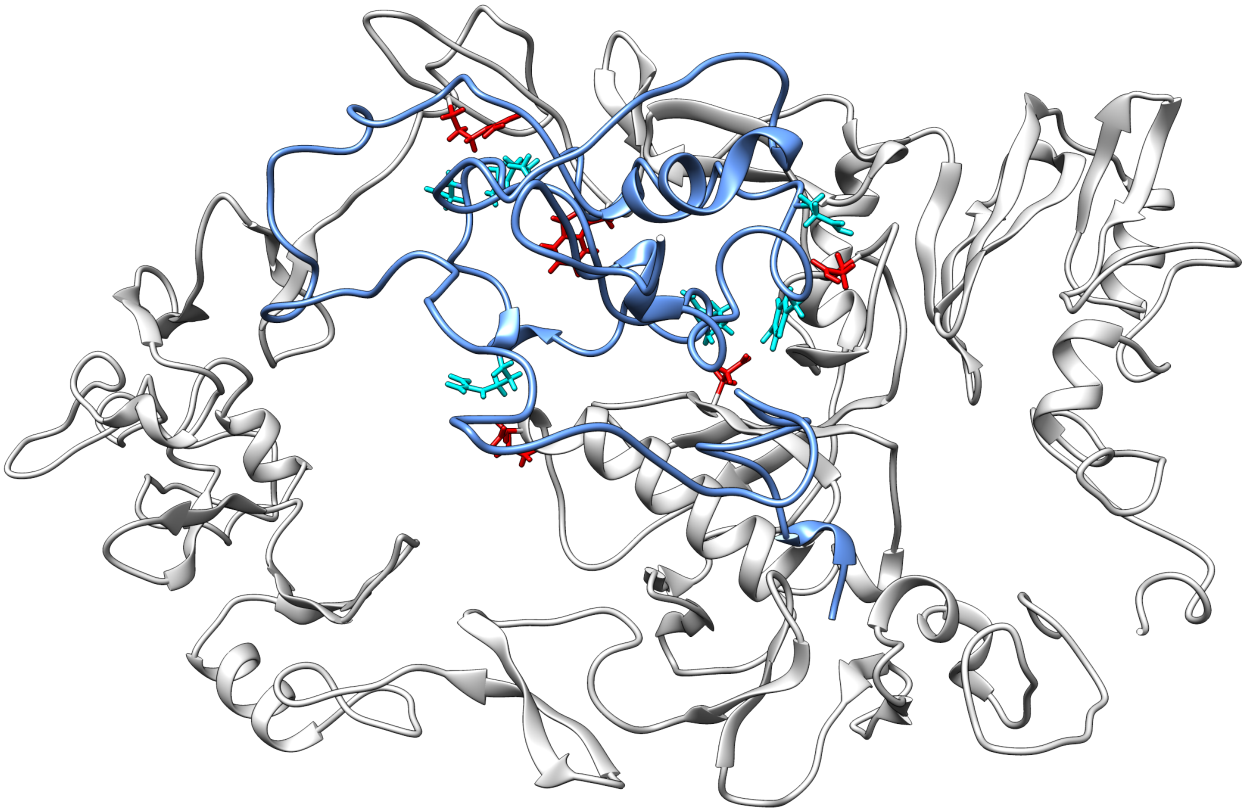

Interaction energy for the in-silico model 4 (AlphaFold). Total interaction energy -222.2 kcal/mol (-0.30 kcal/mol/residue).

BM95	subolesin	Interaction energy (kcal/mol)
ARG334	ASP146	-8.59
ASP354	ARG112	-7.91
ARG478	SER161	-6.98
ASP125	ARG72	-3.74
TYR310	PHE103	-3.17

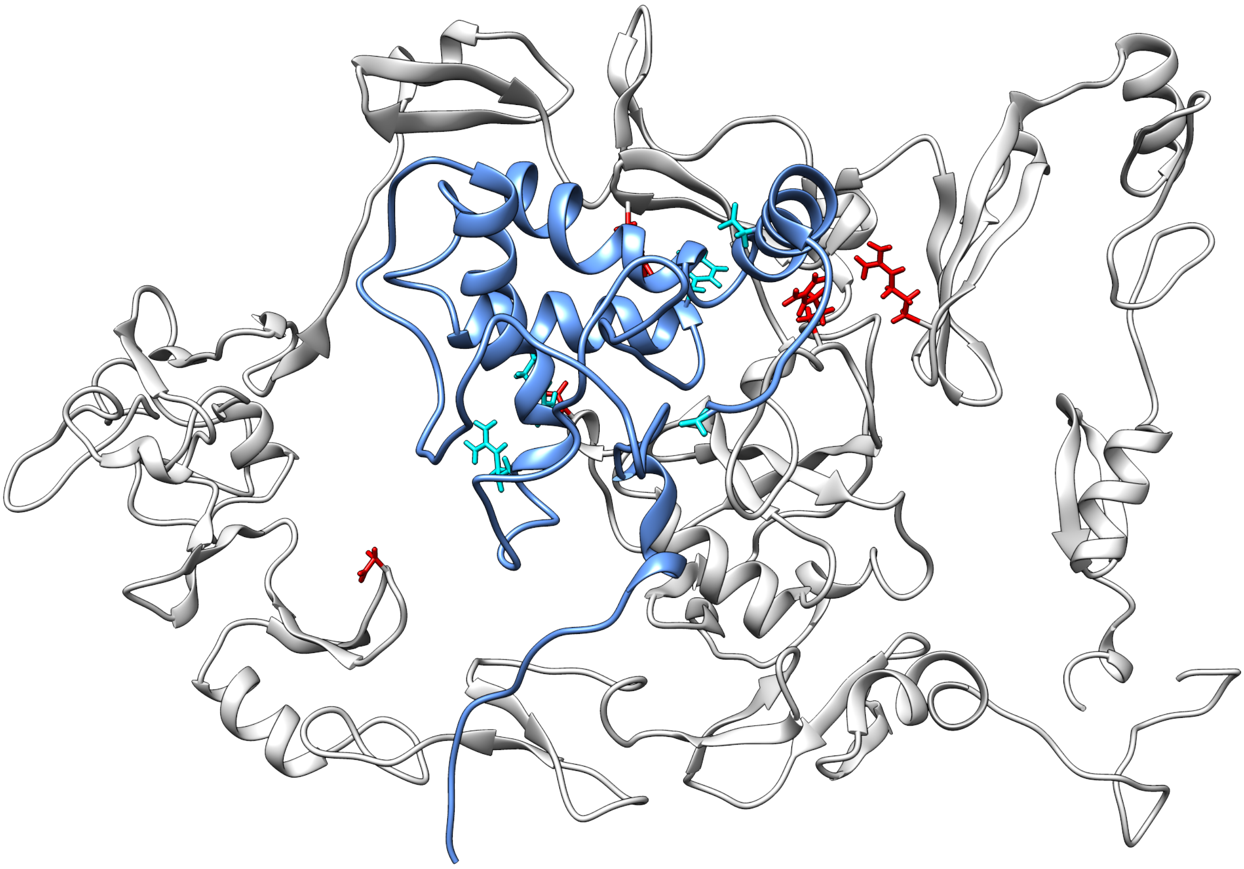

Interaction energy for the in-silico model 5 (HADDOCK). Total interaction energy -253.7 kcal/mol (-0.35 kcal/mol/residue).

BM95	subolesin	Interaction energy (kcal/mol)
GLU360	ARG27	-6.96
ASP361	GLU57	-4.38
MET311	TRP12	-3.93
ARG368	GLU56	-3.85
TRP282	MET29	-3.68
GLU379	ARG27	-3.36
ASP354	ARG25	-3.00

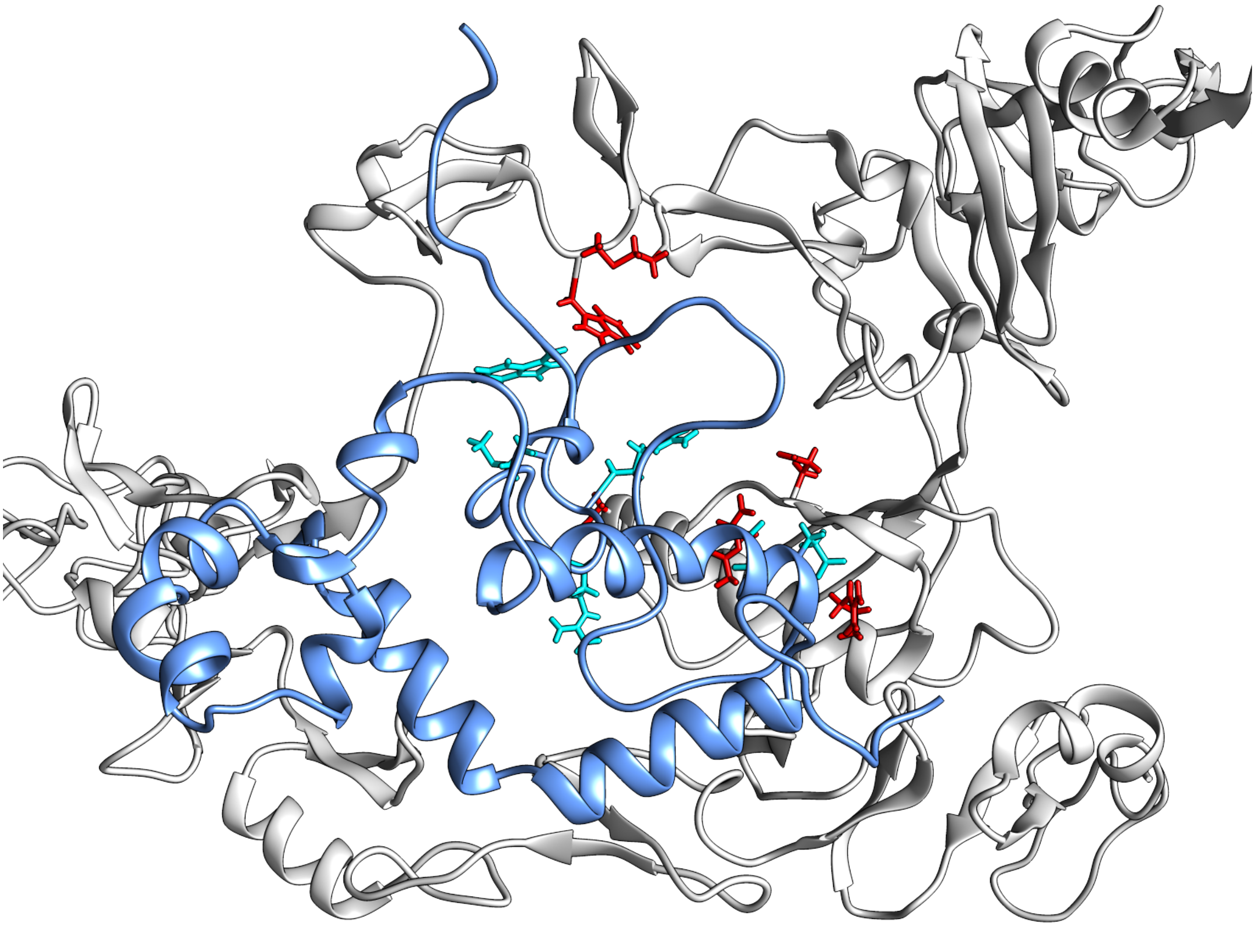

Interaction energy for the in-silico model 6 (HADDOCK). Total interaction energy -198.0 kcal/mol (-0.27 kcal/mol/residue).

BM95	subolesin	Interaction energy (kcal/mol)
ARG80	GLU123	-7.92
GLU62	LYS52	-7.65
GLU360	ARG27	-5.09
GLU94	ARG122	-4.18
ASN328	GLU56	-4.06
ARG123	GLU116	-3.87
ARG80	ASP126	-3.65
ASP125	LYS120	-3.54
LYS72	ASP48	-3.46
PHE127	LYS120	-3.45
ARG358	CYS28	-3.43
ASP361	ARG26	-3.23

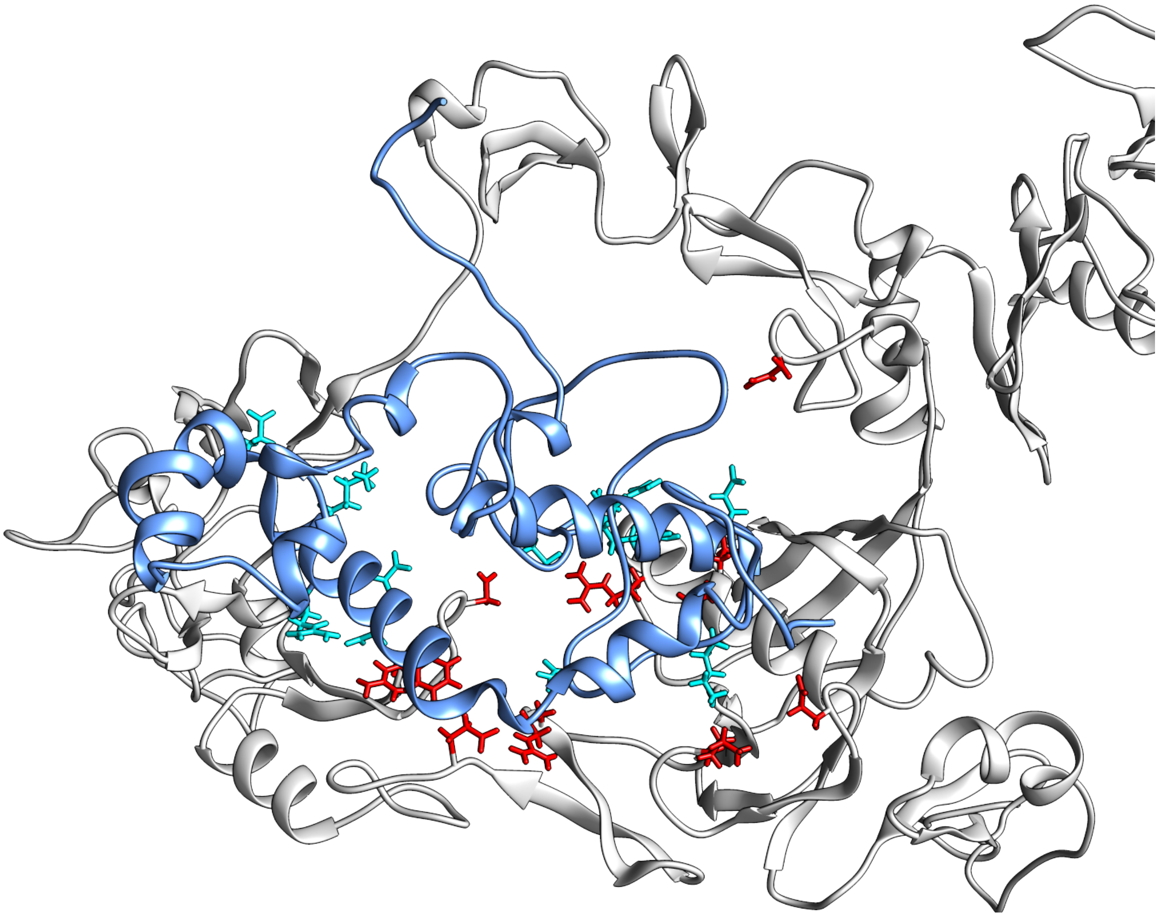

Interaction energy for the in-silico model 7 (HADDOCK). Total interaction energy -319.5 kcal/mol (-0.44 kcal/mol/residue).

BM95	subolesin	Interaction energy (kcal/mol)	BM95	subolesin	Interaction energy (kcal/mol)
ASP268	ARG104	-8.79	LYS219	GLU118	-3.86
ARG323	ASP97	-8.32	GLU303	ARG151	-3.78
ARG80	GLU83	-7.82	ASN328	ASP97	-3.67
LYS421	ASP81	-7.58	ASP125	ARG117	-3.54
ARG334	GLU153	-6.42	ARG80	ASP81	-3.45
GLU38	MET1	-5.07	CYX37	MET1	-3.43
LYS247	GLU111	-4.25	PRO327	ARG96	-3.26
ARG362	ASP81	-4.22	LYS373	ASP11	-3.25
GLU62	ARG21	-4.06	TRP282	LEU100	-3.12
SER248	GLU111	-4.01	ASP125	LYS120	-3.01

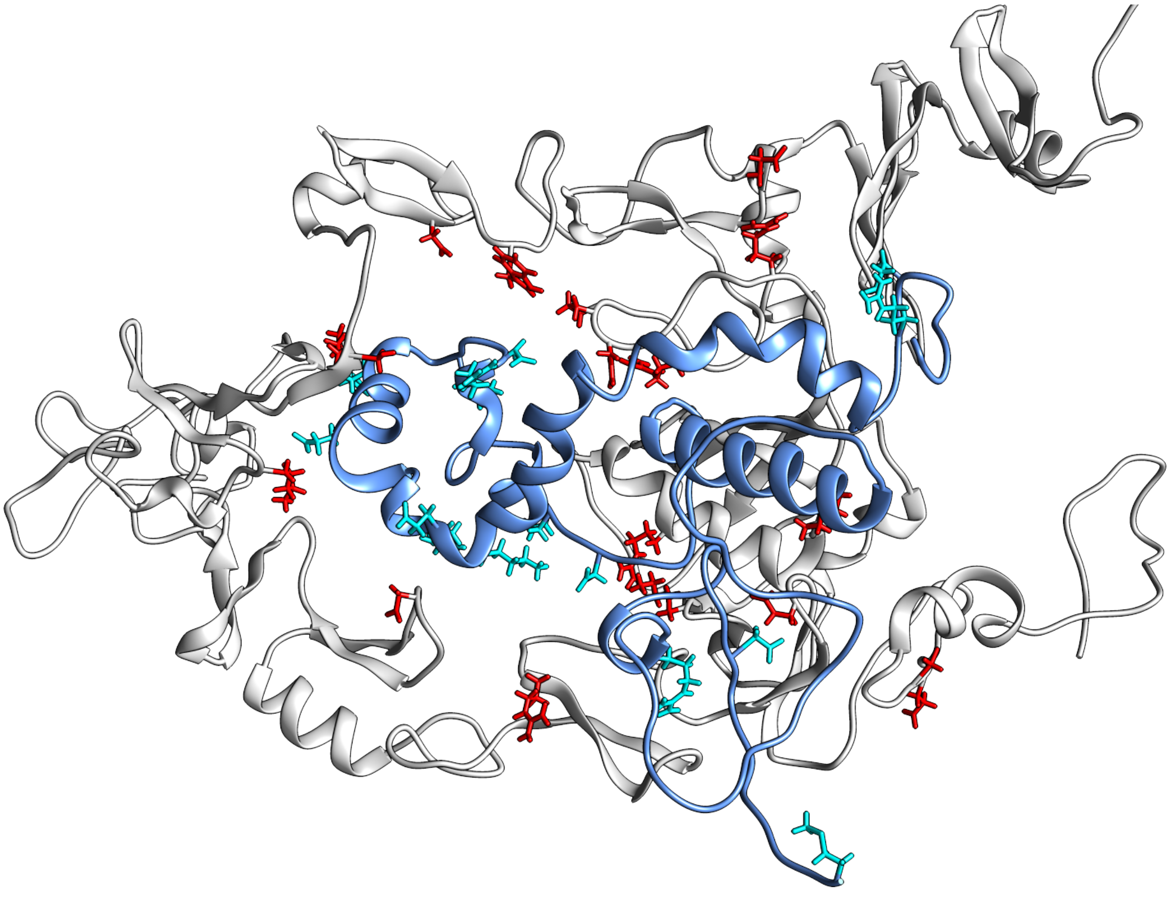

Interaction energy for the in-silico model 5 (HADDOCK). Total interaction energy -188.7 kcal/mol (-0.26 kcal/mol/residue).

BM95	subolesin	Interaction energy (kcal/mol)
ARG123	ASP126	-8.28
ARG323	GLU56	-5.44
ASP354	ARG27	-5.29
ASP125	ARG71	-4.80
HIS220	GLU116	-3.78
ARG80	TYR137	-3.71
ARG123	GLU123	-3.66
LYS377	GLU56	-3.66
LYS142	GLU111	-3.50
TYR251	LYS7	-3.02

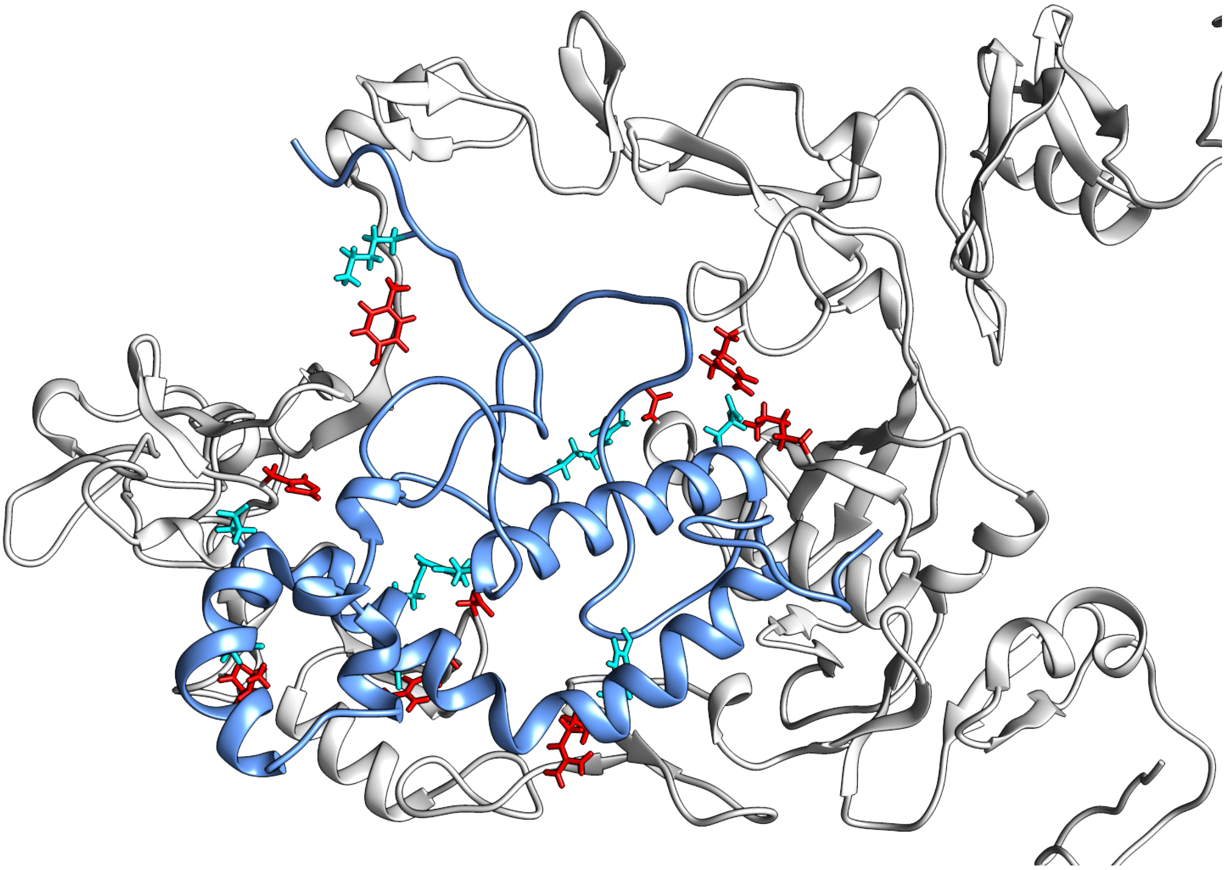

Summary of AlphaFold-predicted BM95-subolesin complexes.

BM95	# of occurrences	subolesin	# of occurrences	Residue pairs	# of occurrences
ARG270	3	ARG104	4	ARG270 GLU56	2
TRP282	3	ARG112	3	ASP354 ARG112	2
ARG569	2	ARG8	3	ASP361 ARG104	2
ASP354	2	GLU135	3		
ASP361	2	ASP138	2		
GLU333	2	ASP42	2		
TYR310	2	GLU56	2		

Summary of HADDOCK-predicted BM95-Subolesin complexes.

BM95	# of occurrences	subolesin	# of occurrences	Residue pairs	# of occurrences
ARG80	5	ARG27	4	ASP125 LYS120	2
ASP125	4	GLU56	4	GLU360 ARG27	2
ARG123	3	ASP81	3		
ARG323	2	GLU111	3		
ASN328	2	LYS120	3		
ASP354	2	ASP126	2		
ASP361	2	ASP97	2		
GLU360	2	GLU116	2		
GLU62	2	GLU123	2		
TRP282	2	MET1	2		

S2 Data. Analysis of musical algorithms applied to SUB-BM95 interactions.

Musical sections in MT241515.1 SUB and AF150891.2 BM95.

**SUB**
Measures 1-6. A short introductory melodic line articulated on the pitch *do (C)*.
Measures 6-27. A polarized section on the pitch *re (D).*
Repetition of the pitch *re (D)*: Ms. 11, 13, 25 and 27
Repetition of the *sol-si-la (G-B-A)* motif: Ms. 17 and 19
a. Repetition of the *mi-re-re (E-D-D)* motif: Ms. 18 and 23
b. Redundant gesture around pitch *re (D)*. Ms. 24-27
Measures 28-47. A polarized section on the pitch *mi (E)*
a. Repetition of the *mi-re-la (E-D-A)* motif: Ms. 30 and 34
b. Repetition of the motif *mi-re-mi-re (E-D-E-D)*: Ms. 43 and Ms. 45
c. The following belong to the same family and are of the same pitch: Ms. 29, 30, 33, 34, 34, 35, 36, 40, 43 and 45
Measures 48-82. A polarized section in pitch *re (D)*
Repetition of the *mi-re (E-D)* motif: Ms. 50-51
a. Repetition of the *fa-sol-re (F-G-D)* motif: Ms. 56-57, 64-65, in the same order
b. Repetition of the *re-sol-la (D-G-A)* motif: Ms. 68-71
c. It presents three very pronounced melodic lines: Ms. 56-59 (ascending), Ms. 64-68 (descending), Ms. 72-76 (ascending)
Measures 83-112. A polarized section in pitch *sol (G)*
a. Repetition of the *re (D)* motif Ms. 96 and 112
b. Two-motif repetition: *sol-la-si-la / la-sol (G-A-B-A / A-G)*: Ms. 89-90 and 107-108
c. Presentation of a very marked melodic line: Ms. 88-91
Measures 112-137. A polarized section in *re (D)*
a. Repetition of the *mi (E)* motif. Ms. 113 and 114
b. Repetition of the *fa-sol-re (F-G-R)* motif. Ms. 116-118 and 123 and 124
c. Repetition of the *re (D)* motif: Ms. 112, 117 and 126
d. Repetition of the *re-do-re (D-C-D)* motif: Ms. 115, 120 and 132
Measures 138-146. A polarized section in *do (C)*
a. Repetition of *do (C)* motif: Ms. 138 and 146
b. It is a phrase with a melodic ascent (Ms. 138-142) and a melodic descent (Ms. 143-146)
Measures 144-161. A heterogeneous final section that tends to *re (D)*
a. The important pitchs mentioned above stand out. *re, mi, sol, do* (*D*, *E*, *G*, *C)* with an ending in *re* (*D*)
**BM95**
Introduction: Ms. 1-24
a. Reiteration of the *sol-la-do-re (G-A-C-D)* motifs Ms. 3 and 17.
Measures 24-48. (24 measures, 8 x 3)
a. Reiteration of the *si-si-la (B-B-A)* motifs Ms. 32, 37, 51
b. Reiteration of the *fa-sol (F-G)* motif: Ms. 36 and 38
c. Reiteration of *si-la-sol-fa (B-A-G-F)* motifs Ms. 39, 40 and 48
d. Motif link to the following section: *do-si-do (C-B-C)* Ms. 45, 46 and 54
Measures 49-65 (16 measures)
a. Linking motif with the preceding section: *do-si-do (C-B-C)*. Ms. 45, 46 and 54

b. Reiteration of the *do-re-la (C-D-A)* motif: Ms. 40-41
Measures 66-81 (Ms. 16)
Measures 82-113 (Ms. 32)
Measures 114-132 (Ms. 19)
Measures 133-147 (Ms. 15)
a. Reiteration of the *sol-la-si-la (G-A-B-A)* motif (Ms. 135 and 138)
Measures 148-163 (Ms. 16)
a. Reiteration of *fa-mi-mi-re (F-E-E-D)* motif Ms. 143 above and 150, 151 and 158
Measures 164-193 (Ms. 20)
a. Reiteration of the *do-re-la (C-D-A)* motif (Ms. 186, 189 and below 214)
b. Reiteration of the *mi-re-la (E-D-A)* bridging motif (Ms. 187 and 195)
Measures 194-209 (Ms. 16)
a. Relationship with previous bridging motifs: *do-re-la (C-D-A)* (Ms. 186, 189 and later 214) and *mi-re-la (E-D-A)* (Ms. 187 and 195)
Measures 210-218 (8 section) linked to the previous section (Ms. 214) and to 9 section (Ms. 214)
Measures 219-267. There are regular interventions of the amino acid C (pitch B) in the following succession of measures: 4 – 9 – 15 – 9 – 8 and 6
a. The vertebral motif, which is usually repeated, is *fa-mi-mi-re (F-E-E-D)* motif, with support almost always in *si (B)*: Ms. 215, 232, 237, 245, 262 and 266
Measures 268-307. It presents a double structuring for the ear, which can be based on either *si* or *do (B* or *C)*
a. Periods on *si* (*B*), ranging between Ms. 15 and 16
b. The periods on *do* (*C)* are symmetrical: Ms. 8 + 8 = 16
c. The *si-la-sol-fa (B-A-G-F)* motif is reiterated: Ms. 271-276-287 and 343 below.
d. The *fa-sol (F-G)* motif is reiterated: Ms. 302-303
Measures 308-378. Regularity is provided not by long rests, but by the reiteration of melodic motifs
a. The *sol-la-do-re (G-A-C-D)* motif is reiterated: Ms. 308-326
b. The r*e-mi-si-do (D-E-B-D)* motif is reiterated: Ms. 312-328-332-337
c. The *la-sol-si-do (A-G-B-C)* motif is reiterated: Ms. 339-340-359
d. Reminiscence of the *si-la-sol-fa* (B-A-G-F) motif: Ms. 343 (formerly in 271, 276, 287)
e. Beginning of the *re-do-re* (D-C-D) motif (Ms. 377), which will have an impact on the next section (Ms. 377, 388,379, 389 421, 425, 443 and 457)
Measures 379-455
a. The separation at regular intervals of *si (B)* (amino acid C): from Ms. 383 to 386 (32 + 1 Ms.), from Ms. 387 to 438 (22 Ms.), from Ms. 439 to 455 (17 Ms.)
b. A reiteration of motifs can be observed: Reiteration of the *re-do-re (D-C-D)* motif (Ms. 377) which will have its impact on the next section (Ms. 377, 388, 379, 389, 421, 425, 443 and 457); in this last interval there are 17 Ms. away
c. Reiteration of the *fa-sol-re (F-G-D)* motif: Ms. 379-394-409. Interestingly, the distribution between these is 16 and 15 Ms., respectively.
d. Reiteration of the *mi-mi (E-E)* motif: Ms. 417-418
Measures 456-492 (37 Ms. between *si* and *si* (*B* and *B*)
a. Reiteration of *si-la-sol* (*B-A-G*) motifs Ms. 456, 458 and 485
b. Reiteration of the *fa-mi* (*F-E*) motifs Ms. 471, 472 and 477
c. Reiteration of the *re-do-re* (*D-C-D*) motifs Ms. 480, 482 and 483

Measures 493- 569. Very regular final section, separated into very regular periods by the appearance of the pitch *si (B)*. These periods are: Ms. 493-500, 8 Ms.; Ms. 501-508, 8 Ms.; Ms. 509-516, 8 Ms.; Ms. 517-529, 13 Ms.; Ms. 530-561, 32 Ms.; Ms. 562-560, 8 Ms.
All these sections are articulated by the following recurrent motifs
a. Reiteration of *re-mi* (*D-E)* motifs Ms. 492, 494, 502, 511, 536, 554
b. Reiteration of the *fa-sol-re (F-G-D)* motifs Ms. 526, 531, 539, 549 and 566

The SUB Ms. 138-146 have a very stable section in C as the melodic phrase begins, rests, and closes its structure on that pitch. Moreover, this melodic line not only presents a bimember design that can be divided into two sections, (Ms. 138-142 and 143-146), but it has an ascending-descending profile of remarkable configuration and melodic success. On the other hand, and this is very significant, an examination of the possible harmonic conditions of this section gives us an authentic functional harmonic sequence, with an extensive development of the subdominant function: Ms. 138: I; Ms. 139-140: IV / II; Ms. 141. V; Ms. 142. I [with apoggiatura of *re* (*D*) by do (C)]; Ms. 143-145. IV; Ms. 145. V [in *sol* (*G*)]; Ms. 146. I.

Cadential progression and audio (Supplementary audio file 4. Cadential.wav).

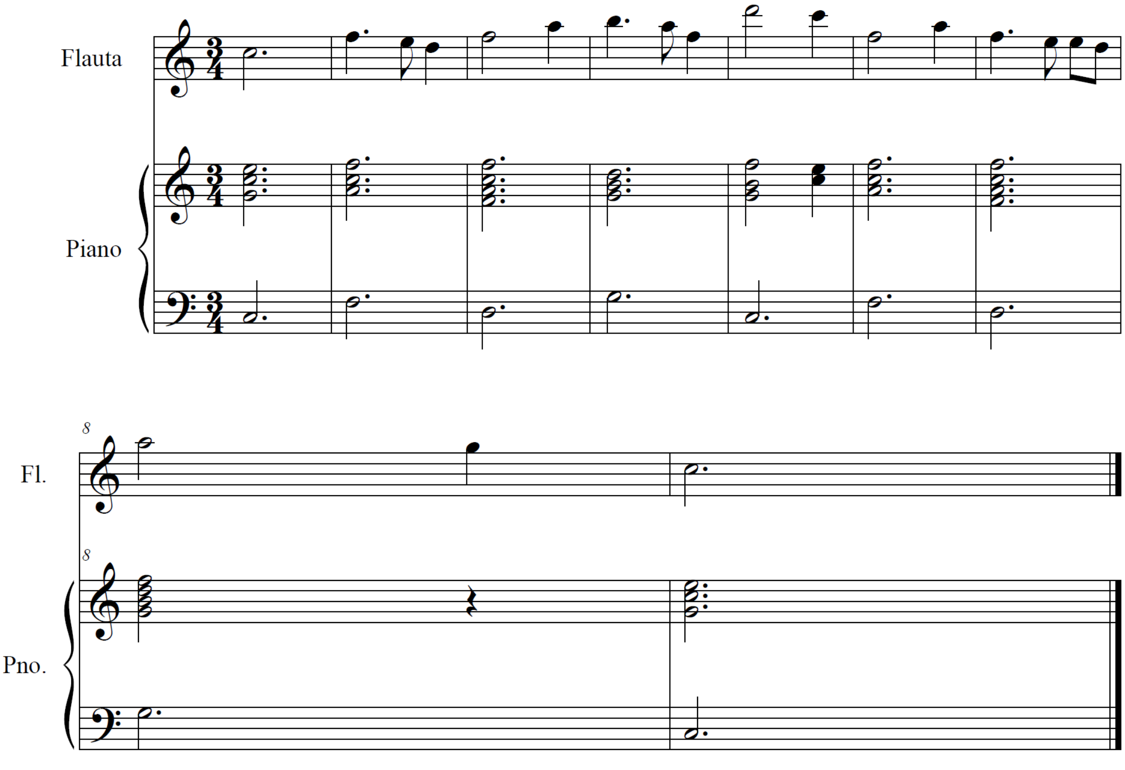

This strong stability, perhaps not so evident in other sections, has a regular 9-bar dimension, although the first and last bars are the same and could be constituted as a self-replicating 8-bar melody in itself.

Having also examined section 13 of BM95 (Ms. 268-307), we saw that there was a double polarity in *B* and *C*, and that the periods in *C* were symmetrical (8 + 8 measures). If, from a

cadential perspective, we understand the *B* as the seventh note (dominant function), we can replicate SUB Ms. 138-145 up to four times, seeing that it is possible to combine both melodies from a contrapuntal perspective, given that the consonant intervals between both voices are very frequent, predominantly octave intervals, sixths, thirds, and sometimes fourths and fifths.

Musical SUB-BM95 interactions. Melodic interactions are highlighted in similar colors and can be listen in Supplementary audio file 3.

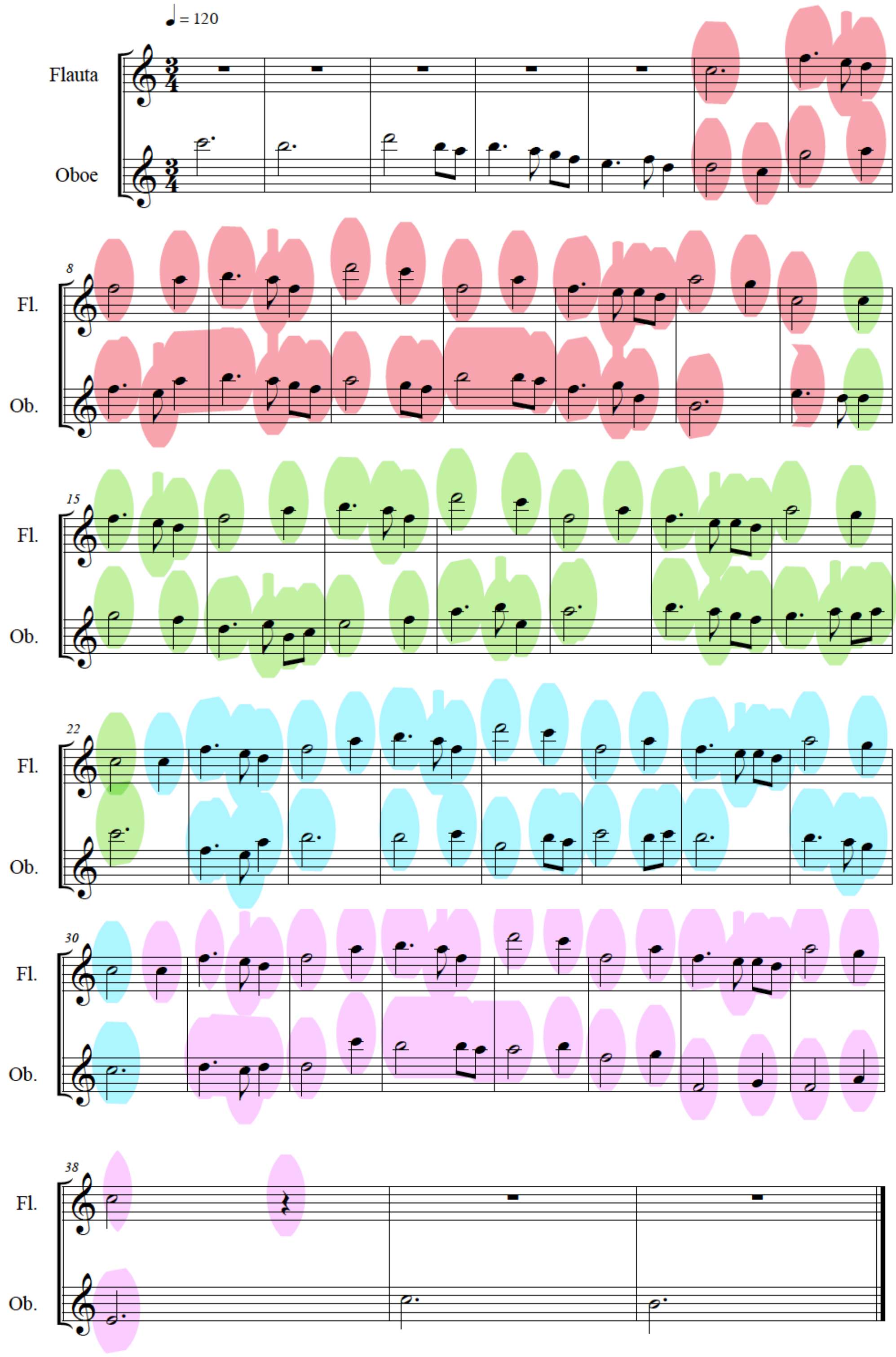

What is even more striking is that this interplay between the two voices has great beauty when accompanied by the cadential functions that are characteristic of SUB, and which, as we pointed out, has a strong harmonic functional structure as shown by the simple harmonisation we proposed. If we now project the harmonic support on the interaction of both voices, the result of the harmonization is extraordinarily solid and compact.

Results of the harmonization and audio (Supplementary audio file 5. Harmonization.wav).

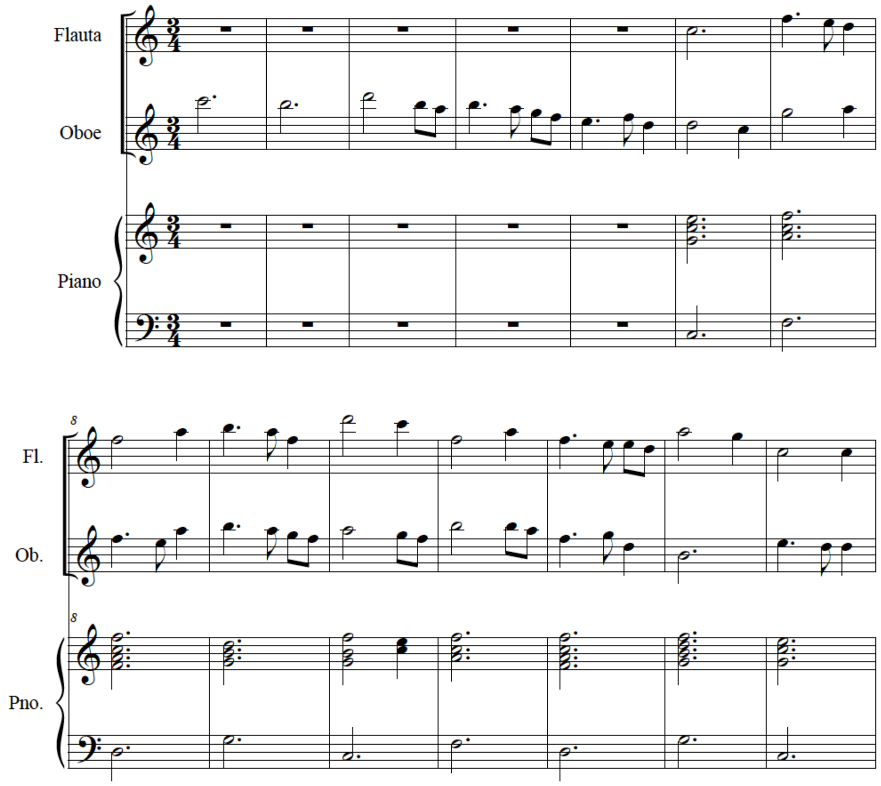

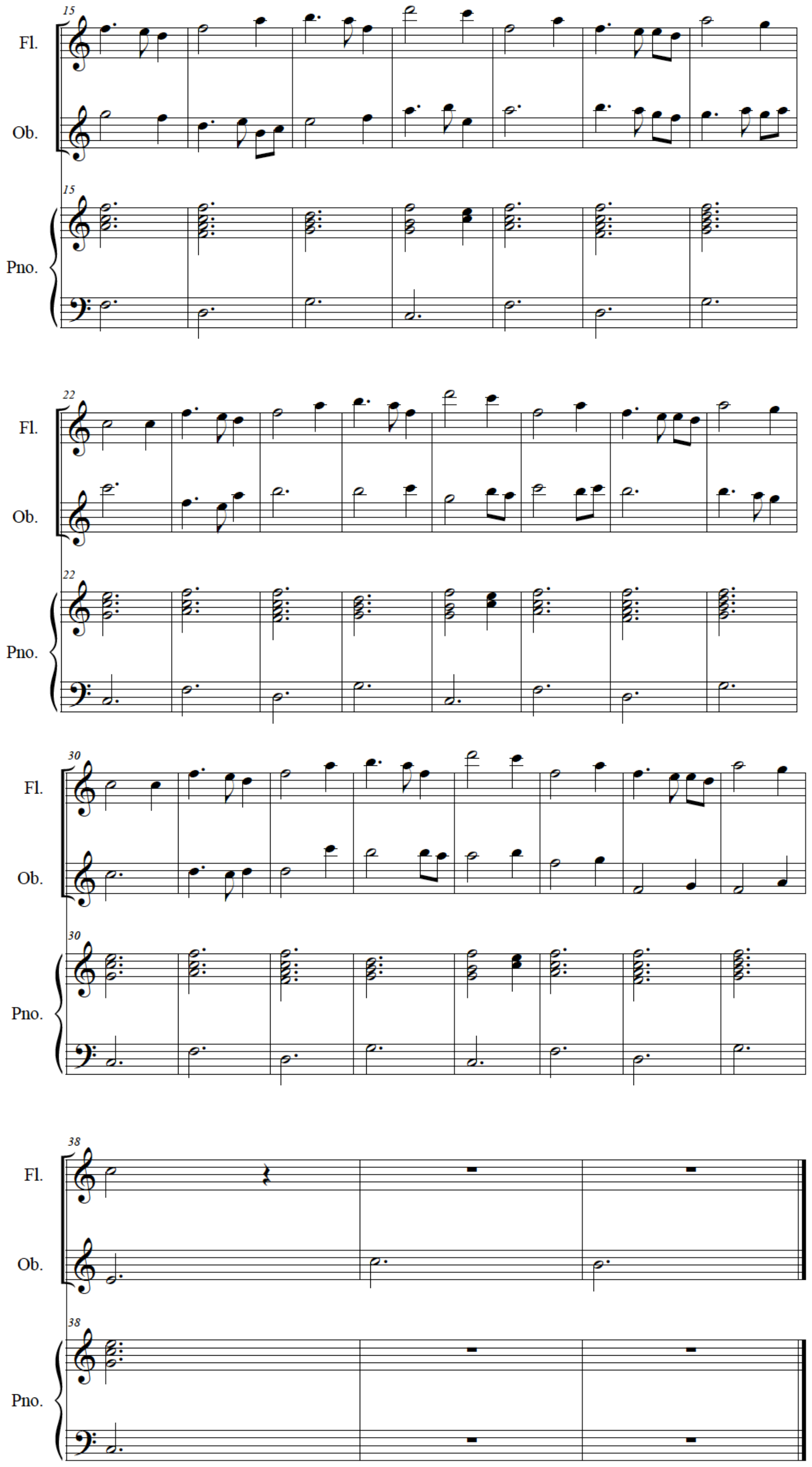
15
Fl.
Ob.
15
Pno.
22
Fl.
Ob.
22
Pno.
30
Fl.
Ob.
30
Pno.
38
Fl.
Ob.
38
Pno.

S3 Data. Analysis of conserved Q38-95 protective epitopes. Analysis was conducted using UniProt (https://www.uniprot.org, accessed on Feb 4, 2024) tools.

Q38-95:
MACATLKRTHDWDPLHSPNGRSPKPSPFGEVPPKSSPLESGSPSATPPASPTGLSPGGLLSPVRRDQPLFTFRQVGLICERMMKERESQIRDEYDHVLSAKLAEQYDTFVKFTYDQIQKRFEGATPSYLSgggsHKPFGSPSSPSSSAIAAAAAAAKRPSPFAEAVCPKQLTFNTGSRPDSPPSMVLFTFKQALREQYDAVLTNKLAEQYDAAAPSYLSgggsRVQKGTVLCECPWNQHLVGDTCISDCVDKKCHE

Consensus sequences: Q38 (SAKLAEQYDTFVKFTYDQIQKRFEGATPSYLS)
BM95 (RVQKGTVLCECPWNQHLVGDTCISDCVDKKCHE)

(A) Analysis of Q38
(B) Analysis of Subolesin
(C) Analysis of BM86/BM95

(A) Peptide search (https://www.uniprot.org/peptide-search) for Q38 consensus sequences SAKLAEQYDTFVKFTYDQIQKRFEGATPSYLS

CLUSTAL O(1.2.4) multiple sequence (KLAEQYD and FVKFTYDQI) alignment for tick spp., mosquitoes *Aedes* spp. and pathogen *Vibrio* spp.
Results: 85.7% identity.

```
tr|A0A0H3ZJX9|A0A0H3ZJX9_9VIBR      MGKIVAIGNEKGGVGKTTTVVNLAYYFSHVRSKKVLVVDMDPQCNLTDKYFDQND-----      55
tr|A0A0H3ZMM6|A0A0H3ZMM6_VIBSP      MGKIVAIGNEKGGVGKTTTVVNLAYYFSHVRSKKVLVVDMDPQCNLTDKYFDQND-----      55
tr|A0A2N7JR33|A0A2N7JR33_VIBSP      MGKIVAIGNEKGGVGKTTSVVNLAYYFSHVRNKKVLVVDMDPQCNLTDKYFDQND-----      55
tr|A0A2N7MJ04|A0A2N7MJ04_VIBSP      MGKIVAIGNEKGGVGKTTSVVNLAYYFSHVRNKKVLVVDMDPQCNLTDKYFDQND-----      55
tr|A0A1Q3F078|A0A1Q3F078_CULTA      -----------------------------------------MACATLKRSLDWESL---N      16
tr|A0A1Q3F0C4|A0A1Q3F0C4_CULTA      ------------------------------------------------------------      0
tr|A0A182X279|A0A182X279_ANOQN      -----------------------------------------MACATLKRSLDWESL---N      16
tr|A0A182I6D9|A0A182I6D9_ANOAR      -----------------------------------------MACATLKRSLDWESL---N      16
tr|A0A182L3Z9|A0A182L3Z9_ANOCL      -----------------------------------------MACATLKRSLDWESL---N      16
tr|A0A182U5E8|A0A182U5E8_9DIPT      ------------------------------------------------------------      0
tr|A0A182VLD0|A0A182VLD0_ANOME      -----------------------------------------MACATLKRSLDWESL---N      16
tr|Q7QIE8|Q7QIE8_ANOGA              -----------------------------------------MACATLKRSLDWESL---N      16
tr|A0A182PIV6|A0A182PIV6_9DIPT      -----------------------------------------MACATLKRSLDWESL---N      16
tr|A0A2M4CTL6|A0A2M4CTL6_ANODA      -----------------------------------------MACATLKRSLDWESL---N      16
tr|A0A2M4AHW6|A0A2M4AHW6_9DIPT      -----------------------------------------MACATLKRSLDWESL---N      16
tr|A0A1L3N235|A0A1L3N235_9DIPT      -----------------------------------------MACATLKRSLDWESL---N      16
tr|A0A1L3N228|A0A1L3N228_9DIPT      -----------------------------------------MACATLKRSLDWESL---N      16
tr|A0A1L3N225|A0A1L3N225_ANOFN      -----------------------------------------MVCATLKRSLDWESL---N      16
tr|A0A182Y884|A0A182Y884_ANOST      -----------------------------------------MACATLKRSLDWESL---N      16
tr|A0A182WI31|A0A182WI31_9DIPT      -----------------------------------------MACATLKRSLDWESL---N      16
tr|A0A182R6Q1|A0A182R6Q1_ANOFN      -----------------------------------------MACATLKRSLDWESL---N      16
tr|A0A182QLK0|A0A182QLK0_9DIPT      -----------------------------------------MACATLKRSLDWESL---N      16
tr|A0A182N7F5|A0A182N7F5_9DIPT      -----------------------------------------MACATLKRSLDWESL---N      16
tr|A0A182FFJ8|A0A182FFJ8_ANOAL      -----------------------------------------MACATLKRSLDWESL---N      16
tr|A0A182MCQ5|A0A182MCQ5_9DIPT      ------------------------------------------------------------      0
tr|A0A182SNZ4|A0A182SNZ4_9DIPT      ------------------------------------------------------------      0
tr|A0A4Y0BFV2|A0A4Y0BFV2_ANOFN      ------------------------------------------------------------      0
tr|A0A182J5N6|A0A182J5N6_9DIPT      -----------------------------------------MACATLKRSLDWESL---N      16
tr|A0A084WMV7|A0A084WMV7_ANOSI      ------------------------------------------------------------      0
tr|A0A2M3Z691|A0A2M3Z691_9DIPT      -----------------------------------------MACATLKRSLDWESL---N      16
tr|A0A1L3N222|A0A1L3N222_AEDAE      -----------------------------------------MACATLKRSLDWESL---N      16
tr|Q16MV0|Q16MV0_AEDAE              -----------------------------------------MACATLKRSLDWESL---N      16
tr|A0A023EJ88|A0A023EJ88_AEDAL      -----------------------------------------MACATLKRSLDWESL---N      16
tr|A0A182H6N2|A0A182H6N2_AEDAL      -----------------------------------------MACATLKRSLDWESL---N      16
tr|A0A131YRI7|A0A131YRI7_RHIAP      -----------------------------------------MACATLKRSNSCDPLSPNE      19
tr|R9R3J1|R9R3J1_AEDAL              -----------------------------------------MACATLKRTHDWDPLHSPN      19
tr|A0A0D5Y9E2|A0A0D5Y9E2_9ACAR      ------------------------------------------ACATLKRTHDWDPLHSPN      18
tr|A0A2R5LKD0|A0A2R5LKD0_9ACAR      -----------------------------------------MACATLKRTHDWDPLHSPN      19
tr|S4U9H1|S4U9H1_ORNSA              -----------------------------------------MACATLKRTHDWDPLHSPN      19
tr|S4UD40|S4UD40_ORNMO              -----------------------------------------MACATLKRTHDWDPLHSPN      19
tr|S4U8D6|S4U8D6_RHIMP              -----------------------------------------MACATLKRIHDWDPLHSPS      19
tr|S4UD31|S4UD31_RHIMP              -----------------------------------------MACATLKRTHDWDPLHSPS      19
tr|S4UD36|S4UD36_RHIMP              -----------------------------------------MACATLKRTHDWDPLHSQS      19
tr|S4U9H0|S4U9H0_RHIEV              -----------------------------------------MACATLKRTHDWDPLHSPS      19
tr|S4U8E0|S4U8E0_9ACAR              -----------------------------------------MACATLKRTHDWDPLHSPS      19
tr|Q1AER5|Q1AER5_RHIMP              -----------------------------------------MACATLKRTHDWDPLHSPS      19
tr|A0A7D5D0I3|A0A7D5D0I3_RHIAP      -----------------------------------------MACATLKRTHDWDPLHSPS      19
tr|S4UAH0|S4UAH0_RHIDE              -----------------------------------------MACATLKRTHDWDPLHSPS      19
tr|A0A224YH38|A0A224YH38_9ACAR      -----------------------------------------MACATLKRTHDWDPLHSPS      19
tr|A0A7D5D0F6|A0A7D5D0F6_RHIDE      -----------------------------------------MACATLKRTHDWDPLHSPS      19
tr|Q1AER4|Q1AER4_RHIAP              -----------------------------------------MACATLKRTHDWDPLHSPS      19
```

```
tr|Q1AER3|Q1AER3_RHISA                  -----------------------------------------MACATLKRTHDWDPLHSPS          19
tr|A0A097BQ10|A0A097BQ10_RHIHE          -----------------------------------------MACATLKRTHDWDPLHSPS          19
tr|A0A0H3WJP2|A0A0H3WJP2_RHIHE          -----------------------------------------MACATLKRTHDWDPLHSPS          19
tr|A0A023FG32|A0A023FG32_AMBCJ          -----------------------------------------MACATLKRTHDWDPLHSPN          19
tr|A0A1E1XVC7|A0A1E1XVC7_AMBSC          -----------------------------------------MACATLKRTHDWDPLHSPN          19
tr|A0A1E1X1X1|A0A1E1X1X1_9ACAR          -----------------------------------------MACATLKRTHDWDPLHSPN          19
tr|A0A023GFE4|A0A023GFE4_AMBTT          -----------------------------------------MACATLKRTHDWDPLHSPN          19
tr|S4UF12|S4UF12_AMBMU                  -----------------------------------------MACATLKRTHDWDPLHSPN          19
tr|A0A7D4XVE8|A0A7D4XVE8_AMBVA          -----------------------------------------MACATLKRTHDWDPLHSPN          19
tr|S4U8D4|S4U8D4_AMBVA                  -----------------------------------------MACATLKRTHDWDPLHSPN          19
tr|S4UAG1|S4UAG1_AMBCJ                  -----------------------------------------MACATLKRTHDWDPLHSPN          19
tr|S4U8D1|S4U8D1_AMBAM                  -----------------------------------------MACATLKRTHDWDPLHSPN          19
tr|A0A0C9RT91|A0A0C9RT91_AMBAM          -----------------------------------------MACATLKRTHDWDPLHSPN          19
tr|Q1AER9|Q1AER9_AMBAM                  -----------------------------------------MACATLKRTHDWDPLHSPN          19
tr|S4U9F0|S4U9F0_AMBAM                  -----------------------------------------MACATLKRTHDWDPLHSPN          19
tr|S4UD38|S4UD38_DERRT                  -----------------------------------------MACATLKRTHDWDPLHSPN          19
tr|A0A097BQ19|A0A097BQ19_9ACAR          -----------------------------------------MACATLKRTHDWDPLHSPN          19
tr|Q1AER2|Q1AER2_DERMR                  -----------------------------------------MACATLKRTHDWDPLHSPN          19
tr|A0A0U2ID78|A0A0U2ID78_9ACAR          -----------------------------------------MACATLKRTHDWDPLHSPN          19
tr|S4U8E3|S4U8E3_HYARU                  -----------------------------------------MACATLKRTHDWDPLHSPN          19
tr|A0A0A1C3I5|A0A0A1C3I5_HYAAA          -----------------------------------------MACATLKRTHDWDPLHSPN          19
tr|A0A131XM91|A0A131XM91_9ACAR          -----------------------------------------MACATLKRTHDWDPLHSPN          19
tr|S4UAH3|S4UAH3_9ACAR                  -----------------------------------------MACATLKRTHDWDPLHSPN          19
tr|Q1AER0|Q1AER0_9ACAR                  -----------------------------------------MACATLKRTHDWDPLHSPN          19
tr|S4UF21|S4UF21_9ACAR                  -----------------------------------------MACATLKRTHDWDPLHSPN          19
tr|A0A077B3N4|A0A077B3N4_HAEFA          -----------------------------------------MACATLKRTHDWDPLHSPN          19
tr|Q1AEQ9|Q1AEQ9_9ACAR                  -----------------------------------------MACATLKRTHDWDPLHSPN          19
tr|R9R416|R9R416_AEDAL                  -----------------------------------------MACATLKRTHDWDPLHSPN          19
tr|S4UAF8|S4UAF8_IXOHE                  -----------------------------------------MACATLKRTHDWDPLHSPN          19
tr|S4UF09|S4UF09_IXOSC                  -----------------------------------------MACATLKRTHDWDPLHSPT          19
tr|S4U8C9|S4U8C9_IXOSC                  -----------------------------------------MACATLKRTHDWYPLHSPN          19
tr|S4U9E5|S4U9E5_IXOSC                  -----------------------------------------MACATLKRTHDWDPLHSPN          19
tr|Q1AES0|Q1AES0_IXORI                  -----------------------------------------MACATLKRTHDWDPLHSPN          19
tr|A0A0A7DZX9|A0A0A7DZX9_IXOPE          -----------------------------------------MACATLKRTHDWDPLHSPN          19
tr|A0A0A7E016|A0A0A7E016_IXOPE          -----------------------------------------MACATLKRTHDWDPLHSPN          19

tr|A0A0H3ZJX9|A0A0H3ZJX9_9VIBR          -ESK-------------AKPA----------------------------SITRKVGEANV          73
tr|A0A0H3ZMM6|A0A0H3ZMM6_VIBSP          -ESK-------------AKPA----------------------------SITRKVGEANV          73
tr|A0A2N7JR33|A0A2N7JR33_VIBSP          -ESK-------------AKPA----------------------------SITRKVGEANV          73
tr|A0A2N7MJ04|A0A2N7MJ04_VIBSP          -ESK-------------AKPA----------------------------SITRKVGEANV          73
tr|A0A1Q3F078|A0A1Q3F078_CULTA          QRPTKRRRCHPFGSPAGAAGPSSSSSSTN------ISNALNSSPSSSAVAARSAVMEPKP          70
tr|A0A1Q3F0C4|A0A1Q3F0C4_CULTA          -----------------------SSSSTN------ISNALNSSPSSSAVAARSAVMEPKP          31
tr|A0A182X279|A0A182X279_ANOQN          QRPTKRRRCHPFGSPSQTASASSSSS--------SPSGSSSTSVAAAAAAASMRVMEPKP          68
tr|A0A182I6D9|A0A182I6D9_ANOAR          QRPTKRRRCHPFGSPSQTASASSSSA--------SPSGSSSTSVAAAAAAASMRVMEPKP          68
tr|A0A182L3Z9|A0A182L3Z9_ANOCL          QRPTKRRRCHPFGSPSQTASASSSSA--------SPSGSSSTSVAAAAAAASMRVMEPKP          68
tr|A0A182U5E8|A0A182U5E8_9DIPT          ------------------------------------------------------------          0
tr|A0A182VLD0|A0A182VLD0_ANOME          QRPTKRRRCHPFGSPSQTASASSSSA--------SPSGSSSTSVAAAAAAASMRVMEPKP          68
tr|Q7QIE8|Q7QIE8_ANOGA                  QRPTKRRRCHPFGSPSQTASASSSSA--------SPSGSSSTSVAAAAAAASMRVMEPKP          68
tr|A0A182PIV6|A0A182PIV6_9DIPT          QRPTKRRRCHPFGSPSQNASASSSPS-----------ASSSTSVAAAAAAASMRVMEPKP          65
tr|A0A2M4CTL6|A0A2M4CTL6_ANODA          QRPTKRRRCHPFGSSPSSQGSSMSASSSPS-------SSSASSAAAVAAAASRRVMEPKP          69
tr|A0A2M4AHW6|A0A2M4AHW6_9DIPT          QRPTKRRRCHPFGSSPSSQGSSMSA---SSSPSS----TSASSAAAVAAAASRRVMEPKP          69
tr|A0A1L3N235|A0A1L3N235_9DIPT          QRPTKRRRCHPFGSPSQSSSASASSPSS-----S---ASSSTSVAAAAAAASMRVMEPKP          68
tr|A0A1L3N228|A0A1L3N228_9DIPT          QRPTKRRRCHPFGSPSQSSSASASSP-----SSS---ASSSTSVAAAAAAASMRVMEPKP          68
tr|A0A1L3N225|A0A1L3N225_ANOFN          QRPTKRRRCHPFGSPSQSPSASS-----SSSPSA---SSSTSVAAAAAAAASMRVMEPKP          68
tr|A0A182Y884|A0A182Y884_ANOST          QRPTKRRRCHPFGSPSQSSSASASSPS-----SS---ASSSTSVAAAAAAASMRVMEPKP          68
tr|A0A182WI31|A0A182WI31_9DIPT          QRPTKRRRCHPFGSPSQSPSASSSP-------SA---S-SATSVAAAAAAASMRVMEPKP          65
tr|A0A182R6Q1|A0A182R6Q1_ANOFN          QRPTKRRRCHPFGSPSQSPSASS-----SSSPSA---SSSTSVAAAAAAAASMRVMEPKP          68
tr|A0A182QLK0|A0A182QLK0_9DIPT          QRPTKRRRCHPFGSPSHSQSPQSAAAALSASPST---S--TTSAAAAAAAASMRVMEPKP          71
tr|A0A182N7F5|A0A182N7F5_9DIPT          QRPTKRRRCHPFGSPSHSQSPQSAAAASSSSPSA---AS-STSAAAAAAAASMRVMEPKP          72
tr|A0A182FFJ8|A0A182FFJ8_ANOAL          QRPTKRRRCHPFGSSPSSQGSSM---SASSSPSS---TA-ASSAAAVAAAASRRVMEPKP          69
tr|A0A182MCQ5|A0A182MCQ5_9DIPT          ------------------------------------------------------------          0
tr|A0A182SNZ4|A0A182SNZ4_9DIPT          ------------------------------------------------------------          0
tr|A0A4Y0BFV2|A0A4Y0BFV2_ANOFN          ------------------------------------------------------------          0
tr|A0A182J5N6|A0A182J5N6_9DIPT          QRPTKRRRCHPFGSPSQSQPSSASMAASASVMSPSSSSATSSAVSSAAAAAAMRVMEPKP          76
tr|A0A084WMV7|A0A084WMV7_ANOSI          ------------------------------------------------------------          0
tr|A0A2M3Z691|A0A2M3Z691_9DIPT          QRPTKRRRCHPFGSSPSSQGPSMSASSS-----PSST--SASSAAAVAAAASRRVMEPKP          69
tr|A0A1L3N222|A0A1L3N222_AEDAE          QRPTKRRRCHPFGSPSSSNAQNSPSSSA-------------SAMASSSSNSAMRVMEPKP          63
tr|Q16MV0|Q16MV0_AEDAE                  QRPTKRRRCHPFGSPSSSNAQNSPSSSA-------------SAMASSSSNSAMRVMEPKP          63
tr|A0A023EJ88|A0A023EJ88_AEDAL          QRPTKRRRCHPFGSPSSNAPNSPSSSAI-------------AAAAASSSNSAMRVMEPKP          63
tr|A0A182H6N2|A0A182H6N2_AEDAL          QRPTKRRRCHPFGSPSSNAPNSPSSSAI-------------AAAAASSSNSAMRVMEPKP          63
tr|A0A131YRI7|A0A131YRI7_RHIAP          NSAKRRRFCLSTSSATTPR-----------------------------HALQARPHQARS          50
tr|R9R3J1|R9R3J1_AEDAL                  GRSPK------------------------------------------------------P          25
tr|A0A0D5Y9E2|A0A0D5Y9E2_9ACAR          GRAPKRRRCMPLCVSPSTPPSR--------------------------------AHQICP          46
tr|A0A2R5LKD0|A0A2R5LKD0_9ACAR          GRAPKRRRCMPLCVSPSTPPSR--------------------------------AHQICP          47
tr|S4U9H1|S4U9H1_ORNSA                  GRSPKRRRCMPFSVSPSAPPSR--------------------------------AHQMSP          47
tr|S4UD40|S4UD40_ORNMO                  GRSPKRRRCMPFSVSPSAPPSR--------------------------------AHQMSP          47
tr|S4U8D6|S4U8D6_RHIMP                  GSSPKRRRCMPLS----PPPT-----------------------RA---------HQIDP          43
tr|S4UD31|S4UD31_RHIMP                  GSSPKRRRCMPLS----PPPT-----------------------RA---------HQIDP          43
tr|S4UD36|S4UD36_RHIMP                  GRSPKRRRCMPLS----PPPT-----------------------RA---------HQVDP          43
tr|S4U9H0|S4U9H0_RHIEV                  GRSPKRRRCMPLS----PPPT-----------------------RA---------HQIDP          43
tr|S4U8E0|S4U8E0_9ACAR                  GRSPKRRRCMPLS----PPPT-----------------------RA---------HQVDP          43
tr|Q1AER5|Q1AER5_RHIMP                  GRSPKRRRCMPLS----PPPT-----------------------RA---------HQIDP          43
tr|A0A7D5D0I3|A0A7D5D0I3_RHIAP          GRSPKRRRCMPLS----PPPT-----------------------RA---------HQIDP          43
tr|S4UAH0|S4UAH0_RHIDE                  GRSPKRRRCMPLS----PPPT-----------------------RA---------HQIDP          43
tr|A0A224YH38|A0A224YH38_9ACAR          GRSPKRRRCMPLS----PPPT-----------------------RA---------HQTDP          43
tr|A0A7D5D0F6|A0A7D5D0F6_RHIDE          GRSPKRRRCMPLS----PPPT-----------------------RA---------HQIDP          43
tr|Q1AER4|Q1AER4_RHIAP                  GRSPKRRRCMPLS----PPPT-----------------------RA---------HQIDP          43
tr|Q1AER3|Q1AER3_RHISA                  GRSPKRRRCMPLS----PPPT-----------------------RA---------HQIDP          43
tr|A0A097BQ10|A0A097BQ10_RHIHE          GRSPKRRRCMPLS----PPPT-----------------------RA---------HQIDP          43
tr|A0A0H3WJP2|A0A0H3WJP2_RHIHE          GRSPKRRRCMPLS----PPPT-----------------------RA---------HQIDP          43
tr|A0A023FG32|A0A023FG32_AMBCJ          GRSPKRRRCMPFSVSPPAPPT-----------------------RA---------HQINP          47
tr|A0A1E1XVC7|A0A1E1XVC7_AMBSC          GRSPKRRRCMPFSVSPPAPPT-----------------------RA---------HQINP          47
tr|A0A1E1X1X1|A0A1E1X1X1_9ACAR          GRSPKRRRCMPFSVSPPAPPT-----------------------RA---------HQINP          47
tr|A0A023GFE4|A0A023GFE4_AMBTT          GRSPKRRRCMPLSVSPAAPPT-----------------------RA---------HQINP          47
```

```
tr|S4UF12|S4UF12_AMBMU               GRSPKRRRCMPLSVSPAAPPT-----------------------RA---------HQINP          47
tr|A0A7D4XVE8|A0A7D4XVE8_AMBVA       GRSPKRRRCMPLSVSPPAPPT-----------------------RA---------HQINP          47
tr|S4U8D4|S4U8D4_AMBVA               GRSPKRRRCMPLSVSPPAPPT-----------------------RA---------HQINP          47
tr|S4UAG1|S4UAG1_AMBCJ               GRSAKRRRCMPFSVSPPAPPT-----------------------RA---------HQINP          47
tr|S4U8D1|S4U8D1_AMBAM               GRSPKRRRCMPFSVNPPAPPT-----------------------RA---------HQINP          47
tr|A0A0C9RT91|A0A0C9RT91_AMBAM       GRSPKRRRCMPFSVNPPAPPT-----------------------RA---------HQINP          47
tr|Q1AER9|Q1AER9_AMBAM               GRSPKRRRCMPFSVNPPAPPT-----------------------RA---------HQINP          47
tr|S4U9F0|S4U9F0_AMBAM               GRSPKRRRCMPFSVNPPAPPT-----------------------RA---------HQINP          47
tr|S4UD38|S4UD38_DERRT               GRSPKRRRCMPLSVSPPAPPT-----------------------RA---------HQINP          47
tr|A0A097BQ19|A0A097BQ19_9ACAR       GRSPKRRRCMPLSVSPPAPPT-----------------------RA---------HQINP          47
tr|Q1AER2|Q1AER2_DERMR               GRSPKRRRCMPLSVSPPAPPT-----------------------RA---------HQINP          47
tr|A0A0U2ID78|A0A0U2ID78_9ACAR       GRSPKRRRCMPL--SPPAPPT-----------------------RA---------HQMNP          45
tr|S4U8E3|S4U8E3_HYARU               GRSPKRRRCMPL--SPPAPPT-----------------------RP---------HQMNP          45
tr|A0A0A1C3I5|A0A0A1C3I5_HYAAA       GRSPKRRRCMPL--SPPAPPT-----------------------RA---------HQMNP          45
tr|A0A131XM91|A0A131XM91_9ACAR       GRSPKRRRCMPL--SPPAPPT-----------------------RA---------HQMNP          45
tr|S4UAH3|S4UAH3_9ACAR               GRSPKRRRCMPL--SPPAPPT-----------------------RA---------HQMNP          45
tr|Q1AER0|Q1AER0_9ACAR               GRSPKRRRCMPL--SPPAPPT-----------------------RA---------HQMNP          45
tr|S4UF21|S4UF21_9ACAR               GRSPKRRRCMPLSVTS-TPPT-----------------------RA---------HQINP          46
tr|A0A077B3N4|A0A077B3N4_HAEFA       GRSPKRRRCMPLSVTP-TPPT-----------------------RA---------HQINP          46
tr|Q1AEQ9|Q1AEQ9_9ACAR               RRSPKRRRCMPLSVTP-TPPT-----------------------RA---------HQINP          46
tr|R9R416|R9R416_AEDAL               GRSPKRRRCMPLGSPSSNAPNSPSSSAIA-----------AAAAAAHQINSAMRVMEPKP          68
tr|S4UAF8|S4UAF8_IXOHE               GRSPKRRRCMPLSVTQAATPPT----------------------RAH---------QINP          48
tr|S4UF09|S4UF09_IXOSC               GRSPKRRRCMPLSVTQAATPPT----------------------RAH---------QINP          48
tr|S4U8C9|S4U8C9_IXOSC               GRSPKRRRCMPLSVTQAATPPT----------------------RAH---------QINP          48
tr|S4U9E5|S4U9E5_IXOSC               GRSPKRRRCMPLSVTQAATPPT----------------------RAH---------QINP          48
tr|Q1AES0|Q1AES0_IXORI               GRSPKRRRCMPLSVTQAATPPT----------------------RAH---------QINP          48
tr|A0A0A7DZX9|A0A0A7DZX9_IXOPE       GRSPKRRRCMPLSVTQAATPPT----------------------RAH---------QINP          48
tr|A0A0A7E016|A0A0A7E016_IXOPE       GRSPKRRRCMPLSVTQAATPPT----------------------RAH---------QINP          48

tr|A0A0H3ZJX9|A0A0H3ZJX9_9VIBR       ISFFDEEFYGEPVELNSNLHIFGATFNISSLNNCTNDEIGFFAQN----INKLAEQYDYV          129
tr|A0A0H3ZMM6|A0A0H3ZMM6_VIBSP       ISFFDEEFYGEPVELNSNLHIFGATFNISSLNNCTNDEIGFFAQN----INKLAEQYDYV          129
tr|A0A2N7JR33|A0A2N7JR33_VIBSP       ISFFDEEFYGKPVELNSNLHIFGATFNISSLNNCTNDEIGFFADN----INKLAEQYDYV          129
tr|A0A2N7MJ04|A0A2N7MJ04_VIBSP       ISFFDEEFYGKPVELNSNLHIFGATFNISSLNNCTNDEIGFFADN----INKLAEQYDYV          129
tr|A0A1Q3F078|A0A1Q3F078_CULTA       SPFAD------A--------------------VCPKLTPEKMAQNITEEIKRLHRRKQLT          104
tr|A0A1Q3F0C4|A0A1Q3F0C4_CULTA       SPFAD------A--------------------VCPKLTPEKMAQNITEEIKRLHRRKQLT          65
tr|A0A182X279|A0A182X279_ANOQN       SPFAE------A--------------------TCSKLTPEKMAQNITEEIKRLHRRKQLT          102
tr|A0A182I6D9|A0A182I6D9_ANOAR       SPFAE------A--------------------TCSKLTPEKMAQNITEEIKRLHRRKQLT          102
tr|A0A182L3Z9|A0A182L3Z9_ANOCL       SPFAE------A--------------------TCSKLTPEKMAQNITEEIKRLHRRKQLT          102
tr|A0A182U5E8|A0A182U5E8_9DIPT       -----------------------------------------MAQNITEEIKRLHRRKQLT          19
tr|A0A182VLD0|A0A182VLD0_ANOME       SPFAE------A--------------------TCSKLTPEKMAQNITEEIKRLHRRKQLT          102
tr|Q7QIE8|Q7QIE8_ANOGA               SPFAE------A--------------------TCSKLTPEKMAQNITEEIKRLHRRKQLT          102
tr|A0A182PIV6|A0A182PIV6_9DIPT       SPFAE------A--------------------TCSKLTPEKMAQNITEEIKRLHRRKQLT          99
tr|A0A2M4CTL6|A0A2M4CTL6_ANODA       SPFAE------A--------------------TCSKLTPEKMAQNITEEIKRLHRRKQLT          103
tr|A0A2M4AHW6|A0A2M4AHW6_9DIPT       SPFAE------A--------------------TCSKLTPEKMAQNITEEIKRLHRRKQLT          103
tr|A0A1L3N235|A0A1L3N235_9DIPT       SPFAE------A--------------------TCSKLTPEKMAQNITEEIKRLHRRKQLT          102
tr|A0A1L3N228|A0A1L3N228_9DIPT       SPFAE------A--------------------TCSKLTPEKMAQNITEEIKRLHRRKQLT          102
tr|A0A1L3N225|A0A1L3N225_ANOFN       SPFAE------A--------------------TCSKLTPEKMAQNITEEIKRLHRRKQLT          102
tr|A0A182Y884|A0A182Y884_ANOST       SPFAE------A--------------------TCSKLTPEKMAQNITEEIKRLHRRKQLT          102
tr|A0A182WI31|A0A182WI31_9DIPT       SPFAE------A--------------------TCSKLTPEKMAQNITEEIKRLHRRKQLT          99
tr|A0A182R6Q1|A0A182R6Q1_ANOFN       SPFAE------A--------------------TCSKLTPEKMAQNITEEIKRLHRRKQLT          102
tr|A0A182QLK0|A0A182QLK0_9DIPT       SPFAE------A--------------------TCSKLTPEKMAQNITEEIKRLHRRKQLT          105
tr|A0A182N7F5|A0A182N7F5_9DIPT       SPFAE------A--------------------TCSKLTPEKMAQNITEEIKRLHRRKQLT          106
tr|A0A182FFJ8|A0A182FFJ8_ANOAL       SPFAE------A--------------------TCSKLTPEKMAQNITEEIKRLHRRKQLT          103
tr|A0A182MCQ5|A0A182MCQ5_9DIPT       -----------------------------------------MAQNITEEIKRLHRRKQLT          19
tr|A0A182SNZ4|A0A182SNZ4_9DIPT       -----------------------------------------MAQNITEEIKRLHRRKQLT          19
tr|A0A4Y0BFV2|A0A4Y0BFV2_ANOFN       -----------------------------------------MAQNITEEIKRLHRRKQLT          19
tr|A0A182J5N6|A0A182J5N6_9DIPT       SPFAE------A--------------------TCSKLTPEKMAQNITEEIKRLHRRKQLN          110
tr|A0A084WMV7|A0A084WMV7_ANOSI       -----------------------------------------MAQNITEEIKRLHRRKQLN          19
tr|A0A2M3Z691|A0A2M3Z691_9DIPT       SPFAE------A--------------------TCSKLTPEKMAQNITEEIKRLHRRKQLT          103
tr|A0A1L3N222|A0A1L3N222_AEDAE       SPFAE------A--------------------VCPKLTPGKFNCCLHREIKRLHRRKQLT          97
tr|Q16MV0|Q16MV0_AEDAE               SPFAE------A--------------------VCPKLTPGKFNCCLHREIKRLHRRKQLT          97
tr|A0A023EJ88|A0A023EJ88_AEDAL       SPFAE------A--------------------VCPKLTPEKMAQNITEEIKRLHRRKQLT          97
tr|A0A182H6N2|A0A182H6N2_AEDAL       SPFAE------A--------------------VCPKLTPEKMAQNITEEIKRLHRRKQLT          97
tr|A0A131YRI7|A0A131YRI7_RHIAP       SPFVD---------------------------ATPKMTSEEIEANVHDEMLRLQRRRQLF          83
tr|R9R3J1|R9R3J1_AEDAL               SPFGE---------------------------VPPK------------------------          34
tr|A0A0D5Y9E2|A0A0D5Y9E2_9ACAR       SPFVE---------------------------VPPKLSSEEIAANIREEMKRLQRRKQLC          79
tr|A0A2R5LKD0|A0A2R5LKD0_9ACAR       SPFVE---------------------------VPPKLSSEEIAANIREEMKRLQRRKQLC          80
tr|S4U9H1|S4U9H1_ORNSA               SPFVE---------------------------VPPKLSSEEIAANIREEMRRLQRRKQLY          80
tr|S4UD40|S4UD40_ORNMO               SPFVE---------------------------VPPKLSSEEIAANIREEMRRLQRRKQLY          80
tr|S4U8D6|S4U8D6_RHIMP               SPFGD---------------------------VPPKLTSEEIAANIREEMRRLQRRKQLC          76
tr|S4UD31|S4UD31_RHIMP               SPFGD---------------------------VPPKLTSEEIAANIREEMRRLQRRKQLC          76
tr|S4UD36|S4UD36_RHIMP               SPFGD---------------------------VPPKLTSEEIAANIREEMRRLQRRKQLC          76
tr|S4U9H0|S4U9H0_RHIEV               SPFGD---------------------------VPPKLTSEEIAANIREEMRRLQRRKQLC          76
tr|S4U8E0|S4U8E0_9ACAR               SPFGD---------------------------VPPKLTSEEIAANIREEMRRLQRRKQLC          76
tr|Q1AER5|Q1AER5_RHIMP               SPFGD---------------------------VPPKLTSEEIAANIREEMRRLQRRKQLC          76
tr|A0A7D5D0I3|A0A7D5D0I3_RHIAP       SPFGD---------------------------VPPKLTSEEIAASIREEMRRLQRRKQLC          76
tr|S4UAH0|S4UAH0_RHIDE               SPFGD---------------------------VPPKLTSEEIAANIREEMRRLQRRKQLC          76
tr|A0A224YH38|A0A224YH38_9ACAR       SPFGD---------------------------VPPKLTSEEIAANIREEMRRLQRRKQLC          76
tr|A0A7D5D0F6|A0A7D5D0F6_RHIDE       SPFGD---------------------------VPPKLTSEEIAANIREEMRRLQRRKQLC          76
tr|Q1AER4|Q1AER4_RHIAP               SPFGD---------------------------VPPKLTSEEIAANIREEMRRLQRRKQLC          76
tr|Q1AER3|Q1AER3_RHISA               SPFGD---------------------------VPPKLTSEEIAANIREEMRRLQRRKQLC          76
tr|A0A097BQ10|A0A097BQ10_RHIHE       SPFGD---------------------------VPPKLTSEEIAANIREEMRRLQRRKQLC          76
tr|A0A0H3WJP2|A0A0H3WJP2_RHIHE       SPFGD---------------------------VPPKLTSEEIAANIREEMRRLQRRKQLC          76
tr|A0A023FG32|A0A023FG32_AMBCJ       SPFGD---------------------------VPPKLTSEEIAANIREEMRRLQRRKQLC          80
tr|A0A1E1XVC7|A0A1E1XVC7_AMBSC       SPFGD---------------------------VPPKLTSEEIAANIREEMRRLQRRKQLC          80
tr|A0A1E1X1X1|A0A1E1X1X1_9ACAR       SPFGD---------------------------VPPKLTSEEIAANIREEMRRLQRRKQLC          80
tr|A0A023GFE4|A0A023GFE4_AMBTT       SPFGD---------------------------VPPKLTSEEIAANIREEMRRLQRRKQLC          80
tr|S4UF12|S4UF12_AMBMU               SPFGD---------------------------VPPKLTSEEIAANIREEMRRLQRRKQLC          80
tr|A0A7D4XVE8|A0A7D4XVE8_AMBVA       SPFGD---------------------------VPPKLTSEEIAANIREEMRRLQRRKQLC          80
tr|S4U8D4|S4U8D4_AMBVA               SPFGD---------------------------VPPKLTSEEIAANIREEMRRLQRRKQLC          80
tr|S4UAG1|S4UAG1_AMBCJ               SPFGD---------------------------VPPKLTSEEIAANIREEMRRLQRRKQLC          80
tr|S4U8D1|S4U8D1_AMBAM               SPFGD---------------------------VPPKLTSEEIAANIREEMRRLQRRKQLC          80
tr|A0A0C9RT91|A0A0C9RT91_AMBAM       SPFGD---------------------------VPPKLTSEEIAANIREEMRRLQRRKQLC          80
tr|Q1AER9|Q1AER9_AMBAM               SPFGD---------------------------VPPKLTSEEIAANIREEMRRLQRRKQLC          80
```

```
tr|S4U9F0|S4U9F0_AMBAM              SPFGD---------------------------VPPKLTSEEIAANIREEMRRLQRPKQLC        80
tr|S4UD38|S4UD38_DERRT              SPFGD---------------------------VPPKLTSEEIAANIREEMRRLQRRKQLC        80
tr|A0A097BQ19|A0A097BQ19_9ACAR      SPFGD---------------------------VPPKLTSEEIAANIREEMRRLQRRKQLC        80
tr|Q1AER2|Q1AER2_DERMR              SPFGD---------------------------VPPKLTSEEIAANIREEMRRLQRRKQLC        80
tr|A0A0U2ID78|A0A0U2ID78_9ACAR      SPFGE---------------------------VPPKMTSEEIAANIREEMRRLQRRKQLC        78
tr|S4U8E3|S4U8E3_HYARU              SPFGE---------------------------VPPKMTSEEIAANIREEMRRLQRRKQLC        78
tr|A0A0A1C3I5|A0A0A1C3I5_HYAAA      SPFGE---------------------------VPPKMTSEEIAANIREEMRRLQRRKQLC        78
tr|A0A131XM91|A0A131XM91_9ACAR      SPFGE---------------------------VPPKMTSEEIAANIREEMRRLQRRKQLC        78
tr|S4UAH3|S4UAH3_9ACAR              SPFGE---------------------------VPPKMTSEEIAANIREEMRRLQRRKQLC        78
tr|Q1AER0|Q1AER0_9ACAR              SPFGE---------------------------VPPKMTSEEIAANIREEMRRLQRRKQLC        78
tr|S4UF21|S4UF21_9ACAR              SPFGD---------------------------VPPKLTSEEIAANIREEMRRLQRRKQLC        79
tr|A0A077B3N4|A0A077B3N4_HAEFA      SPFGD---------------------------VPPKLTSEEIAANIREEMRRLQRRKQLC        79
tr|Q1AEQ9|Q1AEQ9_9ACAR              SPFGD---------------------------VPPKLTSEEIAANIREEMRRLQRRKQLC        79
tr|R9R416|R9R416_AEDAL              SPFAE------A--------------------VCPKLTSEEIAANIREEMRRLQRRKQLT        102
tr|S4UAF8|S4UAF8_IXOHE              SPFGE---------------------------VPPKLTSEEIAANIREEMRRLQRRKQLC        81
tr|S4UF09|S4UF09_IXOSC              SPFGE---------------------------VPPKLTSEEIAANIREEMRRLQRRKQLC        81
tr|S4U8C9|S4U8C9_IXOSC              SPFGE---------------------------VPPKLTSEEIAANIREEMRRLQRRKQLC        81
tr|S4U9E5|S4U9E5_IXOSC              SPFGE---------------------------VPPKLTSEEIAANIREEMRRLQRRKQLC        81
tr|Q1AES0|Q1AES0_IXORI              SPFGE---------------------------VPPKLTSEEIAANIREEMRRLQRRKQLC        81
tr|A0A0A7DZX9|A0A0A7DZX9_IXOPE      SPFGE---------------------------VPPKLTSEEIAANIREEMRRLQRRKQLC        81
tr|A0A0A7E016|A0A0A7E016_IXOPE      SPFGE---------------------------VPPKLTSEEIAANIREEMRRLQRRKQLC        81

tr|A0A0H3ZJX9|A0A0H3ZJX9_9VIBR      FIDTAPSV---------GNLQY---SALIACDGLLIPTTAEEDSFQGVSKILKSVARIKS        177
tr|A0A0H3ZMM6|A0A0H3ZMM6_VIBSP      FIDTAPSV---------GNLQY---SALIACDGLLIPTTAEEDSFQGVSKILKSVARIKS        177
tr|A0A2N7JR33|A0A2N7JR33_VIBSP      FIDTAPSV---------GNLQY---SALIACDGLLIPTTAEEDSFKGVSKIFKSVARIKN        177
tr|A0A2N7MJ04|A0A2N7MJ04_VIBSP      FIDTAPSV---------GNLQY---SALIACDGLLIPTTAEEDSFQGVSKILKSVARIKN        177
tr|A0A1Q3F078|A0A1Q3F078_CULTA      FNHSAAERMQDS-------------ESSGSEMGPDSPRRPDSPP--S---MVKNPEK--A        144
tr|A0A1Q3F0C4|A0A1Q3F0C4_CULTA      FNHSAAERMQDS-------------ESSGSEMGPDSPRRPDSPP--S---MVKNPEK--A        105
tr|A0A182X279|A0A182X279_ANOQN      FNSHNFERMQDS-------------ESSGSEMGPDSPRRPDSPP--S---MVKNPEK--A        142
tr|A0A182I6D9|A0A182I6D9_ANOAR      FNSHNFERMQDS-------------ESSGSEMGPDSPRRPDSPP--S---MVKNPEK--A        142
tr|A0A182L3Z9|A0A182L3Z9_ANOCL      FNSHNFERMQDS-------------ESSGSEMGPDSPRRPDSPP--S---MVKNPEK--A        142
tr|A0A182U5E8|A0A182U5E8_9DIPT      FNSHNFERMQDS-------------ESSGSEMGPDSPRRPDSPP--S---MVKNPEK--A        59
tr|A0A182VLD0|A0A182VLD0_ANOME      FNSHNFERMQDS-------------ESSGSEMGPDSPRRPDSPP--S---MVKNPEK--A        142
tr|Q7QIE8|Q7QIE8_ANOGA              FNSHNFERMQDS-------------ESSGSEMGPDSPRRPDSPP--S---MVKNPEK--A        142
tr|A0A182PIV6|A0A182PIV6_9DIPT      FNSHNVERMQDS-------------ESSGSEMGPDSPRRPDSPP--S---MVKNPEK--A        139
tr|A0A2M4CTL6|A0A2M4CTL6_ANODA      FNSHNVERMQDS-------------ESSGSEMGPDSPRRPDSPP--S---MVKNPEK--A        143
tr|A0A2M4AHW6|A0A2M4AHW6_9DIPT      FNSHNVERMQDS-------------ESSGSEMGPDSPRRPDSPP--S---MVKNPEK--A        143
tr|A0A1L3N235|A0A1L3N235_9DIPT      FNSHNVERMQDS-------------ESSGSEMGPDSPRRPDSPP--S---MVKNPEK--A        142
tr|A0A1L3N228|A0A1L3N228_9DIPT      FNSHNVERMQDS-------------ESSGSEMGPDSPRRPDSPP--S---MVKNPEK--A        142
tr|A0A1L3N225|A0A1L3N225_ANOFN      FNSHNVERMQDS-------------ESSGSEMGPDSPRRPDSPP--S---MVKNPEK--A        142
tr|A0A182Y884|A0A182Y884_ANOST      FNSHNVERMQDS-------------ESSGSEMGPDSPRRPDSPP--S---MVKNPEK--A        142
tr|A0A182WI31|A0A182WI31_9DIPT      FNSHNVERMQDS-------------ESSGSEMGPDSPRRPDSPP--S---MVKNPEK--A        139
tr|A0A182R6Q1|A0A182R6Q1_ANOFN      FNSHNVERMQDS-------------ESSGSEMGPDSPRRPDSPP--S---MVKNPEK--A        142
tr|A0A182QLK0|A0A182QLK0_9DIPT      FNSHNVERMQDS-------------ESSGSEMGPDSPRRPDSPP--S---MVKNPEK--A        145
tr|A0A182N7F5|A0A182N7F5_9DIPT      FNSHNVERMQDS-------------ESSGSEMGPDSPRRPDSPP--S---MVKNPEK--A        146
tr|A0A182FFJ8|A0A182FFJ8_ANOAL      FNSHNVERMQDS-------------ESSGSEMGPDSPRRPDSPP--S---MVKNPEK--A        143
tr|A0A182MCQ5|A0A182MCQ5_9DIPT      FNSHNVERMQDS-------------ESSGSEMGPDSPRRPDSPP--S---MVKNPEK--A        59
tr|A0A182SNZ4|A0A182SNZ4_9DIPT      FNSHNVERMQDS-------------ESSGSEMGPDSPRRPDSPP--S---MVKNPEK--A        59
tr|A0A4Y0BFV2|A0A4Y0BFV2_ANOFN      FNSHNVERMQDS-------------ESSGSEMGPDSPRRPDSPP--S---MVKNPEK--A        59
tr|A0A182J5N6|A0A182J5N6_9DIPT      FSSHTVERMQDS-------------ESSGSEMGPDSPRRPDSPP--S---MVKHPEK--A        150
tr|A0A084WMV7|A0A084WMV7_ANOSI      FNSHTVERMQDS-------------ESSGSEMGPDSPRRPDSPP--S---MVKNPEK--A        59
tr|A0A2M3Z691|A0A2M3Z691_9DIPT      FNSHTVERMQDS-------------ESSGSEMGPDSPRRPDSPP--S---MVKNPEK--A        143
tr|A0A1L3N222|A0A1L3N222_AEDAE      FNTGNMERMQDS-------------ESSGSEMGPDSPRRPDSPP--S---MVKHPEK--A        137
tr|Q16MV0|Q16MV0_AEDAE              FNTGNMERMQDS-------------ESSGSEMGPDSPRRPDSPP--S---MVKHPEK--A        137
tr|A0A023EJ88|A0A023EJ88_AEDAL      FNTGNMERMQDS-------------ESSGSEMGPDSPRRPDSPP--S---MVKHPEK--A        137
tr|A0A182H6N2|A0A182H6N2_AEDAL      FNTGNMERMQDS-------------ESSGSEMGPDSPRRPDSPP--S---MVKHPEK--A        137
tr|A0A131YRI7|A0A131YRI7_RHIAP      FQQGSPDYAA-------------------------SPAVLDTLP--P---QGAKADQ--P        111
tr|R9R3J1|R9R3J1_AEDAL              -SSPLESGSPSATPPA--------SPTGLSPGGLLSPVRRDQP-----------------        68
tr|A0A0D5Y9E2|A0A0D5Y9E2_9ACAR      FPTLDSSPQS-----------ADSLP-SSPTGGLLSPVRRDQP-----------------        110
tr|A0A2R5LKD0|A0A2R5LKD0_9ACAR      FPTLDSSPQS-----------ADSLP-SSPTGGLLSPVRRDQP-----------------        111
tr|S4U9H1|S4U9H1_ORNSA              FPSLGSPPQG-----------SSSSSSTDSPTGLLSPVRRDQP-----------------        112
tr|S4UD40|S4UD40_ORNMO              FPSLDSPPQG-----------TSG-SSADSPTGLLSPVRRDQP-----------------        111
tr|S4U8D6|S4U8D6_RHIMP              FQGRDP------------------ESQ--HTSGLSSPVHRDQP-----------------        99
tr|S4UD31|S4UD31_RHIMP              FQGRDP------------------ESQ--HTSGLSSPVHRDQP-----------------        99
tr|S4UD36|S4UD36_RHIMP              FQGADP------------------ESQ--HTSGLSSPVHRDQP-----------------        99
tr|S4U9H0|S4U9H0_RHIEV              FQGADP------------------ESQ--HTSGLSSPVRRDQP-----------------        99
tr|S4U8E0|S4U8E0_9ACAR              FQGADP------------------ESQ--HTSGLSSPVHRDQP-----------------        99
tr|Q1AER5|Q1AER5_RHIMP              FQGRDP------------------ESQ--HTSGLSSPVHRDQP-----------------        99
tr|A0A7D5D0I3|A0A7D5D0I3_RHIAP      FQGADP------------------ESQ--HTSGLSSPVHRDQP-----------------        99
tr|S4UAH0|S4UAH0_RHIDE              FQGADP------------------ESQ--HTSGLSSPVHRDQP-----------------        99
tr|A0A224YH38|A0A224YH38_9ACAR      FQGTDP------------------ESQ--HTSGLSSPVRRDQP-----------------        99
tr|A0A7D5D0F6|A0A7D5D0F6_RHIDE      FQGTDA------------------ESQ--HTSGLSSPVRRDQP-----------------        99
tr|Q1AER4|Q1AER4_RHIAP              FQGTDA------------------ESQ--HTSGLSSPVRRDQP-----------------        99
tr|Q1AER3|Q1AER3_RHISA              FQGTDP------------------ECQ--PTSGLSSPVRRDQP-----------------        99
tr|A0A097BQ10|A0A097BQ10_RHIHE      FQATDP------------------ECQ--QTSGLSSPVRRDQP-----------------        99
tr|A0A0H3WJP2|A0A0H3WJP2_RHIHE      FQATDP------------------ECQ--QTSGLSSPVRRDQP-----------------        99
tr|A0A023FG32|A0A023FG32_AMBCJ      FQGA---ESPPE-----GCSPVGSPSQ--HTGGLLSPVRRDQP-----------------        113
tr|A0A1E1XVC7|A0A1E1XVC7_AMBSC      FQGA---ESPPE-----GCPPVDSPSQ--HTGGLLSPVRRDQP-----------------        113
tr|A0A1E1X1X1|A0A1E1X1X1_9ACAR      FQGGAECSSPPE-----GCSPVGSPSQ--HTGGLLSPVRRDQP-----------------        116
tr|A0A023GFE4|A0A023GFE4_AMBTT      FQG-AECNSPPE-----GCSPVGSPSQ--HTGGLLSPVRRDQP-----------------        115
tr|S4UF12|S4UF12_AMBMU              FQG-AECNSPPE-----GCSPVGSPSQ--HTGGLLSPVRRDQP-----------------        115
tr|A0A7D4XVE8|A0A7D4XVE8_AMBVA      FQG-AECSSPPE-----GCSPVGSPSQ--HTSGLLSPVRRDQP-----------------        115
tr|S4U8D4|S4U8D4_AMBVA              FQG-AECSSPPE-----GCSPVGSPSQ--HTSGLLSPVRRDQP-----------------        115
tr|S4UAG1|S4UAG1_AMBCJ              FQG-AECSSPPE-----GCSPVGSPSQ--HTGGLLSPVRRDQP-----------------        115
tr|S4U8D1|S4U8D1_AMBAM              FQG-AECSSPPE-----GCSPIGSPSQ--HTGGLLSPVRRDQP-----------------        115
tr|A0A0C9RT91|A0A0C9RT91_AMBAM      FQG-AECSSPPE-----GCSPIGSPSQ--HTGGLLSPVRRDQP-----------------        115
tr|Q1AER9|Q1AER9_AMBAM              FQG-AECSSPPE-----GCSPIGSPSQ--HTGGLLSPVRRDQP-----------------        115
tr|S4U9F0|S4U9F0_AMBAM              FQG-AECSSPPE-----GCSPIGSPSQ--HTGGLLSPVRRDQP-----------------        115
tr|S4UD38|S4UD38_DERRT              FQGADPG-------------------Q--PAGGLSSPVRRDQP-----------------        102
tr|A0A097BQ19|A0A097BQ19_9ACAR      FQGTDPE------------------SQ--PTSGLLSPVRRDQP-----------------        103
tr|Q1AER2|Q1AER2_DERMR              FQGTDPE------------------SQ--PTSGLLSPVRRDQP-----------------        103
tr|A0A0U2ID78|A0A0U2ID78_9ACAR      FQGTDPE------------------SQ--QTSGLLSPVRRDQP-----------------        101
tr|S4U8E3|S4U8E3_HYARU              FQGTDPE------------------SQ--QTSGLLSPVRRDQP-----------------        101
tr|A0A0A1C3I5|A0A0A1C3I5_HYAAA      FQGTDPE------------------SQ--QTSGLLSPVRRDQP-----------------        101
```

```
tr|A0A131XM91|A0A131XM91_9ACAR        FQGTDPE------------------SQ--QTSGLLSPVRRDQP-----------------          101
tr|S4UAH3|S4UAH3_9ACAR                FQGTDPE------------------SQ--QTSGLLSPVRRDQP-----------------          101
tr|Q1AER0|Q1AER0_9ACAR                FQRTDPE------------------SQ--QTSGLLSPVRRDQP-----------------          101
tr|S4UF21|S4UF21_9ACAR                FQGE---GASSG-----GDSPVGSPGCSS-SQGLLSPVRRDQP-----------------          113
tr|A0A077B3N4|A0A077B3N4_HAEFA        FQAGAEGGTSSG-----GDSPIGSPSSSSQAGGLLSPVRRDQP-----------------          117
tr|Q1AEQ9|Q1AEQ9_9ACAR                FQAGAEGSPVGG-----GDSPVGSPSSSSQAGGLLSPMRRDQP-----------------          117
tr|R9R416|R9R416_AEDAL                FSSPLESGSPSATPPAADCGPAS--PTGLSPGGLLSPVRRDQPP--S---MVKHPEK--A          153
tr|S4UAF8|S4UAF8_IXOHE                FSSPLETGSPSGTL----CGVS-------AECGLLSPMRRDQP-----------------          113
tr|S4UF09|S4UF09_IXOSC                FSSPLESGSPSATPPAADCGPAS--PTGLSPGGLLSPVRRDQP-----------------          122
tr|S4U8C9|S4U8C9_IXOSC                FSSPLESGSPSATPPAADCGPAS--PTGLSPGGLLSPVRRDQP-----------------          122
tr|S4U9E5|S4U9E5_IXOSC                FSSPLESGSPSVTPPAADCGPAS--PTGLSPGGLLSPVRRDQP-----------------          122
tr|Q1AES0|Q1AES0_IXORI                FSSPLESGSPSVTPPAAECGPAS--PTGLSPGGLLSPVRRDQP-----------------          122
tr|A0A0A7DZX9|A0A0A7DZX9_IXOPE        FSSPLESGSPSATPPAAECGPAS--PTGLSPGGLLSPVRRDQP-----------------          122
tr|A0A0A7E016|A0A0A7E016_IXOPE        FSSPLESGSPSATPPAAECGPAS--PTGLSPGGLLSPVRRDQP-----------------          122
                                                                        *   :

tr|A0A0H3ZJX9|A0A0H3ZJX9_9VIBR        TYGLDVSVLGM-YLNMVKKV----PTQLQDYFATKLSEDYGDLVFKTQVIHTTRVSEASA          232
tr|A0A0H3ZMM6|A0A0H3ZMM6_VIBSP        TYGLDVSVLGM-YLNMVKKV----PTQLQDYFATKLSEDYGDLVFKTQVIHTTRVSEASA          232
tr|A0A2N7JR33|A0A2N7JR33_VIBSP        TYGLDVSVLGM-YLNMVKKV----PTQLQDYFATKLSEDYGDLLFKTQVIHTTRVSEASA          232
tr|A0A2N7MJ04|A0A2N7MJ04_VIBSP        TYGLDVSVLGM-YLNMVKKV----PTQLQDYFATKLSEDYGDLVFKTQVIHTTRVSEASA          232
tr|A0A1Q3F078|A0A1Q3F078_CULTA        L--FTFKQVQMICERMLKEREDSLREQYDAVLTTKLAEQYDAFVKFTY------------          190
tr|A0A1Q3F0C4|A0A1Q3F0C4_CULTA        L--FTFKQVQMICERMLKEREDSLREQYDAVLTTKLAEQYDAFVKFTY------------          151
tr|A0A182X279|A0A182X279_ANOQN        L--FTFKQVQMICERMLKEREDALREQYDAVLTTKLAEQYDAFVKFTY------------          188
tr|A0A182I6D9|A0A182I6D9_ANOAR        L--FTFKQVQMICERMLKEREDALREQYDAVLTTKLAEQYDAFVKFTY------------          188
tr|A0A182L3Z9|A0A182L3Z9_ANOCL        L--FTFKQVQMICERMLKEREDALREQYDAVLTTKLAEQYDAFVKFTY------------          188
tr|A0A182U5E8|A0A182U5E8_9DIPT        L--FTFKQVQMICERMLKEREDALREQYDAVLTTKLAEQYDAFVKFTY------------          105
tr|A0A182VLD0|A0A182VLD0_ANOME        L--FTFKQVQMICERMLKEREDALREQYDAVLTTKLAEQYDAFVKFTY------------          188
tr|Q7QIE8|Q7QIE8_ANOGA                L--FTFKQVQMICERMLKEREDALREQYDAVLTTKLAEQYDAFVKFTY------------          188
tr|A0A182PIV6|A0A182PIV6_9DIPT        L--FTFKQVQMICERMLKEREDSLREQYDAVLTTKLAEQYDAFVKFTY------------          185
tr|A0A2M4CTL6|A0A2M4CTL6_ANODA        L--FTFKQVQMICERMLKEREDALREQYDAVLTTKLAEQYDAFVKFTY------------          189
tr|A0A2M4AHW6|A0A2M4AHW6_9DIPT        L--FTFKQVQMICERMLKEREDALREQYDAVLTTKLAEQYDAFVKFTY------------          189
tr|A0A1L3N235|A0A1L3N235_9DIPT        L--FTFKQVQMICERMLKEREDALREQYDAVLTTKLAEQYDAFVKFTY------------          188
tr|A0A1L3N228|A0A1L3N228_9DIPT        L--FTFKQVQMICERMLKEREDALREQYDAVLTTKLAEQYDAFVKFTY------------          188
tr|A0A1L3N225|A0A1L3N225_ANOFN        L--FTFKQVQMICERMLKEREDALREQYDAVLTTKLAEQYDAFVKFTY------------          188
tr|A0A182Y884|A0A182Y884_ANOST        L--FTFKQVQMICERMLKEREDALREQYDAVLTTKLAEQYDAFVKFTY------------          188
tr|A0A182WI31|A0A182WI31_9DIPT        L--FTFKQVQMICERMLKEREDALREQYDAVLTTKLAEQYDAFVKFTY------------          185
tr|A0A182R6Q1|A0A182R6Q1_ANOFN        L--FTFKQVQMICERMLKEREDALREQYDAVLTTKLAEQYDAFVKFTY------------          188
tr|A0A182QLK0|A0A182QLK0_9DIPT        L--FTFKQVQMICERMLKEREDALREQYDAVLTTKLAEQYDAFVKFTY------------          191
tr|A0A182N7F5|A0A182N7F5_9DIPT        L--FTFKQVQMICERMLKEREDALREQYDAVLTTKLAEQYDAFVKFTY------------          192
tr|A0A182FFJ8|A0A182FFJ8_ANOAL        L--FTFKQVQMICERMLKEREDALREQYDAVLTTKLAEQYDAFVKFTY------------          189
tr|A0A182MCQ5|A0A182MCQ5_9DIPT        L--FTFKQVQMICERMLKEREDALREQYDAVLTTKLAEQYDAFVKFTY------------          105
tr|A0A182SNZ4|A0A182SNZ4_9DIPT        L--FTFKQVQMICERMLKEREDALREQYDAVLTTKLAEQYDAFVKFTY------------          105
tr|A0A4Y0BFV2|A0A4Y0BFV2_ANOFN        L--FTFKQVQMICERMLKEREDALREQYDAVLTTKLAEQYDAFVKFTY------------          105
tr|A0A182J5N6|A0A182J5N6_9DIPT        L--FTFKQVQMICERMLKEREDALREQYDAVLTTKLAEQYDAFVKFTY------------          196
tr|A0A084WMV7|A0A084WMV7_ANOSI        L--FTFKQVQMICERMLKEREDALREQYDAVLTTKLAEQYDAFVKFTY------------          105
tr|A0A2M3Z691|A0A2M3Z691_9DIPT        L--FTFKQVQMICERMLKEREDALREQYDAVLTTKLAEQYDAFVKFTY------------          189
tr|A0A1L3N222|A0A1L3N222_AEDAE        L--FTFKQVQMICERMLKEREDALREQYDAVLTNKLAEQYDAFVKFTY------------          183
tr|Q16MV0|Q16MV0_AEDAE                L--FTFKQVQMICERMLKEREDALREQYDAVLTNKLAEQYDAFVKFTY------------          183
tr|A0A023EJ88|A0A023EJ88_AEDAL        L--FTFKQVQMICERMLKEREDALREQYDAVLTNKLAEQYDAFVKFTY------------          183
tr|A0A182H6N2|A0A182H6N2_AEDAL        L--FTFKQVQMICERMLKEREDALREQYDAVLTNKLAEQYDAFVKFTY------------          183
tr|A0A131YRI7|A0A131YRI7_RHIAP        V--FTFRQVGLIVERMVSEREKQLREVYDSVLSAKLAEQYDAFVKFTH------------          157
tr|R9R3J1|R9R3J1_AEDAL                L--FTFRQVGLICERMMKERESQIRDEYDHVLSAKLAEQYDTFVKFTY------------          114
tr|A0A0D5Y9E2|A0A0D5Y9E2_9ACAR        L--FTFRQVGLICERMMKERECQIREQYDQVLSTKLAEQYDTFVKFTY------------          156
tr|A0A2R5LKD0|A0A2R5LKD0_9ACAR        L--FTFRQVGLICERMMKERECQIREQYDQVLSTKLAEQYDTFVKFTY------------          157
tr|S4U9H1|S4U9H1_ORNSA                L--FTFRQVGLICERMMKERESQIREQYDQVLSTKLAEQYDTFVKFTY------------          158
tr|S4UD40|S4UD40_ORNMO                L--FTFRQVGLICERMMKERESQIREQYDQVLSTKLAEQYDTFVKFTY------------          157
tr|S4U8D6|S4U8D6_RHIMP                L--FTFRQVGLICERMMKERESKIREEYDHVLSTKLAEQYDTFVKFTY------------          145
tr|S4UD31|S4UD31_RHIMP                L--FTFRQVGLICERMMKERESKIREEYDHVLSTKLAEQYDTFVKFTY------------          145
tr|S4UD36|S4UD36_RHIMP                L--FTFRQVGLICERMMKERESKIREEYDHVLSTKLAEQYDTFVKFTY------------          145
tr|S4U9H0|S4U9H0_RHIEV                L--FTFRQVGLICERMMKERESKIREEYDHVLSTKLAEQYDTFVKFTY------------          145
tr|S4U8E0|S4U8E0_9ACAR                L--FTFRQVGLICERMMKERESKIREEYDHVLSTKLAEQYDTFVKFTY------------          145
tr|Q1AER5|Q1AER5_RHIMP                L--FTFRQVGLICERMMKERESKIREEYDHVLSTKLAEQYDTFVKFTY------------          145
tr|A0A7D5D0I3|A0A7D5D0I3_RHIAP        L--FTFRQVGLICERMMKERESKIREEYDHVLSTKLAEQYDTFVKFTY------------          145
tr|S4UAH0|S4UAH0_RHIDE                L--FTFRQVGLICERMMKERESKIREEYDHVLSTKLAEQYDTFVKFTY------------          145
tr|A0A224YH38|A0A224YH38_9ACAR        L--FTFRQVGLICERMMKERESKIREEYDHVLSTKLAEQYDTFVKFTY------------          145
tr|A0A7D5D0F6|A0A7D5D0F6_RHIDE        L--FTFRQVGLICERMMKERESKIREEYDHVLSTKLAEQYDTFVKFTY------------          145
tr|Q1AER4|Q1AER4_RHIAP                L--FTFRQVGLICERMMKERESKIREEYDHVLSTKLAEQYDTFVKFTY------------          145
tr|Q1AER3|Q1AER3_RHISA                L--FTFRQVGLICERMMKERESKIREEYDHVLSTKLAEQYDTFVKFTY------------          145
tr|A0A097BQ10|A0A097BQ10_RHIHE        L--FTSRQVGLICERMMKERESKIREEYDHVLSTKLAEQYDTFVKFTY------------          145
tr|A0A0H3WJP2|A0A0H3WJP2_RHIHE        L--FTFRQVGLICERMMKERESKIREEYDHVLSTKLAEQYDTFVKFTY------------          145
tr|A0A023FG32|A0A023FG32_AMBCJ        L--FTFRQVGLICERMMKERESQIREEYDHVLSTKLAEQYDTFVKFTY------------          159
tr|A0A1E1XVC7|A0A1E1XVC7_AMBSC        L--FTFRQVGLICERMMKERESQIREEYDHVLSTKLAEQYDTFVKFTY------------          159
tr|A0A1E1X1X1|A0A1E1X1X1_9ACAR        L--FTFRQVGLICERMMKERESQIREEYDQVLSTKLAEQYDTFVKFTY------------          162
tr|A0A023GFE4|A0A023GFE4_AMBTT        L--FTFRQVGLICERMMKERESQIREEYDHVLSTKLAEQYDTFVKFTY------------          161
tr|S4UF12|S4UF12_AMBMU                L--FTFRQVGLICERMMKERESQIREEYDHVLSTKLAEQYDTFVKFTY------------          161
tr|A0A7D4XVE8|A0A7D4XVE8_AMBVA        L--FTFRQVGLICERMMKERESQIREEYDHVLSTKLAEQYDTFVKFTY------------          161
tr|S4U8D4|S4U8D4_AMBVA                L--FTFRQVGLICERMMKERESQIREEYDHVLSTKLAEQYDTFVKFTY------------          161
tr|S4UAG1|S4UAG1_AMBCJ                L--FTFRQVGLICERMMKERESQIREEYDHVLSTKLAEQYDTFVKFTY------------          161
tr|S4U8D1|S4U8D1_AMBAM                L--FTFRQVGLICERMMKERESQIREEYDHVLSTKLAEQYDTFVKFTY------------          161
tr|A0A0C9RT91|A0A0C9RT91_AMBAM        L--FTFRQVGLICERMMKERESQIREEYDHVLSTKLAEQYDTFVKFTY------------          161
tr|Q1AER9|Q1AER9_AMBAM                L--FTFRQVGLICERMMKERESQIREEYDHVLSTKLAEQYDTFVKFTY------------          161
tr|S4U9F0|S4U9F0_AMBAM                L--FTFRQVGLICERMMKERESQIREEYDHVLSTKLAEQYDTFVKFTY------------          161
tr|S4UD38|S4UD38_DERRT                L--FTFRQVGLICERMMKERESQIREQYDHVLSTKLAEQYDTFVKFTY------------          148
tr|A0A097BQ19|A0A097BQ19_9ACAR        L--FTFRQVGLICERMMKERESQIREEYDHVLSTKLAEQYDTFVKFTY------------          149
tr|Q1AER2|Q1AER2_DERMR                L--FTFRQVGLICERMMKERESQIREEYDHVLSTKLAEQYDTFVKFTY------------          149
tr|A0A0U2ID78|A0A0U2ID78_9ACAR        L--FTFRQVGLIREQMMKERESQIREEYVHVLSTKLAEQYDTFVKFTY------------          147
tr|S4U8E3|S4U8E3_HYARU                L--FTFRQVGLICERMMKERESQIREEYDHVLSTKLAEQYDTFVKFTY------------          147
tr|A0A0A1C3I5|A0A0A1C3I5_HYAAA        L--FTFRQVGLICERMMKERESQIREEYDHVLSTKLAEQYDTFVKFTY------------          147
tr|A0A131XM91|A0A131XM91_9ACAR        L--FTFRQVGLICERMMKERESQIREEYDHVLSTKLAEQYDTFVKFTY------------          147
tr|S4UAH3|S4UAH3_9ACAR                L--FTFRQVGLICERMMKERESQIREEYDHVLSTKLAEQYDTFVKFTY------------          147
tr|Q1AER0|Q1AER0_9ACAR                L--FTFRQVGLICERMMKERESQIREEYDHVLSTKLAEQYDTFVKFTY------------          147
tr|S4UF21|S4UF21_9ACAR                L--FTFRQVGLICERMMKERESQIREEYDHVLSTKLAEQYDTFVKFTY------------          159
tr|A0A077B3N4|A0A077B3N4_HAEFA        L--FTFRQVGLICERMMKERESQIREEYDHVLSTKLAEQYDTFVKFTY------------          163
tr|Q1AEQ9|Q1AEQ9_9ACAR                L--FTFRQVGLICERMMKERESQIREEYDHVLSTKLAEQYDTFVKLTY------------          163
tr|R9R416|R9R416_AEDAL                L--FTFRQVGLICERMMKERESQIRDQYDAVLSAKLAEQYDTFVKFTY------------          199
```

```
tr|S4UAF8|S4UAF8_IXOHE              L--FTFRQVGLICERMMKERESQIREEYDRVLSAKLAEQYDTFVKFTY------------     159
tr|S4UF09|S4UF09_IXOSC              L--FTFRQVGLICERMMKERESQIRDEYDHVLSAKLAEQYDTFVKFTY------------     168
tr|S4U8C9|S4U8C9_IXOSC              L--FTFRQVGLICERMMKERESQIRDEYDHVLSAKLAEQYDTFVKFTY------------     168
tr|S4U9E5|S4U9E5_IXOSC              L--FTFRQVGLICERMMKERESQIRDEYDHVLSAKLAEQYDTFVKFTY------------     168
tr|Q1AES0|Q1AES0_IXORI              L--FTFRQVGLICERMMKERESQIRDEYDHVLSAKLAEQYDTFVKFTY------------     168
tr|A0A0A7DZX9|A0A0A7DZX9_IXOPE      L--FTFRQVGLICERMMKERESQIRDEYDHVLSAKLAEQYDTFVKFTY------------     168
tr|A0A0A7E016|A0A0A7E016_IXOPE      L--FTFRQVGLICERMMKERESQIRDEYDNVLSAKLAEQYDTFVKFTY------------     168
                                       :    : :   .*:.:            :: **:*:*. ::  *


tr|A0A0H3ZJX9|A0A0H3ZJX9_9VIBR      FKQSIIEYDAKKAEYININALMQEFELKAEALS---------------------------     265
tr|A0A0H3ZMM6|A0A0H3ZMM6_VIBSP      FKQSIIEYDAKKAEYININALMQEFELKAEALS---------------------------     265
tr|A0A2N7JR33|A0A2N7JR33_VIBSP      FKQSIIEYDAKKAEYINIDALMQEFELKAEALS---------------------------     265
tr|A0A2N7MJ04|A0A2N7MJ04_VIBSP      VKQSIIEYDAKKAEYIHIDALMQEFELKAEAL----------------------------     264
tr|A0A1Q3F078|A0A1Q3F078_CULTA      -DQIQRRYEA-APSYLS-------------------------------------------     205
tr|A0A1Q3F0C4|A0A1Q3F0C4_CULTA      -DQIQRRYEA-APSYLS-------------------------------------------     166
tr|A0A182X279|A0A182X279_ANOQN      -DQIQRRYEA-APSYLS-------------------------------------------     203
tr|A0A182I6D9|A0A182I6D9_ANOAR      -DQIQRRYEA-APSYLS-------------------------------------------     203
tr|A0A182L3Z9|A0A182L3Z9_ANOCL      -DQIQRRYEA-APSYLS-------------------------------------------     203
tr|A0A182U5E8|A0A182U5E8_9DIPT      -DQIQRRYEA-APSYLS-------------------------------------------     120
tr|A0A182VLD0|A0A182VLD0_ANOME      -DQIQRRYEA-APSYLS-------------------------------------------     203
tr|Q7QIE8|Q7QIE8_ANOGA              -DQIQRRYEA-APSYLS-------------------------------------------     203
tr|A0A182PIV6|A0A182PIV6_9DIPT      -DQIQRRYEA-APS----------------------------------------------     197
tr|A0A2M4CTL6|A0A2M4CTL6_ANODA      -DQIQRRYEA-APSYLS-------------------------------------------     204
tr|A0A2M4AHW6|A0A2M4AHW6_9DIPT      -DQIQRRYEA-APSYLS-------------------------------------------     204
tr|A0A1L3N235|A0A1L3N235_9DIPT      -DQIQRRYEA-APSYLS-------------------------------------------     203
tr|A0A1L3N228|A0A1L3N228_9DIPT      -DQIQRRYEA-APSYLS-------------------------------------------     203
tr|A0A1L3N225|A0A1L3N225_ANOFN      -DQIQRRYEA-APSYLS-------------------------------------------     203
tr|A0A182Y884|A0A182Y884_ANOST      -DQIQRRYEA-APSYLS-------------------------------------------     203
tr|A0A182WI31|A0A182WI31_9DIPT      -DQIQRRYEA-APSYLS-------------------------------------------     200
tr|A0A182R6Q1|A0A182R6Q1_ANOFN      -DQIQRRYEA-APSYLS-------------------------------------------     203
tr|A0A182QLK0|A0A182QLK0_9DIPT      -DQIQRRYEA-APSYLS-------------------------------------------     206
tr|A0A182N7F5|A0A182N7F5_9DIPT      -DQIQRRYEA-APSYLS-------------------------------------------     207
tr|A0A182FFJ8|A0A182FFJ8_ANOAL      -DQIQRRYEA-APSYLS-------------------------------------------     204
tr|A0A182MCQ5|A0A182MCQ5_9DIPT      -DQIQRRYEA-APSYLS-------------------------------------------     120
tr|A0A182SNZ4|A0A182SNZ4_9DIPT      -DQIQRRYEA-APSYLS-------------------------------------------     120
tr|A0A4Y0BFV2|A0A4Y0BFV2_ANOFN      -DQIQRRYEA-APSYLS-------------------------------------------     120
tr|A0A182J5N6|A0A182J5N6_9DIPT      -DQIQRRYEA-APSYLS-------------------------------------------     211
tr|A0A084WMV7|A0A084WMV7_ANOSI      -DQIQRRYEA-APSYLS-------------------------------------------     120
tr|A0A2M3Z691|A0A2M3Z691_9DIPT      -DQIQRRYEA-APSYLS-------------------------------------------     204
tr|A0A1L3N222|A0A1L3N222_AEDAE      -DQIQRRYEA-APSYLS-------------------------------------------     198
tr|Q16MV0|Q16MV0_AEDAE              -DQIQRRYEA-APSYLS-------------------------------------------     198
tr|A0A023EJ88|A0A023EJ88_AEDAL      -DQIQRRYEA-APSYLS-------------------------------------------     198
tr|A0A182H6N2|A0A182H6N2_AEDAL      -DQIQRRYEA-APSCEYHYYYQL-FEI---------------------------------     207
tr|A0A131YRI7|A0A131YRI7_RHIAP      -DQIQRRYDSVMPSYLS-------------------------------------------     173
tr|R9R3J1|R9R3J1_AEDAL              -DQIQKRFEGATPSYLSGGGSHKPFGSPSSPSSSAIAAAAAAAKRPSPFAEAVCPKQLTF     173
tr|A0A0D5Y9E2|A0A0D5Y9E2_9ACAR      -DQ---------------------------------------------------------     158
tr|A0A2R5LKD0|A0A2R5LKD0_9ACAR      -DQIQKKFEGATPSYLS-------------------------------------------     173
tr|S4U9H1|S4U9H1_ORNSA              -DQIQKKFEGATPSYLS-------------------------------------------     174
tr|S4UD40|S4UD40_ORNMO              -DQIQKKFEGATPSYLS-------------------------------------------     173
tr|S4U8D6|S4U8D6_RHIMP              -DQ---------------------------------------------------------     147
tr|S4UD31|S4UD31_RHIMP              -DQ---------------------------------------------------------     147
tr|S4UD36|S4UD36_RHIMP              -DQK--------------------------------------------------------     148
tr|S4U9H0|S4U9H0_RHIEV              -DQIQKRFEGATPSYLS-------------------------------------------     161
tr|S4U8E0|S4U8E0_9ACAR              -DQ---------------------------------------------------------     147
tr|Q1AER5|Q1AER5_RHIMP              -DQ---------------------------------------------------------     147
tr|A0A7D5D0I3|A0A7D5D0I3_RHIAP      -DQIQKRFEGATPSYLS-------------------------------------------     161
tr|S4UAH0|S4UAH0_RHIDE              -DQ---------------------------------------------------------     147
tr|A0A224YH38|A0A224YH38_9ACAR      -DQIQKRFEGATPSYLS-------------------------------------------     161
tr|A0A7D5D0F6|A0A7D5D0F6_RHIDE      -DQIQKRFEGATPSYLS-------------------------------------------     161
tr|Q1AER4|Q1AER4_RHIAP              -DQIQKRFEGATPSYLS-------------------------------------------     161
tr|Q1AER3|Q1AER3_RHISA              -DQIQKRFEGATPSYLS-------------------------------------------     161
tr|A0A097BQ10|A0A097BQ10_RHIHE      -DQIQKRFEGATPSYLS-------------------------------------------     161
tr|A0A0H3WJP2|A0A0H3WJP2_RHIHE      -DQIQKRFEGATPSYLS-------------------------------------------     161
tr|A0A023FG32|A0A023FG32_AMBCJ      -DQIQKRFEG--------------------------------------------------     168
tr|A0A1E1XVC7|A0A1E1XVC7_AMBSC      -DQIQKRFEGATPSYLS-------------------------------------------     175
tr|A0A1E1X1X1|A0A1E1X1X1_9ACAR      -DQIQKRFEGATPSYLS-------------------------------------------     178
tr|A0A023GFE4|A0A023GFE4_AMBTT      -DQIQKRFEGATPSYLS-------------------------------------------     177
tr|S4UF12|S4UF12_AMBMU              -DQ---------------------------------------------------------     163
tr|A0A7D4XVE8|A0A7D4XVE8_AMBVA      -DQIQKRFEGATPSYLS-------------------------------------------     177
tr|S4U8D4|S4U8D4_AMBVA              -DQ---------------------------------------------------------     163
tr|S4UAG1|S4UAG1_AMBCJ              -DQ---------------------------------------------------------     163
tr|S4U8D1|S4U8D1_AMBAM              -DQII-------------------------------------------------------     165
tr|A0A0C9RT91|A0A0C9RT91_AMBAM      -DQIQKRFEGATPSYLS-------------------------------------------     177
tr|Q1AER9|Q1AER9_AMBAM              -DQ---------------------------------------------------------     163
tr|S4U9F0|S4U9F0_AMBAM              -DQ---------------------------------------------------------     163
tr|S4UD38|S4UD38_DERRT              -DQ---------------------------------------------------------     150
tr|A0A097BQ19|A0A097BQ19_9ACAR      -DQIQKRFEGATPSYLS-------------------------------------------     165
tr|Q1AER2|Q1AER2_DERMR              -DQIQKRFEGATPSYLS-------------------------------------------     165
tr|A0A0U2ID78|A0A0U2ID78_9ACAR      -DQIQKRFEGATPSYLS-------------------------------------------     163
tr|S4U8E3|S4U8E3_HYARU              -DQ---------------------------------------------------------     149
tr|A0A0A1C3I5|A0A0A1C3I5_HYAAA      -DQIQKRFEGATPSYLS-------------------------------------------     163
tr|A0A131XM91|A0A131XM91_9ACAR      -DQIQKRFEGATPSYLS-------------------------------------------     163
tr|S4UAH3|S4UAH3_9ACAR              -DQIQKRFEGATPSYLS-------------------------------------------     163
tr|Q1AER0|Q1AER0_9ACAR              -DQ---------------------------------------------------------     149
tr|S4UF21|S4UF21_9ACAR              -DQIQKRFEGATPSYLS-------------------------------------------     175
tr|A0A077B3N4|A0A077B3N4_HAEFA      -DQIQKRFEGATPSYLS-------------------------------------------     179
tr|Q1AEQ9|Q1AEQ9_9ACAR              -DQIQKRFEGATPSYLS-------------------------------------------     179
tr|R9R416|R9R416_AEDAL              -DQIQKRFEGAAPSYLS-------------------------------------------     215
tr|S4UAF8|S4UAF8_IXOHE              -DQIQKRFEGATPSYLS-------------------------------------------     175
tr|S4UF09|S4UF09_IXOSC              -DQIQKRFEGATPSYLS-------------------------------------------     184
tr|S4U8C9|S4U8C9_IXOSC              -DQIQKRFEGATPSYLS-------------------------------------------     184
tr|S4U9E5|S4U9E5_IXOSC              -DQIQKRFEGATPSYLS-------------------------------------------     184
tr|Q1AES0|Q1AES0_IXORI              -DQIQKRFEGATPSYLS-------------------------------------------     184
```

```
tr|A0A0A7DZX9|A0A0A7DZX9_IXOPE      -DQIQKRFEGATPSYLS-------------------------------------------           184
tr|A0A0A7E016|A0A0A7E016_IXOPE      -DQIQKRFEGATPSYLS-------------------------------------------           184
                                     .*

tr|A0A0H3ZJX9|A0A0H3ZJX9_9VIBR      ----------------------------------------------265
tr|A0A0H3ZMM6|A0A0H3ZMM6_VIBSP      ----------------------------------------------265
tr|A0A2N7JR33|A0A2N7JR33_VIBSP      ----------------------------------------------265
tr|A0A2N7MJ04|A0A2N7MJ04_VIBSP      ----------------------------------------------264
tr|A0A1Q3F078|A0A1Q3F078_CULTA      ----------------------------------------------205
tr|A0A1Q3F0C4|A0A1Q3F0C4_CULTA      ----------------------------------------------166
tr|A0A182X279|A0A182X279_ANOQN      ----------------------------------------------203
tr|A0A182I6D9|A0A182I6D9_ANOAR      ----------------------------------------------203
tr|A0A182L3Z9|A0A182L3Z9_ANOCL      ----------------------------------------------203
tr|A0A182U5E8|A0A182U5E8_9DIPT      ----------------------------------------------120
tr|A0A182VLD0|A0A182VLD0_ANOME      ----------------------------------------------203
tr|Q7QIE8|Q7QIE8_ANOGA              ----------------------------------------------203
tr|A0A182PIV6|A0A182PIV6_9DIPT      ----------------------------------------------197
tr|A0A2M4CTL6|A0A2M4CTL6_ANODA      ----------------------------------------------204
tr|A0A2M4AHW6|A0A2M4AHW6_9DIPT      ----------------------------------------------204
tr|A0A1L3N235|A0A1L3N235_9DIPT      ----------------------------------------------203
tr|A0A1L3N228|A0A1L3N228_9DIPT      ----------------------------------------------203
tr|A0A1L3N225|A0A1L3N225_ANOFN      ----------------------------------------------203
tr|A0A182Y884|A0A182Y884_ANOST      ----------------------------------------------203
tr|A0A182WI31|A0A182WI31_9DIPT      ----------------------------------------------200
tr|A0A182R6Q1|A0A182R6Q1_ANOFN      ----------------------------------------------203
tr|A0A182QLK0|A0A182QLK0_9DIPT      ----------------------------------------------206
tr|A0A182N7F5|A0A182N7F5_9DIPT      ----------------------------------------------207
tr|A0A182FFJ8|A0A182FFJ8_ANOAL      ----------------------------------------------204
tr|A0A182MCQ5|A0A182MCQ5_9DIPT      ----------------------------------------------120
tr|A0A182SNZ4|A0A182SNZ4_9DIPT      ----------------------------------------------120
tr|A0A4Y0BFV2|A0A4Y0BFV2_ANOFN      ----------------------------------------------120
tr|A0A182J5N6|A0A182J5N6_9DIPT      ----------------------------------------------211
tr|A0A084WMV7|A0A084WMV7_ANOSI      ----------------------------------------------120
tr|A0A2M3Z691|A0A2M3Z691_9DIPT      ----------------------------------------------204
tr|A0A1L3N222|A0A1L3N222_AEDAE      ----------------------------------------------198
tr|Q16MV0|Q16MV0_AEDAE              ----------------------------------------------198
tr|A0A023EJ88|A0A023EJ88_AEDAL      ----------------------------------------------198
tr|A0A182H6N2|A0A182H6N2_AEDAL      ----------------------------------------------207
tr|A0A131YRI7|A0A131YRI7_RHIAP      ----------------------------------------------173
tr|R9R3J1|R9R3J1_AEDAL              NTGSRPDSPPSMVLFTFKQALREQYDAVLTNKLAEQYDAAAPSYLS219
tr|A0A0D5Y9E2|A0A0D5Y9E2_9ACAR      ----------------------------------------------158
tr|A0A2R5LKD0|A0A2R5LKD0_9ACAR      ----------------------------------------------173
tr|S4U9H1|S4U9H1_ORNSA              ----------------------------------------------174
tr|S4UD40|S4UD40_ORNMO              ----------------------------------------------173
tr|S4U8D6|S4U8D6_RHIMP              ----------------------------------------------147
tr|S4UD31|S4UD31_RHIMP              ----------------------------------------------147
tr|S4UD36|S4UD36_RHIMP              ----------------------------------------------148
tr|S4U9H0|S4U9H0_RHIEV              ----------------------------------------------161
tr|S4U8E0|S4U8E0_9ACAR              ----------------------------------------------147
tr|Q1AER5|Q1AER5_RHIMP              ----------------------------------------------147
tr|A0A7D5D0I3|A0A7D5D0I3_RHIAP      ----------------------------------------------161
tr|S4UAH0|S4UAH0_RHIDE              ----------------------------------------------147
tr|A0A224YH38|A0A224YH38_9ACAR      ----------------------------------------------161
tr|A0A7D5D0F6|A0A7D5D0F6_RHIDE      ----------------------------------------------161
tr|Q1AER4|Q1AER4_RHIAP              ----------------------------------------------161
tr|Q1AER3|Q1AER3_RHISA              ----------------------------------------------161
tr|A0A097BQ10|A0A097BQ10_RHIHE      ----------------------------------------------161
tr|A0A0H3WJP2|A0A0H3WJP2_RHIHE      ----------------------------------------------161
tr|A0A023FG32|A0A023FG32_AMBCJ      ----------------------------------------------168
tr|A0A1E1XVC7|A0A1E1XVC7_AMBSC      ----------------------------------------------175
tr|A0A1E1X1X1|A0A1E1X1X1_9ACAR      ----------------------------------------------178
tr|A0A023GFE4|A0A023GFE4_AMBTT      ----------------------------------------------177
tr|S4UF12|S4UF12_AMBMU              ----------------------------------------------163
tr|A0A7D4XVE8|A0A7D4XVE8_AMBVA      ----------------------------------------------177
tr|S4U8D4|S4U8D4_AMBVA              ----------------------------------------------163
tr|S4UAG1|S4UAG1_AMBCJ              ----------------------------------------------163
tr|S4U8D1|S4U8D1_AMBAM              ----------------------------------------------165
tr|A0A0C9RT91|A0A0C9RT91_AMBAM      ----------------------------------------------177
tr|Q1AER9|Q1AER9_AMBAM              ----------------------------------------------163
tr|S4U9F0|S4U9F0_AMBAM              ----------------------------------------------163
tr|S4UD38|S4UD38_DERRT              ----------------------------------------------150
tr|A0A097BQ19|A0A097BQ19_9ACAR      ----------------------------------------------165
tr|Q1AER2|Q1AER2_DERMR              ----------------------------------------------165
tr|A0A0U2ID78|A0A0U2ID78_9ACAR      ----------------------------------------------163
tr|S4U8E3|S4U8E3_HYARU              ----------------------------------------------149
tr|A0A0A1C3I5|A0A0A1C3I5_HYAAA      ----------------------------------------------163
tr|A0A131XM91|A0A131XM91_9ACAR      ----------------------------------------------163
tr|S4UAH3|S4UAH3_9ACAR              ----------------------------------------------163
tr|Q1AER0|Q1AER0_9ACAR              ----------------------------------------------149
tr|S4UF21|S4UF21_9ACAR              ----------------------------------------------175
tr|A0A077B3N4|A0A077B3N4_HAEFA      ----------------------------------------------179
tr|Q1AEQ9|Q1AEQ9_9ACAR              ----------------------------------------------179
tr|R9R416|R9R416_AEDAL              ----------------------------------------------215
tr|S4UAF8|S4UAF8_IXOHE              ----------------------------------------------175
tr|S4UF09|S4UF09_IXOSC              ----------------------------------------------184
tr|S4U8C9|S4U8C9_IXOSC              ----------------------------------------------184
tr|S4U9E5|S4U9E5_IXOSC              ----------------------------------------------184
tr|Q1AES0|Q1AES0_IXORI              ----------------------------------------------184
tr|A0A0A7DZX9|A0A0A7DZX9_IXOPE      ----------------------------------------------184
tr|A0A0A7E016|A0A0A7E016_IXOPE      ----------------------------------------------184
```

(B) Sequence alignment (Align, https://www.uniprot.org/align/) for SUB SAKLAEQYDTFVKFTYDQIQKRFEGATPSYLS in tick spp.

Results: 71.9% identity.

S-A/T-KLAEQYD-T/A-FVKFTYDQI-Q/E-K/R-R-F/Y-E/G/D-G/S/A/D/T-A/V-T/R/M-PSYLS

```
CLUSTAL O(1.2.4) multiple sequence alignment
Results: 100% identity.

tr|B5L666|B5L666_RHIMP              -------------------------------------RPTD--------------KPLRL     9
tr|B5L665|B5L665_RHIMP              ------MEAGIGAAS---SGSSAQETTWKAPWPLT-SRM----------LKSRRFTTLLL     40
tr|A0A6M2D4F4|A0A6M2D4F4_RHIMP      ----RPLAAGIGAAS---SGSSAQETTWKAPWPLT-SRR----------LKSRRFTTLLL     42
tr|Q6JIC2|Q6JIC2_ANAMA              MNYRELFTGGLSAATVCACSLLVSGAVVASPMS---------------------------     33
tr|R9R561|R9R561_9DIPT              ------------------------------------------------------------     0
tr|S4U8E4|S4U8E4_9DIPT              ----------------MACATLKRSLDW-VSLN---QRP----------TKRRRRDRFGS     30
tr|S4UAH5|S4UAH5_CULPP              ----------------MACATLECSLDW-ESPN---QRP----------TKRRRCHPFGS     30
tr|C3RX28|C3RX28_AEDAL              ----------------MACATLKRSLDW-ESLN---QRP----------TKRRRCHPFGS     30
tr|A0A3M7Q970|A0A3M7Q970_BRAPC      ----------------MACCTTI------SPLNSAQKRRTSAMFDFPHSAKRRRPLSSAH     38
tr|J7K3S8|J7K3S8_9ACAR              ----------------MACATLKRASSW-DPLS-PEGKP----------SKRRRFY-A-T     30
tr|J7K8Y7|J7K8Y7_9ACAR              ----------------MACATLKRASSW-DPLS-PEGKP----------SKRRRFY-A-T     30
tr|J7K9Q0|J7K9Q0_9ACAR              ----------------MACATLKRASSW-DPLS-PEGKP----------SKRRRFY-A-T     30
tr|J7K8Z4|J7K8Z4_9ACAR              ----------------MACATLKRASSW-DPLS-PEGKP----------SKRRRFY-A-T     30
tr|J7K706|J7K706_9ACAR              ----------------MACATLKRASSW-DPLS-PEGKP----------SKRRRFY-A-T     30
tr|A0A0X9PMU2|A0A0X9PMU2_9ACAR      ----------------MACATLKRASSW-DPLS-PEGKP----------SKRRRFY-A-T     30
tr|J7K9P7|J7K9P7_9ACAR              ----------------MACATLKRASSW-DPLS-PEGKP----------SKRRRFY-A-T     30
tr|A0A9J6F3J6|A0A9J6F3J6_RHIMP      ----------------MACATLKRSTSC-DPLS-PDGSS----------AKRRRLS-LAT     31
tr|A0A9D4Q191|A0A9D4Q191_RHISA      ----------------MACATLKRSSSC-DPLS-PNGNS----------AKRRRFC-LST     31
tr|A0A131YRI7|A0A131YRI7_RHIAP      ----------------MACATLKRSNSC-DPLS-PNENS----------AKRRRFC-LST     31
tr|A0A224Z051|A0A224Z051_9ACAR      ----------------MACATLKRSNSC-DPLS-PNGNS----------AKRQRFC-LST     31
tr|A0A443S7G3|A0A443S7G3_9ACAR      ----------------MACATLKRSLDW-DPLNSPSNSGTQ------SRPSKKRCLNHMY     37
tr|R9R3J1|R9R3J1_AEDAL              ----------------MACATLKRTHDW-DPLHSPNGRS----------PK---------     24
tr|A0A0B5J531|A0A0B5J531_9ACAR      ----------------MACATLKRTHDW-DPLHSPNGRS----------PKRRRCMPLSV     33
tr|S4UAF8|S4UAF8_IXOHE              ----------------MACATLKRTHDW-DPLHSPNGRS----------PKRRRCMPLSV     33
tr|R9R416|R9R416_AEDAL              ----------------MACATLKRTHDW-DPLHSPNGRS----------PKRRRCMPLGS     33
tr|Q1AES0|Q1AES0_IXORI              ----------------MACATLKRTHDW-DPLHSPNGRS----------PKRRRCMPLSV     33
tr|S4UF09|S4UF09_IXOSC              ----------------MACATLKRTHDW-DPLHSPTGRS----------PKRRRCMPLSV     33
tr|S4U8C9|S4U8C9_IXOSC              ----------------MACATLKRTHDW-YPLHSPNGRS----------PKRRRCMPLSV     33
tr|S4U9E5|S4U9E5_IXOSC              ----------------MACATLKRTHDW-DPLHSPNGRS----------PKRRRCMPLSV     33
tr|A0A0A7E016|A0A0A7E016_IXOPE      ----------------MACATLKRTHDW-DPLHSPNGRS----------PKRRRCMPLSV     33
tr|Q4VRW2|Q4VRW2_IXOSC              ----------------MACATLKRTHDW-DPLHSPNGRS----------PKRRRCMPLSV     33
tr|A0A0A7DZX9|A0A0A7DZX9_IXOPE      ----------------MACATLKRTHDW-DPLHSPNGRS----------PKRRRCMPLSV     33
tr|W8E787|W8E787_IXOPE              ------------------------------------------------------------     0
tr|W8E0V9|W8E0V9_IXOPE              ------------------------------------------------------------     0
tr|W8E329|W8E329_9ACAR              ------------------------------------------------------------     0
tr|A0A1E1XVC7|A0A1E1XVC7_AMBSC      ----------------MACATLKRTHDW-DPLHSPNGRS----------PKRRRCMPFSV     33
tr|A0A1E1X1X1|A0A1E1X1X1_9ACAR      ----------------MACATLKRTHDW-DPLHSPNGRS----------PKRRRCMPFSV     33
tr|S4U9F0|S4U9F0_AMBAM              ----------------MACATLKRTHDW-DPLHSPNGRS----------PKRRRCMPFSV     33
tr|A0A0C9RT91|A0A0C9RT91_AMBAM      ----------------MACATLKRTHDW-DPLHSPNGRS----------PKRRRCMPFSV     33
tr|S4U8D1|S4U8D1_AMBAM              ----------------MACATLKRTHDW-DPLHSPNGRS----------PKRRRCMPFSV     33
tr|Q1AER9|Q1AER9_AMBAM              ----------------MACATLKRTHDW-DPLHSPNGRS----------PKRRRCMPFSV     33
tr|S4UF12|S4UF12_AMBMU              ----------------MACATLKRTHDW-DPLHSPNGRS----------PKRRRCMPLSV     33
tr|S4UAG1|S4UAG1_AMBCJ              ----------------MACATLKRTHDW-DPLHSPNGRS----------AKRRRCMPFSV     33
tr|S4U8D4|S4U8D4_AMBVA              ----------------MACATLKRTHDW-DPLHSPNGRS----------PKRRRCMPLSV     33
tr|A0A7D4XVE8|A0A7D4XVE8_AMBVA      ----------------MACATLKRTHDW-DPLHSPNGRS----------PKRRRCMPLSV     33
tr|B7SCS6|B7SCS6_AMBHE              ----------------MACATLKRTHDW-DPLHSPNGRS----------PKRRRCMPLSV     33
tr|Q1AER3|Q1AER3_RHISA              ----------------MACATLKRTHDW-DPLHSPSGRS----------PKRRRCMPLS-     32
tr|A0A097BQ10|A0A097BQ10_RHIHE      ----------------MACATLKRTHDW-DPLHSPSGRS----------PKRRRCMPLS-     32
tr|A0A0H3WJP2|A0A0H3WJP2_RHIHE      ----------------MACATLKRTHDW-DPLHSPSGRS----------PKRRRCMPLS-     32
tr|I0B6E2|I0B6E2_RHIMP              ----------------MACATLKRTHDW-DPLHSPSGRS----------PKRRRCMPLS-     32
tr|I3XPN4|I3XPN4_RHIMP              ----------------MACATLKRTHDW-DPLHSPSGRS----------PKRRRCMPLS-     32
tr|I0B6D9|I0B6D9_RHIMP              ----------------MACATLKRTHDW-DPLHSPSGRS----------PKRRRCMPLS-     32
tr|I0B6D7|I0B6D7_RHIMP              ----------------MACATLKRTHDW-DPLHSPSGRS----------PKRRRCMPLS-     32
tr|I0B6E5|I0B6E5_RHIMP              ----------------MACATLKRTHDW-DPLHSPSGRS----------PKRRRCMPLS-     32
tr|S4UD36|S4UD36_RHIMP              ----------------MACATLKRTHDW-DPLHSQSGRS----------PKRRRCMPLS-     32
tr|S4U8E0|S4U8E0_9ACAR              ----------------MACATLKRTHDW-DPLHSPSGRS----------PKRRRCMPLS-     32
tr|A0A7D5D0F6|A0A7D5D0F6_RHIDE      ----------------MACATLKRTHDW-DPLHSPSGRS----------PKRRRCMPLS-     32
tr|J7K2D7|J7K2D7_RHIMP              ----------------MACATLKRTHDW-DPLHSLSGRS----------PKRRRCMPLS-     32
tr|I0B6E3|I0B6E3_RHIMP              ----------------MACATLKRTHDW-DPLHSSSGRS----------PKRRRCMPLS-     32
tr|S4U8D6|S4U8D6_RHIMP              ----------------MACATLKRIHDW-DPLHSPSGSS----------PKRRRCMPLS-     32
tr|S4UD31|S4UD31_RHIMP              ----------------MACATLKRTHDW-DPLHSPSGSS----------PKRRRCMPLS-     32
tr|Q1AER5|Q1AER5_RHIMP              ----------------MACATLKRTHDW-DPLHSPSGRS----------PKRRRCMPLS-     32
tr|A0A7D5D0I3|A0A7D5D0I3_RHIAP      ----------------MACATLKRTHDW-DPLHSPSGRS----------PKRRRCMPLS-     32
tr|J7K8Z9|J7K8Z9_RHIMP              ----------------MACATLKRTHDW-DPLHSPSGRS----------PKRRRCMPLS-     32
tr|I0B6D6|I0B6D6_RHIMP              ----------------MACATLKRTHDW-DPLHSPSGRS----------PKRRRCMPLS-     32
tr|S4U9H0|S4U9H0_RHIEV              ----------------MACATLKRTHDW-DPLHSPSGRS----------PKRRRCMPLS-     32
tr|J7K715|J7K715_RHIMP              ----------------MACATLKRTHDW-DPLHSPSGRS----------PKRRRCMPLS-     32
tr|S4UAH0|S4UAH0_RHIDE              ----------------MACATLKRTHDW-DPLHSPSGRS----------PKRRRCMPLS-     32
tr|Q1AER6|Q1AER6_RHIMP              ----------------MACATLKRTHDW-DPLHSPSGRS----------PKRRRCMPLS-     32
tr|B6CM56|B6CM56_RHIMP              ----------------MACATLKRTHDW-DPLHSPSGRS----------PKRRRCMPLS-     32
tr|S4UD38|S4UD38_DERRT              ----------------MACATLKRTHDW-DPLHSPNGRS----------PKRRRCMPLSV     33
tr|A0A097BQ19|A0A097BQ19_9ACAR      ----------------MACATLKRTHDW-DPLHSPNGRS----------PKRRRCMPLSV     33
tr|K9R5H5|K9R5H5_DERSI              ----------------MACATLKRTHDW-DPLHSPNGRS----------PKRRRCMPLSV     33
tr|A0A0U2ID78|A0A0U2ID78_9ACAR      ----------------MACATLKRTHDW-DPLHSPNGRS----------PKRRRCMPL--     31
tr|A0A0U2L412|A0A0U2L412_9ACAR      ----------------MACATLKRTHDR-GPLHSPNGRS----------PKRRRCMPL--     31
tr|A0A0U2TZZ5|A0A0U2TZZ5_9ACAR      ----------------MACATLKRTHDW-DPLHSPNGRS----------PKRRRCMPL--     31
tr|S4U8E3|S4U8E3_HYARU              ----------------MACATLKRTHDW-DPLHSPNGRS----------PKRRRCMPL--     31
tr|A0A0U2URL3|A0A0U2URL3_9ACAR      ----------------MACATLKRTHDW-DPLHSPNGRS----------PKRRRCMPL--     31
tr|A0A0U2T382|A0A0U2T382_9ACAR      ----------------MACATLKRTHDW-DPLHSPNGRS----------PKRRRCMPL--     31
```

```
tr|A0A0U2LZ49|A0A0U2LZ49_9ACAR      ----------------MACATLKRTHDW-DPLHSPNGRS----------PKRRRCMPL--        31
tr|A0A0U2T040|A0A0U2T040_9ACAR      ----------------MACATLKRTHGW-DPLHSPNGRS----------PKRRRCMPL--        31
tr|A0A0U2SNR8|A0A0U2SNR8_9ACAR      ----------------MACATLKRTHDW-DPLHSPNGRS----------PKRRRCMPL--        31
tr|A0A0U2K641|A0A0U2K641_9ACAR      ----------------MACATLKRTHDW-DPLHSPNGRS----------PKRRRCMPL--        31
tr|S4UAH3|S4UAH3_9ACAR              ----------------MACATLKRTHDW-DPLHSPNGRS----------PKRRRCMPL--        31
tr|A0A131XM91|A0A131XM91_9ACAR      ----------------MACATLKRTHDW-DPLHSPNGRS----------PKRRRCMPL--        31
tr|A0A077B3N4|A0A077B3N4_HAEFA      ----------------MACATLKRTHDW-DPLHSPNGRS----------PKRRRCMPLSV        33
tr|S4UF21|S4UF21_9ACAR              ----------------MACATLKRTHDW-DPLHSPNGRS----------PKRRRCMPLSV        33
tr|A0A7L7T4G3|A0A7L7T4G3_HAELO      ----------------MACATLKRTHDW-DPLHSPNGRS----------PKRRRCMPLSV        33
tr|E4W3Y9|E4W3Y9_HAELO              ----------------MACATLKRTHDW-DPLHSPNGRS----------PKRRRCMPLSV        33
tr|A0A293MGQ8|A0A293MGQ8_ORNER      ------------------------------------------------------------        0
tr|S4U9H1|S4U9H1_ORNSA              ----------------MACATLKRTHDW-DPLHSPNGRS----------PKRRRCMPFSV        33
tr|S4UD40|S4UD40_ORNMO              ----------------MACATLKRTHDW-DPLHSPNGRS----------PKRRRCMPFSV        33
tr|E2IU45|E2IU45_ORNMO              ----------------MACATLKRTHDW-DPLHSPNGRS----------PKRRRCMPFSV        33
tr|E2IU46|E2IU46_ORNER              ----------------MACATLKRTHDW-DPLHSPNGRA----------SKRRRCVPLCV        33
tr|A0A0D5Y9E2|A0A0D5Y9E2_9ACAR      -----------------ACATLKRTHDW-DPLHSPNGRA----------PKRRRCMPLCV        32
tr|A0A2R5LKD0|A0A2R5LKD0_9ACAR      ----------------MACATLKRTHDW-DPLHSPNGRA----------PKRRRCMPLCV        33


tr|B5L666|B5L666_RHIMP              PLQDVY-------------------------KIGGIGTVPVGRVETGVLKPGMVVTFAPA        44
tr|B5L665|B5L665_RHIMP              PAIQRPGMATTITESGSHGVFC------VLTVPIALGVTPWR-----------GSTEI--        81
tr|A0A6M2D4F4|A0A6M2D4F4_RHIMP      PAIQRPGMATTITESGSHGVFC------VLTVPIALGVTPWR-----------GSTEI--        83
tr|Q6JIC2|Q6JIC2_ANAMA              --------------------------HEVASEGGVMGGSFYVGAAYSPAFPSVTSFDMRE        67
tr|R9R561|R9R561_9DIPT              -----------------------------------------------------SPDKMAA        7
tr|S4U8E4|S4U8E4_9DIPT              PSQTASASSSSASPSGSSSTSVAAAAATASMRTIEPKPSPFA----EAACSKLTPEKIVQ        86
tr|S4UAH5|S4UAH5_CULPP              PAGAAGSSTSISNAL---NSSPSSSSAATRSAVMEPKPSPFA----ESFVLHLTPEKLAQ        83
tr|C3RX28|C3RX28_AEDAL              PSSNAPNSPSSSAIA---AAAAAASSSNSAMRVMEPKPSPFA----EAVCPKLTPEKMAQ        83
tr|A0A3M7Q970|A0A3M7Q970_BRAPC      PASTMLTTTATV--LNSNKQVVIESADKKAGAFQPLPCSPTGGLVSDECNELQAKSELME        96
tr|J7K3S8|J7K3S8_9ACAR              ASDPTPPA----------------PRHGPPTRPQQVRSSPFV-----DATPKMTTGEIEA        69
tr|J7K8Y7|J7K8Y7_9ACAR              ASDPTPPA----------------PRHDPPTRPQQVRSSPFV-----DATPKMTTGEIEA        69
tr|J7K9Q0|J7K9Q0_9ACAR              ASDPTPPA----------------PRHDPPTRPQQVRSSPFV-----DATPKMTTGEIEA        69
tr|J7K8Z4|J7K8Z4_9ACAR              ASDPTPPA----------------PRHDPPTRPQQVRSSPFV-----DATPKMTAGEIEA        69
tr|J7K706|J7K706_9ACAR              ASDPTPPA----------------PRHDPPTRPQQVRSSPFV-----DATPKMTTGEIEA        69
tr|A0A0X9PMU2|A0A0X9PMU2_9ACAR      ASDPTPPA----------------PRHDPPTRPQQVRSSPFV-----DATPKMTTGEIEA        69
tr|J7K9P7|J7K9P7_9ACAR              ASDPTPPA----------------PRHDPPTRPQQVRSSPFV-----DATPKMTTGEIEA        69
tr|A0A9J6F3J6|A0A9J6F3J6_RHIMP      SSDT---V----------------PRRTLQARPQQAHSSSFV-----DATPKITSEEIEA        67
tr|A0A9D4Q191|A0A9D4Q191_RHISA      SSVTTPHT----------------PRHALQARPHQVRSSPFV-----DATPKMTSEEIEA        70
tr|A0A131YRI7|A0A131YRI7_RHIAP      SSAT---T----------------PRHALQARPHQARSSPFV-----DATPKMTSEEIEA        67
tr|A0A224Z051|A0A224Z051_9ACAR      TSAT---T----------------PRHALQARPHQARSSPFV-----DATPKMTSEEIEA        67
tr|A0A443S7G3|A0A443S7G3_9ACAR      TSI------------------------------PPKDPSPFG-----EVKPKITPEYIAA        62
tr|R9R3J1|R9R3J1_AEDAL              -------------------------------------PSPFG-----EVPPKSSP-----        37
tr|A0A0B5J531|A0A0B5J531_9ACAR      TQAATPPT-----------------------RAHQINPSPFG-----EVPPKLTSEEIAA        65
tr|S4UAF8|S4UAF8_IXOHE              TQAATPPT-----------------------RAHQINPSPFG-----EVPPKLTSEEIAA        65
tr|R9R416|R9R416_AEDAL              PSSNAPNSPSSS--AIAAA-AAAAHQINSAMRVMEPKPSPFAE----AVCPKLTSEEIAA        86
tr|Q1AES0|Q1AES0_IXORI              TQAATPPT-----------------------RAHQINPSPFG-----EVPPKLTSEEIAA        65
tr|S4UF09|S4UF09_IXOSC              TQAATPPT-----------------------RAHQINPSPFG-----EVPPKLTSEEIAA        65
tr|S4U8C9|S4U8C9_IXOSC              TQAATPPT-----------------------RAHQINPSPFG-----EVPPKLTSEEIAA        65
tr|S4U9E5|S4U9E5_IXOSC              TQAATPPT-----------------------RAHQINPSPFG-----EVPPKLTSEEIAA        65
tr|A0A0A7E016|A0A0A7E016_IXOPE      TQAATPPT-----------------------RAHQINPSPFG-----EVPPKLTSEEIAA        65
tr|Q4VRW2|Q4VRW2_IXOSC              TQAATPPT-----------------------RAHQINPSPFG-----EVPPKLTSEEIAA        65
tr|A0A0A7DZX9|A0A0A7DZX9_IXOPE      TQAATPPT-----------------------RAHQINPSPFG-----EVPPKLTSEEIAA        65
tr|W8E787|W8E787_IXOPE              -------------------------------------------------------EEIAA        5
tr|W8E0V9|W8E0V9_IXOPE              -------------------------------------------------------EEIAA        5
tr|W8E329|W8E329_9ACAR              -------------------------------------------------------EEIAA        5
tr|A0A1E1XVC7|A0A1E1XVC7_AMBSC      SPPAPPT------------------------RAHQINPSPFG-----DVPPKLTSEEIAA        64
tr|A0A1E1X1X1|A0A1E1X1X1_9ACAR      SPPAPPT------------------------RAHQINPSPFG-----DVPPKLTSEEIAA        64
tr|S4U9F0|S4U9F0_AMBAM              NPPAPPT------------------------RAHQINPSPFG-----DVPPKLTSEEIAA        64
tr|A0A0C9RT91|A0A0C9RT91_AMBAM      NPPAPPT------------------------RAHQINPSPFG-----DVPPKLTSEEIAA        64
tr|S4U8D1|S4U8D1_AMBAM              NPPAPPT------------------------RAHQINPSPFG-----DVPPKLTSEEIAA        64
tr|Q1AER9|Q1AER9_AMBAM              NPPAPPT------------------------RAHQINPSPFG-----DVPPKLTSEEIAA        64
tr|S4UF12|S4UF12_AMBMU              SPAAPPT------------------------RAHQINPSPFG-----DVPPKLTSEEIAA        64
tr|S4UAG1|S4UAG1_AMBCJ              SPPAPPT------------------------RAHQINPSPFG-----DVPPKLTSEEIAA        64
tr|S4U8D4|S4U8D4_AMBVA              SPPAPPT------------------------RAHQINPSPFG-----DVPPKLTSEEIAA        64
tr|A0A7D4XVE8|A0A7D4XVE8_AMBVA      SPPAPPT------------------------RAHQINPSPFG-----DVPPKLTSEEIAA        64
tr|B7SCS6|B7SCS6_AMBHE              SPPTPPT------------------------RAHQINPSPFG-----DVPPKLTSEEIAA        64
tr|Q1AER3|Q1AER3_RHISA              ---PPPT------------------------RAHQIDPSPFG-----DVPPKLTSEEIAA        60
tr|A0A097BQ10|A0A097BQ10_RHIHE      ---PPPT------------------------RAHQIDPSPFG-----DVPPKLTSEEIAA        60
tr|A0A0H3WJP2|A0A0H3WJP2_RHIHE      ---PPPT------------------------RAHQIDPSPFG-----DVPPKLTSEEIAA        60
tr|I0B6E2|I0B6E2_RHIMP              ---PPPT------------------------RAHQIEPSPFG-----DVPPKLTSEEIAA        60
tr|I3XPN4|I3XPN4_RHIMP              ---PPPT------------------------RAHQIEPSPFG-----DVPPKLTSEEIAA        60
tr|I0B6D9|I0B6D9_RHIMP              ---PPPT------------------------RAHQIEPSPFG-----DVPPKLTSEEIAA        60
tr|I0B6D7|I0B6D7_RHIMP              ---PPPT------------------------RAHQIEPSPFG-----DVPPKLTSEEIAA        60
tr|I0B6E5|I0B6E5_RHIMP              ---PPPT------------------------RAHQIEPSPFG-----DVPPKLTSEEIAA        60
tr|S4UD36|S4UD36_RHIMP              ---PPPT------------------------RAHQVDPSPFG-----DVPPKLTSEEIAA        60
tr|S4U8E0|S4U8E0_9ACAR              ---PPPT------------------------RAHQVDPSPFG-----DVPPKLTSEEIAA        60
tr|A0A7D5D0F6|A0A7D5D0F6_RHIDE      ---PPPT------------------------RAHQIDPSPFG-----DVPPKLTSEEIAA        60
tr|J7K2D7|J7K2D7_RHIMP              ---PPPT------------------------RAHQIDPSPFG-----DVPPKLTSEEIAA        60
tr|I0B6E3|I0B6E3_RHIMP              ---PPPT------------------------RAHQIDPSPFG-----DVPPKLTSEEIAA        60
tr|S4U8D6|S4U8D6_RHIMP              ---PPPT------------------------RAHQIDPSPFG-----DVPPKLTSEEIAA        60
tr|S4UD31|S4UD31_RHIMP              ---PPPT------------------------RAHQIDPSPFG-----DVPPKLTSEEIAA        60
tr|Q1AER5|Q1AER5_RHIMP              ---PPPT------------------------RAHQIDPSPFG-----DVPPKLTSEEIAA        60
tr|A0A7D5D0I3|A0A7D5D0I3_RHIAP      ---PPPT------------------------RAHQIDPSPFG-----DVPPKLTSEEIAA        60
tr|J7K8Z9|J7K8Z9_RHIMP              ---PPPT------------------------RAHQIDPSPLG-----DVPPKLTSEEIAA        60
tr|I0B6D6|I0B6D6_RHIMP              ---PPPT------------------------RAHQIDPSPFG-----DVPPKLTSEEIAA        60
tr|S4U9H0|S4U9H0_RHIEV              ---PPPT------------------------RAHQIDPSPFG-----DVPPKLTSEEIAA        60
tr|J7K715|J7K715_RHIMP              ---PPPT------------------------RAHQIDPSPFG-----DVPPKLTSEEIAA        60
tr|S4UAH0|S4UAH0_RHIDE              ---PPPT------------------------RAHQIDPSPFG-----DVPPKLTSEEIAA        60
tr|Q1AER6|Q1AER6_RHIMP              ---PPPT------------------------RAHQIDPSPFG-----DVPPKLTSEEIAA        60
tr|B6CM56|B6CM56_RHIMP              ---PPPT------------------------RAHQIDPSPFG-----DVPPKLTSEEIAA        60
tr|S4UD38|S4UD38_DERRT              SPPAPPT------------------------RAHQINPSPFG-----DVPPKLTSEEIAA        64
tr|A0A097BQ19|A0A097BQ19_9ACAR      SPPAPPT------------------------RAHQINPSPFG-----DVPPKLTSEEIAA        64
tr|K9R5H5|K9R5H5_DERSI              SPPAPPT------------------------RAHQINPSPFG-----DVPPKLTSEEIAA        64
tr|A0A0U2ID78|A0A0U2ID78_9ACAR      SPPAPPT------------------------RAHQMNPSPFG-----EVPPKMTSEEIAA        62
tr|A0A0U2L412|A0A0U2L412_9ACAR      SPPAPPT------------------------RAHQMNPSPFG-----EVPPKMTSEEIAA        62
```

```
tr|A0A0U2TZZ5|A0A0U2TZZ5_9ACAR      SPPAPPT------------------------RAHQMNPSPFG-----EVPPKLTSEEIAA          62
tr|S4U8E3|S4U8E3_HYARU              SPPAPPT------------------------RPHQMNPSPFG-----EVPPKMTSEEIAA          62
tr|A0A0U2URL3|A0A0U2URL3_9ACAR      SPPAPPT------------------------RAHQMNPSPFG-----EVPPKMTSEEIAA          62
tr|A0A0U2T382|A0A0U2T382_9ACAR      SPPAPPT------------------------RAHQMNPSPFG-----EVPPKMTSEEIAA          62
tr|A0A0U2LZ49|A0A0U2LZ49_9ACAR      SPPAPPT------------------------RAHQMNPSPFG-----EVPPKMTSEEIAA          62
tr|A0A0U2T040|A0A0U2T040_9ACAR      SPPAPPT------------------------RAHQMNPSPFG-----EVPPKMTSEEIAA          62
tr|A0A0U2SNR8|A0A0U2SNR8_9ACAR      SPPAPPT------------------------RAHHMNPSPFG-----EVPPKMTSEEIAA          62
tr|A0A0U2K641|A0A0U2K641_9ACAR      SPPAPPT------------------------RAHQMNPSPFG-----GVPPKMTSEEIAA          62
tr|S4UAH3|S4UAH3_9ACAR              SPPAPPT------------------------RAHQMNPSPFG-----EVPPKMTSEEIAA          62
tr|A0A131XM91|A0A131XM91_9ACAR      SPPAPPT------------------------RAHQMNPSPFG-----EVPPKMTSEEIAA          62
tr|A0A077B3N4|A0A077B3N4_HAEFA      TP-TPPT------------------------RAHQINPSPFG-----DVPPKLTSEEIAA          63
tr|S4UF21|S4UF21_9ACAR              TS-TPPT------------------------RAHQINPSPFG-----DVPPKLTSEEIAA          63
tr|A0A7L7T4G3|A0A7L7T4G3_HAELO      TS-TPPT------------------------RAHQINPSPFG-----DVPPKLTSEEIAA          63
tr|E4W3Y9|E4W3Y9_HAELO              TS-TPPT------------------------RAHQINPSPFG-----DVPPKLTSEEIAA          63
tr|A0A293MGQ8|A0A293MGQ8_ORNER      -----------------------------------MSPSPFV-----EVPPKLSSEEIAA          20
tr|S4U9H1|S4U9H1_ORNSA              SPSAPPS------------------------RAHQMSPSPFV-----EVPPKLSSEEIAA          64
tr|S4UD40|S4UD40_ORNMO              SPSAPPS------------------------RAHQMSPSPFV-----EVPPKLSSEEIAA          64
tr|E2IU45|E2IU45_ORNMO              SPSAPPS------------------------RAHQMSPSPLV-----EVPPKLSSEEIAA          64
tr|E2IU46|E2IU46_ORNER              SPSTAPA------------------------RAHQMSPSPFV-----EVPPKLSSEEIAA          64
tr|A0A0D5Y9E2|A0A0D5Y9E2_9ACAR      SPSTPPS------------------------RAHQICPSPFV-----EVPPKLSSEEIAA          63
tr|A0A2R5LKD0|A0A2R5LKD0_9ACAR      SPSTPPS------------------------RAHQICPSPFV-----EVPPKLSSEEIAA          64


tr|B5L666|B5L666_RHIMP              NLTTEVKSVEMHHEALAEAVPGDNVGFNVKNVSVK------------------------E          80
tr|B5L665|B5L665_RHIMP              -------------------------CLNEHNYLTQQEK----------------------          94
tr|A0A6M2D4F4|A0A6M2D4F4_RHIMP      -------------------------CLNEHNYLTQQEK----------------------          96
tr|Q6JIC2|Q6JIC2_ANAMA              SS----------------------------------------------------------          69
tr|R9R561|R9R561_9DIPT              GLYNEIKRLHKR----------KQLPITSSALERMQDSESSGS-----------------          40
tr|S4U8E4|S4U8E4_9DIPT              NITEEIKRLHRR----------KQLTLNTHTMRPMQDSKSSGS-----------------          119
tr|S4UAH5|S4UAH5_CULPP              NITGEIKRLDRR----------KQLTFNH--SERMQDSESSGS-----------------          114
tr|C3RX28|C3RX28_AEDAL              NITEEIKRLHRR----------KQLTFNTGSMERMQDSESSGS-----------------          116
tr|A0A3M7Q970|A0A3M7Q970_BRAPC      RIRHEAKRLIRR----------RQLGVSSVNMLSVEAAE------PVEPVSPLHSPSEPK          140
tr|J7K3S8|J7K3S8_9ACAR              TVHDEMVRLQRR----------RQLCFQQGPPERA---A-----------VLDTLP----          101
tr|J7K8Y7|J7K8Y7_9ACAR              TVHDEMMRLQRR----------RQLCFQQGPPERA---A-----------VLDTLP----          101
tr|J7K9Q0|J7K9Q0_9ACAR              TVHDEMMRPQRR----------RQLCFQQGPPERA---A-----------VLDTLP----          101
tr|J7K8Z4|J7K8Z4_9ACAR              TVHDEMMRLQRR----------RQLCFQQGPPERA---A-----------VLDTLP----          101
tr|J7K706|J7K706_9ACAR              TVHDEMMRLQRR----------RQLCFQQGPPERA---A-----------VLDTLP----          101
tr|A0A0X9PMU2|A0A0X9PMU2_9ACAR      TVHDEMMRLQRR----------RQLCFQQGPPERA---A-----------VLDTLP----          101
tr|J7K9P7|J7K9P7_9ACAR              TVHDEMMRLQRR----------RQLCFQQGPPERA---A-----------VLDTLP----          101
tr|A0A9J6F3J6|A0A9J6F3J6_RHIMP      YVHDEMMRLQHR----------RQLFYQQGYQYCAASPA-----------GPDAMP----          102
tr|A0A9D4Q191|A0A9D4Q191_RHISA      NVHDEMLRLQRR----------RQLCFQEGSPDYA-CPA-----------VLDTWP----          104
tr|A0A131YRI7|A0A131YRI7_RHIAP      NVHDEMLRLQRR----------RQLFFQQGSPDYAASPA-----------VLDTLP----          102
tr|A0A224Z051|A0A224Z051_9ACAR      NVHDEMLRLQRR----------RQLFFQQGSPDYAASPA-----------VFDTWP----          102
tr|A0A443S7G3|A0A443S7G3_9ACAR      TIKEEMKRLQNR----------KQLHYVSSGPANTSSNSD-TTSNECS--STESLPPVLS          109
tr|R9R3J1|R9R3J1_AEDAL              ------------------------------LESGSPSA--------TPPASPTGL-----          54
tr|A0A0B5J531|A0A0B5J531_9ACAR      NIREETRRLQRR----------KQLCFSSPLEAGSSPGTSAGVPPECGLASLTGLSPSSV          115
tr|S4UAF8|S4UAF8_IXOHE              NIREEMRRLQRR----------KQLCFSSPLETGSPSGTLCGVSAECG------------          103
tr|R9R416|R9R416_AEDAL              NIREEMRRLQRR----------KQLTFSSPLESGSPSATP--PAADCGPASPTGLSP---          131
tr|Q1AES0|Q1AES0_IXORI              NIREEMRRLQRR----------KQLCFSSPLESGSPSVTP--PAAECGPASPTGLSP---          110
tr|S4UF09|S4UF09_IXOSC              NIREEMRRLQRR----------KQLCFSSPLESGSPSATP--PAADCGPASPTGLSP---          110
tr|S4U8C9|S4U8C9_IXOSC              NIREEMRRLQRR----------KQLCFSSPLESGSPSATP--PAADCGPASPTGLSP---          110
tr|S4U9E5|S4U9E5_IXOSC              NIREEMRRLQRR----------KQLCFSSPLESGSPSVTP--PAADCGPASPTGLSP---          110
tr|A0A0A7E016|A0A0A7E016_IXOPE      NIREEMRRLQRR----------KQLCFSSPLESGSPSATP--PAAECGPASPTGLSP---          110
tr|Q4VRW2|Q4VRW2_IXOSC              NIREEMRRLQRR----------KQLCFSSPLESGSPSATP--PAADCGPASPTGLSP---          110
tr|A0A0A7DZX9|A0A0A7DZX9_IXOPE      NIREEMRRLQRR----------KQLCFSSPLESGSPSATP--PAAECGPASPTGLSP---          110
tr|W8E787|W8E787_IXOPE              NIREEMRRLQRR----------KQLCFSSPLESGSPSATP--PAAECGPASPTGLSP---          50
tr|W8E0V9|W8E0V9_IXOPE              NIREEMRRLQRR----------KQLCFSSPLESGSPSATP--PAAECGPASPTGLSP---          50
tr|W8E329|W8E329_9ACAR              NIREEMRRLQRR----------KQLCFSSPLESGSPSATP--PAAECGPASPTGLSP---          50
tr|A0A1E1XVC7|A0A1E1XVC7_AMBSC      NIREEMRRLQRR----------KQLCFQGA---ESPPEG---C-------PPVDSPSQ--          99
tr|A0A1E1X1X1|A0A1E1X1X1_9ACAR      NIREEMRRLQRR----------KQLCFQGGAECSSPPEG---C-------SPVGSPSQ--          102
tr|S4U9F0|S4U9F0_AMBAM              NIREEMRRLQRP----------KQLCFQG-AECSSPPEG---C-------SPIGSPSQ--          101
tr|A0A0C9RT91|A0A0C9RT91_AMBAM      NIREEMRRLQRR----------KQLCFQG-AECSSPPEG---C-------SPIGSPSQ--          101
tr|S4U8D1|S4U8D1_AMBAM              NIREEMRRLQRR----------KQLCFQG-AECSSPPEG---C-------SPIGSPSQ--          101
tr|Q1AER9|Q1AER9_AMBAM              NIREEMRRLQRR----------KQLCFQG-AECSSPPEG---C-------SPIGSPSQ--          101
tr|S4UF12|S4UF12_AMBMU              NIREEMRRLQRR----------KQLCFQG-AECNSPPEG---C-------SPVGSPSQ--          101
tr|S4UAG1|S4UAG1_AMBCJ              NIREEMRRLQRR----------KQLCFQG-AECSSPPEG---C-------SPVGSPSQ--          101
tr|S4U8D4|S4U8D4_AMBVA              NIREEMRRLQRR----------KQLCFQG-AECSSPPEG---C-------SPVGSPSQ--          101
tr|A0A7D4XVE8|A0A7D4XVE8_AMBVA      NIREEMRRLQRR----------KQLCFQG-AECSSPPEG---C-------SPVGSPSQ--          101
tr|B7SCS6|B7SCS6_AMBHE              NIREEMRRLQRR----------KQLCFQG-AECSSPPEG---C-------SPVGSPSQ--          101
tr|Q1AER3|Q1AER3_RHISA              NIREEMRRLQRR----------KQLCFQGTDPE-----------------------CQ--          85
tr|A0A097BQ10|A0A097BQ10_RHIHE      NIREEMRRLQRR----------KQLCFQATDPE-----------------------CQ--          85
tr|A0A0H3WJP2|A0A0H3WJP2_RHIHE      NIREEMRRLQRR----------KQLCFQATDPE-----------------------CQ--          85
tr|I0B6E2|I0B6E2_RHIMP              NIREEMRRLQRR----------KQLCFQGADPE-----------------------SQ--          85
tr|I3XPN4|I3XPN4_RHIMP              NIREEMRRLQRR----------KQLCFQGADPE-----------------------SQ--          85
tr|I0B6D9|I0B6D9_RHIMP              NIREEMRRLQRR----------EQLCFQGADPE-----------------------SQ--          85
tr|I0B6D7|I0B6D7_RHIMP              NIREEMRRLQRR----------KQLCFQGADPE-----------------------SQ--          85
tr|I0B6E5|I0B6E5_RHIMP              NIREEMRRLQRR----------KQLCFQGADPE-----------------------SQ--          85
tr|S4UD36|S4UD36_RHIMP              NIREEMRRLQRR----------KQLCFQGADPE-----------------------SQ--          85
tr|S4U8E0|S4U8E0_9ACAR              NIREEMRRLQRR----------KQLCFQGADPE-----------------------SQ--          85
tr|A0A7D5D0F6|A0A7D5D0F6_RHIDE      NIREEMRRLQRR----------KQLCFQGTDAE-----------------------SQ--          85
tr|J7K2D7|J7K2D7_RHIMP              NIREEMRRLQRR----------KQLCFQGADPE-----------------------SQ--          85
tr|I0B6E3|I0B6E3_RHIMP              NIREEMRRLQRR----------KQLCFQGADPE-----------------------SQ--          85
tr|S4U8D6|S4U8D6_RHIMP              NIREEMRRLQRR----------KQLCFQGRDPE-----------------------SQ--          85
tr|S4UD31|S4UD31_RHIMP              NIREEMRRLQRR----------KQLCFQGRDPE-----------------------SQ--          85
tr|Q1AER5|Q1AER5_RHIMP              NIREEMRRLQRR----------KQLCFQGRDPE-----------------------SQ--          85
tr|A0A7D5D0I3|A0A7D5D0I3_RHIAP      SIREEMRRLQRR----------KQLCFQGADPE-----------------------SQ--          85
tr|J7K8Z9|J7K8Z9_RHIMP              NIREEMRRLQRR----------KQLCFQGADPE-----------------------SQ--          85
tr|I0B6D6|I0B6D6_RHIMP              NIREEMRRLQRR----------KQLCFQGADPE-----------------------SQ--          85
tr|S4U9H0|S4U9H0_RHIEV              NIREEMRRLQRR----------KQLCFQGADPE-----------------------SQ--          85
tr|J7K715|J7K715_RHIMP              NIREEMRRLQRR----------KQLCFQGADPE-----------------------SQ--          85
tr|S4UAH0|S4UAH0_RHIDE              NIREEMRRLQRR----------KQLCFQGADPE-----------------------SQ--          85
tr|Q1AER6|Q1AER6_RHIMP              NIREEMRRLQRR----------KQLCFQGADPE-----------------------SQ--          85
tr|B6CM56|B6CM56_RHIMP              NIREEMRRLQRR----------KQLCFQGADPE-----------------------SQ--          85
tr|S4UD38|S4UD38_DERRT              NIREEMRRLQRR----------KQLCFQGADPG------------------------Q--          88
```

```
tr|A0A097BQ19|A0A097BQ19_9ACAR      NIREEMRRLQRR----------KQLCFQGTDPE-----------------------SQ--     89
tr|K9R5H5|K9R5H5_DERSI              NIREEMRRLQRR----------KQLCFQGTDPE-----------------------SQ--     89
tr|A0A0U2ID78|A0A0U2ID78_9ACAR      NIREEMRRLQRR----------KQLCFQGTDPE-----------------------SQ--     87
tr|A0A0U2L412|A0A0U2L412_9ACAR      NIREEMRRLQRR----------KQLCFQGTDPE-----------------------SQ--     87
tr|A0A0U2TZZ5|A0A0U2TZZ5_9ACAR      NIREEMRRLQRR----------KQLCFQGTDPE-----------------------SQ--     87
tr|S4U8E3|S4U8E3_HYARU              NIREEMRRLQRR----------KQLCFQGTDPE-----------------------SQ--     87
tr|A0A0U2URL3|A0A0U2URL3_9ACAR      NIREEMRWLQRR----------KQLCFQGTDPE-----------------------SQ--     87
tr|A0A0U2T382|A0A0U2T382_9ACAR      NIREEMRRLQRR----------KQLCFQGTDPE-----------------------SQ--     87
tr|A0A0U2LZ49|A0A0U2LZ49_9ACAR      NIREEMRRLQRR----------KQLCFQGTDPE-----------------------SQ--     87
tr|A0A0U2T040|A0A0U2T040_9ACAR      NIREEMRRLQRR----------KQLCFQGTDPE-----------------------SQ--     87
tr|A0A0U2SNR8|A0A0U2SNR8_9ACAR      NIREEMRRLQRR----------KQLCFQGTDPE-----------------------SQ--     87
tr|A0A0U2K641|A0A0U2K641_9ACAR      NIREEMRRLQRR----------KQLCFQGTDPE-----------------------SQ--     87
tr|S4UAH3|S4UAH3_9ACAR              NIREEMRRLQRR----------KQLCFQGTDPE-----------------------SQ--     87
tr|A0A131XM91|A0A131XM91_9ACAR      NIREEMRRLQRR----------KQLCFQGTDPE-----------------------SQ--     87
tr|A0A077B3N4|A0A077B3N4_HAEFA      NIREEMRRLQRR----------KQLCFQAGAEGGTSSGG---D-------SPIGSPSSSS     103
tr|S4UF21|S4UF21_9ACAR              NIREEMRRLQRR----------KQLCFQGEGAS---SGG---D-------SPVGSPGCS-     99
tr|A0A7L7T4G3|A0A7L7T4G3_HAELO      NIREEMRRLQRR----------KQLCFQGEGGASSSGGD---S-------SPLGSPSCPS     103
tr|E4W3Y9|E4W3Y9_HAELO              NIREEMRRLQRR----------KQLCFQGEGGASSSGGD---S-------SPLGSPSCPS     103
tr|A0A293MGQ8|A0A293MGQ8_ORNER      NIREEMRRLQRR----------KHLCFTALDSSPQI-SS---T-------DSCSGPSSPT     59
tr|S4U9H1|S4U9H1_ORNSA              NIREEMRRLQRR----------KQLYFPSLGSPPQG-SS---S-------SS-----STD     98
tr|S4UD40|S4UD40_ORNMO              NIREEMRRLQRR----------KQLYFPSLDSPPQG-TS---G--------S-----SAD     97
tr|E2IU45|E2IU45_ORNMO              NIREEMRRLQRR----------KQLYFPSLDSPPQG-TS---G--------S-----SAD     97
tr|E2IU46|E2IU46_ORNER              NIREEMRRLQRR----------KQLCFTALDSSPQN-SS---T-------DSCSGPSSPT     103
tr|A0A0D5Y9E2|A0A0D5Y9E2_9ACAR      NIREEMKRLQRR----------KQLCFPTLDSSPQS-AD---S-----------LPSSP-     97
tr|A0A2R5LKD0|A0A2R5LKD0_9ACAR      NIREEMKRLQRR----------KQLCFPTLDSSPQS-AD---S-----------LPSSP-     98


tr|B5L666|B5L666_RHIMP              LRRGYVCGDSKDTPPKSTEEFTAQV------------IVLNHPGQIANGYTPVLDCHTAH     128
tr|B5L665|B5L665_RHIMP              ------------------------------------------------------------     94
tr|A0A6M2D4F4|A0A6M2D4F4_RHIMP      ------------------------------------------------------------     96
tr|Q6JIC2|Q6JIC2_ANAMA              ------------KETSYVRGYDKSIA---------TIDVSVPANFSKSGYTFAFSKNLIT     108
tr|R9R561|R9R561_9DIPT              -EMGPESPHRPDSPQNLMRHGEKALFTF--------------------------------     67
tr|S4U8E4|S4U8E4_9DIPT              -EMSPESSRRPKSTLSMVKNPEKALF----------------------------------     144
tr|S4UAH5|S4UAH5_CULPP              -EIGPVSPRRPDSPPSMVKNLIRQLFTFKQVQMICERMLKEREDSLREQYEALLTTKVCE     173
tr|C3RX28|C3RX28_AEDAL              -EMGPDSPRRPDSPPSMVKHPEKALFTFKQVQMICERMLKEREDALREQYDAVLTNKLAE     175
tr|A0A3M7Q970|A0A3M7Q970_BRAPC      SSRLKALLSHNDVP----------LFSISQVNQICDKMMKEREQFIREQYDKILADKLTE     190
tr|J7K3S8|J7K3S8_9ACAR              -----LQGEKADQP----------VFTFRQVGLIIDRMVSEREKQLRGVYDAVLSAKLAE     146
tr|J7K8Y7|J7K8Y7_9ACAR              -----LQGEKADQP----------VFTFRQVGLIIDRMVSEREKQLRGVYDAVLSAKLAE     146
tr|J7K9Q0|J7K9Q0_9ACAR              -----LQGEKADQP----------VFTFRQVGLIIDRMVSEREKQLRGVYDAVLSAKLAE     146
tr|J7K8Z4|J7K8Z4_9ACAR              -----LQGEKADQP----------VFTFRQVGLIIDRMVSEREKQLRGVYDAVLSAKLAE     146
tr|J7K706|J7K706_9ACAR              -----LQGEKADQP----------VFTFRQVGLIIDRMVSEREKQLRGVYDAVLPAKLAE     146
tr|A0A0X9PMU2|A0A0X9PMU2_9ACAR      -----LQGEKADQP----------VFTFRQVGLIIDRMVSEREKQLRGVYDAVLSAKLAE     146
tr|J7K9P7|J7K9P7_9ACAR              -----LQGEKADQP----------VFTFRQVGLIIDRMVSEREKQLRGVYDAVLSAKLAE     146
tr|A0A9J6F3J6|A0A9J6F3J6_RHIMP      -----PQGAKTEQP----------AFTFRQVGMIVERMVSEREKQLREVYDRVLSAKLAE     147
tr|A0A9D4Q191|A0A9D4Q191_RHISA      -----PQGAKADQP----------IFTFRQVGLIVERMVGEREKQLREVYDSVLSAKLAE     149
tr|A0A131YRI7|A0A131YRI7_RHIAP      -----PQGAKADQP----------VFTFRQVGLIVERMVSEREKQLREVYDSVLSAKLAE     147
tr|A0A224Z051|A0A224Z051_9ACAR      -----PQGAKADQP----------VFTFRQVGLIVERMVSEREKQLREVYDSVLSAKLAE     147
tr|A0A443S7G3|A0A443S7G3_9ACAR      SNVGLMSPCRRDQP----------LFTFRQVGIICERLMKERESQMREEYDSVLNTKLAE     159
tr|R9R3J1|R9R3J1_AEDAL              SPGGLLSPVRRDQP----------LFTFRQVGLICERMMKERESQIRDEYDHVLSAKLAE     104
tr|A0A0B5J531|A0A0B5J531_9ACAR      GGAGLLSPMRRDQP----------LFTFRQVGLICERMMKERESQIREEYDHVLSAKLAE     165
tr|S4UAF8|S4UAF8_IXOHE              ----LLSPMRRDQP----------LFTFRQVGLICERMMKERESQIREEYDRVLSAKLAE     149
tr|R9R416|R9R416_AEDAL              --GGLLSPVRRDQPPSMVKHPEKALFTFRQVGLICERMMKERESQIRDQYDAVLSAKLAE     189
tr|Q1AES0|Q1AES0_IXORI              --GGLLSPVRRDQP----------LFTFRQVGLICERMMKERESQIRDEYDHVLSAKLAE     158
tr|S4UF09|S4UF09_IXOSC              --GGLLSPVRRDQP----------LFTFRQVGLICERMMKERESQIRDEYDHVLSAKLAE     158
tr|S4U8C9|S4U8C9_IXOSC              --GGLLSPVRRDQP----------LFTFRQVGLICERMMKERESQIRDEYDHVLSAKLAE     158
tr|S4U9E5|S4U9E5_IXOSC              --GGLLSPVRRDQP----------LFTFRQVGLICERMMKERESQIRDEYDHVLSAKLAE     158
tr|A0A0A7E016|A0A0A7E016_IXOPE      --GGLLSPVRRDQP----------LFTFRQVGLICERMMKERESQIRDEYDNVLSAKLAE     158
tr|Q4VRW2|Q4VRW2_IXOSC              --GGLLSPVRRDQP----------LFTFRQVGLICERMMKERESQIRDEYDHVLSAKLAE     158
tr|A0A0A7DZX9|A0A0A7DZX9_IXOPE      --GGLLSPVRRDQP----------LFTFRQVGLICERMMKERESQIRDEYDHVLSAKLAE     158
tr|W8E787|W8E787_IXOPE              --GGLLSPVRRDQP----------LFTFRQVGLICERMMKERESQIRDEYDH--------     90
tr|W8E0V9|W8E0V9_IXOPE              --GGLLSPVRRDQP----------LFTFRQVGLICERMMKERESQIRDEYDHV-------     91
tr|W8E329|W8E329_9ACAR              --GGLLSPVRRDQP----------LFTFRQVGLICERMMKERESQIRDEYDHV-------     91
tr|A0A1E1XVC7|A0A1E1XVC7_AMBSC      HTGGLLSPVRRDQP----------LFTFRQVGLICERMMKERESQIREEYDHVLSTKLAE     149
tr|A0A1E1X1X1|A0A1E1X1X1_9ACAR      HTGGLLSPVRRDQP----------LFTFRQVGLICERMMKERESQIREEYDQVLSTKLAE     152
tr|S4U9F0|S4U9F0_AMBAM              HTGGLLSPVRRDQP----------LFTFRQVGLICERMMKERESQIREEYDHVLSTKLAE     151
tr|A0A0C9RT91|A0A0C9RT91_AMBAM      HTGGLLSPVRRDQP----------LFTFRQVGLICERMMKERESQIREEYDHVLSTKLAE     151
tr|S4U8D1|S4U8D1_AMBAM              HTGGLLSPVRRDQP----------LFTFRQVGLICERMMKERESQIREEYDHVLSTKLAE     151
tr|Q1AER9|Q1AER9_AMBAM              HTGGLLSPVRRDQP----------LFTFRQVGLICERMMKERESQIREEYDHVLSTKLAE     151
tr|S4UF12|S4UF12_AMBMU              HTGGLLSPVRRDQP----------LFTFRQVGLICERMMKERESQIREEYDHVLSTKLAE     151
tr|S4UAG1|S4UAG1_AMBCJ              HTGGLLSPVRRDQP----------LFTFRQVGLICERMMKERESQIREEYDHVLSTKLAE     151
tr|S4U8D4|S4U8D4_AMBVA              HTSGLLSPVRRDQP----------LFTFRQVGLICERMMKERESQIREEYDHVLSTKLAE     151
tr|A0A7D4XVE8|A0A7D4XVE8_AMBVA      HTSGLLSPVRRDQP----------LFTFRQVGLICERMMKERESQIREEYDHVLSTKLAE     151
tr|B7SCS6|B7SCS6_AMBHE              HTSGLLSPVRRDQP----------LFTFRQVGLICERMMKERESQIREEYDHVLSTKLAE     151
tr|Q1AER3|Q1AER3_RHISA              PTSGLSSPVRRDQP----------LFTFRQVGLICERMMKERESKIREEYDHVLSTKLAE     135
tr|A0A097BQ10|A0A097BQ10_RHIHE      QTSGLSSPVRRDQP----------LFTSRQVGLICERMMKERESKIREEYDHVLSTKLAE     135
tr|A0A0H3WJP2|A0A0H3WJP2_RHIHE      QTSGLSSPVRRDQP----------LFTFRQVGLICERMMKERESKIREEYDHVLSTKLAE     135
tr|I0B6E2|I0B6E2_RHIMP              HTSGLSSPVHRDQP----------PFTFRQVGLICERMMKERESKIREEYDHVLSTKLAE     135
tr|I3XPN4|I3XPN4_RHIMP              HTSGLSSPVHRDQP----------LFTFRQVGLICERMMKERESKIREEYDHALSTKLAE     135
tr|I0B6D9|I0B6D9_RHIMP              HTSGLSSPVHRDQP----------LFTFRQVGLICERMMKERESKIREEYDHVLSTKLAE     135
tr|I0B6D7|I0B6D7_RHIMP              HTSGLSSSVHRDQP----------LFTFRQVGLICERMMKERESKIREEYDHVLSTKLAE     135
tr|I0B6E5|I0B6E5_RHIMP              HTSGLSSPVHRDQP----------LFTFRQVGLICERMMKERESKIREEYDHVLSTKLAE     135
tr|S4UD36|S4UD36_RHIMP              HTSGLSSPVHRDQP----------LFTFRQVGLICERMMKERESKIREEYDHVLSTKLAE     135
tr|S4U8E0|S4U8E0_9ACAR              HTSGLSSPVHRDQP----------LFTFRQVGLICERMMKERESKIREEYDHVLSTKLAE     135
tr|A0A7D5D0F6|A0A7D5D0F6_RHIDE      HTSGLSSPVRRDQP----------LFTFRQVGLICERMMKERESKIREEYDHVLSTKLAE     135
tr|J7K2D7|J7K2D7_RHIMP              HTSGLSSPVHRDQP----------LFTFRQVGLICERMMKERESKIREEYDHVLSTKLAE     135
tr|I0B6E3|I0B6E3_RHIMP              HTSGLSSPVHRDQP----------LFTFRQVGLICERMMKERESKIREEYDHVLSTKLAE     135
tr|S4U8D6|S4U8D6_RHIMP              HTSGLSSPVHRDQP----------LFTFRQVGLICERMMKERESKIREEYDHVLSTKLAE     135
tr|S4UD31|S4UD31_RHIMP              HTSGLSSPVHRDQP----------LFTFRQVGLICERMMKERESKIREEYDHVLSTKLAE     135
tr|Q1AER5|Q1AER5_RHIMP              HTSGLSSPVHRDQP----------LFTFRQVGLICERMMKERESKIREEYDHVLSTKLAE     135
tr|A0A7D5D0I3|A0A7D5D0I3_RHIAP      HTSGLSSPVHRDQP----------LFTFRQVGLICERMMKERESKIREEYDHVLSTKLAE     135
tr|J7K8Z9|J7K8Z9_RHIMP              HTSGLSSPVHRDQP----------LFTFRQVGLICERMMKERESKIREEYDHVLSTKLAE     135
tr|I0B6D6|I0B6D6_RHIMP              HTSGLSSPVHRDQP----------LFTFRQVGLICERMMREReskIREEYDHVLSTKLAE     135
tr|S4U9H0|S4U9H0_RHIEV              HTSGLSSPVRRDQP----------LFTFRQVGLICERMMKERESKIREEYDHVLSTKLAE     135
tr|J7K715|J7K715_RHIMP              HTSGLSSPVHRDQP----------LFTFRQVGLICERMMKERESKIREEYDRVLSTKLAE     135
```

```
tr|S4UAH0|S4UAH0_RHIDE              HTSGLSSPVHRDQP----------LFTFRQVGLICERMMKERESKIREEYDHVLSTKLAE        135
tr|Q1AER6|Q1AER6_RHIMP              HTSGLSSPVHRDQP----------LFTFRQVGLICERMMKERESKIREEYDHVLSTKLAE        135
tr|B6CM56|B6CM56_RHIMP              HTSGLSSPVHRDQP----------LFTFRQVGLICERMMKERESKIREEYDHVLSTKLAE        135
tr|S4UD38|S4UD38_DERRT              PAGGLSSPVRRDQP----------LFTFRQVGLICERMMKERESQIREQYDHVLSTKLAE        138
tr|A0A097BQ19|A0A097BQ19_9ACAR      PTSGLLSPVRRDQP----------LFTFRQVGLICERMMKERESQIREEYDHVLSTKLAE        139
tr|K9R5H5|K9R5H5_DERSI              PTSGLLSPVRRDQP----------LFTFRQVGLICERMMKERESQIREEYDHVLSTKLAE        139
tr|A0A0U2ID78|A0A0U2ID78_9ACAR      QTSGLLSPVRRDQP----------LFTFRQVGLIREQMMKERESQIREEYVHVLSTKLAE        137
tr|A0A0U2L412|A0A0U2L412_9ACAR      QTSGLLSPVRRDQP----------LFTFRQVGLICERMMKERESQIREEYDHVLSTKLAE        137
tr|A0A0U2TZZ5|A0A0U2TZZ5_9ACAR      QTSGLLSPVHRDQP----------LFTFRQVGLICERMMKERESQIREEYDHVLSTKLAE        137
tr|S4U8E3|S4U8E3_HYARU              QTSGLLSPVRRDQP----------LFTFRQVGLICERMMKERESQIREEYDHVLSTKLAE        137
tr|A0A0U2URL3|A0A0U2URL3_9ACAR      QTSGLLSPVRRDQP----------LFTFRQVGLICERMMKERESQIREEYDHVLSTKLAE        137
tr|A0A0U2T382|A0A0U2T382_9ACAR      QTSGLLSPVRRDQP----------LFTFRQVGLICERMMKERESQIREEYDHVLSTKLAE        137
tr|A0A0U2LZ49|A0A0U2LZ49_9ACAR      QTSGLLSPVRRDQP----------LFTFRQVGLICERMMKERESQIREEYDHVLSTKLAE        137
tr|A0A0U2T040|A0A0U2T040_9ACAR      QTSGLLSPVRRDQP----------LFTFRQVGLICERMMKERESQIREEYDHVLSTKLAE        137
tr|A0A0U2SNR8|A0A0U2SNR8_9ACAR      QTSGLLSPVRRDQP----------LFTFRQVGLICERMMKERESQIREEYDHVLSTKLAE        137
tr|A0A0U2K641|A0A0U2K641_9ACAR      QTSGLLSPVRRDQP----------LFTFRQVGLICERMMKERESQIREEYDHVLSTKLAE        137
tr|S4UAH3|S4UAH3_9ACAR              QTSGLLSPVRRDQP----------LFTFRQVGLICERMMKERESQIREEYDHVLSTKLAE        137
tr|A0A131XM91|A0A131XM91_9ACAR      QTSGLLSPVRRDQP----------LFTFRQVGLICERMMKERESQIREEYDHVLSTKLAE        137
tr|A0A077B3N4|A0A077B3N4_HAEFA      QAGGLLSPVRRDQP----------LFTFRQVGLICERMMKERESQIREEYDHVLSTKLAE        153
tr|S4UF21|S4UF21_9ACAR              SSQGLLSPVRRDQP----------LFTFRQVGLICERMMKERESQIREEYDHVLSTKLAE        149
tr|A0A7L7T4G3|A0A7L7T4G3_HAELO      SSQGLLSPVRRDQP----------LFTFRQVGLICERMMKERESQIREEYDHVLSTKLAE        153
tr|E4W3Y9|E4W3Y9_HAELO              SSQGLLSPVRRDQP----------LFTFRQVGLICERMMKERES----------------        137
tr|A0A293MGQ8|A0A293MGQ8_ORNER      GASGLLSPVRRDQP----------LFTFRQVGLICERMMKERECQIREQYDQVLSTKLAE        109
tr|S4U9H1|S4U9H1_ORNSA              SPTGLLSPVRRDQP----------LFTFRQVGLICERMMKERESQIREQYDQVLSTKLAE        148
tr|S4UD40|S4UD40_ORNMO              SPTGLLSPVRRDQP----------LFTFRQVGLICERMMKERESQIREQYDQVLSTKLAE        147
tr|E2IU45|E2IU45_ORNMO              SPTGLLSPVRRDQP----------LFTFRQVGLICERMMKERESQIREQYDQVLSTKLAE        147
tr|E2IU46|E2IU46_ORNER              GASGLLSPVRRDQP----------LFTFRQVGLICERMMKERECQIREQYDQVLSTKLAE        153
tr|A0A0D5Y9E2|A0A0D5Y9E2_9ACAR      -TGGLLSPVRRDQP----------LFTFRQVGLICERMMKERECQIREQYDQVLSTKLAE        146
tr|A0A2R5LKD0|A0A2R5LKD0_9ACAR      -TGGLLSPVRRDQP----------LFTFRQVGLICERMMKERECQIREQYDQVLSTKLAE        147


tr|B5L666|B5L666_RHIMP              IACKF-----REIKEKCDR-----RSGKKLEDNPKFIKSGDAAIIDLVPSKPM-------        171
tr|B5L665|B5L665_RHIMP              ------------------------------------------------------------        94
tr|A0A6M2D4F4|A0A6M2D4F4_RHIMP      ------------------------------------------------------------        96
tr|Q6JIC2|Q6JIC2_ANAMA              SFDGAVGYSLGGARVELEASYRRFATL----ADGQYAKSGAESLAAITRDANITETNYFV        164
tr|R9R561|R9R561_9DIPT              ------------------------------------------------------------        67
tr|S4U8E4|S4U8E4_9DIPT              ------------------------------------------------------------        144
tr|S4UAH5|S4UAH5_CULPP              QYDAFVKFTYDQIERRYEA-------------EPSHLS----------------------        198
tr|C3RX28|C3RX28_AEDAL              QYDAFVKFTYDQIQRRYEA-------------APSYLS----------------------        200
tr|A0A3M7Q970|A0A3M7Q970_BRAPC      QYDSFVKFTHEQIQRRFET------------SQCSYVS----------------------        216
tr|J7K3S8|J7K3S8_9ACAR              QYDAFVKFTYDQ------------------------------------------------        158
tr|J7K8Y7|J7K8Y7_9ACAR              QYGAFVKFTYDQ------------------------------------------------        158
tr|J7K9Q0|J7K9Q0_9ACAR              QYDAFVKFTYDQ------------------------------------------------        158
tr|J7K8Z4|J7K8Z4_9ACAR              QYDAFVKFTYDQ------------------------------------------------        158
tr|J7K706|J7K706_9ACAR              QYDAFVKFTYDQ------------------------------------------------        158
tr|A0A0X9PMU2|A0A0X9PMU2_9ACAR      QYDAFVKFTHDQIRRRYDG------------VTPSYLS----------------------        172
tr|J7K9P7|J7K9P7_9ACAR              QYDAFVKFTYDQ------------------------------------------------        158
tr|A0A9J6F3J6|A0A9J6F3J6_RHIMP      QYDAFVKFTHDQIQRRYGS------------VRPSYLS----------------------        173
tr|A0A9D4Q191|A0A9D4Q191_RHISA      QYDAFVKFTHDQIQRRYDS------------VTPSYLS----------------------        175
tr|A0A131YRI7|A0A131YRI7_RHIAP      QYDAFVKFTHDQIQRRYDS------------VMPSYLS----------------------        173
tr|A0A224Z051|A0A224Z051_9ACAR      QYDAFVKFTHDQIQRRYDS------------VMPSYLS----------------------        173
tr|A0A443S7G3|A0A443S7G3_9ACAR      QYDTFVKFTYDQIQKRFETGTLPSCKLEHLDF----------------------------        191
tr|R9R3J1|R9R3J1_AEDAL              QYDTFVKFTYDQIQKRFEG------------ATPSYLSGGGS-HKPFGSPSSPS------        145
tr|A0A0B5J531|A0A0B5J531_9ACAR      QYGTFVKFTYDQIQKRFEG------------ATPSYLS----------------------        191
tr|S4UAF8|S4UAF8_IXOHE              QYDTFVKFTYDQIQKRFEG------------ATPSYLS----------------------        175
tr|R9R416|R9R416_AEDAL              QYDTFVKFTYDQIQKRFEG------------AAPSYLS----------------------        215
tr|Q1AES0|Q1AES0_IXORI              QYDTFVKFTYDQIQKRFEG------------ATPSYLS----------------------        184
tr|S4UF09|S4UF09_IXOSC              QYDTFVKFTYDQIQKRFEG------------ATPSYLS----------------------        184
tr|S4U8C9|S4U8C9_IXOSC              QYDTFVKFTYDQIQKRFEG------------ATPSYLS----------------------        184
tr|S4U9E5|S4U9E5_IXOSC              QYDTFVKFTYDQIQKRFEG------------ATPSYLS----------------------        184
tr|A0A0A7E016|A0A0A7E016_IXOPE      QYDTFVKFTYDQIQKRFEG------------ATPSYLS----------------------        184
tr|Q4VRW2|Q4VRW2_IXOSC              QYDTFVKFTYDQIQKRFEG------------ATPSYLS----------------------        184
tr|A0A0A7DZX9|A0A0A7DZX9_IXOPE      QYDTFVKFTYDQIQKRFEG------------ATPSYLS----------------------        184
tr|W8E787|W8E787_IXOPE              ------------------------------------------------------------        90
tr|W8E0V9|W8E0V9_IXOPE              ------------------------------------------------------------        91
tr|W8E329|W8E329_9ACAR              ------------------------------------------------------------        91
tr|A0A1E1XVC7|A0A1E1XVC7_AMBSC      QYDTFVKFTYDQIQKRFEG------------ATPSYLS----------------------        175
tr|A0A1E1X1X1|A0A1E1X1X1_9ACAR      QYDTFVKFTYDQIQKRFEG------------ATPSYLS----------------------        178
tr|S4U9F0|S4U9F0_AMBAM              QYDTFVKFTYDQ------------------------------------------------        163
tr|A0A0C9RT91|A0A0C9RT91_AMBAM      QYDTFVKFTYDQIQKRFEG------------ATPSYLS----------------------        177
tr|S4U8D1|S4U8D1_AMBAM              QYDTFVKFTYDQII----------------------------------------------        165
tr|Q1AER9|Q1AER9_AMBAM              QYDTFVKFTYDQ------------------------------------------------        163
tr|S4UF12|S4UF12_AMBMU              QYDTFVKFTYDQ------------------------------------------------        163
tr|S4UAG1|S4UAG1_AMBCJ              QYDTFVKFTYDQ------------------------------------------------        163
tr|S4U8D4|S4U8D4_AMBVA              QYDTFVKFTYDQ------------------------------------------------        163
tr|A0A7D4XVE8|A0A7D4XVE8_AMBVA      QYDTFVKFTYDQIQKRFEG------------ATPSYLS----------------------        177
tr|B7SCS6|B7SCS6_AMBHE              QYDTFVKFTYDQ------------------------------------------------        163
tr|Q1AER3|Q1AER3_RHISA              QYDTFVKFTYDQIQKRFEG------------ATPSYLS----------------------        161
tr|A0A097BQ10|A0A097BQ10_RHIHE      QYDTFVKFTYDQIQKRFEG------------ATPSYLS----------------------        161
tr|A0A0H3WJP2|A0A0H3WJP2_RHIHE      QYDTFVKFTYDQIQKRFEG------------ATPSYLS----------------------        161
tr|I0B6E2|I0B6E2_RHIMP              QYDTFVKFTYDQIQKRFEG------------ATPSYLS----------------------        161
tr|I3XPN4|I3XPN4_RHIMP              QYDTFVKFTYDQIQKRFEG------------ATPSYLS----------------------        161
tr|I0B6D9|I0B6D9_RHIMP              QYDTFVKFTYDQIQKRFEG------------ATPSYLS----------------------        161
tr|I0B6D7|I0B6D7_RHIMP              QYDTFVKFTYDQIQKRFEG------------ATPSYLS----------------------        161
tr|I0B6E5|I0B6E5_RHIMP              QYDTFVKFTYDQIQKRFEG------------ATPSYLS----------------------        161
tr|S4UD36|S4UD36_RHIMP              QYDTFVKFTYDQK-----------------------------------------------        148
tr|S4U8E0|S4U8E0_9ACAR              QYDTFVKFTYDQ------------------------------------------------        147
tr|A0A7D5D0F6|A0A7D5D0F6_RHIDE      QYDTFVKFTYDQIQKRFEG------------ATPSYLS----------------------        161
tr|J7K2D7|J7K2D7_RHIMP              QYDTFVKFTYDQIQKRFEG------------ATPSYLS----------------------        161
tr|I0B6E3|I0B6E3_RHIMP              QYDTFVKFTYDQIQKRFEG------------ATPSYLS----------------------        161
tr|S4U8D6|S4U8D6_RHIMP              QYDTFVKFTYDQ------------------------------------------------        147
tr|S4UD31|S4UD31_RHIMP              QYDTFVKFTYDQ------------------------------------------------        147
tr|Q1AER5|Q1AER5_RHIMP              QYDTFVKFTYDQ------------------------------------------------        147
tr|A0A7D5D0I3|A0A7D5D0I3_RHIAP      QYDTFVKFTYDQIQKRFEG------------ATPSYLS----------------------        161
```

```
tr|J7K8Z9|J7K8Z9_RHIMP              QYDTFVKFTYDQIQKRFEG------------ATPSYLS----------------------      161
tr|I0B6D6|I0B6D6_RHIMP              QYDTFVKFTYDQIQKRFEG------------ATPSYLS----------------------      161
tr|S4U9H0|S4U9H0_RHIEV              QYDTFVKFTYDQIQKRFEG------------ATPSYLS----------------------      161
tr|J7K715|J7K715_RHIMP              QYDTFVKFTYDQIQKRFEG------------ATPSYLS----------------------      161
tr|S4UAH0|S4UAH0_RHIDE              QYDTFVKFTYDQ------------------------------------------------      147
tr|Q1AER6|Q1AER6_RHIMP              QYDTFVKFTYDQ------------------------------------------------      147
tr|B6CM56|B6CM56_RHIMP              QYDTFVKFTYDQIQKRFEG------------ATPSYLS----------------------      161
tr|S4UD38|S4UD38_DERRT              QYDTFVKFTYDQ------------------------------------------------      150
tr|A0A097BQ19|A0A097BQ19_9ACAR      QYDTFVKFTYDQIQKRFEG------------ATPSYLS----------------------      165
tr|K9R5H5|K9R5H5_DERSI              QYDTFVKFTYDQIQKRFEG------------ATPSYLS----------------------      165
tr|A0A0U2ID78|A0A0U2ID78_9ACAR      QYDTFVKFTYDQIQKRFEG------------ATPSYLS----------------------      163
tr|A0A0U2L412|A0A0U2L412_9ACAR      QYDTFVKFTYDQIQKRFEG------------ATPSYLS----------------------      163
tr|A0A0U2TZZ5|A0A0U2TZZ5_9ACAR      QYDTFVKFTYDQIQKRFEG------------ATPSYLS----------------------      163
tr|S4U8E3|S4U8E3_HYARU              QYDTFVKFTYDQ------------------------------------------------      149
tr|A0A0U2URL3|A0A0U2URL3_9ACAR      QYDTFVKFTYDQIQKRFEG------------ATPSYLS----------------------      163
tr|A0A0U2T382|A0A0U2T382_9ACAR      QYDTFVKFTYDQIQKRFED------------ATPSYLS----------------------      163
tr|A0A0U2LZ49|A0A0U2LZ49_9ACAR      QYDTFVKFTYVQIQKRFEG------------ATPSYLS----------------------      163
tr|A0A0U2T040|A0A0U2T040_9ACAR      QYDTFVKFTYDQIQKRFEG------------ATPSYLS----------------------      163
tr|A0A0U2SNR8|A0A0U2SNR8_9ACAR      QYDTFVKFTYDQIQKRFEG------------ATPSYLS----------------------      163
tr|A0A0U2K641|A0A0U2K641_9ACAR      QYDTFVKFTYDQIQKRFEG------------ATPSYLS----------------------      163
tr|S4UAH3|S4UAH3_9ACAR              QYDTFVKFTYDQIQKRFEG------------ATPSYLS----------------------      163
tr|A0A131XM91|A0A131XM91_9ACAR      QYDTFVKFTYDQIQKRFEG------------ATPSYLS----------------------      163
tr|A0A077B3N4|A0A077B3N4_HAEFA      QYDTFVKFTYDQIQKRFEG------------ATPSYLS----------------------      179
tr|S4UF21|S4UF21_9ACAR              QYDTFVKFTYDQIQKRFEG------------ATPSYLS----------------------      175
tr|A0A7L7T4G3|A0A7L7T4G3_HAELO      QYDTFVKFTYDQIQKRFEG------------ATPSYLS----------------------      179
tr|E4W3Y9|E4W3Y9_HAELO              ------------------------------------------------------------      137
tr|A0A293MGQ8|A0A293MGQ8_ORNER      QYDTFVKFTYDQIQKKFEGATYDQIQKKFEGATPSYLS----------------------      147
tr|S4U9H1|S4U9H1_ORNSA              QYDTFVKFTYDQIQKKFEG------------ATPSYLS----------------------      174
tr|S4UD40|S4UD40_ORNMO              QYDTFVKFTYDQIQKKFEG------------ATPSYLS----------------------      173
tr|E2IU45|E2IU45_ORNMO              QYDTFVKFTYDQ------------------------------------------------      159
tr|E2IU46|E2IU46_ORNER              QYDTFVKFTYDQ------------------------------------------------      165
tr|A0A0D5Y9E2|A0A0D5Y9E2_9ACAR      QYDTFVKFTYDQ------------------------------------------------      158
tr|A0A2R5LKD0|A0A2R5LKD0_9ACAR      QYDTFVKFTYDQIQKKFEG------------ATPSYLS----------------------      173


tr|B5L666|B5L666_RHIMP              ---------------CVETFTDFPPLGRFAVRDMRQ-------TVA--------------      195
tr|B5L665|B5L665_RHIMP              ------------------------------------------------------------      94
tr|A0A6M2D4F4|A0A6M2D4F4_RHIMP      ------------------------------------------------------------      96
tr|Q6JIC2|Q6JIC2_ANAMA              VKIDEITNTSVMLNGCYDVLHTDLPVSPYVCAGIGASFVDISKQVTTKLAYRGKVGISYQ      224
tr|R9R561|R9R561_9DIPT              ------------------------------------------------------------      67
tr|S4U8E4|S4U8E4_9DIPT              ------------------------------------------------------------      144
tr|S4UAH5|S4UAH5_CULPP              ------------------------------------------------------------      198
tr|C3RX28|C3RX28_AEDAL              ------------------------------------------------------------      200
tr|A0A3M7Q970|A0A3M7Q970_BRAPC      ------------------------------------------------------------      216
tr|J7K3S8|J7K3S8_9ACAR              ------------------------------------------------------------      158
tr|J7K8Y7|J7K8Y7_9ACAR              ------------------------------------------------------------      158
tr|J7K9Q0|J7K9Q0_9ACAR              ------------------------------------------------------------      158
tr|J7K8Z4|J7K8Z4_9ACAR              ------------------------------------------------------------      158
tr|J7K706|J7K706_9ACAR              ------------------------------------------------------------      158
tr|A0A0X9PMU2|A0A0X9PMU2_9ACAR      ------------------------------------------------------------      172
tr|J7K9P7|J7K9P7_9ACAR              ------------------------------------------------------------      158
tr|A0A9J6F3J6|A0A9J6F3J6_RHIMP      ------------------------------------------------------------      173
tr|A0A9D4Q191|A0A9D4Q191_RHISA      ------------------------------------------------------------      175
tr|A0A131YRI7|A0A131YRI7_RHIAP      ------------------------------------------------------------      173
tr|A0A224Z051|A0A224Z051_9ACAR      ------------------------------------------------------------      173
tr|A0A443S7G3|A0A443S7G3_9ACAR      ------------------------------------------------------------      191
tr|R9R3J1|R9R3J1_AEDAL              ---------SSAIAAAAAAAKRPSPFAEAVCPK----------QL----TFN--TGSRPD      180
tr|A0A0B5J531|A0A0B5J531_9ACAR      ------------------------------------------------------------      191
tr|S4UAF8|S4UAF8_IXOHE              ------------------------------------------------------------      175
tr|R9R416|R9R416_AEDAL              ------------------------------------------------------------      215
tr|Q1AES0|Q1AES0_IXORI              ------------------------------------------------------------      184
tr|S4UF09|S4UF09_IXOSC              ------------------------------------------------------------      184
tr|S4U8C9|S4U8C9_IXOSC              ------------------------------------------------------------      184
tr|S4U9E5|S4U9E5_IXOSC              ------------------------------------------------------------      184
tr|A0A0A7E016|A0A0A7E016_IXOPE      ------------------------------------------------------------      184
tr|Q4VRW2|Q4VRW2_IXOSC              ------------------------------------------------------------      184
tr|A0A0A7DZX9|A0A0A7DZX9_IXOPE      ------------------------------------------------------------      184
tr|W8E787|W8E787_IXOPE              ------------------------------------------------------------      90
tr|W8E0V9|W8E0V9_IXOPE              ------------------------------------------------------------      91
tr|W8E329|W8E329_9ACAR              ------------------------------------------------------------      91
tr|A0A1E1XVC7|A0A1E1XVC7_AMBSC      ------------------------------------------------------------      175
tr|A0A1E1X1X1|A0A1E1X1X1_9ACAR      ------------------------------------------------------------      178
tr|S4U9F0|S4U9F0_AMBAM              ------------------------------------------------------------      163
tr|A0A0C9RT91|A0A0C9RT91_AMBAM      ------------------------------------------------------------      177
tr|S4U8D1|S4U8D1_AMBAM              ------------------------------------------------------------      165
tr|Q1AER9|Q1AER9_AMBAM              ------------------------------------------------------------      163
tr|S4UF12|S4UF12_AMBMU              ------------------------------------------------------------      163
tr|S4UAG1|S4UAG1_AMBCJ              ------------------------------------------------------------      163
tr|S4U8D4|S4U8D4_AMBVA              ------------------------------------------------------------      163
tr|A0A7D4XVE8|A0A7D4XVE8_AMBVA      ------------------------------------------------------------      177
tr|B7SCS6|B7SCS6_AMBHE              ------------------------------------------------------------      163
tr|Q1AER3|Q1AER3_RHISA              ------------------------------------------------------------      161
tr|A0A097BQ10|A0A097BQ10_RHIHE      ------------------------------------------------------------      161
tr|A0A0H3WJP2|A0A0H3WJP2_RHIHE      ------------------------------------------------------------      161
tr|I0B6E2|I0B6E2_RHIMP              ------------------------------------------------------------      161
tr|I3XPN4|I3XPN4_RHIMP              ------------------------------------------------------------      161
tr|I0B6D9|I0B6D9_RHIMP              ------------------------------------------------------------      161
tr|I0B6D7|I0B6D7_RHIMP              ------------------------------------------------------------      161
tr|I0B6E5|I0B6E5_RHIMP              ------------------------------------------------------------      161
tr|S4UD36|S4UD36_RHIMP              ------------------------------------------------------------      148
tr|S4U8E0|S4U8E0_9ACAR              ------------------------------------------------------------      147
tr|A0A7D5D0F6|A0A7D5D0F6_RHIDE      ------------------------------------------------------------      161
tr|J7K2D7|J7K2D7_RHIMP              ------------------------------------------------------------      161
tr|I0B6E3|I0B6E3_RHIMP              ------------------------------------------------------------      161
```

```
tr|S4U8D6|S4U8D6_RHIMP            ------------------------------------------------------------      147
tr|S4UD31|S4UD31_RHIMP            ------------------------------------------------------------      147
tr|Q1AER5|Q1AER5_RHIMP            ------------------------------------------------------------      147
tr|A0A7D5D0I3|A0A7D5D0I3_RHIAP    ------------------------------------------------------------      161
tr|J7K8Z9|J7K8Z9_RHIMP            ------------------------------------------------------------      161
tr|I0B6D6|I0B6D6_RHIMP            ------------------------------------------------------------      161
tr|S4U9H0|S4U9H0_RHIEV            ------------------------------------------------------------      161
tr|J7K715|J7K715_RHIMP            ------------------------------------------------------------      161
tr|S4UAH0|S4UAH0_RHIDE            ------------------------------------------------------------      147
tr|Q1AER6|Q1AER6_RHIMP            ------------------------------------------------------------      147
tr|B6CM56|B6CM56_RHIMP            ------------------------------------------------------------      161
tr|S4UD38|S4UD38_DERRT            ------------------------------------------------------------      150
tr|A0A097BQ19|A0A097BQ19_9ACAR    ------------------------------------------------------------      165
tr|K9R5H5|K9R5H5_DERSI            ------------------------------------------------------------      165
tr|A0A0U2ID78|A0A0U2ID78_9ACAR    ------------------------------------------------------------      163
tr|A0A0U2L412|A0A0U2L412_9ACAR    ------------------------------------------------------------      163
tr|A0A0U2TZZ5|A0A0U2TZZ5_9ACAR    ------------------------------------------------------------      163
tr|S4U8E3|S4U8E3_HYARU            ------------------------------------------------------------      149
tr|A0A0U2URL3|A0A0U2URL3_9ACAR    ------------------------------------------------------------      163
tr|A0A0U2T382|A0A0U2T382_9ACAR    ------------------------------------------------------------      163
tr|A0A0U2LZ49|A0A0U2LZ49_9ACAR    ------------------------------------------------------------      163
tr|A0A0U2T040|A0A0U2T040_9ACAR    ------------------------------------------------------------      163
tr|A0A0U2SNR8|A0A0U2SNR8_9ACAR    ------------------------------------------------------------      163
tr|A0A0U2K641|A0A0U2K641_9ACAR    ------------------------------------------------------------      163
tr|S4UAH3|S4UAH3_9ACAR            ------------------------------------------------------------      163
tr|A0A131XM91|A0A131XM91_9ACAR    ------------------------------------------------------------      163
tr|A0A077B3N4|A0A077B3N4_HAEFA    ------------------------------------------------------------      179
tr|S4UF21|S4UF21_9ACAR            ------------------------------------------------------------      175
tr|A0A7L7T4G3|A0A7L7T4G3_HAELO    ------------------------------------------------------------      179
tr|E4W3Y9|E4W3Y9_HAELO            ------------------------------------------------------------      137
tr|A0A293MGQ8|A0A293MGQ8_ORNER    ------------------------------------------------------------      147
tr|S4U9H1|S4U9H1_ORNSA            ------------------------------------------------------------      174
tr|S4UD40|S4UD40_ORNMO            ------------------------------------------------------------      173
tr|E2IU45|E2IU45_ORNMO            ------------------------------------------------------------      159
tr|E2IU46|E2IU46_ORNER            ------------------------------------------------------------      165
tr|A0A0D5Y9E2|A0A0D5Y9E2_9ACAR    ------------------------------------------------------------      158
tr|A0A2R5LKD0|A0A2R5LKD0_9ACAR    ------------------------------------------------------------      173


tr|B5L666|B5L666_RHIMP            -----------------------------------------------------------       195
tr|B5L665|B5L665_RHIMP            -----------------------------------------------------------       94
tr|A0A6M2D4F4|A0A6M2D4F4_RHIMP    -----------------------------------------------------------       96
tr|Q6JIC2|Q6JIC2_ANAMA            FTPEISLVAGGFYHGLFDESYKDIPAHNS-VKFSGEAKASVKAHIADYGFNLGARFLFS       282
tr|R9R561|R9R561_9DIPT            -----------------------------------------------------------       67
tr|S4U8E4|S4U8E4_9DIPT            -----------------------------------------------------------       144
tr|S4UAH5|S4UAH5_CULPP            -----------------------------------------------------------       198
tr|C3RX28|C3RX28_AEDAL            -----------------------------------------------------------       200
tr|A0A3M7Q970|A0A3M7Q970_BRAPC    -----------------------------------------------------------       216
tr|J7K3S8|J7K3S8_9ACAR            -----------------------------------------------------------       158
tr|J7K8Y7|J7K8Y7_9ACAR            -----------------------------------------------------------       158
tr|J7K9Q0|J7K9Q0_9ACAR            -----------------------------------------------------------       158
tr|J7K8Z4|J7K8Z4_9ACAR            -----------------------------------------------------------       158
tr|J7K706|J7K706_9ACAR            -----------------------------------------------------------       158
tr|A0A0X9PMU2|A0A0X9PMU2_9ACAR    -----------------------------------------------------------       172
tr|J7K9P7|J7K9P7_9ACAR            -----------------------------------------------------------       158
tr|A0A9J6F3J6|A0A9J6F3J6_RHIMP    -----------------------------------------------------------       173
tr|A0A9D4Q191|A0A9D4Q191_RHISA    -----------------------------------------------------------       175
tr|A0A131YRI7|A0A131YRI7_RHIAP    -----------------------------------------------------------       173
tr|A0A224Z051|A0A224Z051_9ACAR    -----------------------------------------------------------       173
tr|A0A443S7G3|A0A443S7G3_9ACAR    -----------------------------------------------------------       191
tr|R9R3J1|R9R3J1_AEDAL            SPPSMVL--FTF-KQALREQYDAVLTNKLAEQYDAAAP----------------SYLS-       219
tr|A0A0B5J531|A0A0B5J531_9ACAR    -----------------------------------------------------------       191
tr|S4UAF8|S4UAF8_IXOHE            -----------------------------------------------------------       175
tr|R9R416|R9R416_AEDAL            -----------------------------------------------------------       215
tr|Q1AES0|Q1AES0_IXORI            -----------------------------------------------------------       184
tr|S4UF09|S4UF09_IXOSC            -----------------------------------------------------------       184
tr|S4U8C9|S4U8C9_IXOSC            -----------------------------------------------------------       184
tr|S4U9E5|S4U9E5_IXOSC            -----------------------------------------------------------       184
tr|A0A0A7E016|A0A0A7E016_IXOPE    -----------------------------------------------------------       184
tr|Q4VRW2|Q4VRW2_IXOSC            -----------------------------------------------------------       184
tr|A0A0A7DZX9|A0A0A7DZX9_IXOPE    -----------------------------------------------------------       184
tr|W8E787|W8E787_IXOPE            -----------------------------------------------------------       90
tr|W8E0V9|W8E0V9_IXOPE            -----------------------------------------------------------       91
tr|W8E329|W8E329_9ACAR            -----------------------------------------------------------       91
tr|A0A1E1XVC7|A0A1E1XVC7_AMBSC    -----------------------------------------------------------       175
tr|A0A1E1X1X1|A0A1E1X1X1_9ACAR    -----------------------------------------------------------       178
tr|S4U9F0|S4U9F0_AMBAM            -----------------------------------------------------------       163
tr|A0A0C9RT91|A0A0C9RT91_AMBAM    -----------------------------------------------------------       177
tr|S4U8D1|S4U8D1_AMBAM            -----------------------------------------------------------       165
tr|Q1AER9|Q1AER9_AMBAM            -----------------------------------------------------------       163
tr|S4UF12|S4UF12_AMBMU            -----------------------------------------------------------       163
tr|S4UAG1|S4UAG1_AMBCJ            -----------------------------------------------------------       163
tr|S4U8D4|S4U8D4_AMBVA            -----------------------------------------------------------       163
tr|A0A7D4XVE8|A0A7D4XVE8_AMBVA    -----------------------------------------------------------       177
tr|B7SCS6|B7SCS6_AMBHE            -----------------------------------------------------------       163
tr|Q1AER3|Q1AER3_RHISA            -----------------------------------------------------------       161
tr|A0A097BQ10|A0A097BQ10_RHIHE    -----------------------------------------------------------       161
tr|A0A0H3WJP2|A0A0H3WJP2_RHIHE    -----------------------------------------------------------       161
tr|I0B6E2|I0B6E2_RHIMP            -----------------------------------------------------------       161
tr|I3XPN4|I3XPN4_RHIMP            -----------------------------------------------------------       161
tr|I0B6D9|I0B6D9_RHIMP            -----------------------------------------------------------       161
tr|I0B6D7|I0B6D7_RHIMP            -----------------------------------------------------------       161
tr|I0B6E5|I0B6E5_RHIMP            -----------------------------------------------------------       161
tr|S4UD36|S4UD36_RHIMP            -----------------------------------------------------------       148
```

```
tr|S4U8E0|S4U8E0_9ACAR                ------------------------------------------------------------      147
tr|A0A7D5D0F6|A0A7D5D0F6_RHIDE        ------------------------------------------------------------      161
tr|J7K2D7|J7K2D7_RHIMP                ------------------------------------------------------------      161
tr|I0B6E3|I0B6E3_RHIMP                ------------------------------------------------------------      161
tr|S4U8D6|S4U8D6_RHIMP                ------------------------------------------------------------      147
tr|S4UD31|S4UD31_RHIMP                ------------------------------------------------------------      147
tr|Q1AER5|Q1AER5_RHIMP                ------------------------------------------------------------      147
tr|A0A7D5D0I3|A0A7D5D0I3_RHIAP        ------------------------------------------------------------      161
tr|J7K8Z9|J7K8Z9_RHIMP                ------------------------------------------------------------      161
tr|I0B6D6|I0B6D6_RHIMP                ------------------------------------------------------------      161
tr|S4U9H0|S4U9H0_RHIEV                ------------------------------------------------------------      161
tr|J7K715|J7K715_RHIMP                ------------------------------------------------------------      161
tr|S4UAH0|S4UAH0_RHIDE                ------------------------------------------------------------      147
tr|Q1AER6|Q1AER6_RHIMP                ------------------------------------------------------------      147
tr|B6CM56|B6CM56_RHIMP                ------------------------------------------------------------      161
tr|S4UD38|S4UD38_DERRT                ------------------------------------------------------------      150
tr|A0A097BQ19|A0A097BQ19_9ACAR        ------------------------------------------------------------      165
tr|K9R5H5|K9R5H5_DERSI                ------------------------------------------------------------      165
tr|A0A0U2ID78|A0A0U2ID78_9ACAR        ------------------------------------------------------------      163
tr|A0A0U2L412|A0A0U2L412_9ACAR        ------------------------------------------------------------      163
tr|A0A0U2TZZ5|A0A0U2TZZ5_9ACAR        ------------------------------------------------------------      163
tr|S4U8E3|S4U8E3_HYARU                ------------------------------------------------------------      149
tr|A0A0U2URL3|A0A0U2URL3_9ACAR        ------------------------------------------------------------      163
tr|A0A0U2T382|A0A0U2T382_9ACAR        ------------------------------------------------------------      163
tr|A0A0U2LZ49|A0A0U2LZ49_9ACAR        ------------------------------------------------------------      163
tr|A0A0U2T040|A0A0U2T040_9ACAR        ------------------------------------------------------------      163
tr|A0A0U2SNR8|A0A0U2SNR8_9ACAR        ------------------------------------------------------------      163
tr|A0A0U2K641|A0A0U2K641_9ACAR        ------------------------------------------------------------      163
tr|S4UAH3|S4UAH3_9ACAR                ------------------------------------------------------------      163
tr|A0A131XM91|A0A131XM91_9ACAR        ------------------------------------------------------------      163
tr|A0A077B3N4|A0A077B3N4_HAEFA        ------------------------------------------------------------      179
tr|S4UF21|S4UF21_9ACAR                ------------------------------------------------------------      175
tr|A0A7L7T4G3|A0A7L7T4G3_HAELO        ------------------------------------------------------------      179
tr|E4W3Y9|E4W3Y9_HAELO                ------------------------------------------------------------      137
tr|A0A293MGQ8|A0A293MGQ8_ORNER        ------------------------------------------------------------      147
tr|S4U9H1|S4U9H1_ORNSA                ------------------------------------------------------------      174
tr|S4UD40|S4UD40_ORNMO                ------------------------------------------------------------      173
tr|E2IU45|E2IU45_ORNMO                ------------------------------------------------------------      159
tr|E2IU46|E2IU46_ORNER                ------------------------------------------------------------      165
tr|A0A0D5Y9E2|A0A0D5Y9E2_9ACAR        ------------------------------------------------------------      158
tr|A0A2R5LKD0|A0A2R5LKD0_9ACAR        ------------------------------------------------------------      173
```

(C) Peptide search (https://www.uniprot.org/peptide-search) for BM86/BM95 consensus sequence RVQKGTVLCECPWNQHLVGDTCISDCVDKKCHE

```
CLUSTAL O(1.2.4) multiple sequence alignment Rhipicephalus spp.
Results: 100% identity.

tr|Q5ER80|Q5ER80_RHIMP           ------------------------------------------------------------        0
tr|A0A8E2Z927|A0A8E2Z927_RHIMP   MRGIALFVAAVSLIVECTAESSICSDFGNEFCRNAECEVVPGAEDDFVCKCPRDNMYFNA        60
tr|A0A8E2Z7V6|A0A8E2Z7V6_RHIMP   MRGIALFVAAVSLIVECTAESSICSDFGNEFCRNAECEVVPGAEDDFVCKCPRDNMYFNA        60
tr|A0A8E2Z7X7|A0A8E2Z7X7_RHIMP   MRGIALFVAAVSLIVECTAESSICSDFGNEFCRNAECEVVPGAEDDFVCKCPRDNMYFNA        60
tr|A0A8E3CL24|A0A8E3CL24_RHIMP   MRGIALFVAAVSLIVECTAESSICSDFGNEFCRNAECEVVPGAEDDFVCKCPRDNMYFNA        60
tr|A0A8E2Z8E9|A0A8E2Z8E9_RHIMP   MRGIALFVAAVSLIVECTAESSICSDFGNEFCRNAECEVVPSAEDDFVCKCPRDNMYFNA        60
tr|A0A8E2Z916|A0A8E2Z916_RHIMP   MRGIALFVAAVSLIVECTAESSICSDFGNEFCRNAECEVVPGAEDDFVCKCPRDNMYFNA        60
tr|A0A8E2Z7Z1|A0A8E2Z7Z1_RHIMP   MRGIALFVAAVSLIVECTAESSICSDFGNEFCRNAECEVVPGAEDDFVCKCPRDNMYFNA        60
tr|A0A8E3CLH7|A0A8E3CLH7_RHIMP   MRGIALFVAAVSLIVECTAESSICSDFGNEFCRNAECEVVPGAEDDFVCKCPRDNMYFNA        60
tr|A0A8E3CK42|A0A8E3CK42_RHIMP   MRGIALFVAAVSLIVECTAESSICSDFGNEFCRNAECEVVPGAEDDFVCKCPRDNMYFNA        60
tr|C4NAN0|C4NAN0_RHIMP           -------------------ESSICSDFGNEFCRNAECEVVPGAEDDFVCKCPRDNMYFNA        41
tr|E5KCH6|E5KCH6_RHIMP           MRGIALFVAAVSLIVECTAESSICSDFGNEFCRNAECEVVPGAEDDFVCKCPRDNMYFNA        60
tr|Q5XKU6|Q5XKU6_RHIMP           ------------------------------------------------------------        0
tr|Q5ER81|Q5ER81_RHIMP           ------------------------------------------------------------        0
tr|Q5ER93|Q5ER93_RHIMP           ------------------------------------------------------------        0
tr|Q5ER89|Q5ER89_RHIMP           ------------------------------------------------------------        0
tr|A0A8E2Z7V2|A0A8E2Z7V2_RHIMP   MRGIALFVAAVSLIVECTAESSICSDFGNEFCRNAECEVVPGAEDDFVCKCPRYNMYFNA        60
tr|Q5ER77|Q5ER77_RHIMP           ------------------------------------------------------------        0
tr|Q5ER78|Q5ER78_RHIMP           ------------------------------------------------------------        0
tr|Q5ER69|Q5ER69_RHIMP           ------------------------------------------------------------        0
tr|Q5XKU9|Q5XKU9_RHIMP           ------------------------------------------------------------        0
tr|Q9Y0V1|Q9Y0V1_RHIMP           MRGIALFVAAVSLIVEGTAESSICSDFGNEFCRNAECEVVPGAEDDFVCKCPRDNMYFNA        60
tr|Q5XKU7|Q5XKU7_RHIMP           ------------------------------------------------------------        0
tr|Q5I6P2|Q5I6P2_RHIMP           ------------------------------------------------------------        0
tr|Q5ER92|Q5ER92_RHIMP           ------------------------------------------------------------        0
tr|Q5ER88|Q5ER88_RHIMP           ------------------------------------------------------------        0
tr|Q5ER82|Q5ER82_RHIMP           ------------------------------------------------------------        0
tr|A0A8E3CK52|A0A8E3CK52_RHIMP   MRGIALFVAAVSLIVECTAESSICSDFGNEFCRNAECEVVPGAEDDFVCKCPRDNMYFNA        60
tr|A0A8E2ZC71|A0A8E2ZC71_RHIMP   MRGVALFVAAVSLIVECTAESSICSDFGNEFCRNAECEVVPGAEDDFVCKCPRDNMYFNA        60
tr|A0A8E2Z937|A0A8E2Z937_RHIMP   MRGIALFVAAVSLIVECTAESSICSDFGNEFCRNAECEVVPGAEDDFVCKCPRDNMYFNA        60
tr|A0A8E2Z8F5|A0A8E2Z8F5_RHIMP   MRGIALFVAAVSLIVECTAESSICSDFGNEFCRNAECEVVPGAEDDFVCKCPRYNMYFNA        60
tr|A0A8E3CHP7|A0A8E3CHP7_RHIMP   MRGIALFVAAVSLIVECTAESSICSDFGNEFCRNAECEVVPGAEDDFVCKCPRYNMYFNA        60
tr|E5KCI4|E5KCI4_RHIMP           MRGIALFVAAVSLIVECTAESSICSDFGNEFCRNAECEVVPGAEDDFVCKCPRDNMYFNA        60
tr|A0A8E3CM27|A0A8E3CM27_RHIMP   MRGIALFVAAVSLIVECTAESSICSDFGNEFCRNAECEVVPGAEDDFVCKCPRDNMYFNA        60
tr|Q5XKU8|Q5XKU8_RHIMP           ------------------------------------------------------------        0
tr|Q5I6P1|Q5I6P1_RHIMP           ------------------------------------------------------------        0
tr|Q5ER85|Q5ER85_RHIMP           ------------------------------------------------------------        0
tr|Q5ER75|Q5ER75_RHIMP           ------------------------------------------------------------        0
tr|Q5ER64|Q5ER64_RHIMP           ------------------------------------------------------------        0
tr|E5KCI2|E5KCI2_RHIMP           MRGIALFVAAVSLIVECTAESSICSDFGNEFCRNAECEVVPGAEDDFVCKCPRDNMYFNA        60
tr|A0A8E2ZDL2|A0A8E2ZDL2_RHIMP   MRGIALFVAAVSLIVECTAESSICSDFGNEFCRNAECEVVPGAEDDFVCKCPRDNMYFNA        60
tr|A0A8E2ZDK4|A0A8E2ZDK4_RHIMP   MRGIALFVAAVSLIVECTAESSICSDFGNEFCRNAECEVVPGAEDDFVCKCPRDNMYFNA        60
tr|E5KCI6|E5KCI6_RHIMP           MRGIALFVAAVSLIVECTAESSICSDFGNEFCRNAECEVVPGAEDNFVCKCPRDNMYFNA        60
tr|E5KCI5|E5KCI5_RHIMP           MRGIALFVAAVSLIVECTAESSICSDFGNEFCRNAECEVVPGAEDDFVCKCPRDNMYFNA        60
tr|E5KCI1|E5KCI1_RHIMP           MRGIALFVAAVSLIVECTAESSICSDFGNEFCRNAECEVVPGAEDDFVCKCPRDNMYFNA        60
tr|E5KCI0|E5KCI0_RHIMP           MRGIALFVAAVSLIVECTAESSICSDFGNEFCRNAECEVVPGAEDDFVCKCPRDNMYFNA        60
tr|E5KCH9|E5KCH9_RHIMP           MRGIALFVAAVSLIVECTAESSICSDFGNEFCRNAECEVVPGAEDDFVCKCPRDNMYFNA        60
tr|E5KCH3|E5KCH3_RHIMP           MRGIALFVAAVSLIVECTAESSICSDFGNEFCRNAECEVVPGAEDDFVCKCPRDNMYFNA        60
tr|B6CNU3|B6CNU3_RHIMP           --------------------SSICSDFGNEFCRNAECEVVPGAEDDFVCKCPRDNMYFNA        40
tr|A0A8E3CM42|A0A8E3CM42_RHIMP   MRGVALFVAAVSLIVECTAESSICSDFGNEFCRNAECEVVPGAEDDFVCKCPRDNMYFNA        60
tr|A0A8E3CM37|A0A8E3CM37_RHIMP   MRGIALFVAAVSLIVECTAESSICSDFGNEFCRNAECEVVPGAEDDFVCKCPRDNMYFNA        60
tr|A0A8E3CM32|A0A8E3CM32_RHIMP   MRGVALFVAAVSLIVECTAESSICSDFGNEFCRNAECEVVPGAEDDFVCKCPRDNMYFNA        60
tr|A0A8E3CLI5|A0A8E3CLI5_RHIMP   MRGVALFVAAVSLIVECTAESSICSDFGNEFCRNAECEVVPGAEDDFVCKCPRDNMYFNA        60
tr|A0A8E3CLI1|A0A8E3CLI1_RHIMP   MRGVALFVAAVSLIVECTAESSICSDFGNEFCRNAECEVVPGAEDDFVCKCPRDNMYFNA        60
tr|A0A8E3CK47|A0A8E3CK47_RHIMP   MRGVALFVAAVSLIVECTAESSICSDFGNEFCRNAECEVVPGAEDDFVCKCPRDNMYFNA        60
tr|A0A8E3CK39|A0A8E3CK39_RHIMP   MRGIALFVAAVSLIVECTAESSICSDFGNEFCRNAECEVVPGAEDDFVCKCPRDNMYFNA        60
tr|A0A8E3CHR0|A0A8E3CHR0_RHIMP   MRGVALFVAAVSLIVECTAESSICSDFGNEFCRNAECEVVPGAEDDFVCKCPRDNMYFNA        60
tr|A0A8E3CHQ5|A0A8E3CHQ5_RHIMP   MRGVALFVAAVSLIVECTAESSICSDFGNEFCRNAECEVVPGAEDDFVCKCPRDNMYFNA        60
tr|A0A8E2ZDN2|A0A8E2ZDN2_RHIMP   MRGVALFVAAVSLIVECTAESSICSDFGNEFCRNAECEVVPGAEDDFVCKCPRDNMYFNA        60
tr|A0A8E2ZC62|A0A8E2ZC62_RHIMP   MRGIALFVAAVSLIVECTAESSICSDFGNEFCRNAECEVVPGAEDDFVCKCPRDNMYFNA        60
tr|A0A8E2ZC54|A0A8E2ZC54_RHIMP   MRGIALFVAAVSLIVECTAESSICSDFGNEFCRNAECEVVPGAEDDFVCKCPRDNMYFNA        60
tr|A0A8E2ZC45|A0A8E2ZC45_RHIMP   MRGIALFVAAVSLIVECTAESSICSDFGNEFCRNAECEVVPGAEDDFVCKCPRDNMYFNA        60
tr|A0A8E2ZAC1|A0A8E2ZAC1_RHIMP   MRGVALFVAAVSLIVECTAESSICSDFGNEFCRNAECEVVPGAEDDFVCKCPRDNMYFNA        60
tr|A0A8E2ZAB3|A0A8E2ZAB3_RHIMP   MRGIALFVAAVSLIVECTAESSICSDFGNEFCRNAECEVVPGAEDDFVCKCPRDNMYFNA        60
tr|A0A8E2Z9N3|A0A8E2Z9N3_RHIMP   MRGVALFVAAVSLIVECTAESSICSDFGNEFCRNAECEVVPGAEDDFVCKCPRDNMYFNA        60
tr|A0A8E2Z9M6|A0A8E2Z9M6_RHIMP   MRGVALFVAAVSLIVECTAESSICSDFGNEFCRNAECEVVPGAEDDFVCKCPRDNMYFNA        60
tr|A0A8E2Z9L6|A0A8E2Z9L6_RHIMP   MRGVALFVAAVSLIVECTAESSICSDFGNEFCRNAECEVVPGAEDDFVCKCPRDNMYFNA        60
tr|A0A8E2Z8G3|A0A8E2Z8G3_RHIMP   MRGIALFVAAVSLIVECTAESSICSDFGNEFCRNAECEVVPGAEDDFVCKCPRDNMYFNA        60
tr|A0A8E2Z7X1|A0A8E2Z7X1_RHIMP   MRGIALFVAAVSLIVECTAESSICSDFGNEFCRNAECEVVPGAEDDFVCKCPRDNMYFNA        60
tr|A0A8E2Z7W9|A0A8E2Z7W9_RHIMP   MRGIALFVAAVSLIVECTAESSICSDFGNEFCRNAECEVVPGAEDDFVCKCPRDNMYFNA        60
tr|A0A8E2Z7W5|A0A8E2Z7W5_RHIMP   MRGIALFVAAVSLIVECTAESSICSDFGNEFCRNAECEVVPGAEDDFVCKCPRDNMYFNA        60
tr|A0A8E2Z7V4|A0A8E2Z7V4_RHIMP   MRGIALFVAAVSLIVECTAESSICSDFGNEFCRNAECEVVPGAEDDFVCKCPRDNMYFNA        60
tr|A0A8E2Z7U7|A0A8E2Z7U7_RHIMP   MRGIALFVAAVSLIVECTAESSICSDFGNEFCRNAECEVVPGAEDDFVCKCPRDNMYFNA        60
tr|A0A8E2Z7U5|A0A8E2Z7U5_RHIMP   MRGIALFVAAVSLIVECTAESSICSDFGNEFCRNAECEVVPGAEDDFVCKCPRDNMYFNA        60
tr|A0A8E2Z7U4|A0A8E2Z7U4_RHIMP   MRGIALFVAAVSLIVECTAESSICSGFGNEFCRNAECEVVPGAEDDFVCKCPRDNMYFNA        60
tr|A0A8E2Z7T7|A0A8E2Z7T7_RHIMP   MRGVALFVAAVSLIVECTAESSICSDFGNEFCRNAECEVVPGAEDDFVCKCPRDNMYFNA        60
tr|A0A8E2Z7S4|A0A8E2Z7S4_RHIMP   MRGVALFVAAVSLIVECTAESSICSDFGNEFCRNAECEVVPGAEDDFVCKCPRDNMYFNA        60
tr|A0A6M2CI36|A0A6M2CI36_RHIMP   MRGIALFVAAVSLIVECTAESSICSDFGNEFCRNAECEVVPGAEDDFVCKCPRDNMYFNA        60
tr|Q5XKV2|Q5XKV2_RHIMP           ------------------------------------------------------------        0


tr|Q5ER80|Q5ER80_RHIMP           ----------------------ESNPSKGSCVCEASDDLTLQCKIKNDFTTDCRNRGGTA        38
tr|A0A8E2Z927|A0A8E2Z927_RHIMP   AEKQCEYKDTCKTRECSYGRCVESNPSKASCVCEASDDLTLQCKIKKEYATDCQNRGGTA        120
tr|A0A8E2Z7V6|A0A8E2Z7V6_RHIMP   AEKQCEYKDTCKTRECSYGRCVESNPSKASCVCEASDDLTLQCKIKKEFATDCQNRGGTA        120
```

```
tr|A0A8E2Z7X7|A0A8E2Z7X7_RHIMP      AEKQCEYKDTCKTRECSYGRCVESNPSKGSCVCEASDDLTLQCKIKKEFATDCQNRGGTA      120
tr|A0A8E3CL24|A0A8E3CL24_RHIMP      AEKQCEYKDTCKTRECSYGRCVESNPSKASCVCEASDDLTLQCKIKKEYATDCQNRGGTA      120
tr|A0A8E2Z8E9|A0A8E2Z8E9_RHIMP      AEKQCEYKDTCKTRECSYGRCVESNPSKASCVCEASDDLTLQCKIKKEYATDCQNRGGTA      120
tr|A0A8E2Z916|A0A8E2Z916_RHIMP      AEKQCEYKDTCKTRECSYGRCVESNPSKASCVCEASDDLTLQCKIKKEYATDCQNRGGTA      120
tr|A0A8E2Z7Z1|A0A8E2Z7Z1_RHIMP      AEKQCEYKDTCKTRECSYGRCVESNPSKGSCVCEASDDLTLQCKIKKDFATDCRNRGGTA      120
tr|A0A8E3CLH7|A0A8E3CLH7_RHIMP      AEKQCEYKDTCKTRECSYGRCVESNPSKGSCVCEASDDLTLQCKIKNDFATDCRNRGGTA      120
tr|A0A8E3CK42|A0A8E3CK42_RHIMP      AEKQCEYKDTCKTRECSYGRCVESNPSKGSCVCEASDDLTLQCKIKNDFATDCRNRGGTA      120
tr|C4NAN0|C4NAN0_RHIMP              AEKQCEYKDTCKTRECSYGRCVESNPSKGSCVCEASDDLTLQCKIKNDFATDCRNRGGTA      101
tr|E5KCH6|E5KCH6_RHIMP              AEKQCEYKDTCKTRECSYGRCVESNPSKGSCVCEASDDLTLQCKIKNDFATDCRNRGGTA      120
tr|Q5XKU6|Q5XKU6_RHIMP              ----------------------ESNPSKGSCVCEASDDLTLQCKIKNDFATDCRNRGGTA      38
tr|Q5ER81|Q5ER81_RHIMP              ----------------------ESNPSKGSCVCEASDDLTLQCKIKNDFATDCRNRGGTA      38
tr|Q5ER93|Q5ER93_RHIMP              ----------------------ESNPSKGSCVCEASDDLTLQCKIKNDFATDCRNRGGTA      38
tr|Q5ER89|Q5ER89_RHIMP              ----------------------ESNPSKGSCVCEASDDLTLQCKIKNDFATDCRNRGGTA      38
tr|A0A8E2Z7V2|A0A8E2Z7V2_RHIMP      AEKQCEYKDTCKTRECSYGRCVQSNPSKASCVCEASDTLTLQCKINNDYATDCRNRGGTA      120
tr|Q5ER77|Q5ER77_RHIMP              ----------------------ESNPSKGSCVCEASDDLTLQCKIKNDFATDCRNRGGTA      38
tr|Q5ER78|Q5ER78_RHIMP              ----------------------ESNPSKGSCVCEASDDLTLQCKIKNDFATDCRNRGGTA      38
tr|Q5ER69|Q5ER69_RHIMP              ----------------------ESNPSKGSCVCEASDDLTLQCKIKNDFATDCRNRGGTA      38
tr|Q5XKU9|Q5XKU9_RHIMP              ----------------------ESNPSKGSCVCEASDDLTLQCKIKNDFATDCRNRGGTA      38
tr|Q9Y0V1|Q9Y0V1_RHIMP              AEKQCEYKDTCKTRECSYGRCVESNPSKGSCVCERSDDLTLQCKIKNDYATDCRNRGGTA      120
tr|Q5XKU7|Q5XKU7_RHIMP              ----------------------ESNPSKGSCVCEASDDLTLQCKIKNDFATDCRNRGGTA      38
tr|Q5I6P2|Q5I6P2_RHIMP              ----------------------ESNPSKGSCVCEASDDLTLQCKIKNDFATDCRNRGGTA      38
tr|Q5ER92|Q5ER92_RHIMP              ----------------------ESNPSKGSCVCEASDDLTLLCKIKNDFATDCRNRGGTA      38
tr|Q5ER88|Q5ER88_RHIMP              ----------------------ESNPSKGSCVCEESDDLTLQCKIKNDFATDCRNRGGTA      38
tr|Q5ER82|Q5ER82_RHIMP              ----------------------ESNPSKGSCVCEASDDLTLQCKIKNDFATDCRNRGGTA      38
tr|A0A8E3CK52|A0A8E3CK52_RHIMP      AEKQCEYKDTCKTRECSYGRCVESNPSKGSCVCEASDDLTLQCKIKNDFATDCRNRGGTA      120
tr|A0A8E2ZC71|A0A8E2ZC71_RHIMP      AEKQCEYKDTCKTRECSYGRCVESNPSKGSCVCEASDDLTLQCKIKNDFATDCRNRGGTA      120
tr|A0A8E2Z937|A0A8E2Z937_RHIMP      AEKQCEYKDTCKTRECSYGRCVQSNPSKGSCVCEASDDLTLQCNINNDFATDCRNRGGTA      120
tr|A0A8E2Z8F5|A0A8E2Z8F5_RHIMP      AEKQCEYKDTCKTRECSYGRCVQSNPSKASCVCEASDALTLQCNINNDYATDCRNRGGTA      120
tr|A0A8E3CHP7|A0A8E3CHP7_RHIMP      AEKQCEYKDTCKTRECSYGRCVQSNPSKASCVCEASDTLTLQCNINNDYATDCRNRGGTA      120
tr|E5KCI4|E5KCI4_RHIMP              AEKQCEYKDTCKTRECSYGRCVESNPSKGSCVCEASDDLTLQCNINNDYATDCRNRGGTA      120
tr|A0A8E3CM27|A0A8E3CM27_RHIMP      AEKQCEYKDTCKTRECSYGRCVESNPSKASCVCEASDDLTLQCNINNDFATDCRNRGGTA      120
tr|Q5XKU8|Q5XKU8_RHIMP              ----------------------ESNPSKGSCVCEASDDLTLQCKIKNDFATDCRNRGGTA      38
tr|Q5I6P1|Q5I6P1_RHIMP              ----------------------ESNPSKGSCVCEASDDLTLQCKIKNDFATDCRNRGGTA      38
tr|Q5ER85|Q5ER85_RHIMP              ----------------------ESNPSKGSCVCEASDDLTLQCKIKNDFATDCRNRGGTA      38
tr|Q5ER75|Q5ER75_RHIMP              ----------------------ESNPSKGSCVCEASDDLTLQCKIKNDFATDCRNRGGTA      38
tr|Q5ER64|Q5ER64_RHIMP              ----------------------ESNPSKGSCVCEASDDLTLQCKIKNDFATDCRNRGGTA      38
tr|E5KCI2|E5KCI2_RHIMP              AEKQCEYKDTCKTRECSYGRCVESNPSKASCVCEASDDLTLQCKIKNDYATDCRNRGGTA      120
tr|A0A8E2ZDL2|A0A8E2ZDL2_RHIMP      AEKQCEYKDTCKTRECSYGRCVESNPSKGSCVCEASDDLTLQCKIKNDFATDCRNRGGTA      120
tr|A0A8E2ZDK4|A0A8E2ZDK4_RHIMP      AEKQCEYKDTCKTRECSYGRCVESNPSKGSCVCEASDDLTLQCKIKNDFATDCRNRGGTA      120
tr|E5KCI6|E5KCI6_RHIMP              AEKQCEYKDTCKTRECSYGRCVESNPSKGSCVCEASDDLTLQCKIKNDFATDCRNRGGTA      120
tr|E5KCI5|E5KCI5_RHIMP              AEKQCEYKDTCKTRECSYGRCVESNPSKGSCVCEASDDLTLQCKIKNDFATDCRNRGGTA      120
tr|E5KCI1|E5KCI1_RHIMP              AEKQCEYKDTCKTRECSYGRCVESNPSKGSCVCEASDDLTLQCKIKNDFATDCRNRGGTA      120
tr|E5KCI0|E5KCI0_RHIMP              AEKQCEYKDTCKTRECSYGRCVESNPSKGSCVCEASDDLTLQCKIKNDFATDCRNRGGTA      120
tr|E5KCH9|E5KCH9_RHIMP              AEKQCEYKDTCKTRECSYGRCVESNPSKGSCVCEASDDLTLQCKIKNDFATDCRNRGGTA      120
tr|E5KCH3|E5KCH3_RHIMP              AEKQCEYKDTCKTRECSYGRCVESNPSKGSCVCEASDDLTLQCKIKNDFATDCRNRGGTA      120
tr|B6CNU3|B6CNU3_RHIMP              AEKQCEYKDTCKTRECSYGRCVESNPSKGSCVCEASDDLTLQCKIKNDFATDCRNRGGTA      100
tr|A0A8E3CM42|A0A8E3CM42_RHIMP      AEKQCEYKDTCKTRECSYGRCVESNPSKGSCVCEASDDLTLQCKIKNDFATDCRNRGGTA      120
tr|A0A8E3CM37|A0A8E3CM37_RHIMP      AEKQCEYKDTCKTRECSYGRCVESNPSKGSCVCEASDDLTLQCKIKNDFATDCRNRGGTA      120
tr|A0A8E3CM32|A0A8E3CM32_RHIMP      AEKQCEYKDTCKTRECSYGRCVESNPSKGSCVCEASDDLTLQCKIKNDFATDCRNRGGTA      120
tr|A0A8E3CLI5|A0A8E3CLI5_RHIMP      AEKQCEYKDTCKTRECSYGRCVESNPSKGSCVCEASDDLTLQCKIKNDFATDCRNRGGTA      120
tr|A0A8E3CLI1|A0A8E3CLI1_RHIMP      AEKQCEYKDTCKTRECSYGRCVESNPSKGSCVCEASDDLTLQCKIKNDFATDCRNRGGTA      120
tr|A0A8E3CK47|A0A8E3CK47_RHIMP      AEKQCEYKDTCKTRECSYGRCVESNPSKGSCVCEASDDLTLQCKIKNDFATDCRNRGGTA      120
tr|A0A8E3CK39|A0A8E3CK39_RHIMP      AEKQCEYKDTCKTRECSYGRCVESNPSKGSCVCEASDDLTLQCKIKNDFATDCRNRGGTA      120
tr|A0A8E3CHR0|A0A8E3CHR0_RHIMP      AEKQCEYKDTCKTRECSYGRCVESNPSKGSCVCEASDDLTLQCKIKNDFATDCRNRGGTA      120
tr|A0A8E3CHQ5|A0A8E3CHQ5_RHIMP      AEKQCEYKDTCKTRECSYGRCVESNPSKGSCVCEASDDLTLQCKIKNDFATDCRNRGGTA      120
tr|A0A8E2ZDN2|A0A8E2ZDN2_RHIMP      AEKQCEYKDTCKTRECSYGRCVESNPSKGSCVCEASDDLTLQCKIKNDFATDCRNRGGTA      120
tr|A0A8E2ZC62|A0A8E2ZC62_RHIMP      AEKQCEYKDTCKTRECSYGRCVESNPSKGSCVCEASDDLTLQCKIKNDFATDCRNRGGTA      120
tr|A0A8E2ZC54|A0A8E2ZC54_RHIMP      AEKQCEYKDTCKTRECSYGRCVESNPSKGSCVCEASDDLTLQCKIKNDFATDCRNRGGTA      120
tr|A0A8E2ZC45|A0A8E2ZC45_RHIMP      AEKQCEYKDTCKTRECSYGRCVESNPSKGSCVCEASDDLTLQCKIKNDFATDCRNRGGTA      120
tr|A0A8E2ZAC1|A0A8E2ZAC1_RHIMP      AEKQCEYKDTCKTRECSYGRCVESNPSKGSCVCEASDDLTLQCKIKNDFATDCRNRGGTA      120
tr|A0A8E2ZAB3|A0A8E2ZAB3_RHIMP      AEKQCEYKDTCKTRECSYGRCVESNPSKGSCVCEASDDLTLQCKIKNDFATDCRNRGGTA      120
tr|A0A8E2Z9N3|A0A8E2Z9N3_RHIMP      AEKQCEYKDTCKTRECSYGRCVESNPSKGSCVCEASDDLTLQCKIKNDFATDCRNRGGTA      120
tr|A0A8E2Z9M6|A0A8E2Z9M6_RHIMP      AEKQCEYKDTCKTRECSYGRCVESNPSKGSCVCEASDDLTLQCKIKNDFATDCRNRGGTA      120
tr|A0A8E2Z9L6|A0A8E2Z9L6_RHIMP      AEKQCEYKDTCKTRECSYGRCVESNPSKGSCVCEASDDLTLQCKIKNDFATDCRNRGGTA      120
tr|A0A8E2Z8G3|A0A8E2Z8G3_RHIMP      AEKQCEYKDTCKTRECSYGRCVESNPSKGSCVCEASDDLTLQCKIKNDFATDCRNRGGTA      120
tr|A0A8E2Z7X1|A0A8E2Z7X1_RHIMP      AEKQCEYKDTCKTRECSYGRCVESNPSKGSCVCEASDDLTLQCKIKNDFATDCRNRGGTA      120
tr|A0A8E2Z7W9|A0A8E2Z7W9_RHIMP      AEKQCEYKDTCKTRECSYGRCVESNPSKGSCVCEASDDLTLQCKIKNDFATDCRNRGGTA      120
tr|A0A8E2Z7W5|A0A8E2Z7W5_RHIMP      AEKQCEYKDTCKTRECSYGRCVESNPSKGSCVCEASDDLTLQCKIKNDFATDCRNRGGTA      120
tr|A0A8E2Z7V4|A0A8E2Z7V4_RHIMP      AEKQCEYKDTCKTRECSYGRCVESNPSKGSCVCEASDDLTLQCKIKNDFATDCRNRGGTA      120
tr|A0A8E2Z7U7|A0A8E2Z7U7_RHIMP      AEKQCEYKDTCKTRECSYGRCVESNPSKGSCVCEASDDLTLQCKIKNDFATDCRNRGGTA      120
tr|A0A8E2Z7U5|A0A8E2Z7U5_RHIMP      AEKQCEYKDTCKTRECSYGRCVESNPSKGSCVCEASDDLTLQCKIKNDFATDCRNRGGTA      120
tr|A0A8E2Z7U4|A0A8E2Z7U4_RHIMP      AEKQCEYKDTCKTRECSYGRCVESNPSKGSCVCEASDDLTLQCKIKNDFATDCRNRGGTA      120
tr|A0A8E2Z7T7|A0A8E2Z7T7_RHIMP      AEKQCEYKDTCKTRECSYGRCVESNPSKGSCVCEASDDLTLQCKIKNDFATDCRNRGGTA      120
tr|A0A8E2Z7S4|A0A8E2Z7S4_RHIMP      AEKQCEYKDTCKTRECSYGRCVESNPSKGSCVCEASDDLTLQCKIKNDFATDCRNRGGTA      120
tr|A0A6M2CI36|A0A6M2CI36_RHIMP      AEKQCEYKDTCKTRECSYGRCVESNPSKGSCVCEASDDLTLQCKIKNDFATDCRNRGGTA      120
tr|Q5XKV2|Q5XKV2_RHIMP              ----------------------ESNPSKGSCVCEASDDLTLQCKIKNDFATDCRNRGGTA      38
                                                          :*****.***** ** *** *:*:::::***:******

tr|Q5ER80|Q5ER80_RHIMP              KLRTDGFIGATCDCGEWGAMNKTTRNCVPTTCLRPDLTCKDLCEKDLLQRDSRCWQGWNT      98
tr|A0A8E2Z927|A0A8E2Z927_RHIMP      KLRTDGFIGATCDCGEWGAMNKTTRNCVPTTCLRPDLTCKDLCEKNLLQRDSRCCQGWNT      180
tr|A0A8E2Z7V6|A0A8E2Z7V6_RHIMP      KLRTDGFIGATCDCGEWGAMNKTTRNCVPTTCLRPDLTCKDLCEKNLLQRDSRCCQGWNT      180
tr|A0A8E2Z7X7|A0A8E2Z7X7_RHIMP      KLRTDGFIGATCDCGEWGAMNKTTRNCVPTTCLRPDLTCKDLCEKNLLQRDSRCCQGWNT      180
tr|A0A8E3CL24|A0A8E3CL24_RHIMP      KLRTDGFIGATCDCGEWGAMNKTTRNCVPTTCLRPDLTCKDLCEKNLLQRDSRCCQGWNT      180
tr|A0A8E2Z8E9|A0A8E2Z8E9_RHIMP      KLRTDGFIGATCDCGEWGAMNKTTRNCVPTTCLRPDLTCKDLCEKNLLQRDSRCCQGWNT      180
tr|A0A8E2Z916|A0A8E2Z916_RHIMP      KLRTDGFIGATCDCGEWGAMNKTTRNCVPTTCLRPDLTCKDLCEKNLLQRDSRCCQGWNT      180
tr|A0A8E2Z7Z1|A0A8E2Z7Z1_RHIMP      KLRTDGFIGATCDCGEWGAMNKTTRNCVPTTCLRPDLTCKDLCEKNLLQRDSRCCQGWNT      180
tr|A0A8E3CLH7|A0A8E3CLH7_RHIMP      KLRTDGFIGATCDCGEWGAMNKTTRNCVPTTCLRPDLTCKDLCEKNLLQRDSRCCQGWNT      180
tr|A0A8E3CK42|A0A8E3CK42_RHIMP      KLRTDGFIGATCDCGEWGAMNKTTRNCVPTTCLRPDLTCKDLCEKNLLQRDSRCCQGWNT      180
tr|C4NAN0|C4NAN0_RHIMP              KLRTDGFIGATCDCGEWGAMNKTTRNCVPTTCLRPDLTCKDLCEKNLLQRDSRCCQGWNT      161
tr|E5KCH6|E5KCH6_RHIMP              KLRTDGFIGATCDCGEWGAMNKTTRNCVPTTCLRPDLTCKDLCEKNLLQRDSRCCQGWNT      180
tr|Q5XKU6|Q5XKU6_RHIMP              KLRTDGFIGATCDCGEWGAMNKTTQNCVPTTCLRPDLTCKDLCEKNLLQRDSRCCQGWST      98
tr|Q5ER81|Q5ER81_RHIMP              KLRTDGFIGATCDCGEWGAMNKTTRNRVPTTCLRPDLTCKDLCEKNLLQRDSRCCQGWNT      98
tr|Q5ER93|Q5ER93_RHIMP              KLRTDGFIGATCDCGEWGAVYKTTRNCVPTTCLRPDLTCKDLCEKNLLQRDSRCCQGWNT      98
tr|Q5ER89|Q5ER89_RHIMP              KLRTDGFIGATCDCGEWGAMNKTTRNCVPTTCLHPDLTCKDPCEKNLLQRDSRCCQGWNT      98
```

```
tr|A0A8E2Z7V2|A0A8E2Z7V2_RHIMP      KLRTDGFIGATCDCGEWGAMNKTTRNCVPTTCLRPDLTCKDLCEKNLLQRDSRCCQGWNT      180
tr|Q5ER77|Q5ER77_RHIMP              KLRTDGFIGATCDCGEWGAMNKTTRNCVPTTCLRPDLACKDLCEKNLLQRDSRCCQGWNT      98
tr|Q5ER78|Q5ER78_RHIMP              KLRTDGFIGATCDCGEWGAMNKTTRNCVPTTCLRPDLTCKDLCEKNLLQRDSRCCQGWNT      98
tr|Q5ER69|Q5ER69_RHIMP              KLRTDGFIGATCDCGEWGAMNKTTRNCVPTTCLRPDLTCKDLCEKNLPQRDSRCCQGWNT      98
tr|Q5XKU9|Q5XKU9_RHIMP              KLRTDGFIGATCDCGEWGAMNKTTRNCVPTTCLRPDLTCKDLCEKNLPQRDSRCCQGWNT      98
tr|Q9Y0V1|Q9Y0V1_RHIMP              KLRTDGFIGATCDCGEWGAMNKTTRNCVPTTCLRPDLTCKDLCEKNLLQRDSRCCQGWNT      180
tr|Q5XKU7|Q5XKU7_RHIMP              KLRTDGFIGATCDCGEWGAMNKTTRNCVPTTCLRPDLTYKDLCEKNLLQRDSRCCQGWST      98
tr|Q5I6P2|Q5I6P2_RHIMP              KLRTDGFIGATCDCGEWGAMNKTTRNCVPTTCLRPDLTCKDLCEKNLLQRDSRCCQGWNT      98
tr|Q5ER92|Q5ER92_RHIMP              KLRTDGFIGATCDRGEWGAMNKTTRNCVPTTCLRPDLTCKDLCEKNLLQRDSRCCQGWNT      98
tr|Q5ER88|Q5ER88_RHIMP              KLRTDGFIGATCDCGEWGAMSKTTRNCVPTTCLRPDLTCKDLCEKNLLQRDSRCCQGWNT      98
tr|Q5ER82|Q5ER82_RHIMP              KLRTDGFIGATCDCGEWGAMNKTTRNCVPTTCLRPDLTCKDLCEKNLLQRDSRCCQGWNT      98
tr|A0A8E3CK52|A0A8E3CK52_RHIMP      KLRTDGFIGATCDCGEWGAMNKTTRNCVPTTCLRPDLTCKDLCEKNLLQRDSRCCQGWNT      180
tr|A0A8E2ZC71|A0A8E2ZC71_RHIMP      KLRTDGFIGATCDCGEWGAMNKTTRNCVPTTCLRPDLTCKDLCEKNLLQRDSRCCQGWNT      180
tr|A0A8E2Z937|A0A8E2Z937_RHIMP      KLRTDGFIGATCDCGEWGAMNKTTRNCVPTTCLRPDLTCKDLCEKNLLQRDSRCCQGWNT      180
tr|A0A8E2Z8F5|A0A8E2Z8F5_RHIMP      KLRTDGFIGATCDCGEWGAMNKTTRNCVPTTCLRPDLTCKDLCEKNLLQRDSRCCQGWNT      180
tr|A0A8E3CHP7|A0A8E3CHP7_RHIMP      KLRTDGFIGATCDCGEWGAMNKTTRNCVPTTCLRPDLTCKDLCEKNLLQRDSRCCQGWNT      180
tr|E5KCI4|E5KCI4_RHIMP              KLRTDGFIGATCDCGEWGAMNKTTRNCVPTTCLRPDLTCKDLCEKNLLQRDSRCCQGWNT      180
tr|A0A8E3CM27|A0A8E3CM27_RHIMP      KLRTDGFIGATCDCGEWGAMNKTTRNCVPTTCLRPDLTCKDLCEKNLLQRDSRCCQGWNT      180
tr|Q5XKU8|Q5XKU8_RHIMP              KLRTDGFIGATCDCGEWGAMNKTTRNCVPTTCLRPDLTCKDLCEKNLLQRDSRCCQGWNT      98
tr|Q5I6P1|Q5I6P1_RHIMP              KLRTDGFIGATCDCGEWGAMNKTTRNCVPTTCLRPDLTCKDLCEKNLLQRDSRCCQGWNT      98
tr|Q5ER85|Q5ER85_RHIMP              KLRTDGFIGATCDRGEWGAMNKTTRNCVPTTCLRPDLTCKDLCEKNLLQRDSRCCQGWNT      98
tr|Q5ER75|Q5ER75_RHIMP              KLRTDGFIGATCDCGEWGAMNKTTRNCVPTTCLRPDLTCKDLCEKNLLQRDSRCCQGRNT      98
tr|Q5ER64|Q5ER64_RHIMP              KLRTDGFIGATCDCGEWGAMNKTTRNCVPTMCLRPDLTCKDLCEKNLLQRDSRCCQGWNT      98
tr|E5KCI2|E5KCI2_RHIMP              KLRTDGFIGATCDCGEWGAMNKTTRNCVPTTCLRPDLTCKDLCEKNLLQRDSRCCQGWNT      180
tr|A0A8E2ZDL2|A0A8E2ZDL2_RHIMP      KLRTDGFIGATCDCGEWGAMNKTTRNCVPTTCLRPDLTCKDLCEKNLLQRDSRCCQGWNT      180
tr|A0A8E2ZDK4|A0A8E2ZDK4_RHIMP      KLRTDGFIGATCDCGEWGAMNKTTRNCVPTTCLRPDLTCKDLCEKNLLQRDSRCCQGWNT      180
tr|E5KCI6|E5KCI6_RHIMP              KLRTDGFIGATCDCGEWGAMNKTTRNCVPTTCLRPDLTCKDLCEKNLLQRDSRCCQGWNT      180
tr|E5KCI5|E5KCI5_RHIMP              KLRTDGFIGATCDCGEWGAMNKTTRNCVPTTCLRPDLTCKDLCEKNLLQRDSRCCQGWNT      180
tr|E5KCI1|E5KCI1_RHIMP              KLRTDGFIGATCDCGEWGAMNKTTRNCVPTTCLRPDLTCKDLCEKNLLQRDSRCCQGWNT      180
tr|E5KCI0|E5KCI0_RHIMP              KLRTDGFIGATCDCGEWGAMNKTTRNCVPTTCLRPDLTCKDLCEKNLLQRDSRCCQGWNT      180
tr|E5KCH9|E5KCH9_RHIMP              KLRTDGFIGATCDCGEWGAMNKTTRNCVPTTCLRPDLTCKDLCEKNLLQRDSRCCQGWNT      180
tr|E5KCH3|E5KCH3_RHIMP              KLRTDGFIGATCDCGEWGAMNKTTRNCVPTTCLRPDLTCKDLCEKNLLQRDSRCCQGWNT      180
tr|B6CNU3|B6CNU3_RHIMP              KLRTDGFIGATCDCGEWGAMNKTTRNCVPTTCLRPDLTCKDLCEKNLLQRDSRCCQGWNT      160
tr|A0A8E3CM42|A0A8E3CM42_RHIMP      KLRTDGFIGATCDCGEWGAMNKTTRNCVPTTCLRPDLTCKDLCEKNLLQRDSRCCQGWNT      180
tr|A0A8E3CM37|A0A8E3CM37_RHIMP      KLRTDGFIGATCDCGEWGAMNKTTRNCVPTTCLRPDLTCKDLCEKNLLQRDSRCCQGWNT      180
tr|A0A8E3CM32|A0A8E3CM32_RHIMP      KLRTDGFIGATCDCGEWGAMNKTTRNCVPTTCLRPDLTCKDLCEKNLLQRDSRCCQGWNT      180
tr|A0A8E3CLI5|A0A8E3CLI5_RHIMP      KLRTDGFIGATCDCGEWGAMNKTTRNCVPTTCLRPDLTCKDLCEKNLLQRDSRCCQGWNT      180
tr|A0A8E3CLI1|A0A8E3CLI1_RHIMP      KLRTDGFIGATCDCGEWGAMNKTTRNCVPTTCLRPDLTCKDLCEKNLLQRDSRCCQGWNT      180
tr|A0A8E3CK47|A0A8E3CK47_RHIMP      KLRTDGFIGATCDCGEWGAMNKTTRNCVPTTCLRPDLTCKDLCEKNLLQRDSRCCQGWNT      180
tr|A0A8E3CK39|A0A8E3CK39_RHIMP      KLRTDGFIGATCDCGEWGAMNKTTRNCVPTTCLRPDLTCKDLCEKNLLQRDSRCCQGWNT      180
tr|A0A8E3CHR0|A0A8E3CHR0_RHIMP      KLRTDGFIGATCDCGEWGAMNKTTRNCVPTTCLRPDLTCKDLCEKNLLQRDSRCCQGWNT      180
tr|A0A8E3CHQ5|A0A8E3CHQ5_RHIMP      KLRTDGFIGATCDCGEWGAMNKTTRNCVPTTCLRPDLTCKDLCEKNLLQRDSRCCQGWNT      180
tr|A0A8E2ZDN2|A0A8E2ZDN2_RHIMP      KLRTDGFIGATCDCGEWGAMNKTTRNCVPTTCLRPDLTCKDLCEKNLLQRDSRCCQGWNT      180
tr|A0A8E2ZC62|A0A8E2ZC62_RHIMP      KLRTDGFIGATCDCGEWGAMNKTTRNCVPTTCLRPDLTCKDLCEKNLLQRDSRCCQGWNT      180
tr|A0A8E2ZC54|A0A8E2ZC54_RHIMP      KLRTDGFIGATCDCGEWGAMNKTTRNCVPTTCLRPDLTCKDLCEKNLLQRDSRCCQGWNT      180
tr|A0A8E2ZC45|A0A8E2ZC45_RHIMP      KLRTDGFIGATCDCGEWGAMNKTTRNCVPTTCLRPDLTCKDLCEKNLLQRDSRCCQGWNT      180
tr|A0A8E2ZAC1|A0A8E2ZAC1_RHIMP      KLRTDGFIGATCDCGEWGAMNKTTRNCVPTTCLRPDLTCKDLCEKNLLQRDSRCCQGWNT      180
tr|A0A8E2ZAB3|A0A8E2ZAB3_RHIMP      KLRTDGFIGATCDCGEWGAMNKTTRNCVPTTCLRPDLTCKDLCEKNLLQRDSRCCQGWNT      180
tr|A0A8E2Z9N3|A0A8E2Z9N3_RHIMP      KLRTDGFIGATCDCGEWGAMNKTTRNCVPTTCLRPDLTCKDLCEKNLLQRDSRCCQGWNT      180
tr|A0A8E2Z9M6|A0A8E2Z9M6_RHIMP      KLRTDGFIGATCDCGEWGAMNKTTRNCVPTTCLRPDLTCKDLCEKNLLQRDSRCCQGWNT      180
tr|A0A8E2Z9L6|A0A8E2Z9L6_RHIMP      KLRTDGFIGATCDCGEWGAMNKTTRNCVPTTCLRPDLTCKDLCEKNLLQRDSRCCQGWNT      180
tr|A0A8E2Z8G3|A0A8E2Z8G3_RHIMP      KLRTDGFIGATCDCGEWGAMNKTTRNCVPTTCLRPDLTCKDLCEKNLLQRDSRCCQGWNT      180
tr|A0A8E2Z7X1|A0A8E2Z7X1_RHIMP      KLRTDGFIGATCDCGEWGAMNKTTRNCVPTTCLRPDLTCKDLCEKNLLQRDSRCCQGWNT      180
tr|A0A8E2Z7W9|A0A8E2Z7W9_RHIMP      KLRTDGFIGATCDCGEWGAMNKTTRNCVPTTCLRPDLTCKDLCEKNLLQRDSRCCQGWNT      180
tr|A0A8E2Z7W5|A0A8E2Z7W5_RHIMP      KLRTDGFIGATCDCGEWGAMNKTTRNCVPTTCLRPDLTCKDLCEKNLLQRDSRCCQGWNT      180
tr|A0A8E2Z7V4|A0A8E2Z7V4_RHIMP      KLRTDGFIGATCDCGEWGAMNKTTRNCVPTTCLRPDLTCKDLCEKNLLQRDSRCCQGWNT      180
tr|A0A8E2Z7U7|A0A8E2Z7U7_RHIMP      KLRTDGFIGATCDCGEWGAMNKTTRNCVPTTCLRPDLTCKDLCEKNLLQRDSRCCQGWNT      180
tr|A0A8E2Z7U5|A0A8E2Z7U5_RHIMP      KLRTDGFIGATCDCGEWGAMNKTTRNCVPTTCLRPDLTCKDLCEKNLLQRDSRCCQGWNT      180
tr|A0A8E2Z7U4|A0A8E2Z7U4_RHIMP      KLRTDGFIGATCDCGEWGAMNKTTRNCVPTTCLRPDLTCKDLCEKNLLQRDSRCCQGWNT      180
tr|A0A8E2Z7T7|A0A8E2Z7T7_RHIMP      KLRTDGFIGATCDCGEWGAMNKTTRNCVPTTCLRPDLTCKDLCEKNLLQRDSRCCQGWNT      180
tr|A0A8E2Z7S4|A0A8E2Z7S4_RHIMP      KLRTDGFIGATCDCGEWGAMNKTTRNCVPTTCLRPDLTCKDLCEKNLLQRDSRCCQGWNT      180
tr|A0A6M2CI36|A0A6M2CI36_RHIMP      KLRTDGFIGATCDCGEWGAMNKTTRNCVPTTCLRPDLTCKDLCEKNLLQRDSRCCQGWNT      180
tr|Q5XKV2|Q5XKV2_RHIMP              KLRTDGFIGATCDCGEWGAMNKTTRNCVPTTCLRPDLTCKDLCEKNLLQRDSRCCQGWNT      98
                                    ************* *****: ***:* *** **:***: ** ***:* ****** ** .*

tr|Q5ER80|Q5ER80_RHIMP              ANCLAAPPADSYCSPGSPKGPDGQCKNACRTKEAGFVCKHGCRSTDKAYECTRPSGSTVA      158
tr|A0A8E2Z927|A0A8E2Z927_RHIMP      ANCLAAPPADSYCSPGSPKGPDGQCKNACRTKEAGFVCKHGCRSTGKAYECTCPSGFTVA      240
tr|A0A8E2Z7V6|A0A8E2Z7V6_RHIMP      ANCLAAPPADSYCSPGSPKGPDGQCKNACRTKEAGFVCKHGCRSTDKAYECTCPSGFTVA      240
tr|A0A8E2Z7X7|A0A8E2Z7X7_RHIMP      ANCLAAPPADSYCSPGSPKGPDGQCKNACRTKEAGFVCKHGCRSTDKAYECTCPSGFTVA      240
tr|A0A8E3CL24|A0A8E3CL24_RHIMP      ANCLAAPPADSYCSPGSPKGPDGQCKNACRTKEAGFVCKHGCRSTGKAYECTCPSGFTVA      240
tr|A0A8E2Z8E9|A0A8E2Z8E9_RHIMP      ANCLAAPPADSYCSPGSPKGPDGQCKNACRTKEAGFVCKHGCRSTDKAYECTCPSGFTVA      240
tr|A0A8E2Z916|A0A8E2Z916_RHIMP      ANCLAAPPADSYCSPGSPKGPDGQCKNACRTKEAGFVCKHGCRSTDKAYECTCPSGFTVA      240
tr|A0A8E2Z7Z1|A0A8E2Z7Z1_RHIMP      ANCSAAPPADSYCSPGSPKGPDGQCKNACRTKEAGFVCKHGCRSTDKAYECTCPSGFTVA      240
tr|A0A8E3CLH7|A0A8E3CLH7_RHIMP      ANCSAAPPADSYCSPGSPKGPDGQCKNACRTKEAGFVCKHGCRSTDKAYECTCPSGFTVA      240
tr|A0A8E3CK42|A0A8E3CK42_RHIMP      ANCSAAPPADSYCSPGSPKGPDGQCKNACRTKEAGFVCKHGCRSTDKAYECTCPSGSTVA      240
tr|C4NAN0|C4NAN0_RHIMP              ANCSAAPPADSYCSPGSPKGPDGQCKNACRTKEAGFVCKHGCRSTDKAYECTCPSGSTVA      221
tr|E5KCH6|E5KCH6_RHIMP              ANCSAAPPADSYCSPGSPKGPDGQCKNACRTKEAGFVCKHGCRSTDKAYECTCPSGSTVA      240
tr|Q5XKU6|Q5XKU6_RHIMP              ANCSAAPPADSYCSPGSPKGPDGQCKNACRTKEAGFVCKHGCRSTDKAYECTCPSGSTVA      158
tr|Q5ER81|Q5ER81_RHIMP              ANCSAAPPADSYCSPGSPKGPDGRCKNACRTKEAGFVCKHGCRSTDKAYECTCPSGSTVA      158
tr|Q5ER93|Q5ER93_RHIMP              ANCSAAPPADSYCSPGSPKGPDGQCKNACRTKEAGFVCKHGCRSTDKAYECTCPSGSTVA      158
tr|Q5ER89|Q5ER89_RHIMP              ANCSAAPPADSYCSPGSPKGPDGQCKNACRTKEAGFVCKHGCRSTDKAYECTCPSGPTVA      158
tr|A0A8E2Z7V2|A0A8E2Z7V2_RHIMP      ANCLAAPPADSYCSPGSPKGPDGQCKNACRTKEAGFVCKHGCRSTDKAYECTCPSGSTVA      240
tr|Q5ER77|Q5ER77_RHIMP              ANCSAAPPADSYCSPGSPKGPDGQCKNACRTKEAGFVCKHGCRSTDKAYECTCPSGSTVA      158
tr|Q5ER78|Q5ER78_RHIMP              ANCSAAPPADSYCSPGSPKGPDGQCKNACRTKEAGFVCKHGCRSTDKAYECTCPSGSTVA      158
tr|Q5ER69|Q5ER69_RHIMP              ANCSAAPPADSYCSPGSPKGPDGQRKNACRTKEAGFVCKHGCRSTDKAYECTCPSGSTVA      158
tr|Q5XKU9|Q5XKU9_RHIMP              ANCSAAPPADSYCSPGSPKGPDGQCKNACRTKEAGFVCKHGCRSTDKAYECTCPSGSTVA      158
tr|Q9Y0V1|Q9Y0V1_RHIMP              ANCSAAPPADSYCSPGSPKGPDGQCKNACRTKEAGFVCKHGCRSTDKAYECTCPSGSTVA      240
tr|Q5XKU7|Q5XKU7_RHIMP              ANCSAAPPADSYCSPGSPKGPDGQCKNACRTKEAGFVCKHGCRSTDKAYECTCPSGSTVA      158
tr|Q5I6P2|Q5I6P2_RHIMP              ANCSAAPPADSYCSPGSPKGPDGQCKNACRTKEAGFVCKHGCRSTDKAYECTCPSGPTVA      158
tr|Q5ER92|Q5ER92_RHIMP              ANCSAAPPADSYCSPGSPKGPDGQCKNACRTKEAGFVCKHGCRSTDKAYECTCPSGSTVA      158
tr|Q5ER88|Q5ER88_RHIMP              ANCSAAPPADSYCSPGSPKGPDGQCKNACRTKEAGFVCKHGCRSTDKAYECTCPSGSTVA      158
tr|Q5ER82|Q5ER82_RHIMP              ANCSAAPPADSYCSPGSPKGPDGQCKYACRTKEAGFVCKHGCRSTDKAYECTCPSGSTVA      158
tr|A0A8E3CK52|A0A8E3CK52_RHIMP      ANCSAAPPADSYCSPGSPKGPDGQCKNACRTKEAGFVCKHGCRSTDKAYECTCPSGFTVA      240
tr|A0A8E2ZC71|A0A8E2ZC71_RHIMP      ANCSAAPPADSYCSPGSPKGPDGQCKNACRTKEAGFVCKHGCRSTDKAYECTCPSGFTVA      240
```

```
tr|A0A8E2Z937|A0A8E2Z937_RHIMP      ANCSAAPPADSYCSPGSPKGPDGQCKNACRTKEAGFVCKHGCRSTDKAYECTCPSGSTVA      240
tr|A0A8E2Z8F5|A0A8E2Z8F5_RHIMP      ANCLAAPPADSYCSPGSPKGPDGQCKNACRTKEAGFVCKHGCRSTDKAYECTCPSGSTVA      240
tr|A0A8E3CHP7|A0A8E3CHP7_RHIMP      ANCLAAPPADSYCSPGSPKGPDGQCKNACRTKEAGFVCKHGCRSTDKAYECTCPSGSTVA      240
tr|E5KCI4|E5KCI4_RHIMP              ANCLAAPPADSYCSPGSPKGPDGQCKNACRTKEAGFVCKHGCRSTDKAYECTCPSGSTVA      240
tr|A0A8E3CM27|A0A8E3CM27_RHIMP      ANCSAAPPADSYCSPGSPKGPDGQCKNACRTKEAGFVCKHGCRSTDKAYECTCPSGSTVA      240
tr|Q5XKU8|Q5XKU8_RHIMP              ANCSAAPPADSYCSPGSPKGPDGQCKNACRTKEAGFVCKHGCRSTDKAYECTCPSGSTVA      158
tr|Q5I6P1|Q5I6P1_RHIMP              ANCSAAPPADSYCSPGSPKGPDGQCKNACRTKEAGFVCKHGCRSTDKAYECTCPSGSTVA      158
tr|Q5ER85|Q5ER85_RHIMP              ANCSAAPPADSYCSPGSPKGPDGQCKNACRTKEAGFVCKHGCRSTDKAYECTCPSGSTVA      158
tr|Q5ER75|Q5ER75_RHIMP              ANCSAAPPADSYCSPGSPKGPDGQCKNACRTKEAGFVCKHGCRSTDKAYECTCPSGSTVA      158
tr|Q5ER64|Q5ER64_RHIMP              ANCSAAPPADSYCSPGSPKGPDGQCKNACRTKEAGFVCKHGCRSTDKAYECTCPSGSTVA      158
tr|E5KCI2|E5KCI2_RHIMP              ANCSAAPPADSYCSPGSPKGPDGQCKNACRTKEAGFVCKHGCRSTDKAYECTCPSGSTVA      240
tr|A0A8E2ZDL2|A0A8E2ZDL2_RHIMP      ANCSAAPPADSYCSPGSPKGPDGQCKNACRTKEAGFVCKHGCRSTDKAYECTCPSGSTVA      240
tr|A0A8E2ZDK4|A0A8E2ZDK4_RHIMP      ANCSAAPPADSYCSPGSPKGPDGQCKNACRTKEAGFVCKHGCRSTDKAYECTCPSGSTVA      240
tr|E5KCI6|E5KCI6_RHIMP              ANCSAAPPADSYCSPGSPKGPDGQCKNACRTKEAGFVCKHGCRSTDKAYECTCPSGSTVA      240
tr|E5KCI5|E5KCI5_RHIMP              ANCSAAPPADSYCSPGSPKGPDGQCKNACRTKEAGFVCKHGCRSTDKAYECTCPSGSTVA      240
tr|E5KCI1|E5KCI1_RHIMP              ANCSAAPPADSYCSPGSPKGPDGQCKNACRTKEAGFVCKHGCRSTDKAYECTCPSGSTVA      240
tr|E5KCI0|E5KCI0_RHIMP              ANCSAAPPADSYCSPGSPKGPDGQCKNACRTKEAGFVCKHGCRSTDKAYECTCPSGSTVA      240
tr|E5KCH9|E5KCH9_RHIMP              ANCSAAPPADSYCSPGSPKGPDGQCKNACRTKEAGFVCKHGCRSTDKAYECTCPSGSTVA      240
tr|E5KCH3|E5KCH3_RHIMP              ANCSAAPPADSYCSPGSPKGPDGQCKNACRTKEAGFVCKHGCRSTDKAYECTCPSGSTVA      240
tr|B6CNU3|B6CNU3_RHIMP              ANCSAAPPADSYCSPGSPKGPDGQCKNACRTKEAGFVCKHGCRSTDKAYECTCPSGSTVA      220
tr|A0A8E3CM42|A0A8E3CM42_RHIMP      ANCSAAPPADSYCSPGSPKGPDGQCKNACRTKEAGFVCKHGCRSTDKAYECTCPSGSTVA      240
tr|A0A8E3CM37|A0A8E3CM37_RHIMP      ANCSAAPPADSYCSPGSPKGPDGQCKNACRTKEAGFVCKHGCRSTDKAYECTCPSGSTVA      240
tr|A0A8E3CM32|A0A8E3CM32_RHIMP      ANCSAAPPADSYCSPGSPKGPDGQCKNACRTKEAGFVCKHGCRSTDKAYECTCPSGSTVA      240
tr|A0A8E3CLI5|A0A8E3CLI5_RHIMP      ANCSAAPPADSYCSPGSPKGPDGQCKNACRTKEAGFVCKHGCRSTDKAYECTCPSGSTVA      240
tr|A0A8E3CLI1|A0A8E3CLI1_RHIMP      ANCSAAPPADSYCSPGSPKGPDGQCKNACRTKEAGFVCKHGCRSTDKAYECTCPSGSTVA      240
tr|A0A8E3CK47|A0A8E3CK47_RHIMP      ANCSAAPPADSYCSPGSPKGPDGQCKNACRTKEAGFVCKHGCRSTDKAYECTCPSGSTVA      240
tr|A0A8E3CK39|A0A8E3CK39_RHIMP      ANCSAAPPADSYCSPGSPKGPDGQCKNACRTKEAGFVCKHGCRSTDKAYECTCPSGSTVA      240
tr|A0A8E3CHR0|A0A8E3CHR0_RHIMP      ANCSAAPPADSYCSPGSPKGPDGQCKNACRTKEAGFVCKHGCRSTDKAYECTCPSGSTVA      240
tr|A0A8E3CHQ5|A0A8E3CHQ5_RHIMP      ANCSAAPPADSYCSPGSPKGPDGQCKNACRTKEAGFVCKHGCRSTDKAYECTCPSGSTVA      240
tr|A0A8E2ZDN2|A0A8E2ZDN2_RHIMP      ANCSAAPPADSYCSPGSPKGPDGQCKNACRTKEAGFVCKHGCRSTDKAYECTCPSGSTVA      240
tr|A0A8E2ZC62|A0A8E2ZC62_RHIMP      ANCSAAPPADSYCSPGSPKGPDGQCKNACRTKEAGFVCKHGCRSTDKAYECTCPSGSTVA      240
tr|A0A8E2ZC54|A0A8E2ZC54_RHIMP      ANCSAAPPADSYCSPGSPKGPDGQCKNACRTKEAGFVCKHGCRSTDKAYECTCPSGSTVA      240
tr|A0A8E2ZC45|A0A8E2ZC45_RHIMP      ANCSAAPPADSYCSPGSPKGPDGQCKNACRTKEAGFVCKHGCRSTDKAYECTCPSGSTVA      240
tr|A0A8E2ZAC1|A0A8E2ZAC1_RHIMP      ANCSAAPPADSYCSPGSPKGPDGQCKNACRTKEAGFVCKHGCRSTDKAYECTCPSGSTVA      240
tr|A0A8E2ZAB3|A0A8E2ZAB3_RHIMP      ANCSAAPPADSYCSPGSPKGPDGQCKNACRTKEAGFVCKHGCRSTDKAYECTCPSGSTVA      240
tr|A0A8E2Z9N3|A0A8E2Z9N3_RHIMP      ANCSAAPPADSYCSPGSPKGPDGQCKNACRTKEAGFVCKHGCRSTDKAYECTCPSGSTVA      240
tr|A0A8E2Z9M6|A0A8E2Z9M6_RHIMP      ANCSAAPPADSYCSPGSPKGPDGQCKNACRTKEAGFVCKHGCRSTDKAYECTCPSGSTVA      240
tr|A0A8E2Z9L6|A0A8E2Z9L6_RHIMP      ANCSAAPPADSYCSPGSPKGPDGQCKNACRTKEAGFVCKHGCRSTDKAYECTCPSGSTVA      240
tr|A0A8E2Z8G3|A0A8E2Z8G3_RHIMP      ANCSAAPPADSYCSPGSPKGPDGQCKNACRTKEAGFVCKHGCRSTDKAYECTCPSGSTVA      240
tr|A0A8E2Z7X1|A0A8E2Z7X1_RHIMP      ANCSAAPPADSYCSPGSPKGPDGQCKNACRTKEAGFVCKHGCRSTDKAYECTCPSGSTVA      240
tr|A0A8E2Z7W9|A0A8E2Z7W9_RHIMP      ANCSAAPPADSYCSPGSPKGPDGQCKNACRTKEAGFVCKHGCRSTDKAYECTCPSGSTVA      240
tr|A0A8E2Z7W5|A0A8E2Z7W5_RHIMP      ANCSAAPPADSYCSPGSPKGPDGQCKNACRTKEAGFVCKHGCRSTDKAYECTCPSGSTVA      240
tr|A0A8E2Z7V4|A0A8E2Z7V4_RHIMP      ANCSAAPPADSYCSPGSPKGPDGQCKNACRTKEAGFVCKHGCRSTDKAYECTCPSGSTVA      240
tr|A0A8E2Z7U7|A0A8E2Z7U7_RHIMP      ANCSAAPPADSYCSPGSPKGPDGQCKNACRTKEAGFVCKHGCRSTDKAYECTCPSGSTVA      240
tr|A0A8E2Z7U5|A0A8E2Z7U5_RHIMP      ANCSAAPPADSYCSPGSPKGPDGQCKNACRTKEAGFVCKHGCRSTDKAYECTCPSGSTVA      240
tr|A0A8E2Z7U4|A0A8E2Z7U4_RHIMP      ANCSAAPPADSYCSPGSPKGPDGQCKNACRTKEAGFVCKHGCRSTDKAYECTCPSGSTVA      240
tr|A0A8E2Z7T7|A0A8E2Z7T7_RHIMP      ANCSAAPPADSYCSPGSPKGPDGQCKNACRTKEAGFVCKHGCRSTDKAYECTCPSGSTVA      240
tr|A0A8E2Z7S4|A0A8E2Z7S4_RHIMP      ANCSAAPPADSYCSPGSPKGPDGQCKNACRTKEAGFVCKHGCRSTDKAYECTCPSGSTVA      240
tr|A0A6M2CI36|A0A6M2CI36_RHIMP      ANCSAAPPADSYCSPGSPKGPDGQCKNACRTKEAGFVCKHGCRSTDKAYECTCPSGSTVA      240
tr|Q5XKV2|Q5XKV2_RHIMP              ANCSAAPPADSYCSPGSPKGPDGQCKNACRTKEAGFVCKHGCRSTDKAYECTCPSGSTVA      158
                                    *** *******************: * ******************.****** *** ***

tr|Q5ER80|Q5ER80_RHIMP              EDGITCKSISYTVSCTVEQKQTCRPTEDCRVQKGTVLCECPWNQHLVGDTCISDCVDKKC      218
tr|A0A8E2Z927|A0A8E2Z927_RHIMP      EDGITCKSISYTVSCTVEQKQTCRPTEDCRVQKGTVLCECPWNQHLVGDTCISDCVDKKC      300
tr|A0A8E2Z7V6|A0A8E2Z7V6_RHIMP      EDGITCKSISYTVSCTVEQKQTCRPTEDCRVQKGTVLCECPWNQHLVGDTCISDCVDKKC      300
tr|A0A8E2Z7X7|A0A8E2Z7X7_RHIMP      EDGITCKSISYTVSCTVEQKQTCRPTEDCRVQKGTVLCECPWNQHLVGDTCISDCVDKKC      300
tr|A0A8E3CL24|A0A8E3CL24_RHIMP      EDGITCKSISYTVSCTVEQKQTCRPTEDCRVQKGTVLCECPWNQHLVGDTCISDCVDKKC      300
tr|A0A8E2Z8E9|A0A8E2Z8E9_RHIMP      EDGITCKSISYTVSCTVEQKQTCRPTEDCRVQKGTVLCECPWNQHLVGDTCISDCVDKKC      300
tr|A0A8E2Z916|A0A8E2Z916_RHIMP      EDGITCKSISYTVSCTVEQKQTCRPTEDCRVQKGTVLCECPWNQHLVGDTCISDCVDKKC      300
tr|A0A8E2Z7Z1|A0A8E2Z7Z1_RHIMP      EDGITCKSISYTVSCTVEQKQTCRPTEDCRVQKGTVLCECPWNQHLVGDTCISDCVDKKC      300
tr|A0A8E3CLH7|A0A8E3CLH7_RHIMP      EDGITCKSISYTVSCTVEQKQTCRPTEDCRVQKGTVLCECPWNQHLVGDTCISDCVDKKC      300
tr|A0A8E3CK42|A0A8E3CK42_RHIMP      EDGITCKSISYTVGCTVEQKQTCRPTEDCRVQKGTVLCECPWNQHLVGDTCISDCVDKKC      300
tr|C4NAN0|C4NAN0_RHIMP              EDGITCKSISYTVSCTVEQKQTCRPTEDCRVQKGTVLCECPWNQHLVGDTCISDCVDKKC      281
tr|E5KCH6|E5KCH6_RHIMP              EDGITCKSISYTVSCTVEQKQTCRPTEDCRVQKGTVLCECPWNQHLVGDTCISDCVDKKC      300
tr|Q5XKU6|Q5XKU6_RHIMP              EDGITCKSISYTVSCIVEQKQTCRPTEDCRVQKGTVLCECPWNQHLVGDTCISDCVDKKC      218
tr|Q5ER81|Q5ER81_RHIMP              EDGITCESISYTVSCTVEQKQTCRPTEDCRVQKGTVLCECPWNQHLVGDTCISDCVDKKC      218
tr|Q5ER93|Q5ER93_RHIMP              EDGITCKSISYTVSCTVEQKQTCRPTEDCRVQKGTVLCECPWNQHLVGDTCISDCVDKKC      218
tr|Q5ER89|Q5ER89_RHIMP              EDGITCKSISYTVSCTVEQKQTCRPTEDCRVQKGTVLCECPWNQHLVGDTCISDCVDKKC      218
tr|A0A8E2Z7V2|A0A8E2Z7V2_RHIMP      EDGITCKSISYTVSCTVEQKQTCRPTEDCRVQKGTVLCECPWNQHLVGDTCISDCVDKKC      300
tr|Q5ER77|Q5ER77_RHIMP              EDGITCKSISYTVSCTVEQKQTCRPTEDCRVQKGTVLCECPWNQHLVGDTCISDCVDKKC      218
tr|Q5ER78|Q5ER78_RHIMP              EDGITCKSISYTVSCTVEQKQTCRPTEDCRVQKGTVLCECPWNQHLVGDTCISDCVDKKC      218
tr|Q5ER69|Q5ER69_RHIMP              EDGITCKSISYTVSCTVEQKQTCRPTEDCRVQKGTVLCECPWNQHLVGDTCISDCVDKKC      218
tr|Q5XKU9|Q5XKU9_RHIMP              EDGITCKSISYTVSCTVEQKQTCRPTEDCRVQKGTVLCECPWNQHLVGDTCISDCVDKKC      218
tr|Q9Y0V1|Q9Y0V1_RHIMP              EDGITCKSISYTVSCTVEQKQTCRPTEDCRVQKGTVLCECPWNQHLVGDTCISDCVDKKC      300
tr|Q5XKU7|Q5XKU7_RHIMP              EDGITCKSISYTVSCTVEQKQTCRPTEDCRVQKGTVLCECPWNQHLVGDTCISDCVDKKC      218
tr|Q5I6P2|Q5I6P2_RHIMP              EDGITCKSISYTVSCTVEQKQTCRPTEDCRVQKGTVLCECPWNQHLVGDTCISDCVDKKC      218
tr|Q5ER92|Q5ER92_RHIMP              EDGITCKSISYTVSCTVEQKQTCRPTEDCRVQKGTVLCECPWNQHLVGDTCISDCVDKKC      218
tr|Q5ER88|Q5ER88_RHIMP              EDGITCKSISYTVSCTVEQKQTCRPTEDCRVQKGTVLCECPWNQHLVGDTCISDCVDKKC      218
tr|Q5ER82|Q5ER82_RHIMP              EDGITCKSISYTVSCTVEQKQTCRPTEDCRVQKGTVLCECPWNQHLVGDTCISDCVDKKC      218
tr|A0A8E3CK52|A0A8E3CK52_RHIMP      EDGITCKSISYTVGCTVEQKQTCRPTEDCRVQKGTVLCECPWNQHLVGDTCISDCVDKKC      300
tr|A0A8E2ZC71|A0A8E2ZC71_RHIMP      EDGITCKSISYTVGCTVEQKQTCRPTEDCRVQKGTVLCECPWNQHLVGDTCISDCVDKKC      300
tr|A0A8E2Z937|A0A8E2Z937_RHIMP      EDGITCKSISYTVSCTVEQKQTCRPTEDCRVQKGTVLCECPWNQHLVGDTCISDCVDKKC      300
tr|A0A8E2Z8F5|A0A8E2Z8F5_RHIMP      EDGITCKSISYTVSCTVEQKQTCRPTEDCRVQKGTVLCECPWNQHLVGDTCISDCVDKKC      300
tr|A0A8E3CHP7|A0A8E3CHP7_RHIMP      EDGITCKSISYTVSCTVEQKQTCRPTEDCRVQKGTVLCECPWNQHLVGDTCISDCVDKKC      300
tr|E5KCI4|E5KCI4_RHIMP              EDGITCKSISYTVSCTVEQKQTCRPTEDCRVQKGTVLCECPWNQHLVGDTCISDCVDKKC      300
tr|A0A8E3CM27|A0A8E3CM27_RHIMP      EDGITCKSISYTVSCTVEQKQTCRPTEDCRVQKGTVLCECPWNQHLVGDTCISDCVDKKC      300
tr|Q5XKU8|Q5XKU8_RHIMP              EDGITCKSISYTVSCTVEQKQTCRPTEDCRVQKGTVLCECPWNQHLVGDTCISDCVDKKC      218
tr|Q5I6P1|Q5I6P1_RHIMP              EDGITCKSISYTVSCTVEQKQTCRPTEDCRVQKGTVLCECPWNQHLVGDTCISDCVDKKC      218
tr|Q5ER85|Q5ER85_RHIMP              EDGITCKSISYTVSCTVEQKQTCRPTEDCRVQKGTVLCECPWNQHLVGDTCISDCVDKKC      218
tr|Q5ER75|Q5ER75_RHIMP              EDGITCKSISYTVSCTVEQKQTCRPTEDCRVQKGTVLCECPWNQHLVGDTCISDCVDKKC      218
tr|Q5ER64|Q5ER64_RHIMP              EDGITCKSISYTVSCTVEQKQTCRPTEDCRVQKGTVLCECPWNQHLVGDTCISDCVDKKC      218
tr|E5KCI2|E5KCI2_RHIMP              EDGITCKSISYTVSCTVEQKQTCRPTEDCRVQKGTVLCECPWNQHLVGDTCISDCVDKKC      300
tr|A0A8E2ZDL2|A0A8E2ZDL2_RHIMP      EDGITCKSISYTVSCTVEQKQTCRPTEDCRVQKGTVLCECPWNQHLVGDTCISDCVDKKC      300
tr|A0A8E2ZDK4|A0A8E2ZDK4_RHIMP      EDGITCKSISYTVSCTVEQKQTCRPTEDCRVQKGTVLCECPWNQHLVGDTCISDCVDKKC      300
```

```
tr|E5KCI6|E5KCI6_RHIMP                   EDGITCKSISYTVSCTVEQKQTCRPTEDCRVQKGTVLCECPWNQHLVGDTCISDCVDKKC        300
tr|E5KCI5|E5KCI5_RHIMP                   EDGITCKSISYTVSCTVEQKQTCRPTEDCRVQKGTVLCECPWNQHLVGDTCISDCVDKKC        300
tr|E5KCI1|E5KCI1_RHIMP                   EDGITCKSISYTVSCTVEQKQTCRPTEDCRVQKGTVLCECPWNQHLVGDTCISDCVDKKC        300
tr|E5KCI0|E5KCI0_RHIMP                   EDGITCKSISYTVSCTVEQKQTCRPTEDCRVQKGTVLCECPWNQHLVGDTCISDCVDKKC        300
tr|E5KCH9|E5KCH9_RHIMP                   EDGITCKSISYTVSCTVEQKQTCRPTEDCRVQKGTVLCECPWNQHLVGDTCISDCVDKKC        300
tr|E5KCH3|E5KCH3_RHIMP                   EDGITCKSISYTVSCTVEQKQTCRPTEDCRVQKGTVLCECPWNQHLVGDTCISDCVDKKC        300
tr|B6CNU3|B6CNU3_RHIMP                   EDGITCKSISYTVSCTVEQKQTCRPTEDCRVQKGTVLCECPWNQHLVGDTCISDCVDKKC        280
tr|A0A8E3CM42|A0A8E3CM42_RHIMP           EDGITCKSISYTVSCTVEQKQTCRPTEDCRVQKGTVLCECPWNQHLVGDTCISDCVDKKC        300
tr|A0A8E3CM37|A0A8E3CM37_RHIMP           EDGITCKSISYTVSCTVEQKQTCRPTEDCRVQKGTVLCECPWNQHLVGDTCISDCVDKKC        300
tr|A0A8E3CM32|A0A8E3CM32_RHIMP           EDGITCKSISYTVSCTVEQKQTCRPTEDCRVQKGTVLCECPWNQHLVGDTCISDCVDKKC        300
tr|A0A8E3CLI5|A0A8E3CLI5_RHIMP           EDGITCKSISYTVSCTVEQKQTCRPTEDCRVQKGTVLCECPWNQHLVGDTCISDCVDKKC        300
tr|A0A8E3CLI1|A0A8E3CLI1_RHIMP           EDGITCKSISYTVSCTVEQKQTCRPTEDCRVQKGTVLCECPWNQHLVGDTCISDCVDKKC        300
tr|A0A8E3CK47|A0A8E3CK47_RHIMP           EDGITCKSISYTVSCTVEQKQTCRPTEDCRVQKGTVLCECPWNQHLVGDTCISDCVDKKC        300
tr|A0A8E3CK39|A0A8E3CK39_RHIMP           EDGITCKSISYTVSCTVEQKQTCRPTEDCRVQKGTVLCECPWNQHLVGDTCISDCVDKKC        300
tr|A0A8E3CHR0|A0A8E3CHR0_RHIMP           EDGITCKSISYTVSCTVEQKQTCRPTEDCRVQKGTVLCECPWNQHLVGDTCISDCVDKKC        300
tr|A0A8E3CHQ5|A0A8E3CHQ5_RHIMP           EDGITCKSISYTVSCTVEQKQTCRPTEDCRVQKGTVLCECPWNQHLVGDTCISDCVDKKC        300
tr|A0A8E2ZDN2|A0A8E2ZDN2_RHIMP           EDGITCKSISYTVSCTVEQKQTCRPTEDCRVQKGTVLCECPWNQHLVGDTCISDCVDKKC        300
tr|A0A8E2ZC62|A0A8E2ZC62_RHIMP           EDGITCKSISYTVSCTVEQKQTCRPTEDCRVQKGTVLCECPWNQHLVGDTCISDCVDKKC        300
tr|A0A8E2ZC54|A0A8E2ZC54_RHIMP           EDGITCKSISYTVSCTVEQKQTCRPTEDCRVQKGTVLCECPWNQHLVGDTCISDCVDKKC        300
tr|A0A8E2ZC45|A0A8E2ZC45_RHIMP           EDGITCKSISYTVSCTVEQKQTCRPTEDCRVQKGTVLCECPWNQHLVGDTCISDCVDKKC        300
tr|A0A8E2ZAC1|A0A8E2ZAC1_RHIMP           EDGITCKSISYTVSCTVEQKQTCRPTEDCRVQKGTVLCECPWNQHLVGDTCISDCVDKKC        300
tr|A0A8E2ZAB3|A0A8E2ZAB3_RHIMP           EDGITCKSISYTVSCTVEQKQTCRPTEDCRVQKGTVLCECPWNQHLVGDTCISDCVDKKC        300
tr|A0A8E2Z9N3|A0A8E2Z9N3_RHIMP           EDGITCKSISYTVSCTVEQKQTCRPTEDCRVQKGTVLCECPWNQHLVGDTCISDCVDKKC        300
tr|A0A8E2Z9M6|A0A8E2Z9M6_RHIMP           EDGITCKSISYTVSCTVEQKQTCRPTEDCRVQKGTVLCECPWNQHLVGDTCISDCVDKKC        300
tr|A0A8E2Z9L6|A0A8E2Z9L6_RHIMP           EDGITCKSISYTVSCTVEQKQTCRPTEDCRVQKGTVLCECPWNQHLVGDTCISDCVDKKC        300
tr|A0A8E2Z8G3|A0A8E2Z8G3_RHIMP           EDGITCKSISYTVSCTVEQKQTCRPTEDCRVQKGTVLCECPWNQHLVGDTCISDCVDKKC        300
tr|A0A8E2Z7X1|A0A8E2Z7X1_RHIMP           EDGITCKSISYTVSCTVEQKQTCRPTEDCRVQKGTVLCECPWNQHLVGDTCISDCVDKKC        300
tr|A0A8E2Z7W9|A0A8E2Z7W9_RHIMP           EDGITCKSISYTVSCTVEQKQTCRPTEDCRVQKGTVLCECPWNQHLVGDTCISDCVDKKC        300
tr|A0A8E2Z7W5|A0A8E2Z7W5_RHIMP           EDGITCKSISYTVSCTVEQKQTCRPTEDCRVQKGTVLCECPWNQHLVGDTCISDCVDKKC        300
tr|A0A8E2Z7V4|A0A8E2Z7V4_RHIMP           EDGITCKSISYTVSCTVEQKQTCRPTEDCRVQKGTVLCECPWNQHLVGDTCISDCVDKKC        300
tr|A0A8E2Z7U7|A0A8E2Z7U7_RHIMP           EDGITCKSISYTVSCTVEQKQTCRPTEDCRVQKGTVLCECPWNQHLVGDTCISDCVDKKC        300
tr|A0A8E2Z7U5|A0A8E2Z7U5_RHIMP           EDGITCKSISYTVSCTVEQKQTCRPTEDCRVQKGTVLCECPWNQHLVGDTCISDCVDKKC        300
tr|A0A8E2Z7U4|A0A8E2Z7U4_RHIMP           EDGITCKSISYTVSCTVEQKQTCRPTEDCRVQKGTVLCECPWNQHLVGDTCISDCVDKKC        300
tr|A0A8E2Z7T7|A0A8E2Z7T7_RHIMP           EDGITCKSISYTVSCTVEQKQTCRPTEDCRVQKGTVLCECPWNQHLVGDTCISDCVDKKC        300
tr|A0A8E2Z7S4|A0A8E2Z7S4_RHIMP           EDGITCKSISYTVSCTVEQKQTCRPTEDCRVQKGTVLCECPWNQHLVGDTCISDCVDKKC        300
tr|A0A6M2CI36|A0A6M2CI36_RHIMP           EDGITCKSISYTVSCTVEQKQTCRPTEDCRVQKGTVLCECPWNQHLVGDTCISDCVDKKC        300
tr|Q5XKV2|Q5XKV2_RHIMP                   EDGITCKSISYTVSCTVEQKQTCRPTEDCRVQKGTVLCECPWNQHLVGDTCISDCVDKKC        218
                                         ******:******.* ********************************************

tr|Q5ER80|Q5ER80_RHIMP                   HEEFMDCGVYMNRRSCYCPWKSRKPGPNVNINECLLNEYYYTLSFT--------------        264
tr|A0A8E2Z927|A0A8E2Z927_RHIMP           HEEFMDCGVYMNRQSCYCPWKSRKPGPNVNINECLLNEYYYTVSFTPNISFDSDHCKRYE        360
tr|A0A8E2Z7V6|A0A8E2Z7V6_RHIMP           HEEFMDCGVYMNRQSCYCPWKSRKPGPNVNINECLLNEYYYTVSFTPNISFDSDHCKRYE        360
tr|A0A8E2Z7X7|A0A8E2Z7X7_RHIMP           HEEFMDCGVYMNRQSCYCPWKSRKPGPNVNINECLLNEYYYTVSFTPNISFDSDHCKRYE        360
tr|A0A8E3CL24|A0A8E3CL24_RHIMP           HEEFMDCGVYMNRQSCYCPWKSRKPGPNVNINECLLNEYYYTVSFTPNISFDSDHCKRYE        360
tr|A0A8E2Z8E9|A0A8E2Z8E9_RHIMP           HEEFMDCGVYMNRQSCYCPWKSRKPGPNVNINECLLNEYYYTVSFTPNISFDSDHCKRYE        360
tr|A0A8E2Z916|A0A8E2Z916_RHIMP           HEEFMDCGVYMNRQSCYCPWKSRKPGPNVNINECLLNEYYYTVSFTPNISFDSDHCKRYE        360
tr|A0A8E2Z7Z1|A0A8E2Z7Z1_RHIMP           HEEFMDCGVYMNRQSCYCPWKSRKPGPNVNINECLLNEYYYTVSFTPNISFDSDHCKRYE        360
tr|A0A8E3CLH7|A0A8E3CLH7_RHIMP           HEEFMDCGVYMNRQSCYCPWKSRKPGPNVNINECLLNEYYYTVSFTPNISFDSDHCKRYE        360
tr|A0A8E3CK42|A0A8E3CK42_RHIMP           HEEFMDCGVYMNRQSCYCPWKSRKPGPNVNINECLLNEYYYTVSFTPNISFDSDHCKRYE        360
tr|C4NAN0|C4NAN0_RHIMP                   HEEFMDCGVYMNRQSCYCPWKSRKPGPNVNINECLLNEYYYTVSFTPNISFDSDHCKRYE        341
tr|E5KCH6|E5KCH6_RHIMP                   HEEFMDCGVYMNRQSCYCPWKSRKPGPNVNINECLLNEYYYTVSFTPNISFDSDHCKRYE        360
tr|Q5XKU6|Q5XKU6_RHIMP                   HEEFMDCGAYMNRQSCYRPWKSRKPGPNVNINECLLNEYYYTVSFT--------------        264
tr|Q5ER81|Q5ER81_RHIMP                   HEEFMDCGVYMNRQSCYCPWKSRKPGPNVNINKCLLNEYYYTVSFT--------------        264
tr|Q5ER93|Q5ER93_RHIMP                   HEEFMDCGVYMNRQSCYCPWKSRKPGPNVDINECLLNEYYYTVSFT--------------        264
tr|Q5ER89|Q5ER89_RHIMP                   HEEFMDCGVYMNRQSCYCPWKSRKPGPNVNINECLLNEYYYTVSFT--------------        264
tr|A0A8E2Z7V2|A0A8E2Z7V2_RHIMP           HEEFMDCGVYMNRQSCYCPWKSRKPGPNVNINECLLNEYYYTVSFTPNISFDSDHCKRYE        360
tr|Q5ER77|Q5ER77_RHIMP                   HEEFMDCGVYMNRQRCYCPWKSRKPGPNVNINECLLNEYYYTVSFT--------------        264
tr|Q5ER78|Q5ER78_RHIMP                   HEEFMDCGVYMNRQRCYCPWKSRKPGPNVNINECLLNEYYYTVSFT--------------        264
tr|Q5ER69|Q5ER69_RHIMP                   HEEFMDCGVYMNRQSCYCPWKSRKPGPNVNINECLLNEYYYTVSFT--------------        264
tr|Q5XKU9|Q5XKU9_RHIMP                   HEEFMDCGVYMNRQSCYCPWKSRKPGPNVNINECLLNEYYYTVSFT--------------        264
tr|Q9Y0V1|Q9Y0V1_RHIMP                   HEEFMDCGVYMNRQSCYCPWKSRKPGPNVNINERLLNEYYYTVSFTPNISFDSDHCKRYE        360
tr|Q5XKU7|Q5XKU7_RHIMP                   HEEFMDCGVYMNRQSCYCPWKSRKPGPNVNINECLLNEYYYTVSFT--------------        264
tr|Q5I6P2|Q5I6P2_RHIMP                   HEEFMDCGVYMNRRSCYRPWKSRKPGPNVNINECLLNEYYYTVSFT--------------        264
tr|Q5ER92|Q5ER92_RHIMP                   HEEFMDCGVYMNRQSCYRPWKSRKPGPNVNINECLLNEYYYTVSFT--------------        264
tr|Q5ER88|Q5ER88_RHIMP                   HEEFMDCGVYMNRQSCYCPWKSRKPGPNVNINECLLNEYYYTVSFT--------------        264
tr|Q5ER82|Q5ER82_RHIMP                   HEEFMDCGVYMNRQSCYCPWKSRKPGPNVNIDECLLNEYYYTVSFT--------------        264
tr|A0A8E3CK52|A0A8E3CK52_RHIMP           HEEFMDCGVYMNRQSCYCPWKSRKPGPNVNINECLLNEYYYTVSFTPNISLDSDHCEWYE        360
tr|A0A8E2ZC71|A0A8E2ZC71_RHIMP           HEEFMDCGVYMNRQSCYCPWKSRKPGPNVNINECLLNEYYYTVSFTPNISLDSDHCEWYE        360
tr|A0A8E2Z937|A0A8E2Z937_RHIMP           HEEFMDCGVYMNRQSCYCPWKSRKPGPNVNINECLLNEYYYTVSFTPNISFDSDHCKRYE        360
tr|A0A8E2Z8F5|A0A8E2Z8F5_RHIMP           HEEFMDCGVYMNRQSCYCPWKSRKPGPNVNINECLLNEYYYTVSFTPNISFDSDHCKRYE        360
tr|A0A8E3CHP7|A0A8E3CHP7_RHIMP           HEEFMDCGVYMNRQSCYCPWKSRKPGPNVNINECLLNEYYYTVSFTPNISFDSDHCKRYE        360
tr|E5KCI4|E5KCI4_RHIMP                   HEEFMDCGVYMNRQSCYCPWKSRKPGPNVNINECLLNEYYYTVSFTPNISFDSDHCKRYE        360
tr|A0A8E3CM27|A0A8E3CM27_RHIMP           HEEFMDCGVYMNRQSCYCPWKSRKPGPNVNINECLLNEYYYTVSFTPNISFDSDHCKRYE        360
tr|Q5XKU8|Q5XKU8_RHIMP                   HEEFMDCGVYMNRQSCYCPWKSRKPGANVNINECLLNEYYYTVSFT--------------        264
tr|Q5I6P1|Q5I6P1_RHIMP                   HEEFMDCGVYMNRQSCYRPWKSRKPGPNVNINECLLNEYYYTVSFT--------------        264
tr|Q5ER85|Q5ER85_RHIMP                   HEEFMDCGVYMNRQSCYCPWKSRKPGPNVNINECLLNEYYYTVSFT--------------        264
tr|Q5ER75|Q5ER75_RHIMP                   HEEFMDCGVYMNRQSCYCPWKSRKPGPNVNINECLLNEYYYTVSFT--------------        264
tr|Q5ER64|Q5ER64_RHIMP                   HEEFMDCGVYMNRQSCYCPWKSRKPGPNVNINECLLNEYYYTVSFT--------------        264
tr|E5KCI2|E5KCI2_RHIMP                   HEEFMDCGVYMNRQSCYCPWKSRKPGPNVNINECLLNEYYYTVSFTPNISFDSDHCKRYE        360
tr|A0A8E2ZDL2|A0A8E2ZDL2_RHIMP           HEEFMDCGVYMNRQSCYCPWKSRKPGPKVNINECLLNEYYYTVSFTPNISFDSDHCKRYE        360
tr|A0A8E2ZDK4|A0A8E2ZDK4_RHIMP           HEEFMDCGVYMNRQSCYCPWKSRKPGPKVNINECLLNEYYYTVSFTPNISFDSDHCKRYE        360
tr|E5KCI6|E5KCI6_RHIMP                   HEEFMDCGVYMNRQSCYCPWKSRKPGPNVNINECLLNEYYYTVSFTPNISFDSDHCKRYE        360
tr|E5KCI5|E5KCI5_RHIMP                   HEEFMDCGVYMNRQSCYCPWKSRKPGPNVNINECLLNEYYYTVSFTPNISFDSDHCKRYE        360
tr|E5KCI1|E5KCI1_RHIMP                   HEEFMDCGVYMNRQSCYCPWKSRKPGPNVNINECLLNEYYYTVSFTPNISFDSDHCKRYE        360
tr|E5KCI0|E5KCI0_RHIMP                   HEEFMDCGVYMNRQSCYCPWKSRKPGPNVNINECLLNEYYYTVSFTPNISFDSDHCKRYE        360
tr|E5KCH9|E5KCH9_RHIMP                   HEEFMDCGVYMNRQSCYCPWKSRKPGPNVNINECLLNEYYYTVSFTPNISFDSDHCKRYE        360
tr|E5KCH3|E5KCH3_RHIMP                   HEEFMDCGVYMNRQSCYCPWKSRKPGPNVNINECLLNEYYYTVSFTPNISFDSDHCKRYE        360
tr|B6CNU3|B6CNU3_RHIMP                   HEEFMDCGVYMNRQSCYCPWKSRKPGPNVNINECLLNEYYYTVSFTPNISFDSDHCKRYE        340
tr|A0A8E3CM42|A0A8E3CM42_RHIMP           HEEFMDCGVYMNRQSCYCPWKSRKPGPNVNINECLLNEYYYTVSFTPNISLDSDHCDWYE        360
tr|A0A8E3CM37|A0A8E3CM37_RHIMP           HEEFMDCGVYMNRQSCYCPWKSRKPGPNVNINECLLNEYYYTVSFTPNISFDSDHCKRYE        360
tr|A0A8E3CM32|A0A8E3CM32_RHIMP           HEEFMDCGVYMNRQSCYCPWKSRKPGPNVNINECLLNEYYYTVSFTPNISFDSDHCKRYE        360
tr|A0A8E3CLI5|A0A8E3CLI5_RHIMP           HEEFMDCGVYMNRQSCYCPWKSRKPGPNVNINECLLNEYYYTVSFTPNISFDSDHCDWYE        360
tr|A0A8E3CLI1|A0A8E3CLI1_RHIMP           HEEFMDCGVYMNRQSCYCPWKSRKPGPNVNINECLLNEYYYTVSFTPNISLDSDHCDWYE        360
tr|A0A8E3CK47|A0A8E3CK47_RHIMP           HEEFMDCGVYMNRQSCYCPWKSRKPGPNVNINECLLNEYYYTVSFTPNISFDSDHCKRYE        360
```

```
tr|A0A8E3CK39|A0A8E3CK39_RHIMP      HEEFMDCGVYMNRQSCYCPWKSRKPGPNVNINECLLNEYYYTVSFTPNISFDSDHCKRYE      360
tr|A0A8E3CHR0|A0A8E3CHR0_RHIMP      HEEFMDCGVYMNRQSCYCPWKSRKPGPNVNINECLLNEYYYTVSFTPNISLDSDHCDWYE      360
tr|A0A8E3CHQ5|A0A8E3CHQ5_RHIMP      HEEFMDCGVYMNRQSCYCPWKSRKPGPNVNINECLLNEYYYTVSFTPNISFDSDHCKRYE      360
tr|A0A8E2ZDN2|A0A8E2ZDN2_RHIMP      HEEFMDCGVYMNRQSCYCPWKSRKPGPNVNINECLLNEYYYTVSFTPNISFDSDHCKRYE      360
tr|A0A8E2ZC62|A0A8E2ZC62_RHIMP      HEEFMDCGVYMNRQSCYCPWKSRKPGPNVNINECLLNEYYYTVSFTPNISFDSDHCKRYE      360
tr|A0A8E2ZC54|A0A8E2ZC54_RHIMP      HEEFMDCGVYMNRQSCYCPWKSRKPGPNVNINECLLNEYYYTVSFTPNISLDSDHCKRYE      360
tr|A0A8E2ZC45|A0A8E2ZC45_RHIMP      HEEFMDCGVYMNRQSCYCPWKSRKPGPNVNINECLLNEYYYTVSFTPNISFDSDHCKRYE      360
tr|A0A8E2ZAC1|A0A8E2ZAC1_RHIMP      HEEFMDCGVYMNRQSCYCPWKSRKPGPNVNINECLLNEYYYTVSFTPNISLDSDHCDWYE      360
tr|A0A8E2ZAB3|A0A8E2ZAB3_RHIMP      HEEFMDCGVYMNRQSCYCPWKSRKPGPNVNINECLLNEYYYTVSFTPNISLDSDHCDWYE      360
tr|A0A8E2Z9N3|A0A8E2Z9N3_RHIMP      HEEFMDCGVYMNRQSCYCPWKSRKPGPNVNINECLLNEYYYTVSFTPNISLDSDHCDWYE      360
tr|A0A8E2Z9M6|A0A8E2Z9M6_RHIMP      HEEFMDCGVYMNRQSCYCPWKSRKPGPNVNINECLLNEYYYTVSFTPNISFDSDHCKRYE      360
tr|A0A8E2Z9L6|A0A8E2Z9L6_RHIMP      HEEFMDCGVYMNRQSCYCPWKSRKPGPNVNINECLLNEYYYTVSFTPNISFDSDHCKRYE      360
tr|A0A8E2Z8G3|A0A8E2Z8G3_RHIMP      HEEFMDCGVYMNRQSCYCPWKSRKPGPNVNINECLLNEYYYTVSFTPNISFDSDHCKRYE      360
tr|A0A8E2Z7X1|A0A8E2Z7X1_RHIMP      HEEFMDCGVYMNRQSCYCPWKSRKPGPNVNINECLLNEYYYTVSFTPNISFDSDHCKRYE      360
tr|A0A8E2Z7W9|A0A8E2Z7W9_RHIMP      HEEFMDCGVYMNRQSCYCPWKSRKPGPNVNINECLLNEYYYTVSFTPNISFDSDHCKRYE      360
tr|A0A8E2Z7W5|A0A8E2Z7W5_RHIMP      HEEFMDCGVYMNRQSCYCPWKSRKPGPNVNINECLLNEYYYTVSFTPNISFDSDHCKRYE      360
tr|A0A8E2Z7V4|A0A8E2Z7V4_RHIMP      HEEFMDCGVYMNRQSCYCPWKSRKPGPNVNINECLLNEYYYTVSFTPNISFDSDHCKRYE      360
tr|A0A8E2Z7U7|A0A8E2Z7U7_RHIMP      HEEFMDCGVYMNRQSCYCPWKSRKPGPNVNINECLLNEYYYTVSFTPNISFDSDHCKRYE      360
tr|A0A8E2Z7U5|A0A8E2Z7U5_RHIMP      HEEFMDCGVYMNRQSCYCPWKSRKPGPNVNINECLLNEYYYTVSFTPNISFDSDHCKRYE      360
tr|A0A8E2Z7U4|A0A8E2Z7U4_RHIMP      HEEFMDCGVYMNRQSCYCPWKSRKPGPNVNINECLLNEYYYTVSFTPNISFDSDHCKRYE      360
tr|A0A8E2Z7T7|A0A8E2Z7T7_RHIMP      HEEFMDCGVYMNRQSCYCPWKSRKPGPNVNINECLLNEYYYTVSFTPNISFDSDHCKRYE      360
tr|A0A8E2Z7S4|A0A8E2Z7S4_RHIMP      HEEFMDCGVYMNRQSCYCPWKSRKPGPNVNINECLLNEYYYTVSFTPNISFDSDHCKRYE      360
tr|A0A6M2CI36|A0A6M2CI36_RHIMP      HEEFMDCGVYMNRQSCYCPWKSRKPGPNVNINECLLNEYYYTVSFTPNISFDSDHCKRYE      360
tr|Q5XKV2|Q5XKV2_RHIMP              HEEFMDCGVYMNRQSCYCPWKSRKPGPNVNINECLLNEYYYTVSFT--------------      264
                                    ********.****: ** ******** :*:*:: ********:***

tr|Q5ER80|Q5ER80_RHIMP              ------------------------------------------------------------      264
tr|A0A8E2Z927|A0A8E2Z927_RHIMP      DRVLEAIRTSIGKEVFKVEILNCTQDIKARLIAEKPLSKYVLRKLQACEHPIGEWCMMYP      420
tr|A0A8E2Z7V6|A0A8E2Z7V6_RHIMP      DRVLEAIRTSIGKEVFKVEILNCTQDIKARLIAEKPLSKYVLRKLQACEHPIGEWCMMYP      420
tr|A0A8E2Z7X7|A0A8E2Z7X7_RHIMP      DRVLEAIRTSIGKEVFKVEILNCTQDIKARLIAEKPLSKYVLRKLQACEHPIGEWCMMYP      420
tr|A0A8E3CL24|A0A8E3CL24_RHIMP      DRVLEAIRTSIGKEVFKVEILNCTQDIKARLIAEKPLSKYVLRKLQACEHPIGEWCMMYP      420
tr|A0A8E2Z8E9|A0A8E2Z8E9_RHIMP      DRVLEAIRTSIGKEVFKVEILNCTQDIKARLIAEKPLSKYVLRKLQACEHPIGEWCMMYP      420
tr|A0A8E2Z916|A0A8E2Z916_RHIMP      DRVLEAIRTSIGKEVFKVEILNCTQDIKARLIAEKPLSKYVLRKLQACEHPIGEWCMMYP      420
tr|A0A8E2Z7Z1|A0A8E2Z7Z1_RHIMP      DRVLEAIRTSIGKEVFKVEILNCTQDIKARLIAEKPLSKYVLRKLQACEHPIGEWCMMYP      420
tr|A0A8E3CLH7|A0A8E3CLH7_RHIMP      DRVLEAIRTSIGKEVFKVEILNCTQDIKARLIAEKPLSKYVLRKLQACEHPIGEWCMMYP      420
tr|A0A8E3CK42|A0A8E3CK42_RHIMP      DRVLEAIRTSIGKEVFKVEILNCTQDIKARLIAEKPLSKYVLRKLQACEHPIGEWCMMYP      420
tr|C4NAN0|C4NAN0_RHIMP              DRVLEAIRTSIGKEVFKVEILNCTQDIKARLIAEKPLSKYVLRKLQACEHPIGEWCMMYP      401
tr|E5KCH6|E5KCH6_RHIMP              DRVLEAIRTSIGKEVFKVEILNCTQDIKARLIAEKPLSKYVLRKLQACEHPIGEWCMMYP      420
tr|Q5XKU6|Q5XKU6_RHIMP              ------------------------------------------------------------      264
tr|Q5ER81|Q5ER81_RHIMP              ------------------------------------------------------------      264
tr|Q5ER93|Q5ER93_RHIMP              ------------------------------------------------------------      264
tr|Q5ER89|Q5ER89_RHIMP              ------------------------------------------------------------      264
tr|A0A8E2Z7V2|A0A8E2Z7V2_RHIMP      DRVLEAIRTSIGKEVFKVEILNCTQDIKARLIAEKPLSKYVLRKLQACEHPIGEWCMMYP      420
tr|Q5ER77|Q5ER77_RHIMP              ------------------------------------------------------------      264
tr|Q5ER78|Q5ER78_RHIMP              ------------------------------------------------------------      264
tr|Q5ER69|Q5ER69_RHIMP              ------------------------------------------------------------      264
tr|Q5XKU9|Q5XKU9_RHIMP              ------------------------------------------------------------      264
tr|Q9Y0V1|Q9Y0V1_RHIMP              DRVLGAIRTSIGKEVFKVEILNCTQDIKARLIAEKPLSKYVLRKLQACEHPIGEWCMMYP      420
tr|Q5XKU7|Q5XKU7_RHIMP              ------------------------------------------------------------      264
tr|Q5I6P2|Q5I6P2_RHIMP              ------------------------------------------------------------      264
tr|Q5ER92|Q5ER92_RHIMP              ------------------------------------------------------------      264
tr|Q5ER88|Q5ER88_RHIMP              ------------------------------------------------------------      264
tr|Q5ER82|Q5ER82_RHIMP              ------------------------------------------------------------      264
tr|A0A8E3CK52|A0A8E3CK52_RHIMP      DRVLEAIRTSIGKEVFKVEILNCTQDIKARLIAEKPLSNHVLRKLQTCEHPIGEWCMMYP      420
tr|A0A8E2ZC71|A0A8E2ZC71_RHIMP      DRVLEAIRTSIGKEVFKVEILNCTQDIKARLIAEKPLSKHVLRKLQACEHPIGEWCMMYP      420
tr|A0A8E2Z937|A0A8E2Z937_RHIMP      DRVLEAIRTSIGKEVFKVEILNCTQDIKARLIAEKPLSKYVLRKLQACEHPIGEWCMMYP      420
tr|A0A8E2Z8F5|A0A8E2Z8F5_RHIMP      DRVLEAIRTSIGKEVFKVEILNCTQDIKARLIAEKPLSKYVLRKLQACEHPIGEWCMMYP      420
tr|A0A8E3CHP7|A0A8E3CHP7_RHIMP      DRVLEAIRTSIGKEVFKVEILNCTQDIKARLIAEKPLSKYVLRKLQACEHPIGEWCMMYP      420
tr|E5KCI4|E5KCI4_RHIMP              DRVLEAIRTSIGKEVFKVEILNCTQDIKARLIAEKPLSKYVLRKLQACEHPIGEWCMMYP      420
tr|A0A8E3CM27|A0A8E3CM27_RHIMP      DRVLEAIRTSIGKEVFKVEILNCTQDIKARLIAEKPLSKYVLRKLQACEHPIGEWCMMYP      420
tr|Q5XKU8|Q5XKU8_RHIMP              ------------------------------------------------------------      264
tr|Q5I6P1|Q5I6P1_RHIMP              ------------------------------------------------------------      264
tr|Q5ER85|Q5ER85_RHIMP              ------------------------------------------------------------      264
tr|Q5ER75|Q5ER75_RHIMP              ------------------------------------------------------------      264
tr|Q5ER64|Q5ER64_RHIMP              ------------------------------------------------------------      264
tr|E5KCI2|E5KCI2_RHIMP              DRVLEAIRTSIGKEVFKVEILNCTQDIKARLIAEKPLSKYVLRKLQACEHPIGEWCMMYP      420
tr|A0A8E2ZDL2|A0A8E2ZDL2_RHIMP      DRVLEAIRTSIGKEVFKVEILNCTQDIKARLIAEKPLSKYVLRKLQACEHPIGEWCMMYP      420
tr|A0A8E2ZDK4|A0A8E2ZDK4_RHIMP      DRVLEAIRTSIGKEVFKVEILNCTQDIKARLIAEKPLSKYVLRKLQACEHPIGEWCMMYP      420
tr|E5KCI6|E5KCI6_RHIMP              DRVLEAIRTSIGKEVFKVEILNCTQDIKARLIAEKPLSKYVLRKLQACEHPIGEWCMMYP      420
tr|E5KCI5|E5KCI5_RHIMP              DRVLEAIRTSIGKEVFKVEILNCTQDIKARLIAEKPLSKYVLKKLQACEHPIGEWCMMYP      420
tr|E5KCI1|E5KCI1_RHIMP              DRVLEAIRTSIGKEVFKVEILNCTQDIKARLIAEKPLSKYVLRKLQACEHPIGEWCMMYP      420
tr|E5KCI0|E5KCI0_RHIMP              DRVLEAIRTSIGKEVFKVEILNCTQDIKARLIAEKPLSKYVLRKLQACEHPIGEWCMMYP      420
tr|E5KCH9|E5KCH9_RHIMP              DRVLEAIRTSIGKEVFKVEILNCTQDIKARLIAEKPLSKYVLRKLQACEHPIGEWCMMYP      420
tr|E5KCH3|E5KCH3_RHIMP              DRVLEAIRTSIGKEVFKVEILNCTQDIKARLIAEKPLSKYVLRKLQACEHPIGEWCMMYP      420
tr|B6CNU3|B6CNU3_RHIMP              DRVLEAIRTSIGKEVFKVEILNCTQDIKARLIAEKPLSKYVLRKLQACEHPIGEWCMMYP      400
tr|A0A8E3CM42|A0A8E3CM42_RHIMP      DRVLEAIRTSIGKEVFKVEILNCTQDIKARLIAEKPLSNHVLRKLQTCEHPIGEWCMMYP      420
tr|A0A8E3CM37|A0A8E3CM37_RHIMP      DRVLEAIRTSIGKEVFKVEILNCTQDIKARLIAEKPLSKYVLRKLQACEHPIGEWCMMYP      420
tr|A0A8E3CM32|A0A8E3CM32_RHIMP      DRVLEAIRTSIGKEVFKVEILNCTQDIKARLIAEKPLSKYVLRKLQACEHPIGEWCMMYP      420
tr|A0A8E3CLI5|A0A8E3CLI5_RHIMP      DRVLEAIRTSIGKEVFKVEILNCTQDIKARLIAEKPLSNHVLRKLQTCEHPIGEWCMMYP      420
tr|A0A8E3CLI1|A0A8E3CLI1_RHIMP      DRVLEAIRTSIGKEVFKVEILNCTQDIKARLIAEKPLSNHVLRKLQTCEHPIGEWCMMYP      420
tr|A0A8E3CK47|A0A8E3CK47_RHIMP      DRVLEAIRTSIGKEVFKVEILNCTQDIKARLIAEKPLSKYVLRKLQTCEHPIGEWCMMYP      420
tr|A0A8E3CK39|A0A8E3CK39_RHIMP      DRVLEAIRTSIGKEVFKVEILNCTQDIKARLIAEKPLSKYVLRKLQACEHPIGEWCMMYP      420
tr|A0A8E3CHR0|A0A8E3CHR0_RHIMP      DRVLEAIRTSIGKEVFKVEILNCTQDIKARLIAEKPLSNHVLRKLQTCEHPIGEWCMMYP      420
tr|A0A8E3CHQ5|A0A8E3CHQ5_RHIMP      DRVLEAIRTSIGKEVFKVEILNCTQDIKARLIAEKPLSKYVLRKLQACEHPIGEWCMMYP      420
tr|A0A8E2ZDN2|A0A8E2ZDN2_RHIMP      DRVLEAIRTSIGKEVFKVEILNCTQDIKARLIAEKPLSNHVLRKLQTCEHPIGEWCMMYP      420
tr|A0A8E2ZC62|A0A8E2ZC62_RHIMP      DRVLEAIRTSIGKEVFKVEILNCTQDIKARLIAEKPLSKHVLRKLQTCEHPIGEWCMMYP      420
tr|A0A8E2ZC54|A0A8E2ZC54_RHIMP      DRVLEAIRTSIGKEVFKVEILNCTQDIKARLIAEKPLSKYVLRKLQACEHPIGEWCMMYP      420
tr|A0A8E2ZC45|A0A8E2ZC45_RHIMP      DRVLEAIRTSIGKEVFKVEILNCTQDIKARLIAEKPLSKYVLRKLQACEHPIGEWCMMYP      420
tr|A0A8E2ZAC1|A0A8E2ZAC1_RHIMP      DRVLEAIRTSIGKEVFKVEILNCTQDIKARLIAEKPLSNHVLRKLQTCEHPIGEWCMMYP      420
tr|A0A8E2ZAB3|A0A8E2ZAB3_RHIMP      DRVLEAIRTSIGKEVFKVEILNCTQDIKARLIAEKPLSNHVLRKLQACEHPIGEWCMMYP      420
tr|A0A8E2Z9N3|A0A8E2Z9N3_RHIMP      DRVLEAIRTSIGKEVFKVEILNCTQDIKARLIAEKPLSNHVLRKLQACEHPIGEWCMMYP      420
tr|A0A8E2Z9M6|A0A8E2Z9M6_RHIMP      DRVLEAIRTSIGKEVFKVEILNCTQDIKARLIAEKPLSNHVLRKLQTCEHPIGEWCMMYP      420
tr|A0A8E2Z9L6|A0A8E2Z9L6_RHIMP      DRVLEAIRTSIGKEVFKVEILNCTQDIKARLIAEKPLSKYVLRKLQACEHPIGEWCMMYP      420
tr|A0A8E2Z8G3|A0A8E2Z8G3_RHIMP      DRVLEAIRTSIGKEVFKVEILNCTQDIKARLIAEKPLSKYVLRKLQACEHPIGEWCMMYP      420
```

```
tr|A0A8E2Z7X1|A0A8E2Z7X1_RHIMP      DRVLEAIRTSIGKEVFKVEILNCTQDIKARLIAEKPLSNYVLRKLQTCEHPIGEWCMMYP          420
tr|A0A8E2Z7W9|A0A8E2Z7W9_RHIMP      DRVLEAIRTSIGKEVFKVEILNCTQDIKARLIAEKPLSKYVLRKLQACEHPIGEWCMMYP          420
tr|A0A8E2Z7W5|A0A8E2Z7W5_RHIMP      DRVLEAIRTSIGKEVFKVEILNCTQDIKARLIAEKPLSNHVLRKLQTCEHPIGEWCMMYP          420
tr|A0A8E2Z7V4|A0A8E2Z7V4_RHIMP      DRVLEAIRTSIGKEVFKVEILNCTQDIKARLIAEKPLSNHVLRKLQACEHPIGEWCMMYP          420
tr|A0A8E2Z7U7|A0A8E2Z7U7_RHIMP      DRVLEAIRTSIGKEVFKVEILNCTQDIKARLIAEKPLSKYVLRKLQACEHPIGEWCMMYP          420
tr|A0A8E2Z7U5|A0A8E2Z7U5_RHIMP      DRVLEAIRTSIGKEVFKVEILNCTQDIKARLIAEKPLSKYVLRKLQACEHPIGEWCMMYP          420
tr|A0A8E2Z7U4|A0A8E2Z7U4_RHIMP      DRVLEAIRTSIGKEVFKVEILNCTQDIKARLIAEKPLSKYVLRKLQACEHPIGEWCMMYP          420
tr|A0A8E2Z7T7|A0A8E2Z7T7_RHIMP      DRVLEAIRTSIGKEVFKVEILNCTQDIKARLIAEKPLSKYVLRKLQACEHPIGEWCMMYP          420
tr|A0A8E2Z7S4|A0A8E2Z7S4_RHIMP      DRVLEAIRTSIGKEVFKVEILNCTQDIKARLIAEKPLSKYVLRKLQACEHPIGEWCMMYP          420
tr|A0A6M2CI36|A0A6M2CI36_RHIMP      DRVLEAIRTSIGKEVFKVEILNCTQDIKARLIAEKPLSKYVLRKLQACEHPIGEWCMMYP          420
tr|Q5XKV2|Q5XKV2_RHIMP              ------------------------------------------------------------          264


tr|Q5ER80|Q5ER80_RHIMP              ------------------------------------------------------------          264
tr|A0A8E2Z927|A0A8E2Z927_RHIMP      KLLIKKNSATEIEEENLCDSLLKNQEAAYKGQNKCVKVDNLFWFQCADGYTTTYEMTRGR          480
tr|A0A8E2Z7V6|A0A8E2Z7V6_RHIMP      KLLIKKNSATEIEEENLCDSLLKNQEAAYKGQNKCVKVDNLFWFQCADGYTTTYEMTRGR          480
tr|A0A8E2Z7X7|A0A8E2Z7X7_RHIMP      KLLIKKNSATEIEEENLCDSLLKNQEAAYKGQNKCVKVDNLFWFQCADGYTTTYEMTRGR          480
tr|A0A8E3CL24|A0A8E3CL24_RHIMP      KLLIKKNSATEIEEENLCDSLLKNQEAAYKGQNKCVKVDNLFWFQCADGYTTTYEMTRGR          480
tr|A0A8E2Z8E9|A0A8E2Z8E9_RHIMP      KLLIKKNSATEIEEENLCDSLLKNQEAAYKGQNKCVKVDNLFWFQCADGYTTTYEMTRGR          480
tr|A0A8E2Z916|A0A8E2Z916_RHIMP      KLLIKKNSATEIEEENLCDSLLKNQEAAYKGQNKCVKVDNLFWFQCADGYTTTYEMTRGR          480
tr|A0A8E2Z7Z1|A0A8E2Z7Z1_RHIMP      KLLIKKNSATEIEEENLCDSLLKNQEAAYKGQNKCVKVDNLFWFQCADGYTTTYEMTRGR          480
tr|A0A8E3CLH7|A0A8E3CLH7_RHIMP      KLLIKKNSATEIEEENLCDSLLKNQEAAYKGQNKCVKVDNLFWFQCADGYTTTYEMTRGR          480
tr|A0A8E3CK42|A0A8E3CK42_RHIMP      KLLIKKNSATEIEEENLCDSLLKNQEAAYKGQNKCVKVDNLFWFQCADGYTTTYEMTRGR          480
tr|C4NAN0|C4NAN0_RHIMP              KLLIKKNSATEIEEENLCDSLLKNQEAAYKGQNKCVKVDNLFWFQCADGYTTTYEMTRGR          461
tr|E5KCH6|E5KCH6_RHIMP              KLLIKKNSATEIEEENLCDSLLKNQEAAYKGQNKCVKVDNLFWFQCADGYTTTYEMTRGR          480
tr|Q5XKU6|Q5XKU6_RHIMP              ------------------------------------------------------------          264
tr|Q5ER81|Q5ER81_RHIMP              ------------------------------------------------------------          264
tr|Q5ER93|Q5ER93_RHIMP              ------------------------------------------------------------          264
tr|Q5ER89|Q5ER89_RHIMP              ------------------------------------------------------------          264
tr|A0A8E2Z7V2|A0A8E2Z7V2_RHIMP      KLLIKKNSATEIEEENLCDSLLKNQEAAYKGQNKCVKVDNLFWFQCADGYTTTYEMTRGR          480
tr|Q5ER77|Q5ER77_RHIMP              ------------------------------------------------------------          264
tr|Q5ER78|Q5ER78_RHIMP              ------------------------------------------------------------          264
tr|Q5ER69|Q5ER69_RHIMP              ------------------------------------------------------------          264
tr|Q5XKU9|Q5XKU9_RHIMP              ------------------------------------------------------------          264
tr|Q9Y0V1|Q9Y0V1_RHIMP              KLLIKKNSATEIEEENLCDSLLKNQEAAYKGQNKCVKVDNLFWFQCADGYTTTYEMTRGR          480
tr|Q5XKU7|Q5XKU7_RHIMP              ------------------------------------------------------------          264
tr|Q5I6P2|Q5I6P2_RHIMP              ------------------------------------------------------------          264
tr|Q5ER92|Q5ER92_RHIMP              ------------------------------------------------------------          264
tr|Q5ER88|Q5ER88_RHIMP              ------------------------------------------------------------          264
tr|Q5ER82|Q5ER82_RHIMP              ------------------------------------------------------------          264
tr|A0A8E3CK52|A0A8E3CK52_RHIMP      KLLIKKNSATEIEEENLCDSLLKNQEAAYKGQNKCVKVDNLFWFQCADGYTTTYEMTRGR          480
tr|A0A8E2ZC71|A0A8E2ZC71_RHIMP      KLLIKKNSATEIEEENLCDSLLKNQEAAYKGQNKCVKVDNLFWFQCADGYTTTYEMTRGR          480
tr|A0A8E2Z937|A0A8E2Z937_RHIMP      KLLIKKNSATEIEEENLCDSLLKNQEAAYKGQNKCVKVDNLFWFQCADGYTTTYEMTRGR          480
tr|A0A8E2Z8F5|A0A8E2Z8F5_RHIMP      KLLIKKNSATEIEEENLCDSLLKNQEAAYKGQNKCVKVDNLFWFQCADGYTTTYEMTRGR          480
tr|A0A8E3CHP7|A0A8E3CHP7_RHIMP      KLLIKKNSATEIEEENLCDSLLKNQEAAYKGQNKCVKVDNLFWFQCADGYTTTYEMTRGR          480
tr|E5KCI4|E5KCI4_RHIMP              KLLIKKNSATEIEEENLCDSLLKNQEAAYKGQNKCVKVDNLFWFQCADGYTTTYEMTRGR          480
tr|A0A8E3CM27|A0A8E3CM27_RHIMP      KLLIKKNSATEIEEENLCDSLLKNQEAAYKGQNKCVKVDNLFWFQCADGYTTTYEMTRGR          480
tr|Q5XKU8|Q5XKU8_RHIMP              ------------------------------------------------------------          264
tr|Q5I6P1|Q5I6P1_RHIMP              ------------------------------------------------------------          264
tr|Q5ER85|Q5ER85_RHIMP              ------------------------------------------------------------          264
tr|Q5ER75|Q5ER75_RHIMP              ------------------------------------------------------------          264
tr|Q5ER64|Q5ER64_RHIMP              ------------------------------------------------------------          264
tr|E5KCI2|E5KCI2_RHIMP              KLLIKKNSATEIEEENLCDSLLKNQEAAYKGQNKCVKVDNLFWFQCADGYTTTYEMTRGR          480
tr|A0A8E2ZDL2|A0A8E2ZDL2_RHIMP      KLLIKKNSATEIEEENLCDSLLKNQEAAYKGQNKCVKVDNLFWFQCADGYTTTYEMTRGR          480
tr|A0A8E2ZDK4|A0A8E2ZDK4_RHIMP      KLLIKKNSATEIEEENLCDSLLKNQEAAYKGQNKCVKVDNLFWFQCADGYTTTYEMTRGR          480
tr|E5KCI6|E5KCI6_RHIMP              KLLVKKNSATEIEEENLCDSLLKNQEAAYKGQNKCVKVDNLFWFQCADGYTTTYEMTRGR          480
tr|E5KCI5|E5KCI5_RHIMP              KLLIKKNSATEIEEENLCDSLLKNQEAAYKGQNKCVKVDNLFWFQCADGYTTTYEMTRGR          480
tr|E5KCI1|E5KCI1_RHIMP              KLLIKKNSATEIEEENLCDSLLKNQEAAYKGQNKCVKVDNLFWFQCADGYTTTYEMTRGR          480
tr|E5KCI0|E5KCI0_RHIMP              KLLIKKNSATEIEEENLCDSLLKNQEAAYKGQNKCVKVDNLFWFQCADGYTTTYEMTRGR          480
tr|E5KCH9|E5KCH9_RHIMP              KLLIKKNSATEIEEENLCDSLLKNQEAAYKGQNKCVKVDNLFWFQCADGYTTTYEMTRGR          480
tr|E5KCH3|E5KCH3_RHIMP              KLLIKKNSATEIEEENLCDSLLKNQEAAYKGQNKCVKVDNLFWFQCADGYTTTYEMTRGR          480
tr|B6CNU3|B6CNU3_RHIMP              KLLIKKNSATEIEEENLCDSLLKNQEAAYKGQNKCVKVDNLFWFQCADGYTTTYEMTRGR          460
tr|A0A8E3CM42|A0A8E3CM42_RHIMP      KLLIKKNSATEIEEENLCDSLLKNQEAAYKGQNKCVKVDNLFWFQCADGYTTTYEMTRGR          480
tr|A0A8E3CM37|A0A8E3CM37_RHIMP      KLLIKKNSATEIEEENLCDSLLKNQEAAYKGQNKCVKVDNLFWFQCADGYTTTYEMTRGR          480
tr|A0A8E3CM32|A0A8E3CM32_RHIMP      KLLIKKNSATEIEEENLCDSLLKNQEAAYKGQNKCVKVDNLFWFQCADGYTTTYEMTRGR          480
tr|A0A8E3CLI5|A0A8E3CLI5_RHIMP      KLLIKKNSATEIEEENLCDSLLKNQEAAYKGQNKCVKVDNLFWFQCADGYTTTYEMTRGR          480
tr|A0A8E3CLI1|A0A8E3CLI1_RHIMP      KLLIKKNSATEIEEENLCDSLLKNQEAAYKGQNKCVKVDNLFWFQCADGYTTTYEMTRGR          480
tr|A0A8E3CK47|A0A8E3CK47_RHIMP      KLLIKKNSATEIEEENLCDSLLKNQEAAYKGQNKCVKVDNLFWFQCADGYTTTYEMTRGR          480
tr|A0A8E3CK39|A0A8E3CK39_RHIMP      KLLIKKNSATEIEEENLCDSLLKNQEAAYKGQNKCVKVDNLFWFQCADGYTTTYEMTRGR          480
tr|A0A8E3CHR0|A0A8E3CHR0_RHIMP      KLLIKKNSATEIEEENLCDSLLKNQEAAYKGQNKCVKVDNLFWFQCADGYTTTYEMTRGR          480
tr|A0A8E3CHQ5|A0A8E3CHQ5_RHIMP      KLLIKKNSATEIEEENLCDSLLKNQEAAYKGQNKCVKVDNLFWFQCADGYTTTYEMTRGR          480
tr|A0A8E2ZDN2|A0A8E2ZDN2_RHIMP      KLLIKKNSATEIEEENLCDSLLKNQEAAYKGQNKCVKVDNLFWFQCADGYTTTYEMTRGR          480
tr|A0A8E2ZC62|A0A8E2ZC62_RHIMP      KLLIKKNSATEIEEENLCDSLLKNQEAAYKGQNKCVKVDNLFWFQCADGYTTTYEMTRGR          480
tr|A0A8E2ZC54|A0A8E2ZC54_RHIMP      KLLIKKNSATEIEEENLCDSLLKNQEAAYKGQNKCVKVDNLFWFQCADGYTTTYEMTRGR          480
tr|A0A8E2ZC45|A0A8E2ZC45_RHIMP      KLLIKKNSATEIEEENLCDSLLKNQEAAYKGQNKCVKVDNLFWFQCADGYTTTYEMTRGR          480
tr|A0A8E2ZAC1|A0A8E2ZAC1_RHIMP      KLLIKKNSATEIEEENLCDSLLKNQEAAYKGQNKCVKVDNLFWFQCADGYTTTYEMTRGR          480
tr|A0A8E2ZAB3|A0A8E2ZAB3_RHIMP      KLLIKKNSATEIEEENLCDSLLKNQEAAYKGQNKCVKVDNLFWFQCADGYTTTYEMTRGR          480
tr|A0A8E2Z9N3|A0A8E2Z9N3_RHIMP      KLLIKKNSATEIEEENLCDSLLKNQEAAYKGQNKCVKVDNLFWFQCADGYTTTYEMTRGR          480
tr|A0A8E2Z9M6|A0A8E2Z9M6_RHIMP      KLLIKKNSATEIEEENLCDSLLKNQEAAYKGQNKCVKVDNLFWFQCADGYTTTYEMTRGR          480
tr|A0A8E2Z9L6|A0A8E2Z9L6_RHIMP      KLLIKKNSATEIEEENLCDSLLKNQEAAYKGQNKCVKVDNLFWFQCADGYTTTYEMTRGR          480
tr|A0A8E2Z8G3|A0A8E2Z8G3_RHIMP      KLLIKKNSATEIEEENLCDSLLKNQEAAYKGQNQCVKVDNLFWFQCADGYTTTYEMTRGR          480
tr|A0A8E2Z7X1|A0A8E2Z7X1_RHIMP      KLLIKKNSATEIEEENLCDSLLKNQEAAYKGQNKCVKVDNLFWFQCADGYTTTYEMTRGR          480
tr|A0A8E2Z7W9|A0A8E2Z7W9_RHIMP      KLLIKKNSATEIEEENLCDSLLKNQEAAYKGQNKCVKVDNLFWFQCADGYTTTYEMTRGR          480
tr|A0A8E2Z7W5|A0A8E2Z7W5_RHIMP      KLLIKKNSATEIEEENLCDSLLKNQEAAYKGQNKCVKVDNLFWFQCADGYTTTYEMTRGR          480
tr|A0A8E2Z7V4|A0A8E2Z7V4_RHIMP      KLLIKKNSATEIEEENLCDSLLKNQEAAYKGQNKCVKVDNLFWFQCADGYTTTYEMTRGR          480
tr|A0A8E2Z7U7|A0A8E2Z7U7_RHIMP      KLLIKKNSATEIEEENLCDSLLKNQEAAYKGQNKCVKVDNLFWFQCADGYTTTYEMTRGR          480
tr|A0A8E2Z7U5|A0A8E2Z7U5_RHIMP      KLLIKKNSATEIEEENLCDSLLKNQEAAYKGQNKCVKVDNLFWFQCADGYTTTYEMTRGR          480
tr|A0A8E2Z7U4|A0A8E2Z7U4_RHIMP      KLLIKKNSATEIEEENLCDSLLKNQEAAYKGQNKCVKVDNLFWFQCADGYTTTYEMTRGR          480
tr|A0A8E2Z7T7|A0A8E2Z7T7_RHIMP      KLLIKKNSATEIEEENLCDSLLKNQEAAYKGQNKCVKVDNLFWFQCADGYTTTYEMTRGR          480
tr|A0A8E2Z7S4|A0A8E2Z7S4_RHIMP      KLLIKKNSATEIEEENLCDSLLKNQEAAYKGQNKCVKVDNLFWFQCADGYTTTYEMTRGR          480
tr|A0A6M2CI36|A0A6M2CI36_RHIMP      KLLIKKNSATEIEEENLCDSLLKNREAAYKGQNKCVKVDNLFWFQCADGYTTTYEMTRGR          480
tr|Q5XKV2|Q5XKV2_RHIMP              ------------------------------------------------------------          264
```

```
tr|Q5ER80|Q5ER80_RHIMP                ------------------------------------------------------------          264
tr|A0A8E2Z927|A0A8E2Z927_RHIMP        LRRSVCKAGVSCNENEQLECANKGQICVYENGKANCQCPPDTKPGEIGCIERTTCNPKEI          540
tr|A0A8E2Z7V6|A0A8E2Z7V6_RHIMP        LRRSVCKAGVSCNENEQLECANKGQICVYENGKANCQCPPDTKPGEIGCIERTTCNPKEI          540
tr|A0A8E2Z7X7|A0A8E2Z7X7_RHIMP        LRRSVCKAGVSCNENEQLECANKGQICVYENGKANCQCPPDTKPGEIGCIERTTCNPKEI          540
tr|A0A8E3CL24|A0A8E3CL24_RHIMP        LRRSVCKAGVSCNENEQLECANKGQICVYENGKANCQCPPDTKPGEIGCIERTTCNPKEI          540
tr|A0A8E2Z8E9|A0A8E2Z8E9_RHIMP        LRRSVCKAGVSCNENEQLECANKGQICVYENGKANCQCPPDTKPGEIGCIERTTCNPKEI          540
tr|A0A8E2Z916|A0A8E2Z916_RHIMP        LRRSVCKAGVSCNENEQLECANKGQICVYENGKANCQCPPDTKPGEIGCIERTTCNPKEI          540
tr|A0A8E2Z7Z1|A0A8E2Z7Z1_RHIMP        LRRSVCKAGVSCNENEQLECANKGQICVYENGKANCQCPPDTKPGEIGCIERTTCNPKEI          540
tr|A0A8E3CLH7|A0A8E3CLH7_RHIMP        LRRSVCKAGVSCNENEQLECANKGQICVYENGKANCQCPPDTKPGEIGCIERTTCNPKEI          540
tr|A0A8E3CK42|A0A8E3CK42_RHIMP        LRRSVCKAGVSCNENEQLECANKGQICVYENGKANCQCPPDTKPGEIGCIERTTCNPKEI          540
tr|C4NAN0|C4NAN0_RHIMP                LRRSVCKAGVSCNENEQLECANKGQICVYENGKANCQCPPDTKPGEIGCIERTTCNPKEI          521
tr|E5KCH6|E5KCH6_RHIMP                LRRSVCKAGVSCNENEQLECANKGQICVYENGKANCQCPPDTKPGEIGCIERTTCNPKEI          540
tr|Q5XKU6|Q5XKU6_RHIMP                ------------------------------------------------------------          264
tr|Q5ER81|Q5ER81_RHIMP                ------------------------------------------------------------          264
tr|Q5ER93|Q5ER93_RHIMP                ------------------------------------------------------------          264
tr|Q5ER89|Q5ER89_RHIMP                ------------------------------------------------------------          264
tr|A0A8E2Z7V2|A0A8E2Z7V2_RHIMP        LRRSVCKAGVSCNENEQLECANKGQICVYENGKANCQCPPDTKPGEIGCIERTTCNPKEI          540
tr|Q5ER77|Q5ER77_RHIMP                ------------------------------------------------------------          264
tr|Q5ER78|Q5ER78_RHIMP                ------------------------------------------------------------          264
tr|Q5ER69|Q5ER69_RHIMP                ------------------------------------------------------------          264
tr|Q5XKU9|Q5XKU9_RHIMP                ------------------------------------------------------------          264
tr|Q9Y0V1|Q9Y0V1_RHIMP                LRRSVCKAGVSCNENEQLECANKGQICVYENGKANCQCPPDTKPGEIGCIERTTCNPKEI          540
tr|Q5XKU7|Q5XKU7_RHIMP                ------------------------------------------------------------          264
tr|Q5I6P2|Q5I6P2_RHIMP                ------------------------------------------------------------          264
tr|Q5ER92|Q5ER92_RHIMP                ------------------------------------------------------------          264
tr|Q5ER88|Q5ER88_RHIMP                ------------------------------------------------------------          264
tr|Q5ER82|Q5ER82_RHIMP                ------------------------------------------------------------          264
tr|A0A8E3CK52|A0A8E3CK52_RHIMP        LRRSVCKAGVSCNENEQLECTNKGQICVYENGKANCQCPPDTKPGEIGCIERTTCNPKEI          540
tr|A0A8E2ZC71|A0A8E2ZC71_RHIMP        LRRSVCKAGVSCNENEQLECANKGQICVYENGKANCQCPPDTKPGEIGCIERTTCNPKEI          540
tr|A0A8E2Z937|A0A8E2Z937_RHIMP        LRRSVCKAGVSCNENEQLECANKGQICVYENGKANCQCPPDTKPGEIGCIERTTCNPKEI          540
tr|A0A8E2Z8F5|A0A8E2Z8F5_RHIMP        LRRSVCKAGVSCNENEQLECANKGQICVYENGKANCQCPPDTKPGEIGCIERTTCNPKEI          540
tr|A0A8E3CHP7|A0A8E3CHP7_RHIMP        LRRSVCKAGVSCNENEQLECANKGQICVYENGKANCQCPPDTKPGEIGCIERTTCNPKEI          540
tr|E5KCI4|E5KCI4_RHIMP                LRRSVCKAGVSCNENEQLECANKGQICVYENGKANCQCPPDTKPGEIGCIERTTCNPKEI          540
tr|A0A8E3CM27|A0A8E3CM27_RHIMP        LRRSVCKAGVSCNENEQLECANKGQICVYENGKANCQCPPDTKPGEIGCIERTTCNPKEI          540
tr|Q5XKU8|Q5XKU8_RHIMP                ------------------------------------------------------------          264
tr|Q5I6P1|Q5I6P1_RHIMP                ------------------------------------------------------------          264
tr|Q5ER85|Q5ER85_RHIMP                ------------------------------------------------------------          264
tr|Q5ER75|Q5ER75_RHIMP                ------------------------------------------------------------          264
tr|Q5ER64|Q5ER64_RHIMP                ------------------------------------------------------------          264
tr|E5KCI2|E5KCI2_RHIMP                LRRSVCKAGVSCNENEQLECANKGQICVYENGKANCQCPPDTKPGEIGCIERTTCNPKEI          540
tr|A0A8E2ZDL2|A0A8E2ZDL2_RHIMP        LRRSVCKAGVSCNENEQLECANKGQICVYENGKANCQCPPDTKPGEIGCIERTTCNPKEI          540
tr|A0A8E2ZDK4|A0A8E2ZDK4_RHIMP        LRRSVCKAGVSCNENEQLECANKGQICVYENGKANCQCPPDTKPGEIGCIERTTCNPKEI          540
tr|E5KCI6|E5KCI6_RHIMP                LRRSVCKAGVSCNENEQLECANKGQICVYENGKANCQCPPDTKPGEIGCIERTTCNPKEI          540
tr|E5KCI5|E5KCI5_RHIMP                LRRSVCKAGVSCNENEQLECANKGQICVYENGKANCQCPPDTKPGEIGCIERTTCNPKEI          540
tr|E5KCI1|E5KCI1_RHIMP                LRRSVCKAGVSCNENEQLECANKGQICVYENGKANCQCPPDTKPGEIGCIERTTCNPKEI          540
tr|E5KCI0|E5KCI0_RHIMP                LRRSVCKAGVSCNENEQLECANKGQICVYENGKANCQCPPDTKPGEIGCIERTTCNPKEI          540
tr|E5KCH9|E5KCH9_RHIMP                LRRSVCKAGVSCNENEQLECANKGQICVYENGKANCQCPPDTKPGEIGCIERTTCNPKEI          540
tr|E5KCH3|E5KCH3_RHIMP                LRRSVCKAGVSCNENEQLECANKGQICVYENGKANCQCPPDTKPGEIGCIERTTCNPKEI          540
tr|B6CNU3|B6CNU3_RHIMP                LRRSVCKAGVSCNENEQLECANKGQICVYENGKANCQCPPDTKPGEIGCIERTTCNPKEI          520
tr|A0A8E3CM42|A0A8E3CM42_RHIMP        LRRSVCKAGVSCNENEQLECTNKGQICVYENGKANCQCPPDTKPGEIGCIERTTCNPKEI          540
tr|A0A8E3CM37|A0A8E3CM37_RHIMP        LRRSVCKAGVSCNENEQLECANKGQICVYENGKANCQCPPDTKPGEIGCIERTTCNPKEI          540
tr|A0A8E3CM32|A0A8E3CM32_RHIMP        LRRSVCKAGVSCNENEQLECANKGQICVYENGKANCQCPPDTKPGEIGCIERTTCNPKEI          540
tr|A0A8E3CLI5|A0A8E3CLI5_RHIMP        LRRSVCKAGVSCNENEQLECTNKGQICVYENGKANCQCPPDTKPGEIGCIERTTCNPKEI          540
tr|A0A8E3CLI1|A0A8E3CLI1_RHIMP        LRRSVCKAGVSCNENEQLECTNKGQICVYENGKANCQCPPDTKPGEIGCIERTTCNPKEI          540
tr|A0A8E3CK47|A0A8E3CK47_RHIMP        LRRSVCKAGVSCNENEQLECTNKGQICVYENGKANCQCPPDTKPGEIGCIERTTCNPKEI          540
tr|A0A8E3CK39|A0A8E3CK39_RHIMP        LRRSVCKAGVSCNENEQLECANKGQICVYENGKANCQCPPDTKPGEIGCIERTTCNPKEI          540
tr|A0A8E3CHR0|A0A8E3CHR0_RHIMP        LRRSVCKAGVSCNENEQLECANKGQICVYENGKANCQCPPDTKPGEIGCIERTTCNPKEI          540
tr|A0A8E3CHQ5|A0A8E3CHQ5_RHIMP        LRRSVCKAGVSCNENEQLECTNKGQICVYENGKANCQCPPDTKPGEIGCIERTTCNPKEI          540
tr|A0A8E2ZDN2|A0A8E2ZDN2_RHIMP        LRRSVCKAGVSCNENEQLECTNKGQICVYENGKANCQCPPDTKPGEIGCIERTTCNPKEI          540
tr|A0A8E2ZC62|A0A8E2ZC62_RHIMP        LRRSVCKAGVSCNENEQLECTNKGQICVYENGKANCQCPPDTKPGEIGCIERTTCNPKEI          540
tr|A0A8E2ZC54|A0A8E2ZC54_RHIMP        LRRSVCKAGVSCNENEQLECANKGQICVYENGKANCQCPPDTKPGEIGCIERTTCNPKEI          540
tr|A0A8E2ZC45|A0A8E2ZC45_RHIMP        LRRSVCKAGVSCNENEQLECANKGQICVYENGKANCQCPPDTKPGEIGCIERTTCNPKEI          540
tr|A0A8E2ZAC1|A0A8E2ZAC1_RHIMP        LRRSVCKAGVSCNENEQLECTNKGQICVYENGKANCQCPPDTKPGEIGCIERTTCNPKEI          540
tr|A0A8E2ZAB3|A0A8E2ZAB3_RHIMP        LRRSVCKAGVSCNENEQLECTNKGQICVYENGKANCQCPPDTKPGEIGCIERTTCNPKEI          540
tr|A0A8E2Z9N3|A0A8E2Z9N3_RHIMP        LRRSVCKAGVSCNENEQLECTNKGQICVYENGKANCQCPPDTKPGEIGCIERTTCNPKEI          540
tr|A0A8E2Z9M6|A0A8E2Z9M6_RHIMP        LRRSVCKAGVSCNENEQLECTNKGQICVYENGKANCQCPPDTKPGEIGCIERTTCNPKEI          540
tr|A0A8E2Z9L6|A0A8E2Z9L6_RHIMP        LRRSVCKAGVSCNENEQLECANKGQICVYENGKANCQCPPDTKPGEIGCIERTTCNPKEI          540
tr|A0A8E2Z8G3|A0A8E2Z8G3_RHIMP        LRRSVCKAGVSCNENEQSECANKGQICVYENGKANCQCPPDTKPGEIGCIERTTCNPKEI          540
tr|A0A8E2Z7X1|A0A8E2Z7X1_RHIMP        LRRSVCKAGVSCNENEQLECTNKGQICVYENGKANCQCPPDTKPGEIGCIERTTCNPKEI          540
tr|A0A8E2Z7W9|A0A8E2Z7W9_RHIMP        LRRSVCKAGVSCNENEQSECANKGQICVYENGKANCQCPPDTKPGEIGCIERTTCNPKEI          540
tr|A0A8E2Z7W5|A0A8E2Z7W5_RHIMP        LRRSVCKAGVSCNENEQLECTNKGQICVYENGKANCQCPPDTKPGEIGCIERTTCNPKEI          540
tr|A0A8E2Z7V4|A0A8E2Z7V4_RHIMP        LRRSVCKAGVSCNENEQLECTNKGQICVYENGKANCQCPPDTKPGEIGCIERTTCNPKEI          540
tr|A0A8E2Z7U7|A0A8E2Z7U7_RHIMP        LRRSVCKAGVSCNENEQLECANKGQICVYENGKANCQCPPDTKPGEIGCIERTTCNPKEI          540
tr|A0A8E2Z7U5|A0A8E2Z7U5_RHIMP        LRRSVCKAGVSCNENEQLECANKGQICVYENGKANCQCPPDTKPGEIGCIERTTCNPKEI          540
tr|A0A8E2Z7U4|A0A8E2Z7U4_RHIMP        LRRSVCKAGVSCNENEQLECANKGQICVYENGKANCQCPPDTKPGEIGCIERTTCNPKEI          540
tr|A0A8E2Z7T7|A0A8E2Z7T7_RHIMP        LRRSVCKAGVSCNENEQLECANKGQICVYENGKANCQCPPDTKPGEIGCIERTTCNPKEI          540
tr|A0A8E2Z7S4|A0A8E2Z7S4_RHIMP        LRRSVCKAGVSCNENEQLECANKGQICVYENGKANCQCPPDTKPGEIGCIERTTCNPKEI          540
tr|A0A6M2CI36|A0A6M2CI36_RHIMP        LRRSVCKAGVSCNENEQLECANKGQICVYENGKANCQCPPDTKPGEIGCIERTTCNPKEI          540
tr|Q5XKV2|Q5XKV2_RHIMP                ------------------------------------------------------------          264


tr|Q5ER80|Q5ER80_RHIMP                ------------------------------------------------------------          264
tr|A0A8E2Z927|A0A8E2Z927_RHIMP        QECQDKKLECVYKNHKAECKCPDDHECSREPAKDSCSEEDNGKCQSSGQRCVMENGNAVC          600
tr|A0A8E2Z7V6|A0A8E2Z7V6_RHIMP        QECQDKKLECVYKNHKAECKCPDDHECSRQPAKDSCSEEDNGKCQSSGQRCVMENGKAVC          600
tr|A0A8E2Z7X7|A0A8E2Z7X7_RHIMP        QECQDKKLECVYKNHKAECKCPDDHECSRQPAKDSCSEEDNGKCQSSGQRCVMENGKAVC          600
tr|A0A8E3CL24|A0A8E3CL24_RHIMP        QECQDKKLECVYKNHKAECKCPDDHECSRQPAKDSCSEEDNGKCQSSGQRCVMENGKAVC          600
tr|A0A8E2Z8E9|A0A8E2Z8E9_RHIMP        QECQDKKLECVYKNHKAECKCPDDHECSRQPAKDSCSEEDNGKCQSSGQRCVMENGKAVC          600
tr|A0A8E2Z916|A0A8E2Z916_RHIMP        QECQDKKLECVYKNHKAECKCPDDHECSRQPAKDSCSEEDNGKCQSSGQRCVMENGKAVC          600
tr|A0A8E2Z7Z1|A0A8E2Z7Z1_RHIMP        QECQDKKLECVYKNHKAECKCPDDHECSRQPAKDSCSEEDNGKCQSSGQRCVMENGKAVC          600
tr|A0A8E3CLH7|A0A8E3CLH7_RHIMP        QECQDKKLECVYKNHKAECKCPDDHECSRQPAKDSCSEEDNGKCQSSGQRCVMENGKAVC          600
tr|A0A8E3CK42|A0A8E3CK42_RHIMP        QECQDKKLECVYKNHKAECKCPDDHECSRQPAKDSCSEEDNGKCQSSGQRCVMENGKAVC          600
tr|C4NAN0|C4NAN0_RHIMP                QECQDKKLECVYKNHKAECKCPDDHECSRQPAKDSCSEEDNGKCQSSGQRCVMENGKAVC          581
tr|E5KCH6|E5KCH6_RHIMP                QECQDKKLECVYKNHKAECKCPDDHECSRQPAKDSCSEEDNGKCQSSGQRCVMENGKAVC          600
tr|Q5XKU6|Q5XKU6_RHIMP                ------------------------------------------------------------          264
```

```
tr|Q5ER81|Q5ER81_RHIMP           ------------------------------------------------------------   264
tr|Q5ER93|Q5ER93_RHIMP           ------------------------------------------------------------   264
tr|Q5ER89|Q5ER89_RHIMP           ------------------------------------------------------------   264
tr|A0A8E2Z7V2|A0A8E2Z7V2_RHIMP   QECQDKKLECVYKNHKAECKCPDDHECSRQPAKDSCSEEDNGKCQSSGQRCVMENGKAVC   600
tr|Q5ER77|Q5ER77_RHIMP           ------------------------------------------------------------   264
tr|Q5ER78|Q5ER78_RHIMP           ------------------------------------------------------------   264
tr|Q5ER69|Q5ER69_RHIMP           ------------------------------------------------------------   264
tr|Q5XKU9|Q5XKU9_RHIMP           ------------------------------------------------------------   264
tr|Q9Y0V1|Q9Y0V1_RHIMP           QECQDKKLECVYKNHKAECKCPDDHECSR-------------------------------   569
tr|Q5XKU7|Q5XKU7_RHIMP           ------------------------------------------------------------   264
tr|Q5I6P2|Q5I6P2_RHIMP           ------------------------------------------------------------   264
tr|Q5ER92|Q5ER92_RHIMP           ------------------------------------------------------------   264
tr|Q5ER88|Q5ER88_RHIMP           ------------------------------------------------------------   264
tr|Q5ER82|Q5ER82_RHIMP           ------------------------------------------------------------   264
tr|A0A8E3CK52|A0A8E3CK52_RHIMP   QECQDKKLECVYKNHKAECKCPDDRECSREPAKDSCSEEDNGKCQSSGQRCVMENGNAVC   600
tr|A0A8E2ZC71|A0A8E2ZC71_RHIMP   QECQDKKLECVYKNHKAECKCPDDHECSREPAKDSCSEEDNGKCQSSGQRCVMENGNAVC   600
tr|A0A8E2Z937|A0A8E2Z937_RHIMP   QECQDKKLECVYKNHKAECKCPDDHECSRQPAKDSCSEEDNGKCQSSGQRCVMENGKAVC   600
tr|A0A8E2Z8F5|A0A8E2Z8F5_RHIMP   QECQDKKLECVYKNHKAECKCPDDHECSREPAKDSCSEEDNGKCQSSGQRCVMENGNAVC   600
tr|A0A8E3CHP7|A0A8E3CHP7_RHIMP   QECQDKKLECVYKNHKAECKCPDDHECSREPAKDSCSEEDNGKCQSSGQRCVMENGNAVC   600
tr|E5KCI4|E5KCI4_RHIMP           QECQDKKLECVYKNHKAECKCPDDHECSREPAKDSCSEEDNGKCQSSGQRCVMENGNAVC   600
tr|A0A8E3CM27|A0A8E3CM27_RHIMP   QECQDKKLECVYKNHKAECKCPDDHECSREPAKDSCSEEDNGKCQSSGQRCVMENGNAVC   600
tr|Q5XKU8|Q5XKU8_RHIMP           ------------------------------------------------------------   264
tr|Q5I6P1|Q5I6P1_RHIMP           ------------------------------------------------------------   264
tr|Q5ER85|Q5ER85_RHIMP           ------------------------------------------------------------   264
tr|Q5ER75|Q5ER75_RHIMP           ------------------------------------------------------------   264
tr|Q5ER64|Q5ER64_RHIMP           ------------------------------------------------------------   264
tr|E5KCI2|E5KCI2_RHIMP           QECQDKKLECVYKNHKAECKCPDDHECSREPAKDSCSEEDNGKCQSSGQRCVMENGNAVC   600
tr|A0A8E2ZDL2|A0A8E2ZDL2_RHIMP   QECQDKKLECVYKNHKAECKCPDDHECSREPAKDSCSEEDNGKCQSSGQRCVMENGKAVC   600
tr|A0A8E2ZDK4|A0A8E2ZDK4_RHIMP   QECQDKKLECVYKNHKAECKCPDDHECSREPAKDSCSEEDNGKCQSSGQRCVMENGNAVC   600
tr|E5KCI6|E5KCI6_RHIMP           QECQDKKLECVYKNHKAECKCPDDHECSREPAKDSCSEEDNGKCQSSGQRCVMENGNAVC   600
tr|E5KCI5|E5KCI5_RHIMP           QECQDKKLECVYKNHKAECKCPDDHECSRQPAKDSCSEEDNGKCQSSGQRCVMENGKAVC   600
tr|E5KCI1|E5KCI1_RHIMP           QECQDKKLECVYKNHKAECKCPDDHECSRQPAKDSCSEEDNGKCQSSGQRCVMENGNAVC   600
tr|E5KCI0|E5KCI0_RHIMP           QECQDKKLECVYKNHKAECKCPDDHECSREPAKDSCSEEDNGKCQSSGQRCVMENGNAVC   600
tr|E5KCH9|E5KCH9_RHIMP           QECQDKKLECVYKNHKAECKCPDDHECSREPAKDSCSEEDNGKCQSSGQRCVMENGNAVC   600
tr|E5KCH3|E5KCH3_RHIMP           QECQDKKLECVYKNHKAECKCPDDHECSREPAKDSCSEEDNGKCQSSGQRCVMENGNAVC   600
tr|B6CNU3|B6CNU3_RHIMP           QECQDKKLECVYKNHKAECKCPDDHECSREPAKDSCSEEDNGKCQSSGQRCVMENGNAVC   580
tr|A0A8E3CM42|A0A8E3CM42_RHIMP   QECQDKKLECVYKNHKAECKCPDDHECSREPAKDSCSEEDNGKCQSSGQRCVMENGNAVC   600
tr|A0A8E3CM37|A0A8E3CM37_RHIMP   QECQDKKLECVYKNHKAECKCPDDHECSREPAKDSCSEEDNGKCQSSGQRCVMENGKAVC   600
tr|A0A8E3CM32|A0A8E3CM32_RHIMP   QECQDKKLECVYKNHKAECKCPDDHECSRQPAKDSCSEEDNGKCQSSGQRCVMENGKAVC   600
tr|A0A8E3CLI5|A0A8E3CLI5_RHIMP   QECQDKKLECVYKNHKAECKCPDDRECSREPAKDSCSEEDNGKCQSSGQRCVMENGNAVC   600
tr|A0A8E3CLI1|A0A8E3CLI1_RHIMP   QECQDKKLECVYKNHKAECKCPDDHECSRQPAKDSCSEEDNGKCQSSGQRCVMENGNAVC   600
tr|A0A8E3CK47|A0A8E3CK47_RHIMP   QECQDKKLECVYKNHKAECKCPDDHECSREPAKDSCSEEDNGKCQSSGQRCVMENGNAVC   600
tr|A0A8E3CK39|A0A8E3CK39_RHIMP   QECQDKKLECVYKNHKAECKCPDDHECSREPAKDSCSEEDNGKCQSSGQRCVMENGNAVC   600
tr|A0A8E3CHR0|A0A8E3CHR0_RHIMP   QECQDKKLECVYKNHKAECKCPDDHECSREPAKDSCSEEDNGKCQSSGQRCVMENGNAVC   600
tr|A0A8E3CHQ5|A0A8E3CHQ5_RHIMP   QECQDKKLECVYKNHKAECKCPDDHECSREPAKDSCSEEDNGKCQSSGQRCVMENGKAVC   600
tr|A0A8E2ZDN2|A0A8E2ZDN2_RHIMP   QECQDKKLECVYKNHRAECKCPDDRECSREPAKDSCSEEDNGKCQSSGQRCVMENGNAVC   600
tr|A0A8E2ZC62|A0A8E2ZC62_RHIMP   QECQDKKLECVYKNHKAECKCPDDHECSRQPAKDSCSEEDNGKCQSSGQRCVMENGNAVC   600
tr|A0A8E2ZC54|A0A8E2ZC54_RHIMP   QECQDKKLECVYKNHKAECKCPDDHECSRQPAKDSCSEEDNGKCQSSGQRCVMENGKAVC   600
tr|A0A8E2ZC45|A0A8E2ZC45_RHIMP   QECQDKKLECVYKNHKAECKCPDDHECSREPAKDSCSEEDNGKCQSSGQRCVMENGNAVC   600
tr|A0A8E2ZAC1|A0A8E2ZAC1_RHIMP   QECQDKKLECVYKNHKAECKCPDDRECSREPAKDSCSEEDNGKCQSSGQRCVMENGNAVC   600
tr|A0A8E2ZAB3|A0A8E2ZAB3_RHIMP   QECQDKKLECVYKNHKAECKCPDDHECSRQPAKDSCSEEDNGKCQSSGQRCVMENGNAVC   600
tr|A0A8E2Z9N3|A0A8E2Z9N3_RHIMP   QECQDKKLECVYKNHKAECKCPDDHECSRQPAKDSCSEEDNGKCQSSGQRCVMENGKAVC   600
tr|A0A8E2Z9M6|A0A8E2Z9M6_RHIMP   QECQDKKLECVYKNHKAECKCPDDHECSREPAKDSCSEEDNGKCQSSGQRCVMENGNAVC   600
tr|A0A8E2Z9L6|A0A8E2Z9L6_RHIMP   QECQDKKLECVYKNHKAECKCPDDHECSRQPAKDSCSEEDNGKCQSSGQRCVMENGNAVC   600
tr|A0A8E2Z8G3|A0A8E2Z8G3_RHIMP   QECQDKKLECVYKNHKAECKCPDDHECSRQPAKDSCSEEDNGKCQSSGQRCVMENGKAVC   600
tr|A0A8E2Z7X1|A0A8E2Z7X1_RHIMP   QECQDKKLECVYKNHKAECKCPDDHECSREPAKDSCSEEDNGKCQSSGQRCVMENGNAVC   600
tr|A0A8E2Z7W9|A0A8E2Z7W9_RHIMP   QECQDKKLECVYKNHKAECKCPDDHECSREPAKDSCSEEDNGKCQSSGQRCVMENGNAVC   600
tr|A0A8E2Z7W5|A0A8E2Z7W5_RHIMP   QECQDKKLECVYKNHKAECKCPDDHECSRQPAKDSCSEEDNGKCQSSGQRCVMENGNAVC   600
tr|A0A8E2Z7V4|A0A8E2Z7V4_RHIMP   QECQDKKLECVYKNHKAECKCPDDHECSREPAKDSCSEEDNGKCQSSGQRCVMENGNAVC   600
tr|A0A8E2Z7U7|A0A8E2Z7U7_RHIMP   QECQDKKLECVYKNHKAECKCPDDHECSREPAKDSCSEEDNGKCQSSGQRCVMENGKAVC   600
tr|A0A8E2Z7U5|A0A8E2Z7U5_RHIMP   QECQDKKLECVYKNHKAECKCPDDHECSREPAKDSCSEEDNGKCQSSGQRCVMENGNAVC   600
tr|A0A8E2Z7U4|A0A8E2Z7U4_RHIMP   QECQDKKLECVYKNHKAECKCPDDHECSREPAKDSCSEEDNGKCQSSGQRCVMENGNAVC   600
tr|A0A8E2Z7T7|A0A8E2Z7T7_RHIMP   QECQDKKLECVYKNHKAECKCPDDHECSRQPAKDSCSEEDNGKCQSSGQRCVMENGKAVC   600
tr|A0A8E2Z7S4|A0A8E2Z7S4_RHIMP   QECQDKKLECVYKNHKAECKCPDDHECSREPAKDSCSEEDNGKCQSSGQRCVMENGKAVC   600
tr|A0A6M2CI36|A0A6M2CI36_RHIMP   QECQDKKLECVYKNHKAECKCPDDHECSREPAKDSCSEEDNGKCQSSGQRCVMENGNAVC   600
tr|Q5XKV2|Q5XKV2_RHIMP           ------------------------------------------------------------   264


tr|Q5ER80|Q5ER80_RHIMP           --------------------------------------------------   264
tr|A0A8E2Z927|A0A8E2Z927_RHIMP   KEKSDATTASTTTTKAKDKDPDPEKSSAAAVSATGLLLLLAATSVTAASL   650
tr|A0A8E2Z7V6|A0A8E2Z7V6_RHIMP   KEKSEATTAATTTTKAKDKDPDPGKSSAAAVSATGLLLLLAATSVTVASL   650
tr|A0A8E2Z7X7|A0A8E2Z7X7_RHIMP   KEKSEATTAATTTTKAKDKDPDPGKSSAAAVSATGLLLLLAATSVTVASL   650
tr|A0A8E3CL24|A0A8E3CL24_RHIMP   KEKSEATTAATTTTKAKDKDPDPGKSSAAAVSATGLLLLLAATSVTVASL   650
tr|A0A8E2Z8E9|A0A8E2Z8E9_RHIMP   KEKSEATTAATTTTKAKDKDPDPGKSSAAAVSATGLLLLLAATSVTVASL   650
tr|A0A8E2Z916|A0A8E2Z916_RHIMP   KEKSEATTAATTTTKAKDKDPDPGKSSAAAVSATGLLLLLAATSVTVASL   650
tr|A0A8E2Z7Z1|A0A8E2Z7Z1_RHIMP   KEKSEATTAATTTTKAKDKDPDPGKSSAAAVSATGLLLLLAATSVTAASL   650
tr|A0A8E3CLH7|A0A8E3CLH7_RHIMP   KEKSEATTAATTTTKAKDKDPDPGKSSAAAVSATGLLLLLAATSVTVASL   650
tr|A0A8E3CK42|A0A8E3CK42_RHIMP   KEKSEATTAATTTTKAKDKDPDPGKSSAAAVSATGLLLLLAATSVTVASL   650
tr|C4NAN0|C4NAN0_RHIMP           KEKSEATTAATTTTKAKDKDPDPGKSSA----------------------   609
tr|E5KCH6|E5KCH6_RHIMP           KEKSEATTAATTTTKAKDKDPDPGKSSAAAVSATGLLLLLAATSVTVASL   650
tr|Q5XKU6|Q5XKU6_RHIMP           --------------------------------------------------   264
tr|Q5ER81|Q5ER81_RHIMP           --------------------------------------------------   264
tr|Q5ER93|Q5ER93_RHIMP           --------------------------------------------------   264
tr|Q5ER89|Q5ER89_RHIMP           --------------------------------------------------   264
tr|A0A8E2Z7V2|A0A8E2Z7V2_RHIMP   KEKSEATTAATTTTKAKDKDPDPGKSSAAAVSATGLLLLLAATSVTVASL   650
tr|Q5ER77|Q5ER77_RHIMP           --------------------------------------------------   264
tr|Q5ER78|Q5ER78_RHIMP           --------------------------------------------------   264
tr|Q5ER69|Q5ER69_RHIMP           --------------------------------------------------   264
tr|Q5XKU9|Q5XKU9_RHIMP           --------------------------------------------------   264
tr|Q9Y0V1|Q9Y0V1_RHIMP           --------------------------------------------------   569
tr|Q5XKU7|Q5XKU7_RHIMP           --------------------------------------------------   264
tr|Q5I6P2|Q5I6P2_RHIMP           --------------------------------------------------   264
tr|Q5ER92|Q5ER92_RHIMP           --------------------------------------------------   264
tr|Q5ER88|Q5ER88_RHIMP           --------------------------------------------------   264
```

```
tr|Q5ER82|Q5ER82_RHIMP               --------------------------------------------------        264
tr|A0A8E3CK52|A0A8E3CK52_RHIMP       KEKSEATTAATATTKAKDEDPDPGKSSAAAVSATGLLLLLAATSVTAASL        650
tr|A0A8E2ZC71|A0A8E2ZC71_RHIMP       KEKSEATTAATATTKAKDEDPDPGKSSAAAVSATGLLLLLAATSVTAASL        650
tr|A0A8E2Z937|A0A8E2Z937_RHIMP       KEKSEATTASTTTTKAKDKDPDPGKSSAAAVSATGLLLLLAATSVTAASL        650
tr|A0A8E2Z8F5|A0A8E2Z8F5_RHIMP       KEKSDATTASTTTTKAKDKDPDPEKSSAAAVSATGLLLLLAATSVTAASL        650
tr|A0A8E3CHP7|A0A8E3CHP7_RHIMP       KEKSDATTASTTTTKAKDKDPDPEKSSAAAVSATGLLLLLAATSVTAASL        650
tr|E5KCI4|E5KCI4_RHIMP               KEKSDATTASTTTTKAKDKDPDPEKSSAAAVSATGLLLLLAATSVTAASL        650
tr|A0A8E3CM27|A0A8E3CM27_RHIMP       KEKSDATTASTTTTKAKDKDPDPEKSSAAAVSATGLLLLLAATSVTAASL        650
tr|Q5XKU8|Q5XKU8_RHIMP               --------------------------------------------------        264
tr|Q5I6P1|Q5I6P1_RHIMP               --------------------------------------------------        264
tr|Q5ER85|Q5ER85_RHIMP               --------------------------------------------------        264
tr|Q5ER75|Q5ER75_RHIMP               --------------------------------------------------        264
tr|Q5ER64|Q5ER64_RHIMP               --------------------------------------------------        264
tr|E5KCI2|E5KCI2_RHIMP               KEKSEATTAATTTTKAKDKDPDPGKSSAAAVSATGLLLLLAATSVTAASL        650
tr|A0A8E2ZDL2|A0A8E2ZDL2_RHIMP       KEKSDATTASTTTTKAKDKDPDPEKSSAAAVSATGLLLLLAATSVTAASL        650
tr|A0A8E2ZDK4|A0A8E2ZDK4_RHIMP       KEKSDATTASTTTTKAKDKDPDPEKSSAAAVSATGLLLLLAATSVTVASL        650
tr|E5KCI6|E5KCI6_RHIMP               KEKSDATTASTTTTKAKDKDPDPEKSSAAAVSATGLLLLLAATSVTAASL        650
tr|E5KCI5|E5KCI5_RHIMP               KEKSEATTAATTTTKAKDKDPDPGKSSAAAVSATGLLLLLAATSVTVASL        650
tr|E5KCI1|E5KCI1_RHIMP               KEKSEATTAATTTTKAKDKDPDPGKSSAAAVSATGLLLLLAATSVTAASL        650
tr|E5KCI0|E5KCI0_RHIMP               KEKSEATTAATTTTKAKDKDPDPGKSSAAAVSATGLLLLLAATSVTVASL        650
tr|E5KCH9|E5KCH9_RHIMP               KEKSEATTAATTTTKAKDKDPDPGKSSAAAVSATGLLLLLAATSVTAASL        650
tr|E5KCH3|E5KCH3_RHIMP               KEKSDATTASTTTTKAKDKDPDPEKSSAAAVSATGLLLLLAATSVTAASL        650
tr|B6CNU3|B6CNU3_RHIMP               KEKSDATTASTTTTKAKDKDPDPGKSSA----------------------        608
tr|A0A8E3CM42|A0A8E3CM42_RHIMP       KEKSDATTASTTTTKAKDKDPDPEKSSAAAVSATGLLLLLAATSVTAASL        650
tr|A0A8E3CM37|A0A8E3CM37_RHIMP       KEKSEATTAATATTKAKDKDPDPGKSSAAAVSATGLLLLLAATSVTAASL        650
tr|A0A8E3CM32|A0A8E3CM32_RHIMP       KEKSEATTASTTTTKAKDKDPDPGKSSAAAVSATGLLLLLAATSVTAASL        650
tr|A0A8E3CLI5|A0A8E3CLI5_RHIMP       KEKSEATTAATATTKAKNKDPDPGKSSAAAVSATGLLLLLAATSVTAASL        650
tr|A0A8E3CLI1|A0A8E3CLI1_RHIMP       KEKSEATTAATATTKAKDKDPDPGKSSAAAVSATGLLLLLAATSVTAASL        650
tr|A0A8E3CK47|A0A8E3CK47_RHIMP       KEKSEATTAATTTTKAKDKDPDPEKSSAAAVSATGLLLLLAATSVTAASL        650
tr|A0A8E3CK39|A0A8E3CK39_RHIMP       KEKSEATTAATTTTKAKDKDPDPGKSSAAAVSATGLLLLLAATSVTAASL        650
tr|A0A8E3CHR0|A0A8E3CHR0_RHIMP       KEKSDATTASTTTTKAKDKDPDPEKSSAAAVSATGLLLLLAATSVTAASL        650
tr|A0A8E3CHQ5|A0A8E3CHQ5_RHIMP       KEKSEATTAATATTKAKDKDPDPGKSSAAAVSATGLLLLLAATSVTAASL        650
tr|A0A8E2ZDN2|A0A8E2ZDN2_RHIMP       KEKSEATTAATATTKAKDEDPDPGKSSAAAVSATGLLLLLAATSVTAASL        650
tr|A0A8E2ZC62|A0A8E2ZC62_RHIMP       KEKSEATTAATTTTKAKDKDPDPGKSSAAAVSATGLLLLLAATSVTAASL        650
tr|A0A8E2ZC54|A0A8E2ZC54_RHIMP       KEKSEATTAATTTTKAKDKDPDPGKSSAAAVSATGLLLLLAATSVTVASL        650
tr|A0A8E2ZC45|A0A8E2ZC45_RHIMP       KEKSEATTASTTTTKAKDKDPDPEKSSAAAVSATGLLLLLAATSVTAASL        650
tr|A0A8E2ZAC1|A0A8E2ZAC1_RHIMP       KEKSEATTAATATTKAKNKDPDPGKSSAAAVSATGLLLLLAATSVTAASL        650
tr|A0A8E2ZAB3|A0A8E2ZAB3_RHIMP       KEKSEATTAATTTTKAKDKDPDPGKSSAAAVSATGLLLLLAATSVTAASL        650
tr|A0A8E2Z9N3|A0A8E2Z9N3_RHIMP       KEKSEATTAATTTTKAKDKDPDPGKSSAAAVSATGLLLLLAATSVTAASL        650
tr|A0A8E2Z9M6|A0A8E2Z9M6_RHIMP       KEKSEATTAATTTTKAKDKDPDPEKSSAAAVSATGLLLLLAATSVTAASL        650
tr|A0A8E2Z9L6|A0A8E2Z9L6_RHIMP       KEKSDATTASTTTTKAKDKDPDPGKSSAAAVSATGLLLLLAATSVTVASL        650
tr|A0A8E2Z8G3|A0A8E2Z8G3_RHIMP       KEKSEATTAATTTTKAKDKDPDPEKSSAAAVSATGLLLLLAATSVTAASL        650
tr|A0A8E2Z7X1|A0A8E2Z7X1_RHIMP       KEKSEATTASTATTKAKDKDPDPEKSSAAAVSATGLLLLLAATSVTAASL        650
tr|A0A8E2Z7W9|A0A8E2Z7W9_RHIMP       KEKSDATTASTTTTKAKDKDPDPEKSSAAAVSATGLLLLLAATSVTAASL        650
tr|A0A8E2Z7W5|A0A8E2Z7W5_RHIMP       KEKSEATTAATATTKAKDKDPDPGKSSAAAVSATGLLLLLAATSVTAASL        650
tr|A0A8E2Z7V4|A0A8E2Z7V4_RHIMP       KEKSEATTAATTTTKAKNKDPDPEKSSAAAVSATGLLLLLAATSVTAASL        650
tr|A0A8E2Z7U7|A0A8E2Z7U7_RHIMP       KEKSEATTAATTTTKAKDKDPDPGKSSAAAVSATGLLLLLAATSVTVASL        650
tr|A0A8E2Z7U5|A0A8E2Z7U5_RHIMP       KEKSDATTAATTTTKAKDKDPDPEKSSAAAVSATGLLLLLAATSVTVASL        650
tr|A0A8E2Z7U4|A0A8E2Z7U4_RHIMP       KEKSDATTAATTTTKAKDKDPDPEKSSAAAVSATGLLLLLAATSVTVASL        650
tr|A0A8E2Z7T7|A0A8E2Z7T7_RHIMP       KEKSDATTASTTTTKAKDKDPDPEKSSAAAVSATGLLLLLAATSVTAASL        650
tr|A0A8E2Z7S4|A0A8E2Z7S4_RHIMP       KEKSEATTAATTTTKAKDKDPDPEKSSAAAVSATGLLLLLAATSVTAASL        650
tr|A0A6M2CI36|A0A6M2CI36_RHIMP       KEKSDATTASTTTTKAKDKDPDPEKSSAAAVSATGLLLLLAATSVTAASL        650
tr|Q5XKV2|Q5XKV2_RHIMP               --------------------------------------------------        264
```

S4 Data. Predicted T-cell epitopes (MHC I and MHC II) in Q38-95. The predicted $logIC_{50}$ (M) and IC_{50} (nM) values are disclosed with confidence of prediction max = 1. The analysis was conducted using MHCPred v.2. (http://www.ddg-pharmfac.net/mhcpred/MHCPred/).

The HLA allele used in the test is: A0201			
The query sequence			
M A C A T L K R T H D W D P L H S P N G R S P K P S P F G E V P P K S S P L E S G S P S A T P P A S P T G L S P G G L L S P V R R D Q P L F T F R Q V G L I C E R M M K E R E S Q I R D E Y D H V L S A K L A E Q Y D T F V K F T Y D Q I Q K R F E G A T P S Y L S G G G S H K P F G S P S S P S S S A I A A A A A A A K R P S P F A E A V C P K Q L T F N T G S R P D S P P S M V L F T F K Q A L R E Q Y D A V L T N K L A E Q Y D A A A P S Y L S G G G S R V Q K G T V L C E C P W N Q H L V G D T C I S D C V D K K C H E			
Amino acid groups	Predicted - $logIC_{50}$ (M)	Predicted IC_{50} Value (nM)	Confidence of prediction (Max = 1)
DEYDHVLSA	7.4	39.81	0.89
VLFTFKQAL	7.285	51.88	1.00
GSPSATPPA	7.112	77.27	0.89
YLSGGGSRV	7.062	86.70	1.00
IRDEYDHVL	7.012	97.27	0.89
QYDTFVKFT	6.967	107.89	0.89
SMVLFTFKQ	6.904	124.74	0.89
KLAEQYDTF	6.854	139.96	1.00
HDWDPLHSP	6.832	147.23	0.78
FRQVGLICE	6.815	153.11	0.78
KLAEQYDAA	6.811	154.53	1.00
PWNQHLVGD	6.793	161.06	0.78
ASPTGLSPG	6.745	179.89	0.78
ALREQYDAV	6.732	185.35	1.00
SHKPFGSPS	6.662	217.77	0.89
YLSGGGSHK	6.652	222.84	0.89
KRFEGATPS	6.635	231.74	0.89
LSPGGLLSP	6.619	240.44	0.78
QKGTVLCEC	6.611	244.91	0.89
MMKERESQI	6.586	259.42	1.00
VKFTYDQIQ	6.584	260.62	0.78
AAAPSYLSG	6.581	262.42	0.89
VLTNKLAEQ	6.55	281.84	0.89
PLFTFRQVG	6.547	283.79	0.89
AAPSYLSGG	6.504	313.33	0.89

GLLSPVRRD	6.504	313.33	0.89
AAKRPSPFA	6.452	353.18	1.00
QYDAAAPSY	6.428	373.25	0.78
RVQKGTVLC	6.419	381.07	1.00
PSATPPASP	6.417	382.82	0.78
RRDQPLFTF	6.417	382.82	0.89
AEAVCPKQL	6.405	393.55	0.89
PSMVLFTFK	6.401	397.19	0.78
LSAKLAEQY	6.397	400.87	0.78
YDAVLTNKL	6.397	400.87	0.89
DWDPLHSPN	6.386	411.15	0.78
GLSPGGLLS	6.37	426.58	1.00
AEQYDTFVK	6.362	434.51	0.78
SPFGEVPPK	6.359	437.52	0.89
KQLTFNTGS	6.345	451.86	1.00
SRPDSPPSM	6.332	465.59	0.89
PFGEVPPKS	6.326	472.06	0.89
SYLSGGGSH	6.324	474.24	0.78
AVLTNKLAE	6.321	477.53	0.89
KSSPLESGS	6.32	478.63	0.89
PLESGSPSA	6.316	483.06	1.00
QHLVGDTCI	6.291	511.68	0.89
RFEGATPSY	6.286	517.61	0.78
RMMKERESQ	6.266	542.00	0.89
LSGGGSHKP	6.254	557.19	0.78
ATPSYLSGG	6.247	566.24	0.89
GSRPDSPPS	6.247	566.24	0.89
ACATLKRTH	6.243	571.48	0.78
KRPSPFAEA	6.224	597.04	0.89
YDAAAPSYL	6.22	602.56	0.89
YDHVLSAKL	6.18	660.69	0.89
GATPSYLSG	6.179	662.22	0.89
ECPWNQHLV	6.169	677.64	0.78
FTFKQALRE	6.167	680.77	0.89
EYDHVLSAK	6.166	682.34	0.67

PFAEAVCPK	6.166	682.34	0.78
ATPPASPTG	6.158	695.02	0.89
TFVKFTYDQ	6.144	717.79	0.78
FKQALREQY	6.141	722.77	0.78
KRTHDWDPL	6.14	724.44	0.89
FTYDQIQKR	6.136	731.14	1.00
FVKFTYDQI	6.134	734.51	1.00
QIRDEYDHV	6.111	774.46	1.00
FTFRQVGLI	6.103	788.86	1.00
SYLSGGGSR	6.1	794.33	0.89
VLSAKLAEQ	6.085	822.24	0.89
VCPKQLTFN	6.048	895.36	0.78
EQYDAVLTN	6.048	895.36	0.78
LAEQYDTFV	6.043	905.73	1.00
PKSSPLESG	6.039	914.11	0.78
SPGGLLSPV	6.037	918.33	1.00
YDTFVKFTY	6.026	941.89	0.78
AAAAKRPSP	6.015	966.05	0.89
ESGSPSATP	6.014	968.28	0.67
LTNKLAEQY	6.007	984.01	0.89
RSPKPSPFG	6.005	988.55	0.78
VLCECPWNQ	6.001	997.70	0.89
RDQPLFTFR	5.993	1016.25	0.89
TVLCECPWN	5.992	1018.59	0.89
LREQYDAVL	5.98	1047.13	0.89
AKRPSPFAE	5.978	1051.96	0.78
LVGDTCISD	5.954	1111.73	0.89
TFKQALREQ	5.95	1122.02	0.78
QIQKRFEGA	5.949	1124.60	1.00
KFTYDQIQK	5.93	1174.90	0.78
PSSSAIAAA	5.908	1235.95	0.89
TFNTGSRPD	5.905	1244.51	0.78
LKRTHDWDP	5.899	1261.83	0.78
ISDCVDKKC	5.873	1339.68	0.89
GEVPPKSSP	5.87	1348.96	0.78

VRRDQPLFT	5.867	1358.31	0.89
SRVQKGTVL	5.853	1402.81	0.89
MVLFTFKQA	5.846	1425.61	1.00
EVPPKSSPL	5.836	1458.81	0.89
GRSPKPSPF	5.823	1503.14	0.89
FAEAVCPKQ	5.81	1548.82	0.89
RTHDWDPLH	5.785	1640.59	0.89
QVGLICERM	5.764	1721.87	1.00
WDPLHSPNG	5.754	1761.98	0.78
GGGSHKPFG	5.74	1819.70	0.89
KPFGSPSSP	5.736	1836.54	0.89
AIAAAAAAA	5.729	1866.38	1.00
QYDAVLTNK	5.69	2041.74	0.78
LHSPNGRSP	5.687	2055.89	0.78
FEGATPSYL	5.679	2094.11	0.89
AEQYDAAAP	5.679	2094.11	0.78
DTFVKFTYD	5.674	2118.36	0.89
ERMMKERES	5.666	2157.74	0.78
LAEQYDAAA	5.665	2162.72	1.00
LSGGGSRVQ	5.664	2167.70	0.78
KPSPFGEVP	5.654	2218.20	0.89
YDQIQKRFE	5.646	2259.44	0.78
HVLSAKLAE	5.645	2264.64	0.89
CECPWNQHL	5.635	2317.39	0.89
FNTGSRPDS	5.605	2483.13	0.89
ATLKRTHDW	5.598	2523.48	0.89
CPKQLTFNT	5.59	2570.40	1.00
SPTGLSPGG	5.582	2618.18	0.89
DSPPSMVLF	5.575	2660.73	0.89
TFRQVGLIC	5.575	2660.73	0.89
CISDCVDKK	5.568	2703.96	0.89
PSPFGEVPP	5.566	2716.44	0.78
KERESQIRD	5.566	2716.44	0.78
EQYDAAAPS	5.56	2754.23	0.89
AVCPKQLTF	5.557	2773.32	1.00

GTVLCECPW	5.556	2779.71	0.89
GLICERMMK	5.545	2851.02	0.89
DAVLTNKLA	5.544	2857.59	1.00
PKPSPFGEV	5.541	2877.40	0.89
GSHKPFGSP	5.523	2999.16	0.78
PASPTGLSP	5.502	3147.75	0.89
PVRRDQPLF	5.495	3198.90	1.00
PSYLSGGGS	5.458	3483.37	0.89
PDSPPSMVL	5.454	3515.60	0.89
TCISDCVDK	5.447	3572.73	0.78
SPKPSPFGE	5.443	3605.79	0.89
LTFNTGSRP	5.439	3639.15	0.89
PFGSPSSPS	5.437	3655.95	0.89
SAKLAEQYD	5.41	3890.45	0.89
DQPLFTFRQ	5.402	3962.78	0.89
AAAKRPSPF	5.392	4055.09	1.00
VQKGTVLCE	5.389	4083.19	0.89
GDTCISDCV	5.37	4265.80	0.89
QLTFNTGSR	5.351	4456.56	1.00
SAIAAAAAA	5.336	4613.18	1.00
SATPPASPT	5.334	4634.47	1.00
GSPSSPSSS	5.329	4688.13	0.89
DHVLSAKLA	5.324	4742.42	0.89
VGDTCISDC	5.323	4753.35	1.00
IAAAAAAAK	5.311	4886.52	0.89
TLKRTHDWD	5.305	4954.50	0.89

S5 Data. Sequence alignment. Sequence alignment using the EMBOSS Needle optimal global alignment of two sequences using the Needleman-Wunsch algorithm (https://www.ebi.ac.uk/Tools/psa/).

```
########################################
# Program: needle
# Rundate: Sun 14 May 2023 13:36:26
# Commandline: needle
#    -auto
#    -stdout
#    -asequence emboss_needle-I20230514-133604-0160-93212831-p1m.asequence
#    -bsequence emboss_needle-I20230514-133604-0160-93212831-p1m.bsequence
#    -datafile EBLOSUM62
#    -gapopen 10.0
#    -gapextend 0.5
#    -endopen 10.0
#    -endextend 0.5
#    -aformat3 pair
#    -sprotein1
#    -sprotein2
# Align_format: pair
# Report_file: stdout
########################################

#=======================================
#
# Aligned_sequences: 2
# 1: MT241515.1
# 2: AF150891.2
# Matrix: EBLOSUM62
# Gap_penalty: 10.0
# Extend_penalty: 0.5
#
# Length: 579
# Identity:      40/579 ( 6.9%)
# Similarity:    70/579 (12.1%)
# Gaps:         428/579 (73.9%)
# Score: 24.0
#
#
#=======================================

MT241515.1         1 --------------------------------------------------      0

AF150891.2         1 MRGIALFVAAVSLIVEGTAESSICSDFGNEFCRNAECEVVPGAEDDFVCK     50

MT241515.1         1 --------------------------------------------------      0

AF150891.2        51 CPRDNMYFNAAEKQCEYKDTCKTRECSYGRCVESNPSKGSCVCERSDDLT    100

MT241515.1         1 --------------------------------------------------      0

AF150891.2       101 LQCKIKNDYATDCRNRGGTAKLRTDGFIGATCDCGEWGAMNKTTRNCVPT    150

MT241515.1         1 ------MACATL----------KRTHDWDPLHSPSGRSPKRRRCMPLSP-     33
                           :.|..|          :....|:..:. |...|....|.|.||
AF150891.2       151 TCLRPDLTCKDLCEKNLLQRDSRCCQGWNTANC-SAAPPADSYCSPGSPK    199

MT241515.1        34 ------------------------PPTRAHQIDPSPFGD------VPPKL     53
                                             ...:|::. ..|.|.      :..|.
AF150891.2       200 GPDGQCKNACRTKEAGFVCKHGCRSTDKAYEC-TCPSGSTVAEDGITCKS    248

MT241515.1        54 TSEEIAASIREEMR-------RLQRRKQLCFQGADPESQHTSG-------     89
                     .|..::.::.::..       |:|:...||   ..|.:||..|
AF150891.2       249 ISYTVSCTVEQKQTCRPTEDCRVQKGTVLC---ECPWNQHLVGDTCISDC    295

MT241515.1        90 LSSPVHRDQPLFTFRQVGLICERMMKERESKIREEYDHV-LSTKLAEQYD    138
```

```
                     :....|.:     |...|:...|.......|.|:...:| ::.:|..:|.
AF150891.2       296 VDKKCHEE-----FMDCGVYMNRQSCYCPWKSRKPGPNVNINERLLNEYY     340

MT241515.1       139 TFVKFT----YDQIQ-KRFE----GATPSYLS------------------     161
                     ..|.||    :|... ||:|    ||..:.:.
AF150891.2       341 YTVSFTPNISFDSDHCKRYEDRVLGAIRTSIGKEVFKVEILNCTQDIKAR     390

MT241515.1       162 --------------------------------------------------     161

AF150891.2       391 LIAEKPLSKYVLRKLQACEHPIGEWCMMYPKLLIKKNSATEIEEENLCDS     440

MT241515.1       162 --------------------------------------------------     161

AF150891.2       441 LLKNQEAAYKGQNKCVKVDNLFWFQCADGYTTTYEMTRGRLRRSVCKAGV     490

MT241515.1       162 --------------------------------------------------     161

AF150891.2       491 SCNENEQLECANKGQICVYENGKANCQCPPDTKPGEIGCIERTTCNPKEI     540

MT241515.1       162 -----------------------------     161

AF150891.2       541 QECQDKKLECVYKNHKAECKCPDDHECSR     569
```

Supplementary audio files

S1 Audio file. SUB.wav.

S2 Audio file. BM95.wav.

S3 Audio file. SUB-BM95 interactions.wav.

S4 Audio file. Cadential.wav**.**

S5 Audio file. Harmonization.wav.
