# Supplementary material for: Modelling protein-protein interactions for the design of vaccine chimeric antigens with protective epitopes
Source: PLoS One. 2025 Feb 10;20(2):e0318439. doi: 10.1371/journal.pone.0318439 (PMC11809815; doi:10.1371/journal.pone.0318439)
Supplement: S1 raw images — (PDF) [file pone.0318439.s003.pdf]

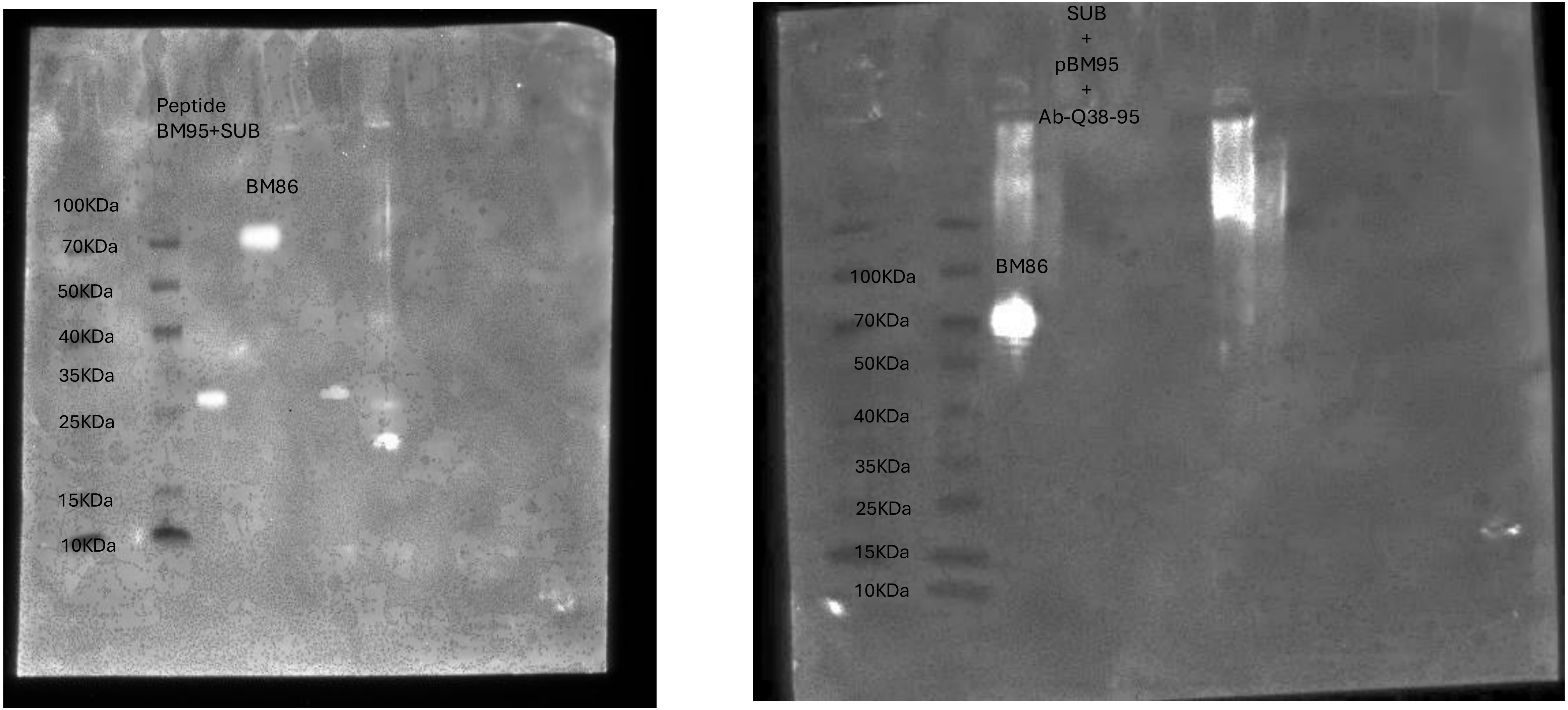
Peptide
BM95+SUB
BM86
100KDa
70KDa
50KDa
40KDa
35KDa
25KDa
15KDa
10KDa
SUB
+
pBM95
+
Ab-Q38-95
BM86
100KDa
70KDa
50KDa
40KDa
35KDa
25KDa
15KDa
10KDa

Anti-BM86

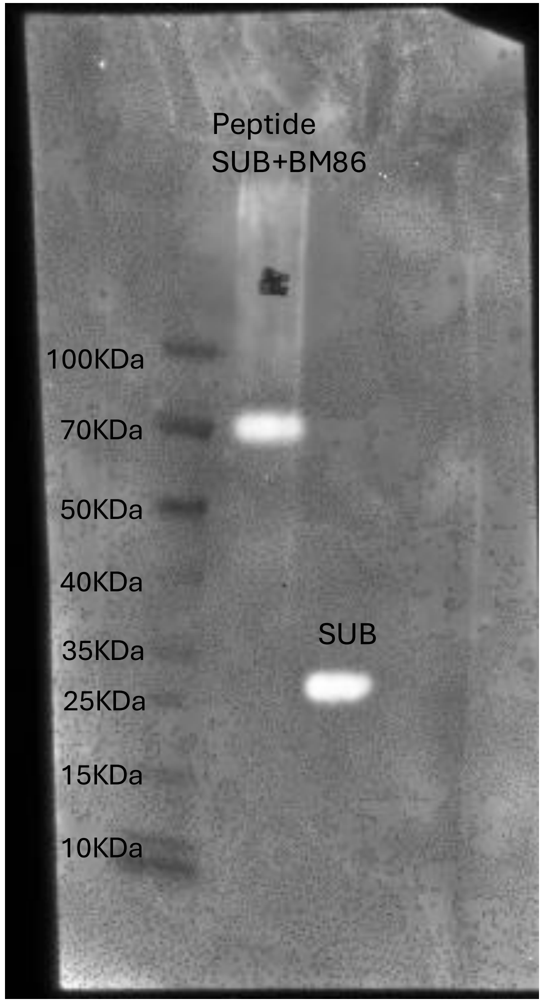

Anti-SUB

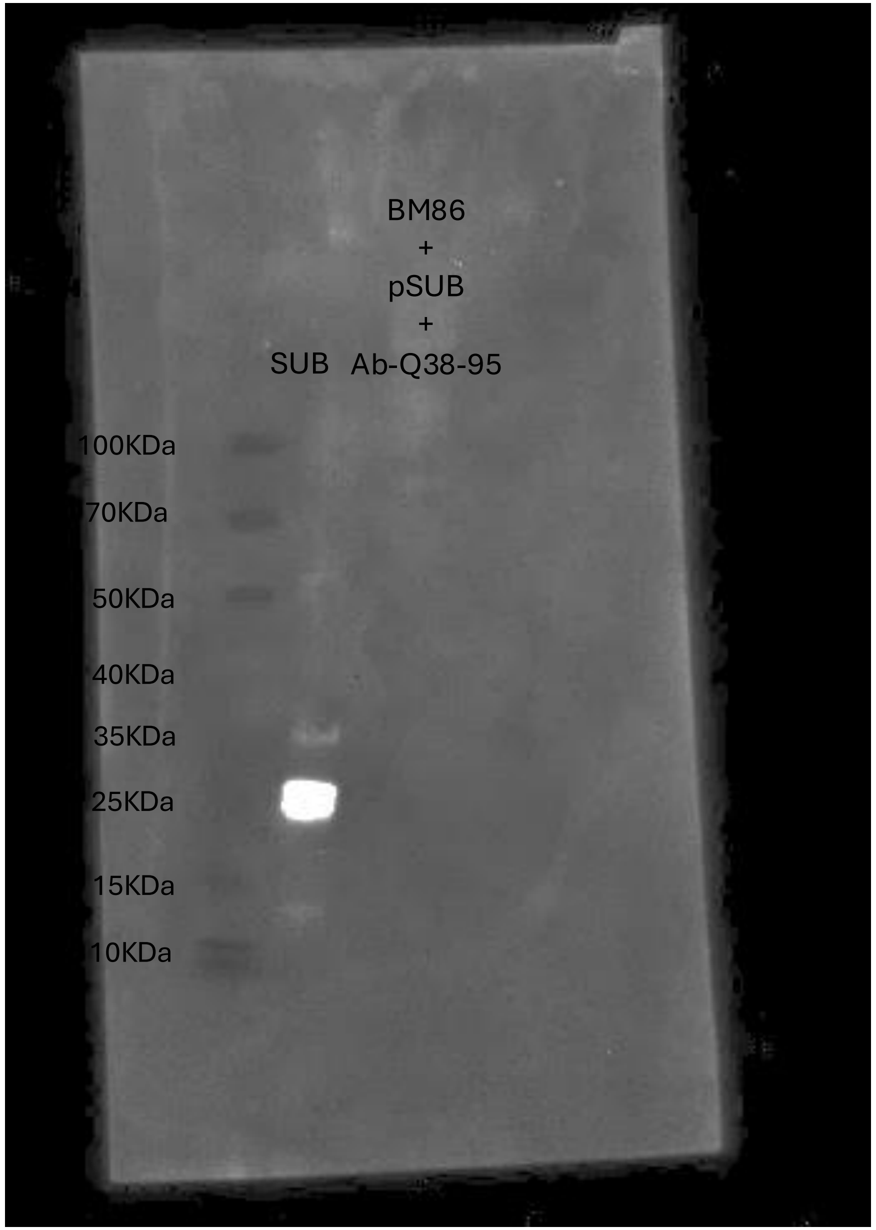

Anti-Q38-95 antibody-mediated inhibition of SUB-BM95 protein-protein interactions was analyzed by Western blot using sera from rabbits immunized with Q38-95 and controls. The SUB-pBM95 and BM86-pSUB interactions were inhibited by anti-Q38-95 antibodies.
